# Synthesis of 3,4‐Dihydro‐2*H*‐Pyrroles from Ketones, Aldehydes, and Nitro Alkanes via Hydrogenative Cyclization

**DOI:** 10.1002/chem.202201307

**Published:** 2022-07-06

**Authors:** Barbara Klausfelder, Patricia Blach, Niels de Jonge, Rhett Kempe

**Affiliations:** ^1^ Anorganische Chemie II Catalyst Design Sustainable Chemistry Centre University of Bayreuth Universitätsstraße 30 95440 Bayreuth Germany; ^2^ INM - Leibniz Institute for New Materials Campus D2 2 66123 Saarbrücken Germany; ^3^ Department of Physics Saarland University Campus D2 2 66123 Saarbrücken Germany

**Keywords:** catalysis, hydrogenation, multicomponent reaction, N-heterocycles, nickel

## Abstract

Syntheses of N‐heterocyclic compounds that permit a flexible introduction of various substitution patterns by using inexpensive and diversely available starting materials are highly desirable. Easy to handle and reusable catalysts based on earth‐abundant metals are especially attractive for these syntheses. We report here on the synthesis of 3,4‐dihydro‐2*H*‐pyrroles via the hydrogenation and cyclization of nitro ketones. The latter are easily accessible from three components: a ketone, an aldehyde and a nitroalkane. Our reaction has a broad scope and 23 of the 33 products synthesized are compounds which have not yet been reported. The key to the general hydrogenation/cyclization reaction is a highly active, selective and reusable nickel catalyst, which was identified from a library of 24 earth‐abundant metal catalysts.

## Graphical abstract

**A highly active**, selective and reusable nickel catalyst has been developed and employed for the synthesis of 3,4‐dihydro‐2*H*‐pyrroles via hydrogenation and subsequent cyclization of nitro ketones. The catalyst was identified from a library of 24 heterogeneous catalysts. The key to our hydrogenation/cyclization reaction is the tolerance of aldehydes and ketones as functional groups and the ability to hydrogenate aliphatic nitro compounds efficiently.

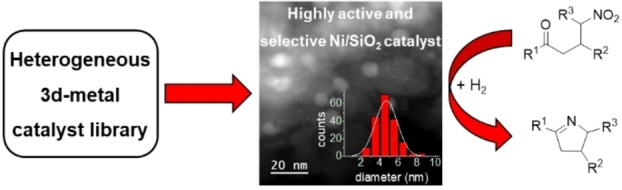

## Introduction

**Scheme 1:**
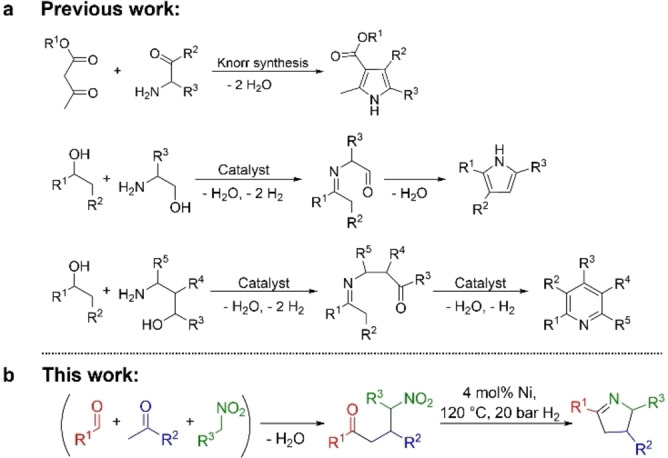
Synthesis of N‐heterocycles employing (catalytically generated) amino ketones or aldehydes: state of the art (a) and reaction introduced here (b).

The development of many catalytic reactions, broadly used in industry and organic synthesis today, was achieved up to a century ago through nanostructured 3d‐metal catalysts, often Raney nickel (Ni) or related materials.^[1]^ Then noble metal catalysts, especially well defined and easy to modify coordination compounds (homogeneous catalysts), took over and have been responsible for catalytic methodology developments.^[2]^ The renaissance of nanostructured 3d‐metals is indicative of high reactivity, reusability, unique selectivity profiles and easy handling of such catalysts,^[3]^ suggesting their use for the discovery of new reactions like the synthesis of N‐heterocyclic compounds. These are very important because of their numerous applications as fine and bulk chemicals.^[4]^ Among the many N‐heterocyclic compounds known, pyrrole derivatives are especially interesting since they figure prominently among drugs, catalysts, natural products and materials.^[4]^ There are classic protocols or name reactions for their synthesis^[4]^ and exciting activities regarding the development of catalytic protocols.^[5]^ An elegant way of synthesizing N‐heterocyclic compounds, among them pyrroles[^5d^, ^5f^] or pyridines,^[6]^ is the catalytic generation of amino ketones or aldehydes via the dehydrogenation^[7]^ of amino alcohols (Scheme 1a). A hydrogenative approach could be an alternative. Amino ketones or aldehydes are also accessible via reductive pathways by starting from an amine precursor that can be converted into an amine via hydrogenation, such as a nitro group or a nitrile.^[8]^ An advantage of the reductive approach is the simple assembly of the nitro ketones or aldehydes via classical aldol condensation^[9]^ and addition^[10]^ reactions (Scheme 1b).

We report here on the general synthesis of 3,4‐dihydro‐2*H*‐pyrroles via the hydrogenation and cyclization of nitro ketones (Scheme 1b). The latter are easily accessible from three components: a ketone, an aldehyde and a nitroalkane, permitting a four‐component 3,4‐dihydro‐2*H*‐pyrrole synthesis for the overall process, with water being the only by‐product. Our reaction has a broad scope and 23 of the 33 products synthesized are compounds which have not yet been reported. The key to the general hydrogenation/cyclization is a highly active and selective nanostructured and reusable 3d‐metal catalyst. Our catalyst is based on Ni, mediates nitrobenzene hydrogenation with less than 1 mol% Ni loading applying 10 bar hydrogen pressure at 40 °C. In addition, the catalyst tolerates ketones as functional groups and is able to hydrogenate nitro alkanes. We identified our catalyst from a library of 24 earth‐abundant metal catalysts where metal precursors and supports were varied by using nitrobenzene hydrogenation as a model reaction. The hydrogenation of nitroarenes employing 3d‐metal catalysts has been investigated intensively in recent years.^[11]^

## Results and Discussion

### Synthesis of the catalyst library and identification of the active catalyst

**Figure 1:**
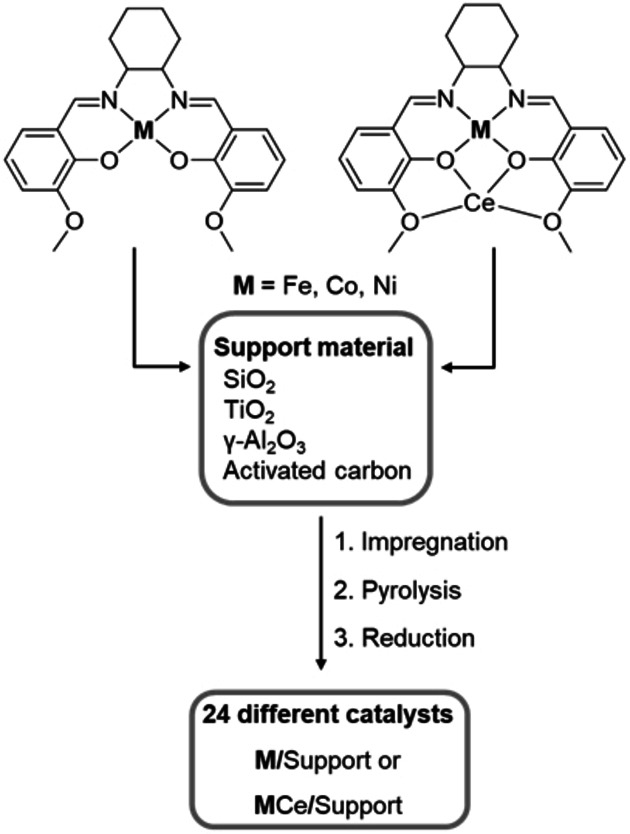
Preparation of the catalyst library. 1. Wet impregnation of four different porous support materials (SiO_2_, TiO_2_, γ‐Al_2_O_3_, activated carbon) with a solution of six different mono‐ or bimetallic salen complexes (metal precursors) in acetonitrile. 2. Pyrolysis of the impregnated materials under nitrogen atmosphere at 700 °C. 3. Reduction of the respective pyrolyzed materials under forming gas.

**Figure 2:**
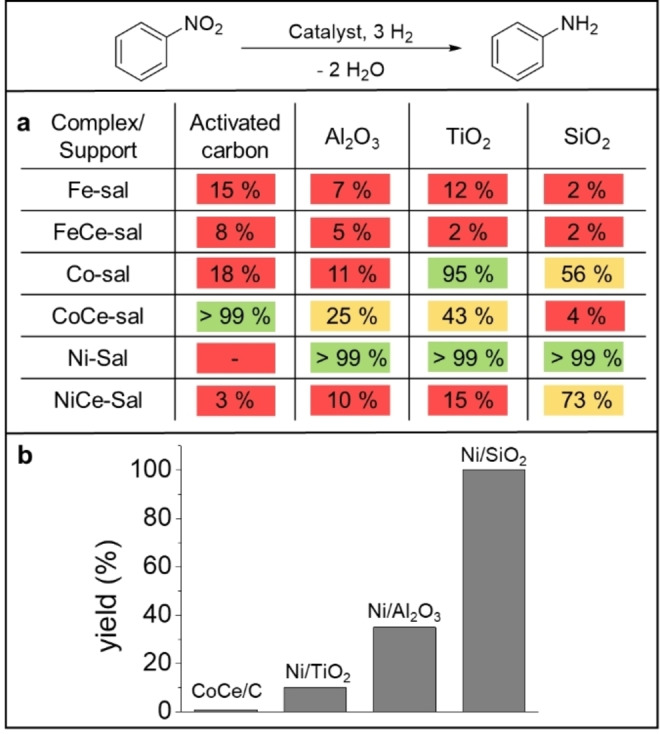
Screening for the most active catalyst. a) Screening of the catalyst library. General reaction conditions: 0.5 mmol nitrobenzene, 20 h, 3 mL solvent (for respective solvent, see Table S3); iron (Fe) catalysts: 10 mol% Fe, 60 bar H_2_, 120 °C; cobalt (Co) catalysts: 2.5 mol% Co, 50 bar H_2_, 110 °C; nickel (Ni) catalysts: 0.8 mol% Ni, 50 bar H_2_, 90 °C. Red <20 %, yellow 20–80 %, green >80 % aniline. b) Screening of the four most active catalysts. Reaction conditions: 0.5 mmol nitrobenzene, 0.8 mol% Ni or 0.8 mol% Co, 20 bar H_2_, 80 °C, 3 mL solvent 20 h. Yields were determined by gas chromatography (GC) using n‐dodecane as an internal standard.

We were initially interested in a highly active catalyst for the selective hydrogenation of nitro compounds. A library of 24 different mono‐ and bimetallic earth‐abundant catalyst systems was synthesized using a catalyst synthesis concept introduced by our group very recently.[^3d^, ^12^] The key is the use of salen ligand complexes as metal precursors. We varied the earth‐abundant metals Ni, cobalt (Co) and iron (Fe), in combination with cerium (Ce) or alone, and four commercially available support materials: SiO_2_, γ‐Al_2_O_3_, TiO_2_, and activated carbon (Figure 1). The supports were impregnated with a solution of the respective mono‐ or bimetallic salen precursor complex (M‐sal or MCe‐sal; See Table S1 and Figure S1 and S2 for their characterization.) in acetonitrile, followed by pyrolysis at 700 °C in a constant nitrogen stream and reduction under forming gas (N_2_/H_2_) at 550 °C. The catalytic activity of the catalysts (for characterization of the catalyst library, see Figure S3–12 and Table S2) was compared using the hydrogenation of nitrobenzene to aniline as a model reaction. Figure 2a outlines the activity of all catalysts regarding the aniline formation under the conditions given. Only a low catalytic activity was discovered for all Fe‐based catalysts, although relatively harsh reaction conditions were applied (10 mol% Fe, 120 °C, 60 bar H_2_). Good yields of aniline could be observed for selected Co catalysts (CoCe/C, Co/TiO_2_) at milder reaction conditions (2.5 mol% Co, 110 °C, 50 bar H_2_), while the other Co‐catalysts showed only moderate (Co/SiO_2_, CoCe/Al_2_O_3_, CoCe/TiO_2_) to low yields (Co/C, Co/Al_2_O_3_, CoCe/SiO_2_). All bimetallic Ni catalysts and the monometallic carbon‐supported Ni system revealed only low conversions for the reduction of nitrobenzene, with the exception of the NiCe/SiO_2_ catalyst (73 % yield). However, excellent yields up to 99 % aniline could be observed for the monometallic Ni catalysts supported on the oxidic supports (SiO_2_, γ‐Al_2_O_3_ or TiO_2_) with successful application of the mildest reaction conditions from the library: 0.8 mol% Ni, 90 °C and 50 bar H_2_ and for the CoCe/C catalyst. A further decrease of the reaction temperature and the hydrogen pressure (Figure 2b) revealed Ni/SiO_2_ to be the most active catalyst from the library. It still showed full conversion of nitrobenzene to aniline even at 0.8 mol% Ni, 40 °C and 10 bar H_2_ (Table S4). The hydrogenation of nitroarenes especially with nanostructured nickel catalysts is a well‐studied reaction.^[11]^ Our catalyst has similar activity to the most active nanostructured nickel catalysts described in the literature for the hydrogenation of nitro groups.^[13]^ We concluded that the combination of a silica support with Ni nanoparticles (Ni‐NPs) resulting from pyrolysis of the Ni‐salen complex and further reduction seems to be beneficial for high nitrobenzene hydrogenation activity and synthesized four SiO_2_‐based catalysts for comparison using different commercially available Ni salts as metal sources. Compared to our Ni/SiO_2_ catalyst (Table S5), all systems showed less or poor conversion of nitrobenzene at the same reaction conditions. The superior activity of our catalyst is probably due to the chosen Ni‐salen complex as metal precursor. Salen complexes are able to form a stabilizing N‐doped carbon shell around the NPs during pyrolysis and could lead to an atomic dispersion of the precursor complex on the surface of the support due to the sublimation before decomposition.[^3d^, ^12c^] In addition, we observed the formation of larger Ni NPs when other support materials like activated carbon or Al_2_O_3_ (See Figure S10) were used.

### Characterization of Ni/SiO_2_

**Figure 3:**
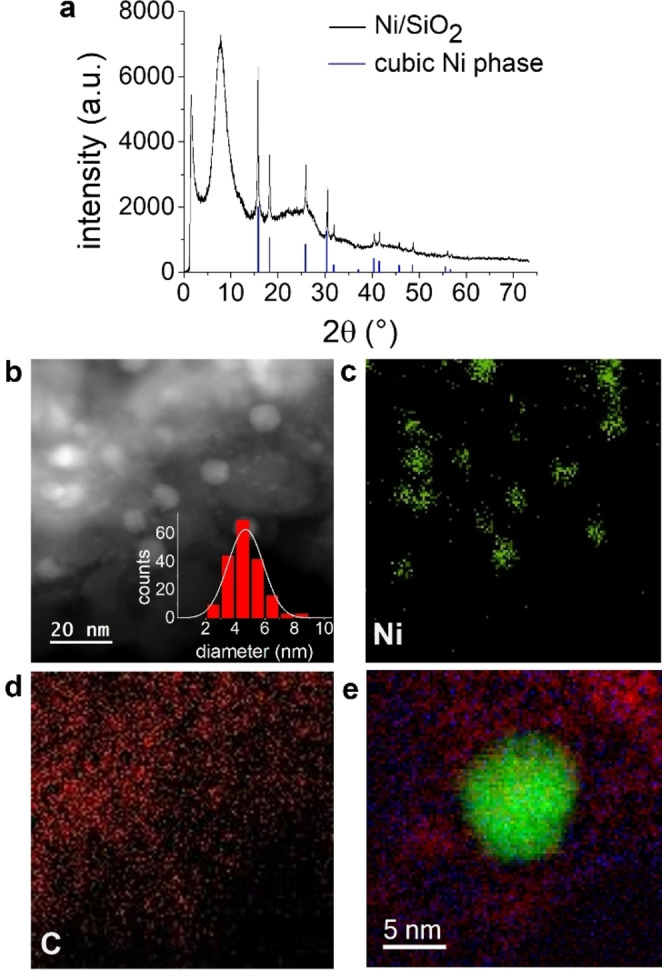
Characterization of Ni/SiO_2_. a) PXRD of the Ni/SiO_2_ catalyst showed the characteristic reflections of cubic Ni^0^ (blue; reference code 00–001‐1258) at 15.8 °, 18.2 °, 25.9 °, 30.3 °, 31.8 °, 37.1 °, 40.4 °, 41.5 °, 45.7 °, 48.6 °, 55.6 ° and 56.6 ° 2θ. b) HAADF‐STEM image of the Ni/SiO_2_ catalyst. The particle size distribution shown in the inset reveals homogeneously distributed nanoparticles with an average diameter of 4.7±1.2 nm (counted particles: 190). c), d) Representative EDX element maps of Ni (green) and C (red). e) Overlapped electron energy loss spectroscopy element maps of Ni (green), C (red) and N (blue) suggest the embedding of a Ni‐NP in a N‐doped carbon layer.

We performed transmission electron microscopy (TEM), high‐angle annular dark‐field scanning transmission electron microscopy (HAADF‐STEM), X‐ray photoelectron spectroscopy (XPS) or powder X‐ray diffraction (PXRD) to gain more insight into the structure of our Ni/SiO_2_ catalyst. A homogeneous distribution of NPs with an average diameter of 4.7 nm could be determined through TEM analysis (Figure 3b, Figure S13). The presence of a metallic cubic Ni phase indicative of Ni‐NPs in the material was confirmed by the XRD pattern (Figure 3a). Magnetic measurements (Figure S14) showed superparamagnetic behavior, which can also be attributed to the presence of Ni‐NPs. The expected lines for oxygen (O), silicon (Si), carbon (C), Ni and traces of nitrogen (N) were identified from the XPS survey spectrum of the catalyst (Figure S15). In addition, the detailed analysis of the Ni 2p region (Figure S16) revealed the presence of both metallic Ni^0^ (binding energy ∼853 eV) and Ni^2+^ (binding energy ∼855 eV), at a ratio of approximately 1.1 to 1.0. However, handling in air could partially oxidize the Ni^0^ on the surface to surface‐oxidized metallic Ni‐NPs. The presence of Ni‐NPs on the SiO_2_ support was again confirmed using HAADF‐STEM in combination with energy‐dispersive X‐ray (EDX) element maps (for Ni (green) and carbon (C, red), see Figures 3c and d; for silicon (Si) and oxygen (O), see Figure S17). Furthermore, the EDX element map of C (Figure 3d) taken in the same image section as the element map of Ni (Figure 3c) shows the distribution of carbon throughout the support material. Electron energy loss spectroscopy (EELS) in the region of a single NP was conducted to analyze the near environment of one Ni‐NP (Figure 3e). The latter (green) is covered by both C (red) and nitrogen (blue) as is the surrounding support material (for more EELS analysis see Figure S18) which is indicating the embedding of the NP in an N‐doped carbon matrix. Thus, impregnation of the SiO_2_ support with the Ni‐salen complex and the subsequent pyrolysis and reduction resulted in homogenously distributed Ni‐NPs embedded in a N‐doped carbon matrix throughout the support material. The formation of the N‐doped carbon matrix is caused most probably by decomposition of the Ni‐salen complex. Furthermore, we performed acid leaching of the Ni/SiO_2_ catalyst to gain more insight into the catalytically active species of the catalyst. After the leaching experiment the catalyst showed no catalytic activity (see Supporting Information 7.) indicating that the Ni‐NPs are the catalytically active species. Nitrogen physisorption measurements (Figure S19a) were performed to determine the specific surface areas (Brunauer‐Emmett‐Teller method). A decrease in the surface area from 233 m^2^/g of the pure SiO_2_ support to 188 m^2^/g of the Ni/SiO_2_ catalyst was observed due to impregnation and pyrolysis, while the pore size distribution (Figure S19b) of both materials was almost identical. Inductively coupled plasma optical emission spectrometry (ICP‐OES) analysis revealed 2.8 wt% Ni in the synthesized catalyst material (see Supporting Information 6.).

### Hydrogenation of selected nitro compounds

For the synthesis of 3,4‐dihydro‐2*H*‐pyrrols via hydrogenation of nitro ketones and subsequent cyclization, the catalyst has to be capable of hydrogenating aliphatic nitro compounds while tolerating a ketone as a functional group. In order to determine the selectivity, we studied the hydrogenation of nitroarenes carrying a ketone as a functional group and that of an aliphatic nitro compound (Table 1). To achieve good yields and high selectivity, the catalysis temperature and the Ni content had to be increased except for one substrate. The nitroarenes bearing a ketone functionality in different ring positions (Table 1, entry **1**–**3**) were tolerated very well with yields up to 99 %. In addition, the product from the hydrogenation of 1‐nitropentane, 1‐pentylamine, (Table 1, entry **4**) was isolated as the corresponding hydrochloride salt in an excellent yield of 99 %.

**Table 1:** Chemoselective hydrogenation of nitro compounds.

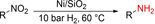		
Entry^[a]^	Product	Yield [%]
1	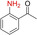	>99^[b]^
2^[c]^	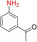	>99^[b]^
3	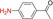	>99^[b]^
4	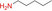	>99^[d]^

Reaction conditions: [a] 0.5 mmol nitro compound, 1.6 mol% Ni (2.8 wt% Ni, 0.008 mmol Ni, 0.44 mg Ni), 60 °C, 10 bar H_2_, 3 mL methylcyclohexane, 20 h; [b] Yields were determined by GC using n‐dodecane as an internal standard and identified by GC coupled with a mass spectrometer (GC‐MS); [c] 0.8 mol% Ni; [d] isolated yield of the corresponding hydrochloride salt.

### Synthesis of 3,4‐dihydro‐2H‐pyrroles

Based on the observed tolerance of ketones as functional groups and the ability to hydrogenate aliphatic substrates, it should be possible to synthesize 3,4‐dihydro‐2*H*‐pyrrols with the Ni/SiO_2_ catalyst via hydrogenation of nitro ketones and subsequent cyclization (Scheme 1b).[^8^, ^14^] The nitro ketones are accessible from three components: a ketone, an aldehyde and a nitroalkane, via aldol condensation^[9]^ and Michael addition.^[10]^ The hydrogenative cyclization of 4‐nitro‐1,3‐diphenylbutan‐1‐one to 3,5‐diphenyl‐3,4‐dihydro‐2*H*‐pyrrole was chosen as the model reaction for the optimization of the reaction conditions. The use of methylcyclohexane (MCH), the optimized solvent for the reduction of nitroarenes, resulted in complete conversion of the educt but gave only a yield of 39 % (Table 2, entry 1) of the product desired (3 mol% Ni, 100 °C, 20 bar H_2_). Therefore, a new solvent screening was performed (Table 2, entries 1–5). Using acetonitrile (MeCN) as a solvent, increased the yield of 3,5‐diphenyl‐3,4‐dihydro‐2*H*‐pyrrole up to 71 % (Table 2, entry 5), while the other solvents led to relatively low yields (Table 2, entries 2–4). After optimization of the catalyst loading and hydrogen pressure (Table S6 and S7), a further enhancement in the yield of the product desired was observed when the reaction temperature was increased to 120 °C (Table 2, entries 6–8). The optimal conditions, 4 mol% Ni, 120 °C and 20 bar H_2_ (Table 2, entry 8), permitted a product yield of 96 %. Furthermore, the hydrogenation and cyclization of 4‐nitro‐1,3‐diphenylbutan‐1‐one was investigated (4 mol% Ni, 20 bar H_2_, 120 °C and 16 h reaction time for 5 runs, see Supporting Information 7. and Figure S20) to demonstrate the recyclability of the Ni/SiO_2_ catalyst. The conversion of the reaction was around 80 % yield under these conditions and no loss of activity was observed after five runs. An upscaling experiment using 5 mmol 4‐nitro‐1,3‐diphenylbutan‐1‐one as an educt resulted in the isolation of 4.5 mmol (90 %) 3,5‐diphenyl‐3,4‐dihydro‐2*H*‐pyrrole.

**Table 2:** Screening the reaction conditions for the synthesis of 3,4‐dihydro‐2*H*‐pyrroles.

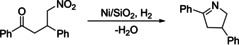		
Entry^[a]^	Solvent	Yield^[b]^ [%]
1	MCH	39
2	Xylene	21
3	Toluene	29
4	MeOH	13
5	MeCN	71
Bold line hereEntry^[c]^	Temperature [°C]	Yield^[b]^ [%]
Bold line her6	100	75
7	110	83
8	120	96

Reaction conditions: [a] 0.2 mmol nitro ketone, 3 mol% Ni (2.8 wt% Ni, 0.006 mmol Ni, 0.36 mg Ni), 3 mL solvent, 100 °C, 20 bar H_2_, 20 h; [b] Yields were determined by GC using n‐dodecane as an internal standard; [c] 0.2 mmol nitro ketone, 4 mol% Ni (2.8 wt% Ni, 0.008 mmol Ni, 0.48 mg Ni), 3 mL MeCN, 20 h.

**Figure 4:**
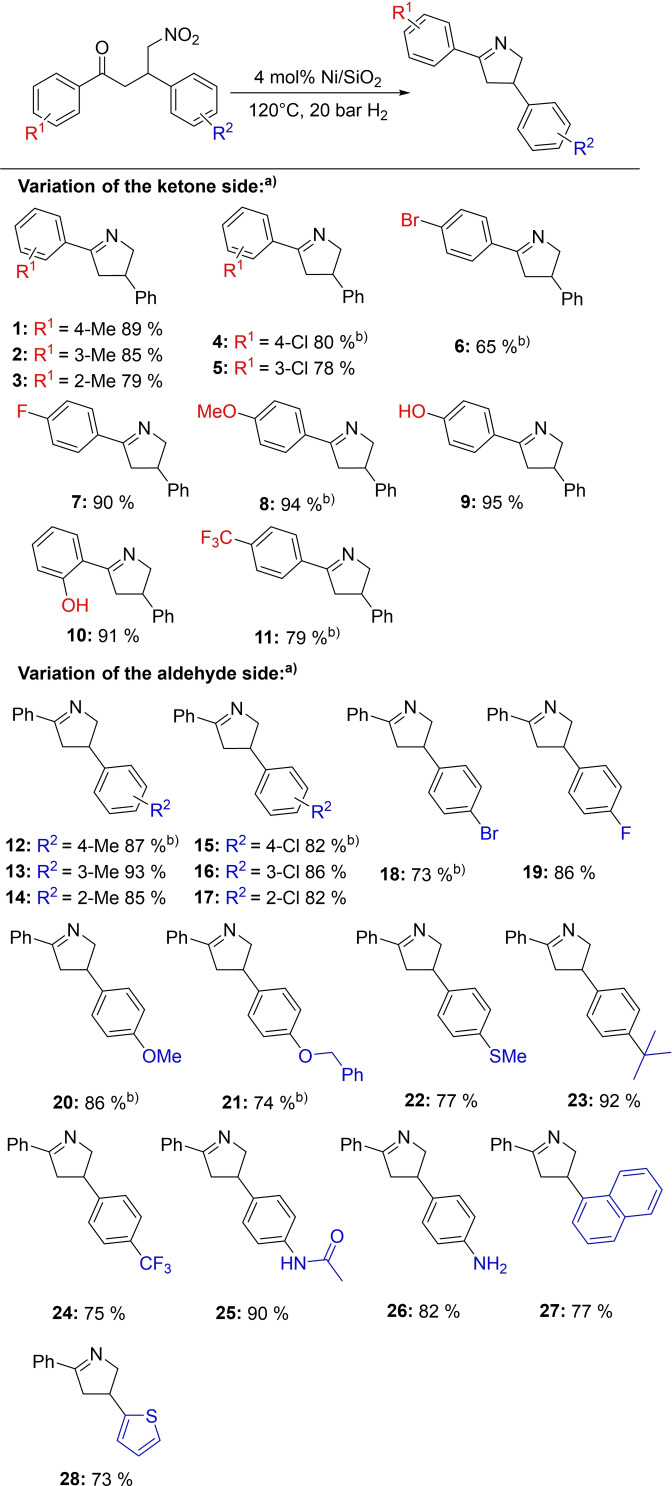
Substrate scope for the synthesis of 3,4‐dihydro‐2*H*‐pyrroles. Reaction conditions: a) 0.2 mmol nitro ketone, 4 mol% Ni (2.8 wt% Ni, 0.008 mmol Ni, 0.48 mg Ni), 120 °C, 20 bar H_2_, 3 mL MeCN, 20 h, molecular sieve; isolated yields are given. b) 5 h.

**Figure 5:**
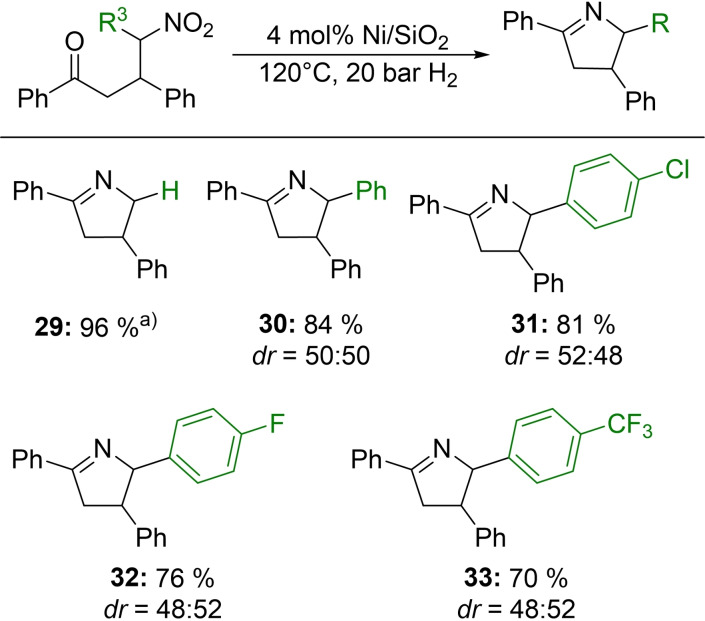
Substrate scope for the synthesis of trisubstituted 3,4‐dihydro‐2*H*‐pyrroles. Reaction conditions: 0.2 mmol nitro ketone, 4 mol% Ni (2.8 wt% Ni, 0.008 mmol Ni, 0.48 mg Ni), 120 °C, 20 bar H_2_, 3 mL MeCN, 20 h molecular sieve; isolated yields are given. a) Yield was determined by GC using n‐dodecane as an internal standard.

Next, we were interested in investigating a substrate scope for the synthesis of various 3,4‐dihydro‐2*H*‐pyrroles. Additionally, we observed a slight increase of the products desired by the addition of molecular sieve to the reaction solution which is likely due to the removal of the resulting water from the cyclization reaction. At first, we varied the ketone side of the nitro ketone starting materials. Substrates with electron‐donating methyl groups in *ortho*‐, *meta*‐ and *para*‐position were obtained in good yields ranging from 79–89 % (Figure 4, **1**–**3**), as were *meta*‐ and *para*‐chlorinated substrates (Figure 4, **4**, **5**) with the *para*‐chlorinated 3,4‐dihydro‐2*H*‐pyrrole observed in an 80 % yield after only 5 h reaction time. Other halogenated nitro ketones, for example, with bromine or the stronger electron‐withdrawing fluorine were hydrogenated to the respective 3,4‐dihydro‐2*H*‐pyrrole derivatives in moderate (65 %, Figure 4, **6**) and good yields (90 %, Figure 4, **7**). The 3,4‐dihydro‐2*H*‐pyrrole bearing a bromo substituent was also received after 5 h. Methoxy and alcohol groups were tolerated during hydrogenation, resulting in excellent yields (91 ‐ 94 %) of the respective products (Figure 4, **8**–**10**). Additionally, a nitro ketone carrying the strongly electron‐withdrawing trifluoromethyl group could be converted in a yield of 79 % (Figure 4, **11**) after only 5 h reaction time. Afterwards, we studied the hydrogenation of nitro ketones in which the aldehyde side was modified. Substrates with either electron‐donating or withdrawing moieties, such as methyl or chlorine, in *ortho*‐, *meta*‐ and *para*‐position were tolerated very well with yields up to 93 % (Figure 4, **12**–**17**). Furthermore, the two *para*‐substituted products required only an abbreviated reaction time of 5 h. Two further halogenated 3,4‐dihydro‐2*H*‐pyrroles (Figure 4, **18**, **19**) could be acquired after 5 h with isolated yields of 73 (R=4‐Br) and 86 % (R=4‐F). Educts bearing stronger electron‐donating ether groups, such as methoxy or benzyloxy, led to yields of 86 (Figure 4, **20**) and 74 % (Figure 4, **21**) of the corresponding 3,4‐dihydro‐2*H*‐pyrroles, respectively, after only 5 h reaction time. Even a nitro ketone with a thioether as a functional group could be easily converted into the product desired without affecting the activity of the catalyst, obtaining a yield of 77 % (Figure 4, **22**). Moreover, a substrate carrying the sterically demanding *tert*‐butyl group was successfully reduced with an isolated yield of 92 % (Figure 4, **23**). The strongly electron‐withdrawing trifluoromethyl group was also introduced into a nitro ketone and converted into the corresponding 3,4‐dihydro‐2*H*‐pyrrole in a good yield of 75 % (Figure 4, **24**). Additionally, an amide group could be tolerated during the reduction, resulting in a very good yield of 90 % (Figure 4, **25**). When a second nitro group was introduced, the catalyst was able to hydrogenate both, yielding an amine‐functionalized 3,4‐dihydro‐2*H*‐pyrrole in 82 % yield (Figure 4, **26**). Nitro ketones, which differ from the substituted benzaldehyde motive used previously, were synthesized from acetophenone, 1‐naphthaldehyde or 2‐thiophenecarbaldehyde and nitromethane. Neither the conjugated aromatic system nor the thiophene heterocycle led to a significant decrease in the catalytic activity of the Ni/SiO_2_ system, resulting in yields of 77 and 73 %, respectively (Figure 4, **27**, **28**). To our delight, trisubstituted 3,4‐dihydro‐2*H*‐pyrroles (Figure 5) can also be synthesized applying our catalytic reaction. Good, isolated yields ranging from 70–84 % (Figure 5, **29**–**33**) were observed. Compounds **30**–**33** were received in a mixture. The diastereomeric ratio (*dr*) of the compounds is shown in Figure 5 and SI 9. and was determined via NMR spectroscopy.

## Conclusion

In conclusion, we synthesized 33 substituted 3,4‐dihydro‐2*H*‐pyrroles in good to excellent yields using our highly active and selective Ni/SiO_2_ catalyst. 23 of the synthesized examples are compounds which have not yet been reported. The nitro ketones used can be assembled easily from three components with each being inexpensive and diversely available. The Ni catalyst was identified from a library with 24 members and consists of homogenously distributed Ni‐NPs which are embedded in a N‐doped carbon matrix throughout the support material.

## Experimental Section

**X‐ray crystallography**: Deposition Number 2152145 contains the supplementary crystallographic data for this paper. These data are provided free of charge by the joint Cambridge Crystallographic Data Centre and Fachinformationszentrum Karlsruhe Access Structures service.

**Catalyst synthesis**: An amount of 300 mg SiO_2_ was added to a suspension of 73 mg of the Ni‐salen complex (0.16 mmol) in 6 mL acetonitrile and stirred vigorously for 5 min. The suspension was heated up to 100 °C and stirred until the solvent was evaporated. Afterwards, the impregnated material was pyrolyzed under nitrogen atmosphere at 700 °C followed by reduction at 550 °C under forming gas (N_2_/H_2_, 90/10).

**General procedure for the hydrogenation of nitrobenzene**: A 10 mL reaction vial was charged with a magnetic stirring bar, 0.5 mmol (51.3 μL) nitrobenzene, 3 mL of the solvent and the desired amount of the chosen catalyst (10 mol% Fe, 2.5 mol % Co, 0.8 mol% Ni). The vial was placed in a 300 mL high‐pressure autoclave (Parr Instruments). The autoclave was flushed with 20 bar hydrogen three times before the final pressure (60 bar for iron catalysts, 50 bar for cobalt catalysts, 50 bar or 20 bar for nickel catalysts, 10 bar for Ni/SiO_2_) was applied. The reaction was stirred at the desired temperature for 20 h. Afterwards the autoclave was cooled to room temperature and the hydrogen was released. For quantitative GC analysis n‐dodecane was added as an internal standard.

**General procedure for the hydrogenation of nitro compounds**: A 10 mL reaction vial was charged with a magnetic stirring bar, 0.5 mmol substrate, 3 mL methylcyclohexane and 8 mg (2.8 wt% Ni, 0.004 mmol Ni, 0.8 mol% Ni) or 16 mg (2.8 wt% Ni, 0.008 mmol Ni, 1.6 mol% Ni) of the Ni/SiO_2_ catalyst. The vial was placed in a 300 mL high‐pressure autoclave (Parr Instruments). The autoclave was flushed with 20 bar hydrogen three times before the final pressure (10 bar) was applied. The reaction was stirred at 60 °C for 20 h. The autoclave was subsequently cooled to room temperature and the hydrogen was released. n‐dodecane was added as an internal standard for quantitative GC analysis. Qualitative analysis was accomplished by GC‐MS. The catalyst was removed by filtration, washed with ethyl acetate and the solvent was removed under reduced pressure to isolate the aliphatic amine. To convert the amine into the corresponding hydrochloride salt, the residue was dissolved in ether and 0.5 mL hydrochloric acid in ether was added to precipitate the salt. The precipitate was isolated, dried under reduced pressure and analyzed with ^1^H and ^13^C NMR spectroscopy.

**General procedure for the hydrogenation of nitro ketones**: A 10 mL reaction vial was charged with a magnetic stirring bar, 0.2 mmol substrate, molecular sieve 3 Å (30 mg), 3 mL solvent and 17 mg (2.8 wt% Ni, 0.008 mmol Ni, 4 mol% Ni) of the Ni/SiO_2_ catalyst. The vial was placed in a 300 mL high‐pressure autoclave (Parr Instruments). The autoclave was flushed with 20 bar hydrogen three times before the final pressure (20 bar) was applied. The reaction was stirred at 120 °C for 5 or 20 h. Afterwards, the autoclave was cooled to room temperature and the hydrogen was released. n‐dodecane was added as an internal standard and the reaction mixture was quantitatively analyzed with GC chromatography for screening reactions. Regarding isolation of the products (1–33), the catalyst was removed by filtration and washed with ethyl acetate. The solvent was removed under reduced pressure and the product was either precipitated in pentane at −24 °C (1–28) or isolated by column chromatography (pentane:ethyl acetate=9.5 : 1) (30–33). The products received were analyzed with ^1^H and ^13^C NMR spectroscopy. High‐resolution mass spectroscopy (HRMS) was carried out for products with incomplete spectroscopic literature data.

## Conflict of interest

The authors declare no conflict of interest.

1

## Supporting information

As a service to our authors and readers, this journal provides supporting information supplied by the authors. Such materials are peer reviewed and may be re‐organized for online delivery, but are not copy‐edited or typeset. Technical support issues arising from supporting information (other than missing files) should be addressed to the authors.

Supporting Information

## Data Availability

The data that support the findings of this study are available on request from the corresponding author. The data are not publicly available due to privacy or ethical restrictions.
