# Supplementary material for: Synthesis of 3,4‐Dihydro‐2H‐Pyrroles from Ketones, Aldehydes, and Nitro Alkanes via Hydrogenative Cyclization
Source: Chemistry. 2022 Jul 6;28(47):e202201307. doi: 10.1002/chem.202201307 (PMC9545131; doi:10.1002/chem.202201307)
Supplement: Supplementary file 1 — Supporting Information [file CHEM-28-0-s001.pdf]

Chemistry–A European Journal

Supporting Information

Synthesis of 3,4-Dihydro-2*H*-Pyrroles from Ketones, Aldehydes, and Nitro Alkanes via Hydrogenative Cyclization

Barbara Klausfelder, Patricia Blach, Niels de Jonge, and Rhett Kempe*

Table of Contents

1. General Considerations 2
2. Material synthesis 5
3. Characterization of the Salen complexes 11
4. Characterization of the catalyst library 14
5. Catalytic studies – Hydrogenation of nitrobenzene 23
6. Characterization of the Ni/SiO_2 catalyst 26
7. Catalytic studies – Hydrogenation of nitro ketones 30
8. Characterization of the nitro ketones.. 33
9. Characterization of the isolated products 51
10. NMR Spectra 70
11. HRMS Spectra.. 138
12. Supplementary References 162

1. General Considerations

All chemicals and solvents were obtained from commercial suppliers with purity over 95 % and used without further purification. SiO_2, TiO_2 (Anatase) and γ-Al_2O_3 were purchased from Alfa Aesar and used without further purification. Activated carbon (Norit CA1) was received from Cabot Corporation and heated up to 700 °C under a nitrogen atmosphere for 3 h (10 K/min) before use. All air- and moisture-sensitive reactions were carried out under standard Schlenk or glovebox techniques (nitrogen-filled).

NMR spectra were collected on Varian INOVA 300 (300 MHz for ^{1}H, 75 MHz for ^{13}C), Varian INOVA 400 (400 MHz for ^{1}H, 100 MHz for ^{13}C) or Bruker Avance III HD 500 (500 MHz for ^{1}H, 125.7 MHz for ^{13}C) instruments at 298 K. Chemical shifts are reported in ppm relative to the residual solvent signal ($CDCl_3$: 7.26 ppm (^{1}H), 77.16 ppm (^{13}C); DMSO-D6: 2.50 ppm (^{1}H), 39.51 ppm (13C), CD_2Cl_2: 5.32 ppm (^{1}H), 53.84 ppm (^{13}C), acetone-D6: 2.05 ppm (^{1}H), 29.84, 206.26 ppm (^{13}C)). Coupling constants (J) are reported in Hz (coupling patterns: s = singlet, d = doublet, dd = doublet of doublets, dt = doublet of triplets, ddd = doublet of doublets of doublets, ddt = doublet of doublets of triplets, t = triplet, td = triplet of doublets, tt = triplet of triplets, p = quintet, and m = multiplet).

GC analyses were performed using an Agilent 6890N GC system equipped with a HP 5 column (30 m x 0.32 mm x 0.25 µm). For quantitative GC analysis n-dodecane was used as an internal standard. The standard was calibrated for each substrate used.
GC-MS analyses were carried out using an Agilent 7890A GC system equipped with a HP-5MS column (30 m x 0.32 mm x 0.25 µm) and a 5975C inert MSD detector (EI, 70 eV).

High-resolution mass spectra (HRMS) were obtained from a Thermo Scientific Q Exactive (Orbitrap) instrument in ESI+ mode.

IR spectra were collected on a JASCO FT/IR-6100 apparatus in a measuring range from 4000 – 400 cm^{-1}.

Elemental analysis for determination of the nitrogen, hydrogen and carbon content was carried out using an elementar vario EL III.
Transmission electron microscopy (TEM) was carried out by using either a LEO 922o (200 kV) or a JEOL JEM 2200FS (200 kV) instrument. The samples were suspended in $CHCl_3$ and sonicated for 5 min. Afterwards, 2 µL of the suspension were dropped onto a CF200-Cu grid or a LC200-Cu grid and allowed to dry.

High-angle annular dark-field scanning transmission electron microscopy (HAADF-STEM) measurements were performed using a JEM-ARM200F (JEOL,200 kV) equipped with an energy-dispersed X-ray analysis (EDX) system (JEOL). An annular dark-field detector (ADF) with a collection angle (inner and outer) of 68 – 280 mrad was used in STEM mode (image size: 1024 x 1024 pixels; pixel size: 0.048 – 0.095 nm; dwell time: 6 µs). The probe current was set to 1000 pA (image size: 128 x 128 pixels; pixel size: 0.4 – 1.0 nm; dwell time: 1 or 0.5 ms) and 10 sweep counts were integrated for EDX measurements. The image collection angle was set to 20.8 mrad and the electron probe convergence semi-angle to 30 – 35 mrad (image size: 100 x 100 pixels; pixel size: 0.21 – 0.34 nm; dwell time: 0.002 s) for the electron energy loss spectroscopy analysis (EELS) with an energy dispersion of 0.1 eV.

SEM-EDX was performed using either a Zeiss Field Emission Scanning Electron Microscope (FESEM) LEO 1530 GEMINI or a Zeiss Ultra Plus with an acceleration voltage of 20 kV. The samples were previously sputtered with platinum (1.3 µm) using a Sputter Coater 208HR (Cressington) to provide a conductive surface.

X-ray photoelectron spectroscopy (XPS) was carried out using a PHI Versa Probe III apparatus from Physical Electronics. A monochromatic AlKα with a spot size of 100 µm (24.5 W) was used as the X-ray source. The determination of the kinetic pass energy of the photoelectrons was carried out using a hemispheric analyzer (45 °). The sample was sputtered for 5 min with 1 kV for the detailed spectra. The spectra were calibrated to C1s.

Powder X-ray diffraction (XRD) was carried out using a STOE STADI P Mythen2 4 K diffractometer (Ge(111) monochromator; Ag Kα1 radiation, λ = 0.5594 Å) with four Dectris MYTHEN2 R 1 K detectors in Debye-Scherrer geometry. The sample was

measured in 0.5 mm diameter glass capillaries from Hilgenberg (special purpose glass number 10).
Inductively coupled plasma optical emission spectrometry (ICP-OES) was measured to determine the nickel content using a Varian Vista Pro.

N_2 physisorption measurements were performed at -196 °C utilizing a Quantachrome Nova 2000e apparatus. The p/p0-values from 0.05 to 0.3 were used by the BET model to calculate the specific surface areas. The average pore volume and width were determined by DFT calculations [N_2 at -196.15 °C on carbon; (SiO_2: slit and cylindrical pores, NLDFT equilibrium model; TiO_2: cylindrical pores, NLDFT equilibrium model; γ Al_2O_3: cylindrical pores, NLDFT equilibrium model; activated carbon: cylindrical pores, NLDFT equilibrium model)].

Pyrolysis and reduction under nitrogen atmosphere and forming gas (90 % N_2/10 % H_2) were carried out in a high temperature furnace (Carbolite, EHA 12/450B200) or ChemBET Pulsar TPR/TPD.

Magnetic measurements of the materials were taken on a SQUID MPMS-XL5 from Quantum Design with the field range of -3 – 3 T in hysteresis mode. The samples were prepared in a capsule held in a plastic straw under a protective atmosphere. The raw data were corrected for the diamagnetic part of the sample holder.

X-ray crystal structure analysis was performed with a STOE STADIVARI (MoKα radiation, 0.71073 Å) equipped with an Oxford Cryostream low temperature unit. The structure solution and refinement were achieved with OLEXS.1 The structure was visualized using Mercury 3.8.2

Macherey Nagel silica gel 60 (40 – 6 µm particle size) was used for column chromatography.

2. Material synthesis

Ligand synthesis – Salen(cy)(methoxy)

6.17 g (40 mmol, 2 eq.) 2-hydroxy-3-methoxybenzaldehyde were dissolved in 100 mL ethanol and 2.51 g (22 mmol, 1.1 eq.) (±)-trans-1,2-diaminocyclohexane were added. The solution was heated under reflux for 1 h. After cooling to room temperature, the product crystallized as yellow crystalline powder, which was separated by filtration and washed with diethyl ether (6.91 g, 90 %).

^{1}H NMR (400 MHz, $CDCl_3$): δ = 13.85 (s, 1H), 8.24 (s, 2H), 6.85 (dd, *J* = 7.8, 1.6 Hz, 2H), 6.78 (dd, *J* = 7.8, 1.6 Hz, 2H), 6.72 (t, *J* = 7.8 Hz, 2H), 3.86 (s, 6H), 3.36 – 3.25 (m, 2H), 1.90 (dd, *J* = 26.4, 10.6 Hz, 4H), 1.78 – 1.63 (m, 2H), 1.64 – 1.40 (m, 2H) ppm.

^{13}C NMR (100 MHz, $CDCl_3$): δ = 164.85, 151.66, 148.34, 123.26, 118.49, 117.99, 113.91, 72.54, 56.13, 33.14, 24.17 ppm.

Complex synthesis

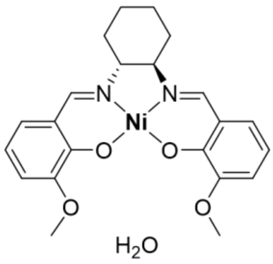

Ni-sal

The monometallic Ni-Salen(cy)(methoxy) complex (Ni-sal) was synthesized according to a known literature procedure.[1]

1.14 g (3 mmol) of the ligand were dissolved in 50 mL ethanol at 50 °C. Then 0.74 g (3 mmol) $Ni(OAc)_2 \cdot 4\ H_2O$ were added and the solution was stirred at 50 °C for 2 h whereby an orange-brown solid precipitates. The precipitate was filtered over a frit and washed with cold ethanol (1.22 g. 89 %).

Elemental analysis: calcd for $C_{22}H_{26}N_2NiO_5$: C 57.80, H 5.74, N 6.13; found: C 57.87, H 5.81, N 6.32

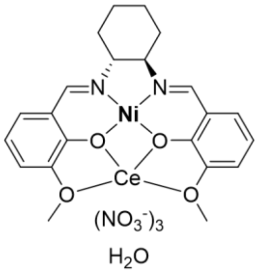

NiCe-sal

The bimetallic NiCe-Salen(cy)(methoxy) complex (NiCe-sal) was synthesized according to a known literature procedure.[1]

1.14 g (3 mmol) of the ligand were dissolved in 50 mL ethanol at 50 °C. Then 0.74 g (3 mmol) $Ni(OAc)_2 \cdot 4\ H_2O$ were added and the solution was stirred at 50 °C for 2 h whereby an orange-brown solid precipitates. Afterwards the solvent was removed under reduced pressure and the residue was dissolved in 90 mL acetonitrile followed by adding of 1.30 g (3 mmol) $Ce(NO_3)_3 \cdot 6\ H_2O$. An orange precipitate was formed during stirring for 12 h at room temperature and heating up to 60 °C for 30 minutes. The precipitate was filtered over a frit and washed with cold ethanol (2.25 g. 96 %).

Elemental analysis: calcd for $C_{22}H_{26}CeN_5NiO_{14}$: C 33.73, H 3.35, N 8.94; found: C 33.79, H 3.22, N 8.95

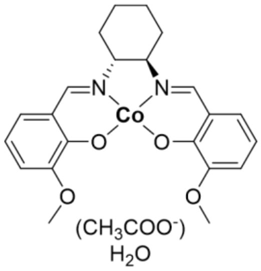

Co-sal

The monometallic Co-Salen(cy)(methoxy) complex (Co-sal) was synthesized according to a modified literature procedure.[2]

1.14 g (3 mmol) of the ligand were dissolved in 50 mL ethanol at 55 °C. Then 0.75 g (3 mmol) $Co(OAc)_2 \cdot 4\ H_2O$ were added and the solution was stirred at 55 °C for 2 h whereby a light brown solid precipitates. The precipitate was filtered over a frit and washed with cold ethanol (1.35 g. 90 %).

Elemental analysis: calcd for $C_{24}H_{29}CoN_2O_7$: C 55.82, H 5.66, N 5.42; found: C 55.58, H 6.15, N 5.29

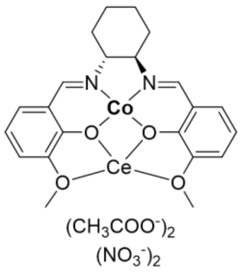

CoCe-sal

The bimetallic CoCe-Salen(cy)(methoxy) complex (CoCe-sal) was synthesized according to a modified literature procedure.[2]

1.14 g (3 mmol) of the ligand were dissolved in 55 mL ethanol at 50 °C. Then 0.75 g (3 mmol) $Co(OAc)_2 \cdot 4\ H_2O$ were added and the solution was stirred at 55 °C for 2 h whereby a light brown solid precipitates. Afterwards the solvent was removed under reduced pressure and the

residue was dissolved in 90 mL acetonitrile followed by adding of 1.30 g (3 mmol) $Ce(NO_3)_3 \cdot 6\ H_2O$. A dark green precipitate was formed during stirring for 16 h at room temperature and heating up to 90 °C for 1 h. The precipitate was filtered over a frit and washed with ethanol (1.52 g. 88 %).

Elemental analysis: calcd for $C_{26}H_{30}CeCoN_4O_{14}$: C 38.01, H 3.68, N 6.82; found: C 36.18, H 3.61, N 7.66

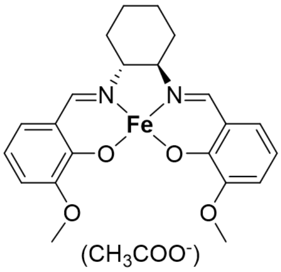

Fe-sal

The monometallic Fe-Salen(cy)(methoxy) complex (Fe-sal) was synthesized according to a known literature procedure.[3]

1.14 g (3 mmol) of the ligand were dissolved in 40 mL acetone. Then 0.52 g (3 mmol) $Fe(OAc)_2$ were added and the solution was stirred under aerobic conditions at room temperature for 24 h whereby a purple solid precipitates. The precipitate was filtered over a frit and washed with acetone and ethanol (1.05 g. 71 %).

Elemental analysis: calcd for $C_{24}H_{27}FeN_2O_6$: C 55.20, H 5.49, N 5.66; found: C 55.23, H 5.69, N 5.48

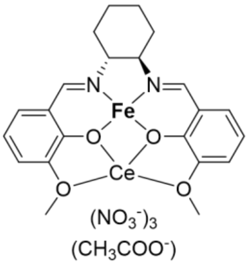

FeCe-sal

The bimetallic FeCe-Salen(cy)(methoxy) complex (FeCe-sal) was synthesized according to a modified literature procedure.[2]

1.14 g (3 mmol) of the ligand were dissolved in 40 mL acetone. Then 0.52 g (3 mmol) $Fe(OAc)_2$ were added and the solution was stirred under aerobic conditions at room temperature for 2 h whereby a purple solid precipitates. Afterwards the solvent was removed under reduced pressure and the residue was dissolved in 90 mL acetonitrile followed by adding of 1.30 g (3 mmol) $Ce(NO_3)_3 \cdot 6\ H_2O$. A light brown precipitate was formed during stirring for 16 h at room temperature and heating up to 90 °C for 1 h. The precipitate was filtered over a frit and washed with acetone (1.94 g. 79 %).

Elemental analysis: calcd for $C_{24}H_{27}CeFeN_5O_{15}$: C 35.09, H 3.31, N 8.53; found: C 35.48, H 3.45, N 8.48

Catalyst synthesis

To a suspension of 0.16 mmol (Ni, Co) or 0.17 mmol (Fe) of the respective complex (73 mg for Ni-Sal, 83 mg for Co-Sal; 84 mg for Fe-Sal; 125 mg for NiCe-Sal, 131 mg for CoCe-Sal and 140 mg for FeCe-Sal) in 6 mL MeCN, 300 mg of the desired support material (SiO_2, TiO_2, γ-Al_2O_3, activated carbon) were added. The suspension was heated up to 100 °C and stirred until the solvent was evaporated. Afterwards the materials were pyrolyzed under nitrogen atmosphere at 700 °C followed by reduction at 550 °C under forming gas (N_2/H_2, 90/10).

The catalysts used for the screening reactions were synthesized using equivalent amounts of the metal salts.

Acid leaching of Ni/SiO_2

50 mg of the Ni/SiO_2 catalyst were suspended in 50 mL of 2 M HCl. The solution was stirred for 20 h at 50 °C. After cooling to room temperature, the catalyst was washed until the supernatant solution was neutral and then dried under vacuum.

Synthesis of the chalcones

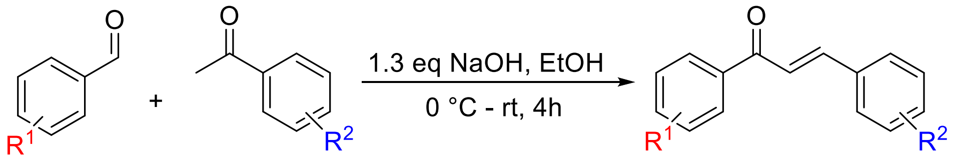

The substituted chalcones were obtained from commercially suppliers or synthesised according to known or modified literature procedures.[4]

5 mmol of the aromatic ketone were dissolved in 4 mL EtOH and 2.32 mL of an aqueous solution of NaOH (2.8 M) was added. The solution was cooled to 0 °C and then 1 eq. of the aromatic aldehyde was added dropwise. The mixture was allowed to warm up to room temperature and stirred for 4-16 h. In case of liquid products, solvent was removed under reduced pressure and the residue was dissolved in ethyl acetate and washed twice with water. The combined organic layers were dried over Na_2SO_4 and the solvent was removed under reduced pressure. In case of solid products, the product was collected by filtration and washed with cold water.

All spectroscopic data of the substituted chalcones are in good agreement with literature data.

Synthesis of the nitro ketones

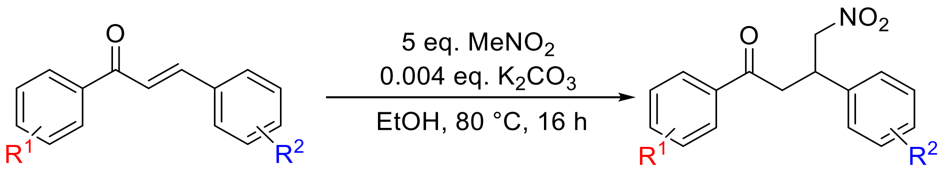

The nitro ketones were synthesised according to a modified literature procedure.[5]

1.5 mmol or 3 mmol of the substituted chalcone, 5 eq. nitromethane and 0.004 eq. K_2CO_3 were dissolved in 4 mL EtOH and heated up to reflux (80 °C) for 16 h. After cooling to room temperature, the solvent was removed under reduced pressure and the residue was dissolved in ethyl acetate. The solution was washed twice with water and brine. The combined organic layers were dried over Na_2SO_4 and the solvent was removed under reduced pressure. In case of solid products, the residue was dissolved in ether and the solid was precipitated at -24 °C. Liquid products were used without further purification.

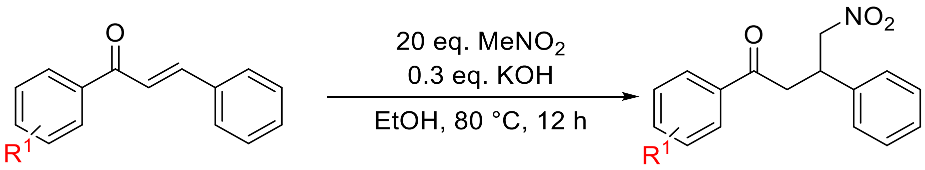

Nitro ketones carrying an alcohol as a functional group were synthesised according to a literature procedure.[6]

1.5 mmol of the substituted chalcone, 20 eq. nitromethane and 0.3 eq. KOH were dissolved in 3 mL EtOH and heated under reflux (80 °C) for 12 h. After cooling to room temperature, the solvent was removed under reduced pressure and the residue was acidified with 0.1 M HCl and taken up in ethyl acetate. The solution was washed two times with water and brine. The combined organic layers were dried over Na_2SO_4 and the solvent was removed under reduced pressure. The oily residue was dissolved in ether and the solid was precipitated at -24 °C. The product was collected by filtration and washed with cold diethyl ether.

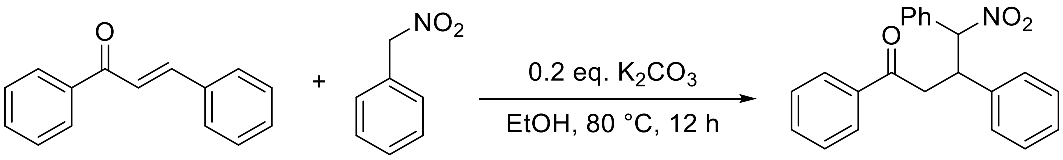

For the nitro ketone synthesised with (nitromethyl)benzene as aliphatic nitro compound 1.5 mmol *trans*-chalcone (312 mg), 1 eq. (nitromethyl)benzene (206 mg, 171 µL) and 0.2 eq. K_2CO_3 were dissolved in 13 mL EtOH and heated under reflux (80 °C) for 16 h.

After cooling to room temperature, the solvent was removed under reduced pressure and the residue was dissolved in $CHCl_3$. The solution was washed twice with water and brine. The combined organic layers were dried over Na_2SO_4 and the solvent was removed under reduced pressure while a white solid precipitated. The solid was washed with cold ether. The product was collected by filtration and washed with cold diethyl ether.

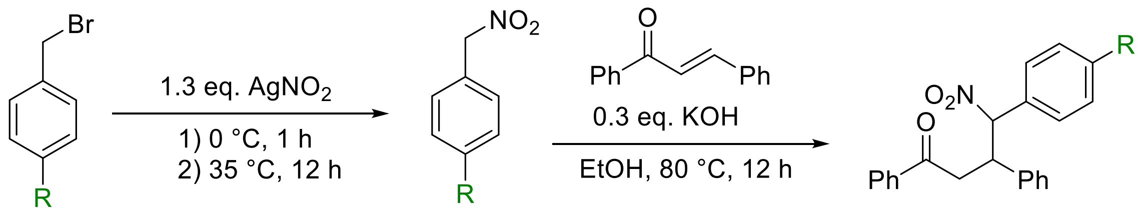

Other substituted (nitromethyl)benzenes for the synthesis of further nitro ketones were synthesized according to a known literature procedure.[7] For this purpose 9 mL anhydrous diethyl ether was added to a round bottom flask covered in tin foil containing 1.3 eq. $AgNO_2$ (3.9 mmol). The mixture was stirred at room temperature for 15 minutes and then cooled to 0 °C. Then a solution of the substituted benzyl bromide (3 mmol) in 1.5 ml anhydrous diethyl ether was added dropwise. After stirring for 1 h at 0 °C the solution was heated under reflux at 35 °C for 12 h. After cooling to room temperature, the solution was filtered over celite using ethyl acetate al solvent. The solvent was removed under reduced pressure and the crude products were received as yellow oils which were used in the next step without further purification.

For the synthesis of the nitro ketones the substituted (nitromethyl)benzene was dissolved in 6 mL EtOH and 1.5 mmol *trans*-chalcone (312 mg) and 0.3 eq. KOH were added. The solution was heated under reflux (80 °C) for 16 h. After cooling to room temperature, the solvent was removed under reduced pressure and the residue was acidified with 0.1 M HCl and taken up in ethyl acetate. The solution was washed two times with water and brine. The combined organic layers were dried over Na_2SO_4 and the solvent was removed under reduced pressure. The oily residue was dissolved in ether and the solid was precipitated at -24 °C. The product was collected by filtration and washed with cold diethyl ether.

3. Characterization of the Salen complexes

Crystallographic data of CoCe-Salen(cy)(methoxy) (CoCe-sal)

Crystals suitable for X-ray analysis were grown from a saturates solution of the compound in acetonitrile at -24 °C.

Deposition Number: 2152145

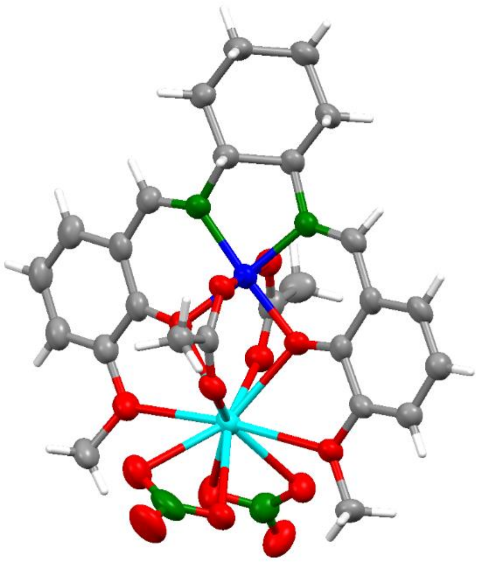

Table S 1: Crystallographic detail of CoCe-Salen(cy)(methoxy).

Compound	CoCe-Salen(cy)(methoxy)
Formula	$C_{26}H_{30}CeCoN_4O_{14}$+solvent
Formula weight	821.59
Crystal system	triclinic
Space group	P-1
a / Å	9.1100(18)
b / Å	12.370(3)
c / Å	14.260(3)
α / °	87.10(3)
β / °	82.40(3)
γ / °	79.00(3)
Cell volume / $Å^3$	1563.1(6)
Z	2
Crystal size / mm^3	0.038*0.011*0.017
Habit	plate
Color	greenish brown
Density / gcm^{-1}	1.746
T / K	170
Theta range	2.295-27.04
Unique reflections	7510
Observed reflections [I>2(I)]	4530
Parameters	419
wR2 all data	0.1402
R [I>2(I)]	0.0514

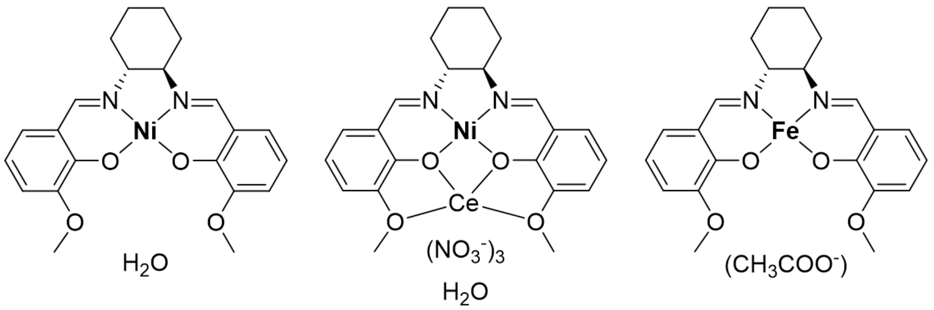

Figure S 1: Structures of the salen complexes known in the literature. Ni-sal[1] and NiCe-sal[1]; Fe-sal[3].

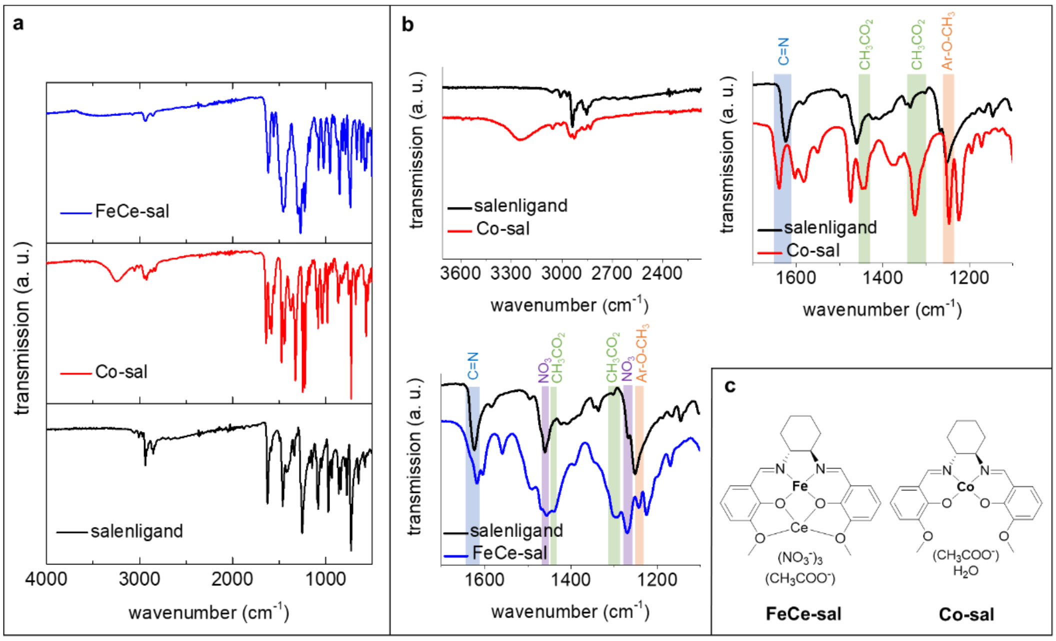

Figure S 2: Characterization of the salen complexes unknown in the literature. a) FT-IR spectra of the salenligand, the Co-salen complex and the FeCe-salen complex (Co-sal, FeCe-sal), b) Sections from the FT-IR spectra of the complexes. For the Co-salen complex the characteristic band of an OH-function could be obtained between 3150 cm^{-1} and 3350 cm^{-1}, as well as the characteristic C=N stretch (blue) at 1650 cm^{-1}, the asymmetric and symmetric stretch of acetate (green) at 1440 cm^{-1} and 1320 cm^{-1} and the characteristic stretch of an aryl ether (orange) at 1250 cm^{-1}. For the FeCe-salen complex the characteristic C=N stretch (blue) at 1650 cm^{-1} could be obtained, as well as the characteristic stretch of nitrate (purple) at 1460 cm^{-1} and 1290 cm^{-1}, the asymmetric and symmetric stretch of acetate (green) at 1440 cm^{-1} and 1320 cm^{-1} and the characteristic stretch of an aryl ether (orange) at 1250 cm^{-1}. c) Proposed structures of the complexes.

4. Characterization of the catalyst library

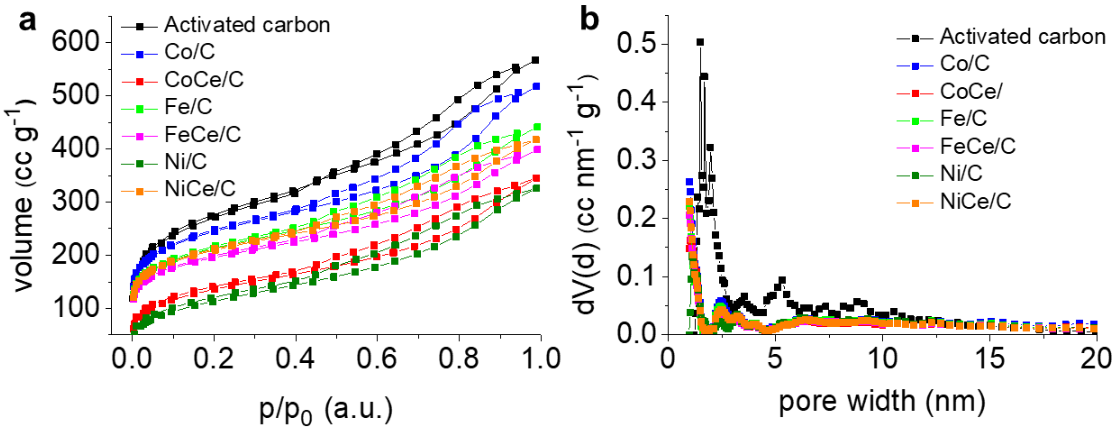

Figure S 3: Characterization of the surface area of the carbon based catalysts by physisorption measurements. a) Isotherms of the activated carbon support and the activated carbon based catalysts. All materials show the typical hysteresis of mesoporous materials. After the impregnation of the carbon support with the different complexes, a decrease of the surface area was observed in all cases. b) Corresponding calculated pore size distributions of the support and the catalysts [N_2 at -196.15 °C on carbon (cylindrical pores, NLDFT equilibrium model)]. No significant differences were noticed.

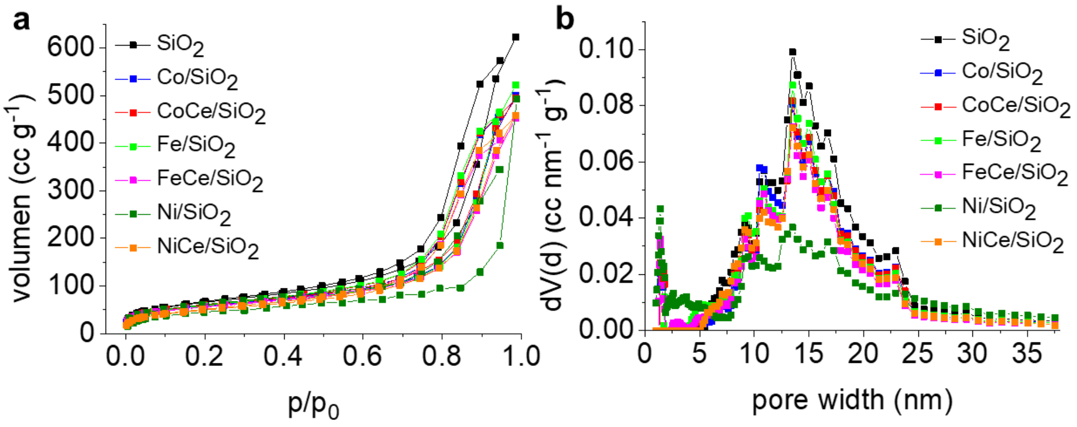

Figure S 4: Characterization of the surface area of the SiO_2 based catalysts by physisorption measurements. a) Isotherms of the SiO_2 support and the SiO_2 based catalysts. All materials show the typical hysteresis of mesoporous materials. After the impregnation of the SiO_2 support with the different complexes, a decrease of the surface area was observed in all cases. b) Corresponding calculated pore size distributions of the support and the catalysts [N_2 at -196.15 °C on carbon (slit and cylindrical pores, NLDFT equilibrium model)]. No significant differences were noticed.

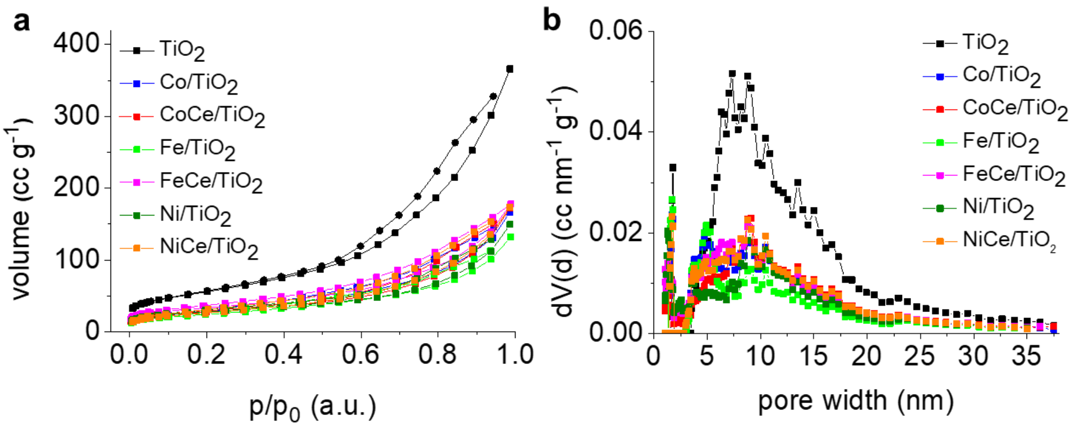

Figure S 5: Characterization of the surface area of the TiO_2 based catalysts by physisorption measurements. a) Isotherms of the TiO_2 support and the TiO_2 based catalysts. All materials show the typical hysteresis of mesoporous materials. After the impregnation of the TiO_2 support with the different complexes, a decrease of the surface area was observed in all cases. b) Corresponding calculated pore size distributions of the support and the catalysts [N_2 at -196.15 °C on carbon (cylindrical pores, NLDFT equilibrium model)]. No significant differences were noticed.

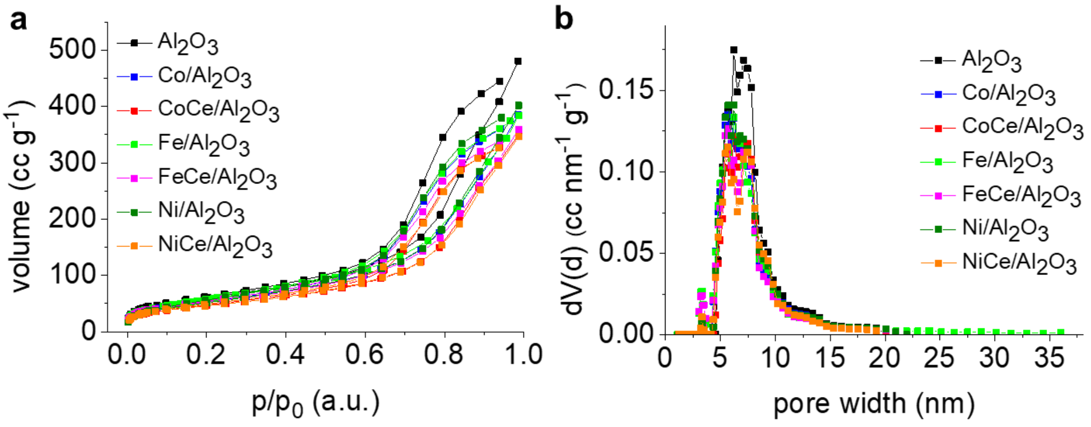

Figure S 6: Characterization of the surface area of the Al_2O_3 based catalysts by physisorption measurements. a) Isotherms of the Al_2O_3 support and the Al_2O_3 based catalysts. All materials show the typical hysteresis of mesoporous materials. After the impregnation of the Al_2O_3 support with the different complexes, a decrease of the surface area was observed in all cases. b) Corresponding calculated pore size distributions of the support and the catalysts [N_2 at -196.15 °C on carbon (cylindrical pores, NLDFT equilibrium model)]. No significant differences were noticed.

Table S 2: Calculated surface areas of the commercially available supports and the synthesised catalysts from the library.

Support/Catalyst	Surface (m^2/g)	Support/Catalyst	Surface (m^2/g)
Activated carbon	911	TiO_2	150
Ni/C	408	Ni/TiO_2	98
NiCe/C	687	NiCe/TiO_2	96
Co/C	805	Co/TiO_2	104
CoCe/C	470	CoCe/TiO_2	97
Fe/C	706	Fe/TiO_2	88
FeCe/C	645	FeCe/SiO_2	118
SiO_2	233	γ-Al_2O_3	220
Ni/SiO_2	188	Ni/Al_2O_3	195
NiCe/SiO_2	178	NiCe/Al_2O_3	168
Co/SiO_2	206	Co/Al_2O_3	196
CoCe/SiO_2	209	CoCe/Al_2O_3	169
Fe/SiO_2	199	Fe/Al_2O_3	207
FeCe/SiO_2	195	FeCe/Al_2O_3	191

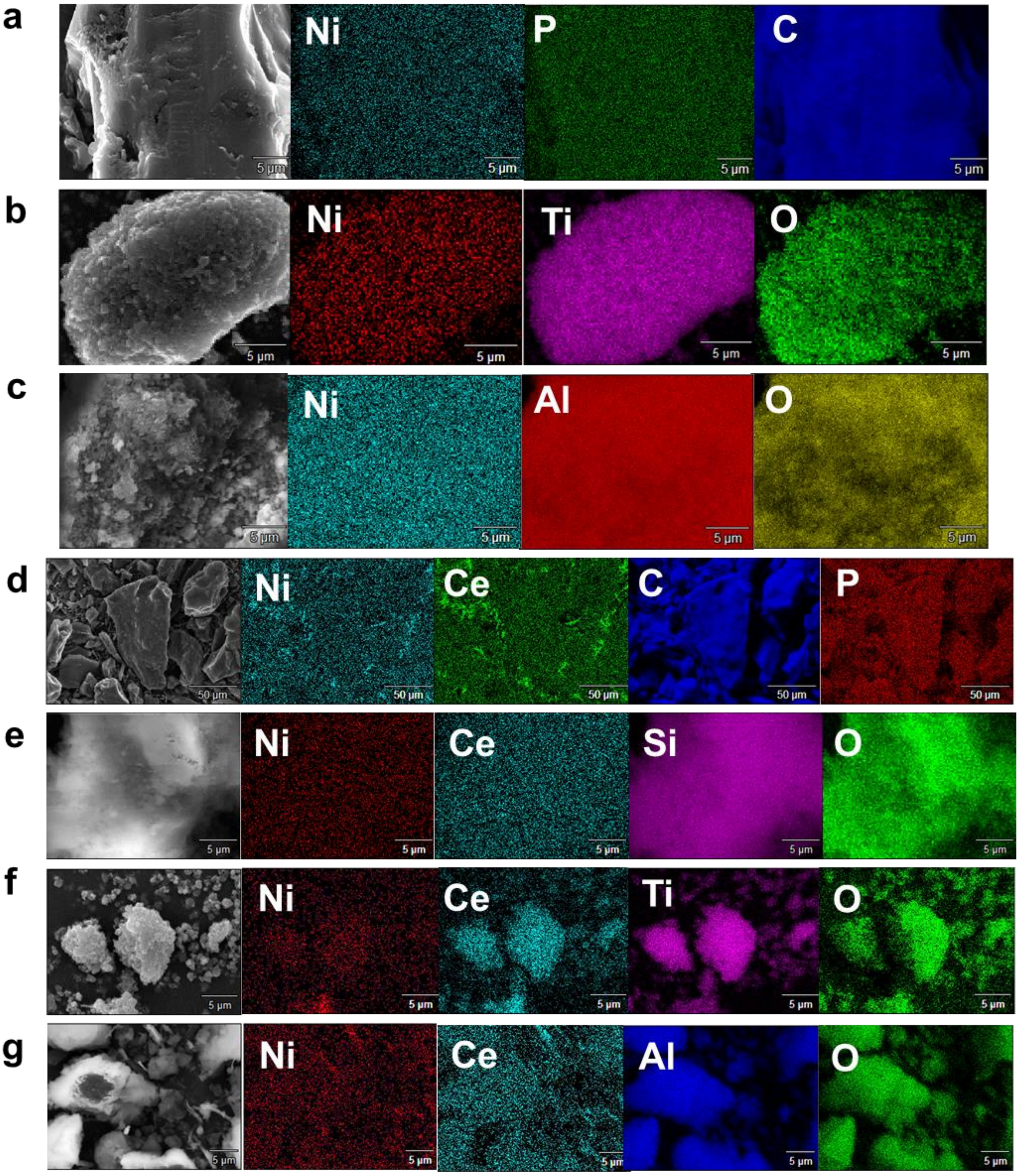

Figure S 7: Scanning electron microscopy (SEM) in combination with energy dispersive X-ray (EDX) mapping of the Ni catalysts. a) Ni/C; b) Ni/TiO_2; c) Ni/Al_2O_3; d) NiCe/C; e) NiCe/SiO_2; f) NiCe/TiO_2; g) NiCe/Al_2O_3. The measurement revealed a homogeneous distribution of nickel and if available cerium on the surface of the support materials. The phosphorus found in the measurement of the carbon supported catalysts is due to a previous activation of the support material.

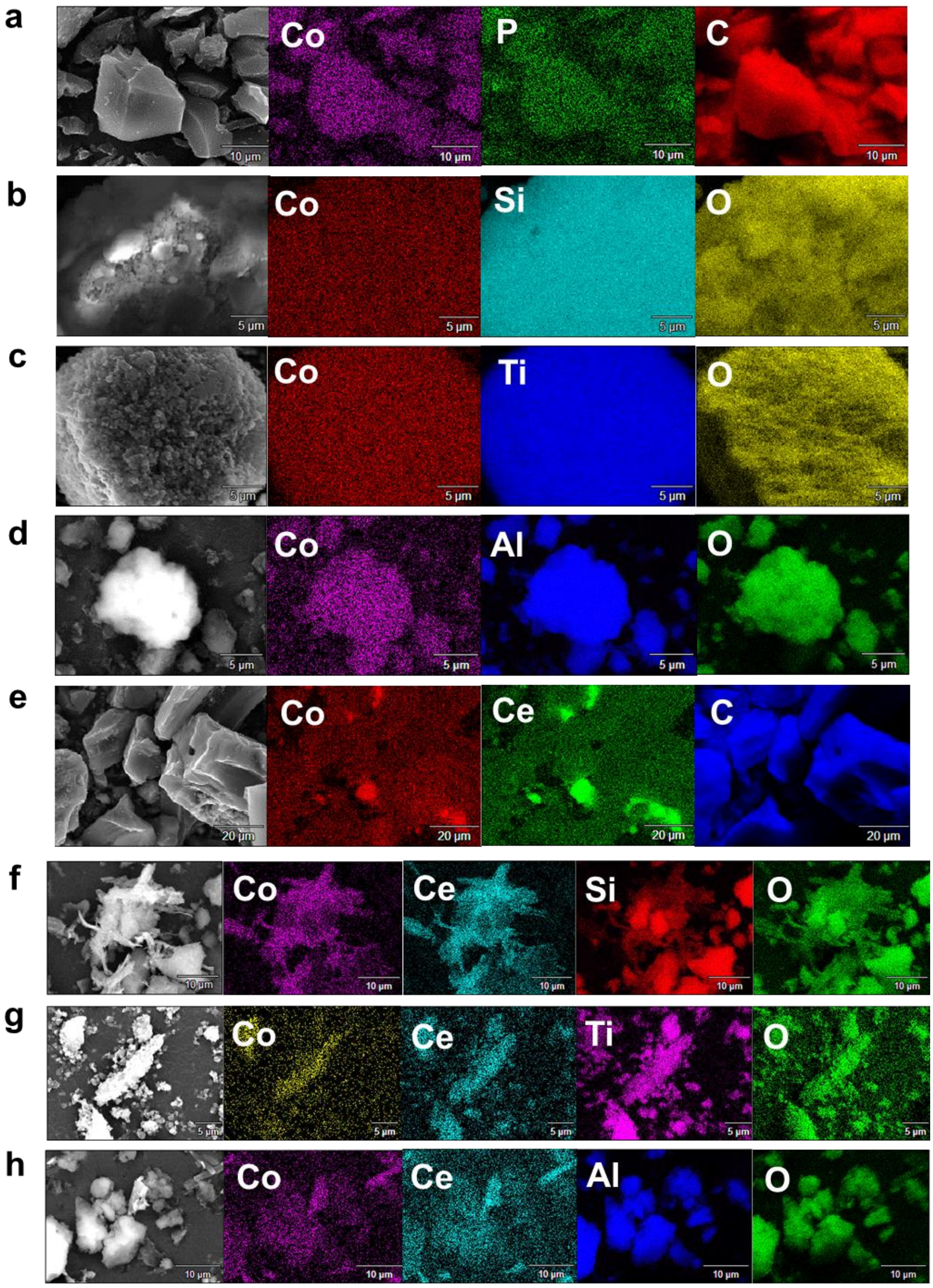

Figure S 8: SEM in combination with EDX mapping of the Co catalysts. a) Co/C; b) Co/SiO_2; c) Co/TiO_2; d) Co/Al_2O_3; e) CoCe/C; f) CoCe/SiO_2; g) CoCe/TiO_2; h) CoCe/Al_2O_3. The measurement revealed a homogeneous distribution of cobalt and if available cerium on the surface of the support materials. The phosphorus found in the measurement of the carbon supported catalysts is due to a previous activation of the support material.

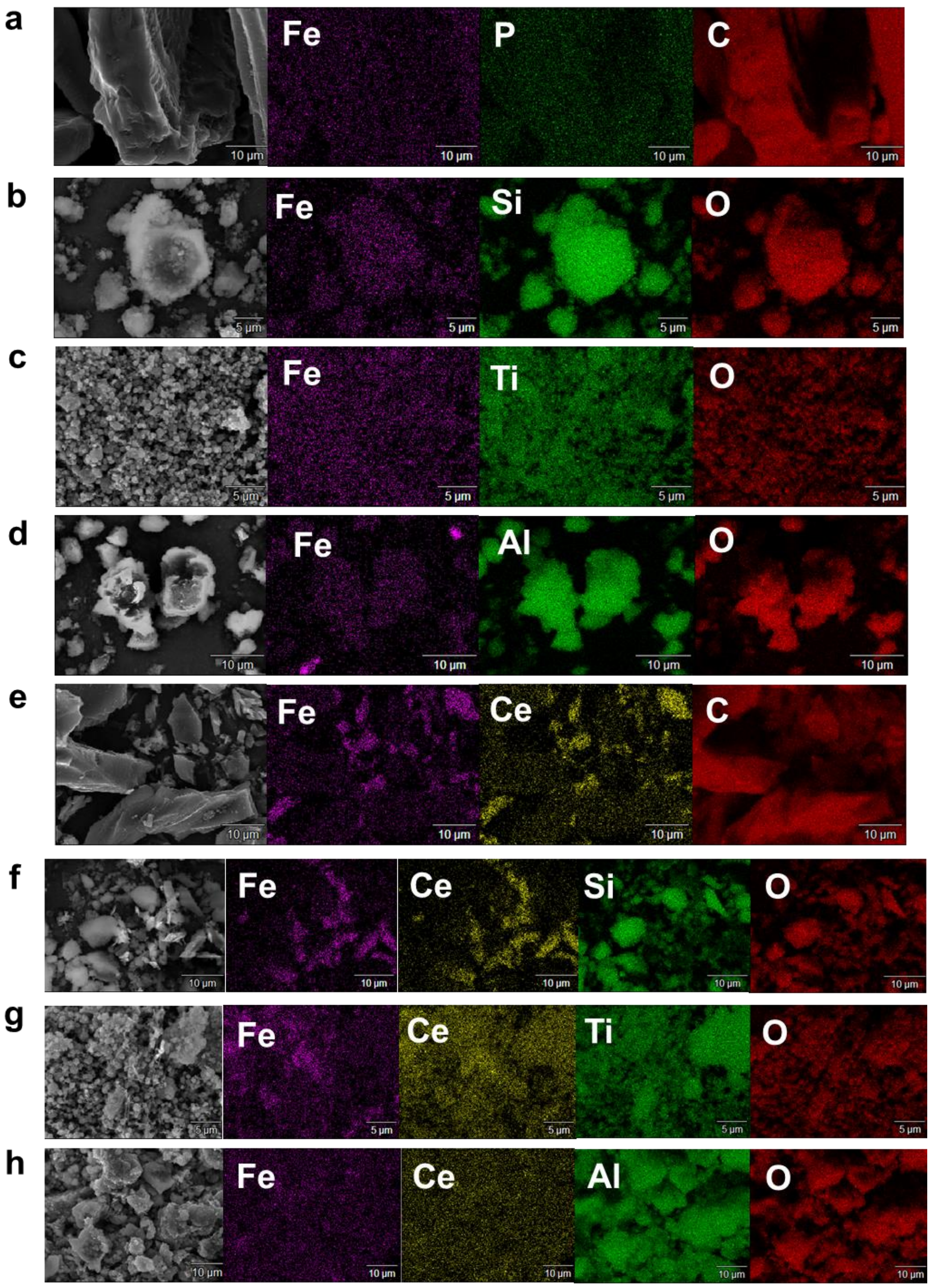

Figure S 9: SEM in combination with EDX mapping of the Fe catalysts. a) Fe/C; b) Fe/SiO_2; c) Fe/TiO_2; d) Fe/Al_2O_3; e) FeCe/C; f) FeCe/SiO_2; g) FeCe/TiO_2; h) FeCe/Al_2O_3. The measurement revealed a homogeneous distribution of iron and if available cerium on the surface of the support materials. The phosphorus found in the measurement of the carbon supported catalysts is due to a previous activation of the support material.

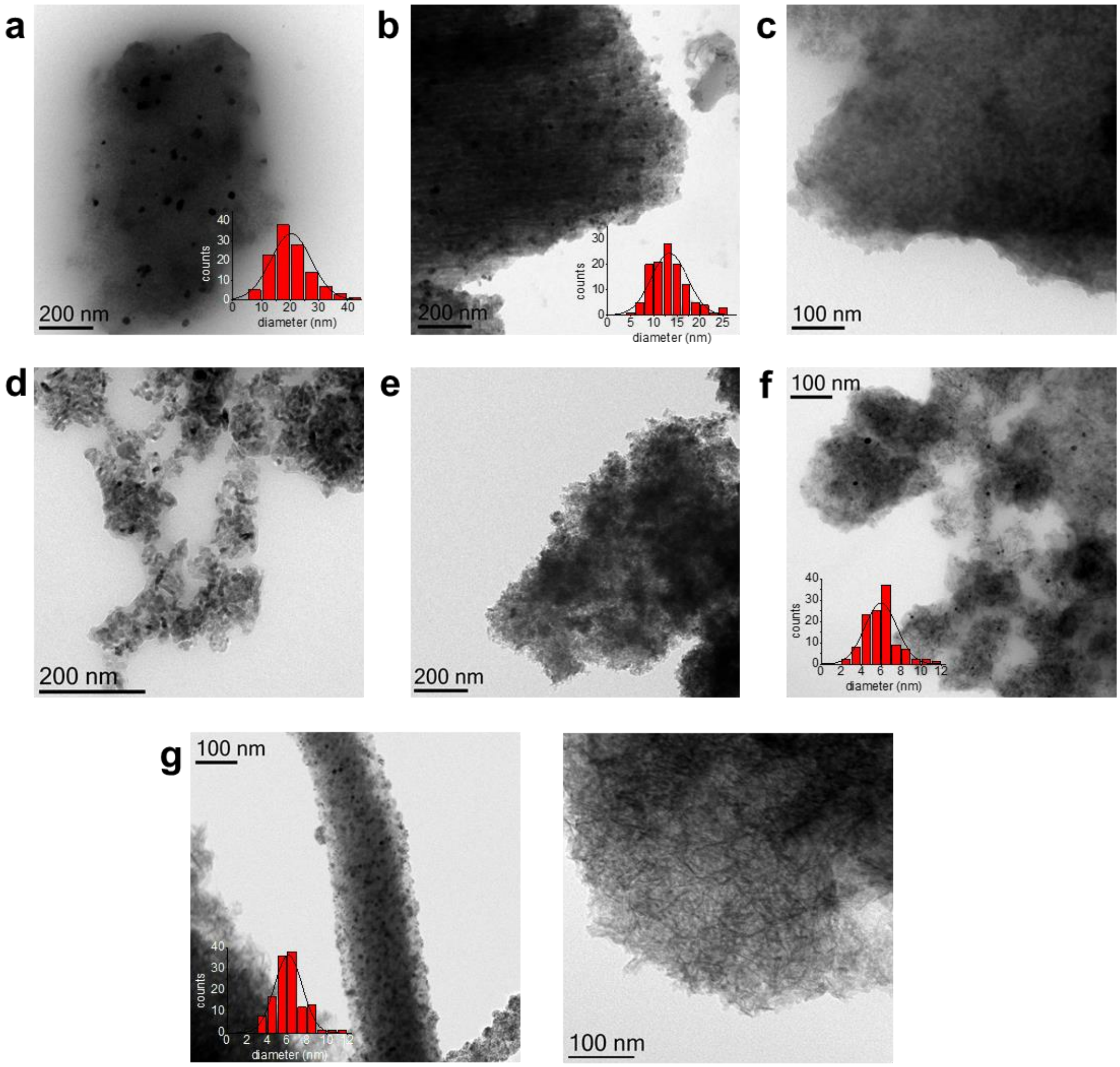

Figure S 10: Characterization of the Ni catalysts by transmissions electron microscopy (TEM). a) For the Ni/C catalyst a homogenous distribution of nanoparticles with an average particle size of 20.2 ± 7.1 nm (counted particles: 119) could be observed. b) For the NiCe/C catalyst a homogenous distribution of nanoparticles with an average particle size of 13.4 ± 3.9 nm (counted particles: 120) could be observed. c) For the NiCe/SiO_2 catalyst no nanoparticles could be observed all over the support material. d) For the Ni/TiO_2 catalyst no nanoparticles could be observed all over the support material. e) For the NiCe/TiO_2 catalyst no nanoparticles could be observed all over the support material. f) For the Ni/Al_2O_3 catalyst a homogenous distribution of nanoparticles with an average particle size of 6.0 ± 1.6 nm (counted particles: 116) could be observed. g) The measurements of the NiCe/Al_2O_3 catalyst revealed two different material species coexist. One species showed a homogenous distribution of nanoparticles with an average particle size of 6.1 ± 1.4 nm (counted particles: 127) (left), while for the other species no nanoparticles could be observed (right).

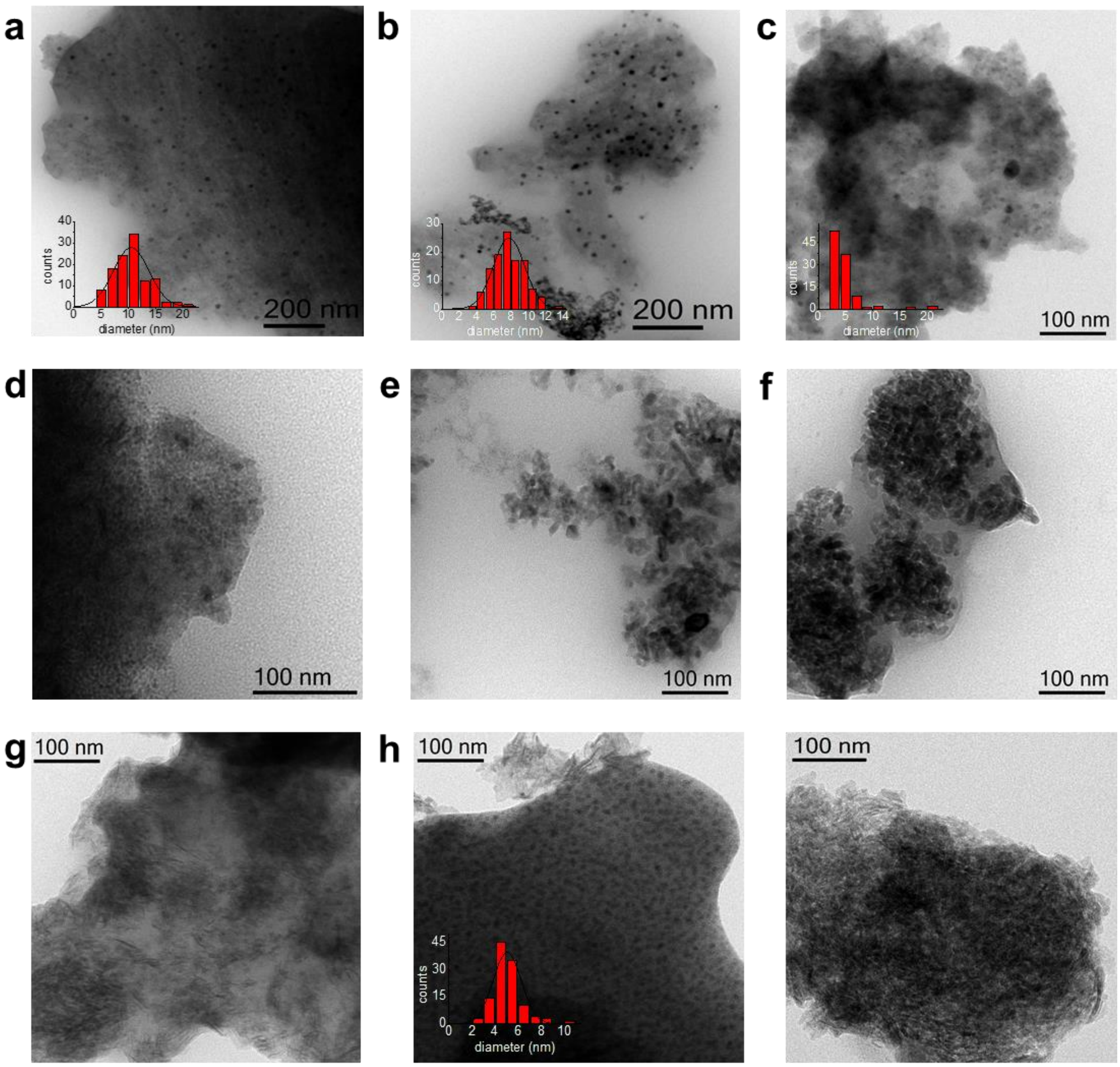

Figure S 11: Characterization of the Co catalysts by TEM. a) For the Co/C catalyst a homogenous distribution of nanoparticles with an average particle size of 10.5 ± 3.3 nm (counted particles: 114) could be observed. b) For the CoCe/C catalyst a homogenous distribution of nanoparticles with an average particle size of 7.7 ± 1.8 nm (counted particles: 113) could be observed. c) For the Co/SiO_2 catalyst an inhomogeneous distribution of nanoparticles with particles between 2-12 nm and 16-22 nm (counted particles: 105) could be observed. d) For the CoCe/SiO_2 catalyst no nanoparticles could be identified all over the support material. e) For the Fe/TiO_2 catalyst no nanoparticles could be observed all over the support material. f) For the FeCe/TiO_2 catalyst no nanoparticles could be observed all over the support material. g) For the Co/Al_2O_3 catalyst no nanoparticles could be identified all over the support material. h) The measurements of the CoCe/Al_2O_3 catalyst revealed two different material species coexist. One species showed a homogenous distribution of nanoparticles with an average particle size of 5.1 ± 1.2 nm (counted particles: 115) (left), while for the other species no nanoparticles could be observed (right).

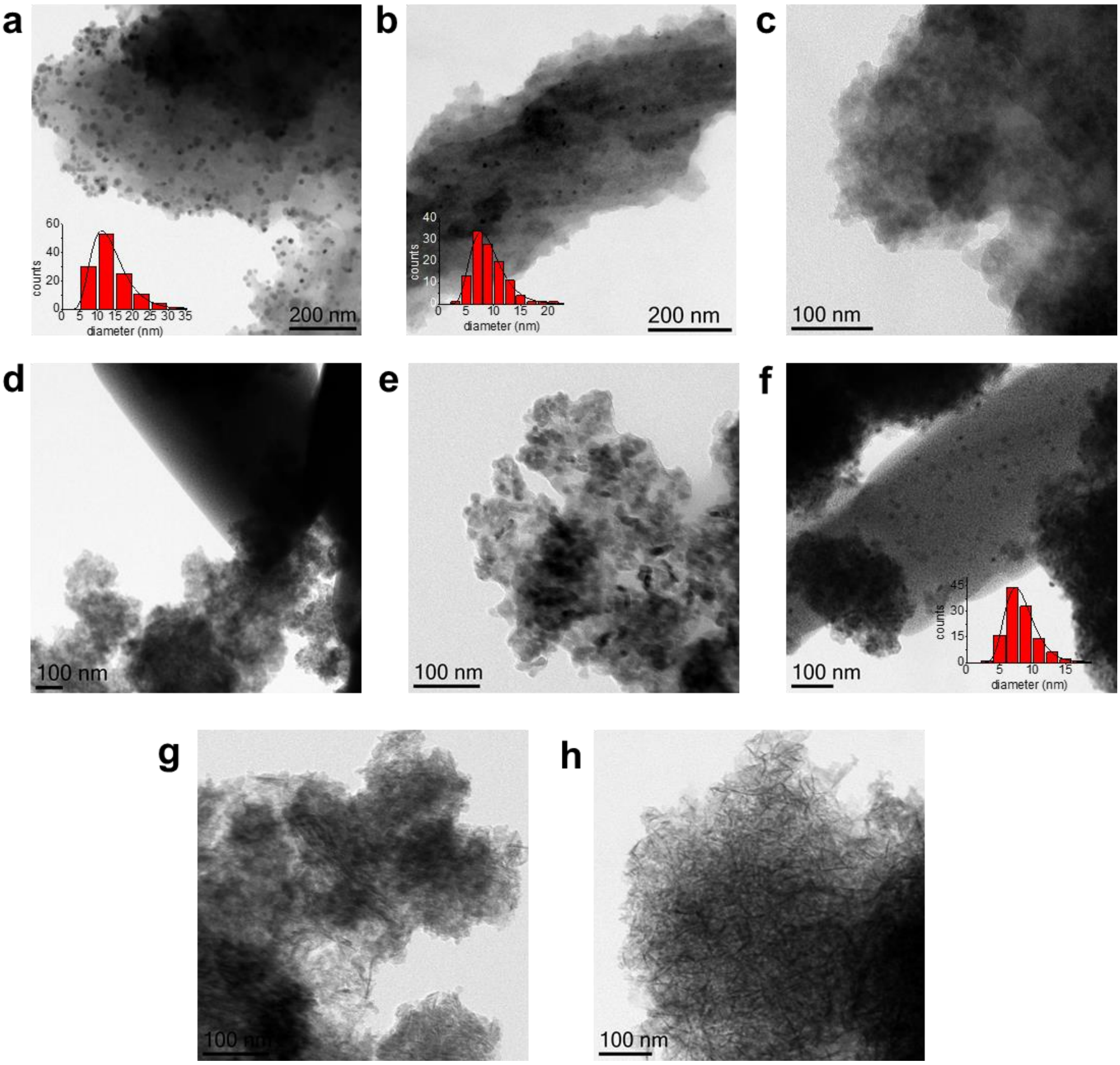

Figure S 12: Characterization of the Fe catalysts by TEM. a) For the Fe/C catalyst a homogenous distribution of nanoparticles with an average particle size of 13.8 ± 5.2 nm (counted particles: 124) could be observed. b) For the FeCe/C catalyst a homogenous distribution of nanoparticles with an average particle size of 9.1 ± 3.1 nm (counted particles: 114) could be observed. c) For the Fe/SiO_2 catalyst no nanoparticles could be identified all over the support material. d) The measurements of the FeCe/SiO_2 catalyst revealed two different material species coexist. No nanoparticles could be identified all over the support material for either catalyst. e) For the Fe/TiO_2 catalyst no nanoparticles could be observed all over the support material. f) The measurements of the FeCe/TiO_2 catalyst revealed two different material species coexist. One species showed a homogenous distribution of nanoparticles with an average particle size of 8.3 ± 2.3 nm (counted particles: 117), while for the other species no nanoparticles could be observed. g) For the Fe/Al_2O_3 catalyst no nanoparticles could be identified all over the support material. h) For the FeCe/Al_2O_3 catalyst no nanoparticles could be identified all over the support material.

5. Catalytic studies – Hydrogenation of nitrobenzene

Table S 3: Solvent screening of the catalyst library based on the hydrogenation of the model substrate nitrobenzene.

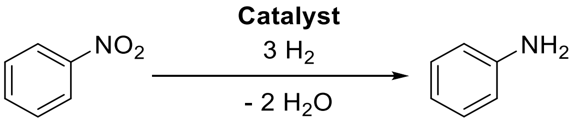

Catalyst/Solvent	Yield (%)[d]					
	H_2O	EtOH	THF	Toluene	MCH	Dioxane
Ni/C [a)]	0	0	0	0	0	0
Ni/SiO_2 [a)]	>99	35	>99	>99	>99	12
Ni/TiO_2 [a)]	10	62	65	>99	69	0
Ni/Al_2O_3 [a)]	78	>99	6	90	92	4
NiCe/C [a)]	4	14	4	15	5	0
$NiCe/SiO_2$ [a)]	73	1	2	2	1	0
$NiCe/TiO_2$ [a)]	0	14	4	15	5	0
$NiCe/Al_2O_3$ [a)]	10	3	1	0	1	0
Co/C [b)]	6	18	5	0	3	0
Co/SiO_2 [b)]	34	47	36	32	56	0
Co/TiO_2 [b)]	23	95	63	51	38	5
Co/Al_2O_3 [b)]	11	1	0	0	0	0
CoCe/C [b)]	48	88	95	84	>99	23
$CoCe/SiO_2$ [b)]	0	0	2	2	4	0
$CoCe/TiO_2$ [b)]	43	17	18	9	5	2
$CoCe/Al_2O_3$ [b)]	25	9	11	2	5	0
Fe/C [c)]	11	-	10	15	12	-
Fe/SiO_2 [c)]	2	-	0	1	0	-
Fe/TiO_2 [c)]	6	-	7	12	12	-
Fe/Al_2O_3 [c)]	7	-	2	0	7	-
FeCe/C [c)]	8	-	3	2	0	-
$FeCe/SiO_2$ [c)]	2	-	0	2	0	-
$FeCe/TiO_2$ [c)]	2	-	2	0	0	-
$FeCe/Al_2O_3$ [c)]	5	-	2	2	0	-

Reaction conditions: 0.5 mmol nitrobenzene, 20 h, 3 mL solvent; a) 0.8 mol% Ni, 90 °C, 50 bar H_2; b) 2.5 mol% Co, 110 °C, 50 bar H_2; c) 10 mol% Fe, 120 °C, 60 bar H_2; d) Yields were determined by GC using *n*-dodecane as an internal standard.

Table S 4: Screening for the most active nickel catalyst and optimization of the reaction conditions of the Ni/SiO_2 catalyst.

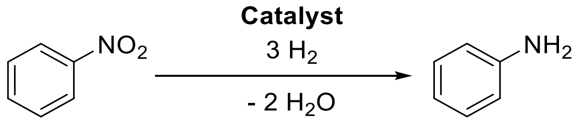

Catalyst	Solvent	Temperature (°C)	Pressure (bar)	Yield (%)[a)]
Ni/TiO_2	Toluene	80	20	10
Ni/Al_2O_3	EtOH	80	20	26
Ni/SiO_2	MCH	80	20	>99
Ni/SiO_2	THF	80	20	60
Ni/SiO_2	H_2O	70	10	23
Ni/SiO_2	Toluene	70	10	95
Ni/SiO_2	MCH	70	10	>99
Ni/SiO_2	MCH	60	10	>99
Ni/SiO_2	MCH	50	10	>99
Ni/SiO_2	MCH	40	10	99
Ni/SiO_2	MCH	30	10	57

Reaction conditions: 0.5 mmol nitrobenzene, 0.8 mol% Ni, 20 h, 3 mL solvent; a) Yields were determined by GC using *n*-dodecane as an internal standard.

Table S 5: Comparison of the Ni/SiO_2 catalyst with SiO_2 supported catalysts using different nickel precursors.

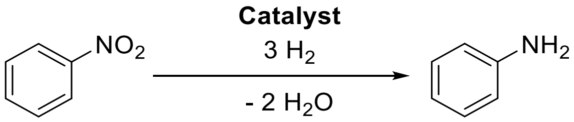

Metal source	Yield (%)[a)]
Ni-sal	>99
$Ni(OAc)_2 \cdot 4\ H_2O$	9
$Ni(NO_3)_2 \cdot 6\ H_2O$	0
$NiCl_2 \cdot 6\ H_2O$	0
$Ni(acac)_2$	32

Reaction conditions: 0.5 mmol nitrobenzene, 0.8 mol% Ni, 40 °C, 10 bar H_2, 3 mL MCH, 20 h; a) Yields were determined by GC using *n*-dodecane as an internal standard.

6. Characterization of the Ni/SiO_2 catalyst

ICP-OES

Theoretical Ni content: 3.0 wt%

Measured Ni content: 2.8 wt %

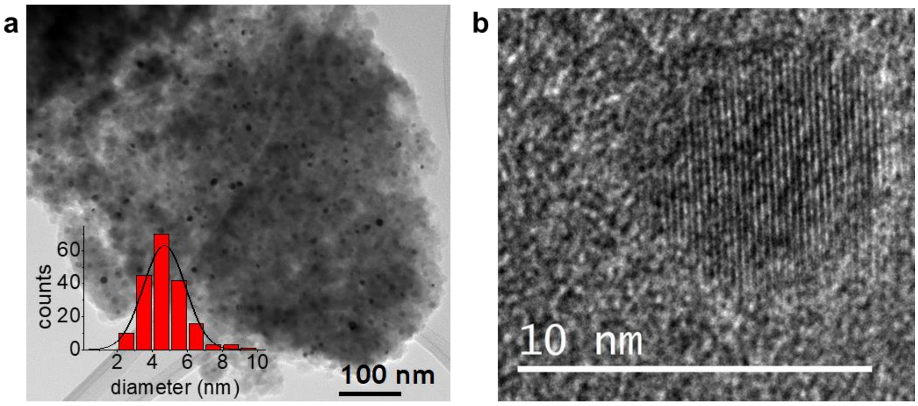

Figure S 13: Characterization of the Ni/SiO_2 catalysts by TEM. a) TEM analysis of the Ni/SiO_2 catalyst suggested the presence of homogeneously distributed nanoparticles on the SiO_2 support with an average diameter of 4.7 ± 1.2 nm (counted particles: 190). b) High-resolution (HR)TEM micrograph of one nanoparticle.

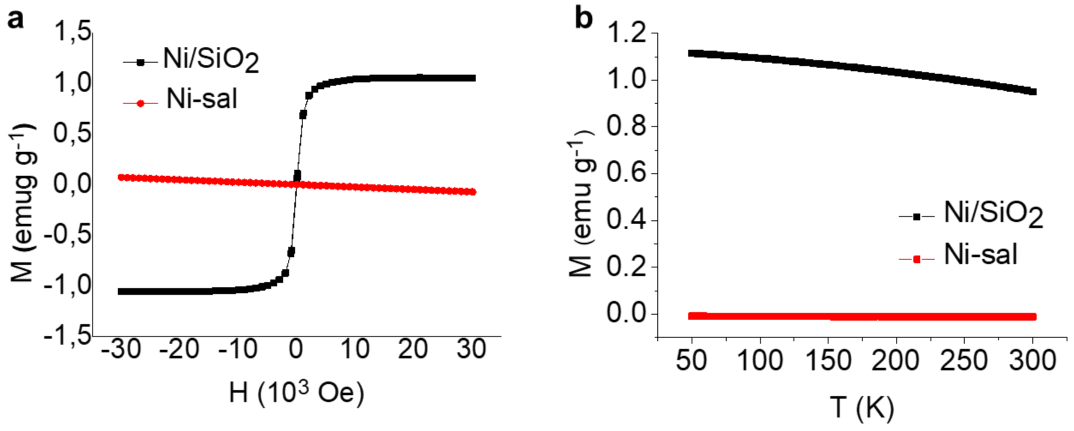

Figure S14: Characterization of the magnetic properties of the catalyst a) Magnetic measurements of the nickel salen complex (red) and the Ni/SiO_2 catalyst (black) at 27 °C. The complex showed the expected diamagnetic behavior of Ni^{2+}. A change to superparamagnetism was observed due to the formation of Ni nanoparticles during the catalyst synthesis via pyrolysis and reduction. b) Temperature-dependent magnetization of the Ni-sal complex (red) and the Ni/SiO_2 catalyst (black). For the complex, no magnetization could be observed all over the temperature range, typical for a diamagnetic species. In contrast, the magnetic catalyst shows a magnetization of 0.95 $emug^{-1}$ at 27 °C and 1.12 $emug^{-1}$ at -233 °C.

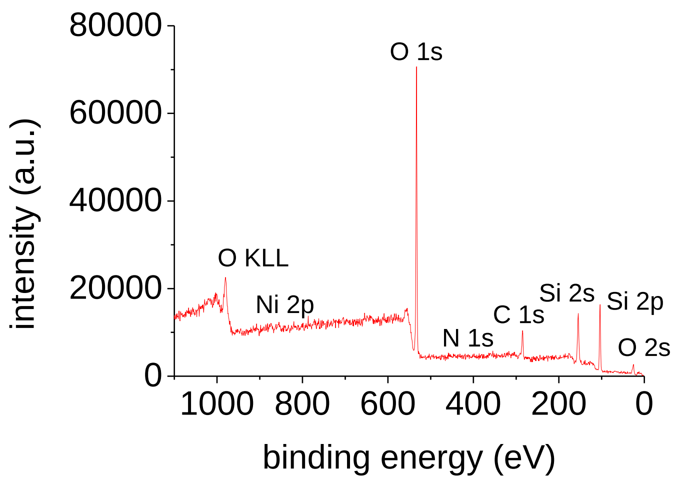

Figure S 15: X-ray photoelectron spectrum (XPS) of the Ni/SiO_2 catalyst. Survey spectrum.

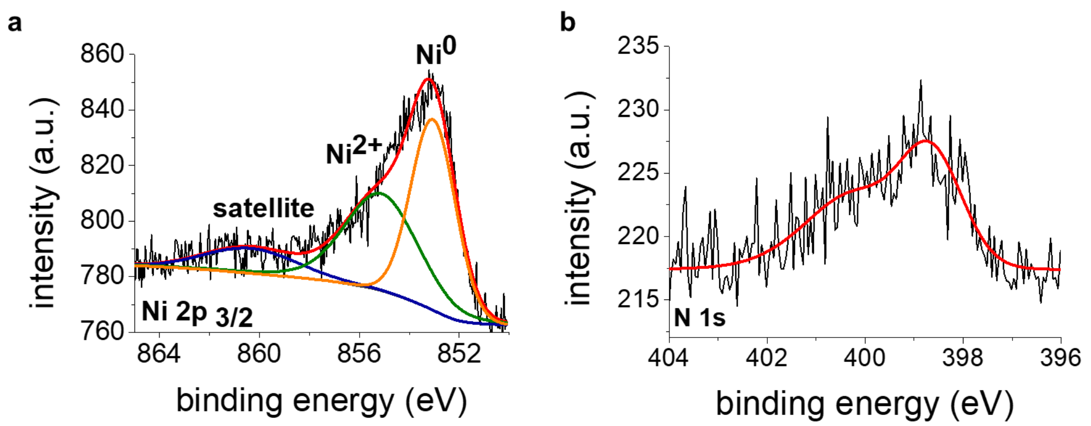

Figure S 16: XPS spectra of the Ni/SiO_2 catalyst. a) XPS of the Ni 2p3/2 region indicates two different Ni species that can be identified as Ni^0 for metallic nickel and Ni^{2+} for an oxidic nickel species. The ration of Ni^0:Ni^{2+} is approximately 1.1:1. b) Analysis of the nitrogen N 1s region revealed traces of nitrogen. Due of the low intensity, a more detailed analysis of the signals is not possible.

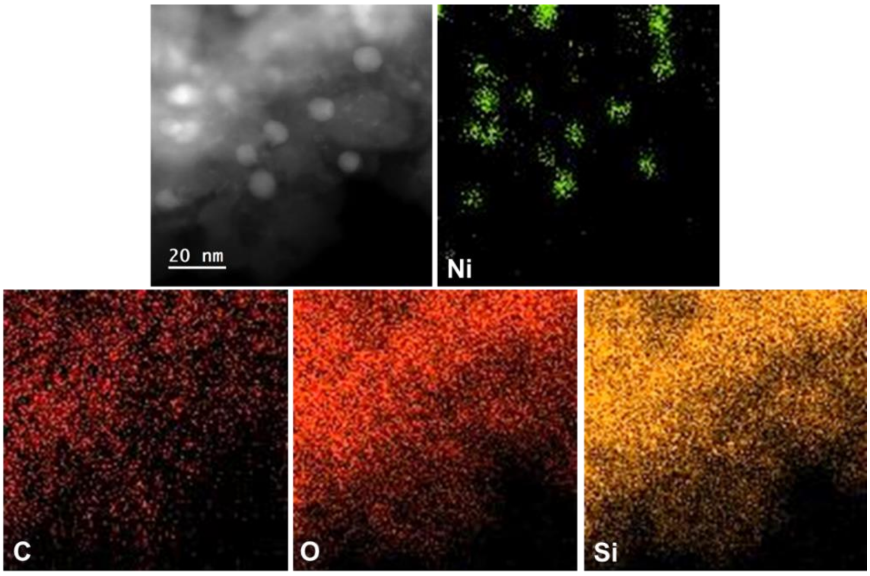

Figure S 17: Characterization of the Ni/SiO_2 catalyst with high-angle annular dark-field scanning TEM (HAADF-STEM) combined with EDX element maps. SiO_2 (Si: yellow, O: orange) is homogeneously covered with Ni nanoparticles (Ni: green). The nanoparticles are embedded in a carbon layer (C: red).

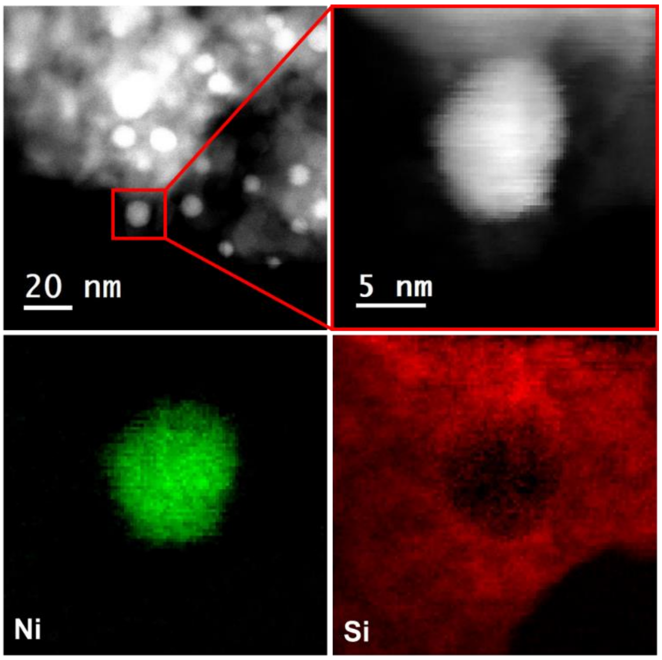

Figure S 18: Analysis of a HAADF–STEM combined with electron energy loss spectroscopy (EELS). Element maps prove the presence of nickel nanoparticles (Ni: green) on the SiO_2 support (Si: red).

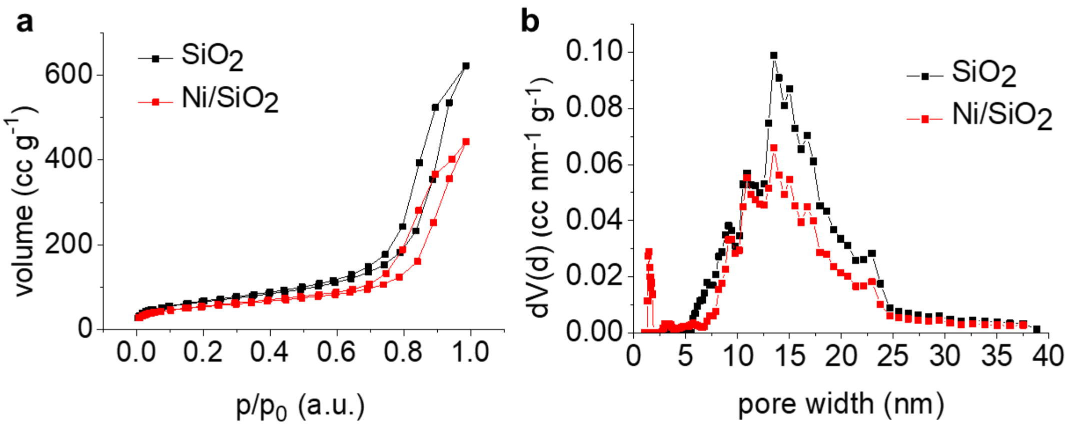

Figure S 19: Characterization of the surface area of the Ni/SiO_2 catalyst by N_2-physisorption measurement. a) Isotherms of the SiO_2 support (black) and the Ni/SiO_2 catalyst (red). The materials show the typical hysteresis of mesoporous materials. After the impregnation of the SiO_2 support with the Ni-salen complex, a decrease of the calculated surface area from 233 to 188 $m^2\ g^{-1}$ was observed. b) Corresponding calculated pore size distributions of the support (black) and the catalyst (red) [N_2 at -196.15 °C on carbon (slit and cylindrical pores, NLDFT equilibrium model)]. No significant differences were noticed.

7. Catalytic studies – Hydrogenation of nitro ketones

Table S 6: Screening of the catalyst loading.

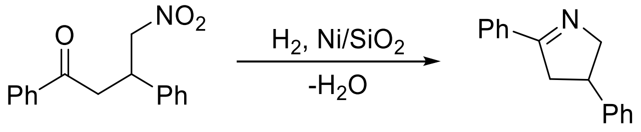

Catalyst loading	Yield (%)[a)]
5 mol% Ni	71
4 mol% Ni	75
3 mol% Ni	71
2 mol% Ni	65
1 mol% Ni	60

Reaction conditions: 0.2 mmol nitro ketone, 3 mL MeCN, 100 °C, 20 bar H_2, 20 h; a) Yields were determined by GC using *n*-dodecane as an internal standard.

Table S 7: Screening of the hydrogen pressure.

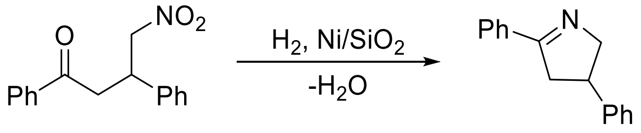

Pressure (bar)	Yield (%)[a)]
30	73
20	75
10	71

Reaction conditions: 0.2 mmol nitro ketone, 4 mol% Ni (2.8 wt% Ni, 0.008 mmol Ni, 0.48 mg Ni), 3 mL MeCN, 100 °C, 20 h; a) Yields were determined by GC using *n*-dodecane as an internal standard.

Catalytic activity of Ni/SiO_2 after leaching with HCl

To determine the catalytic activity of the Ni/SiO_2 catalyst after leaching with HCl the hydrogenation of 4-nitro-1,3-diphenylbutan-1-one was carried out under standard conditions:

A 10 mL reaction vial was charged with a magnetic stirring bar, 0.2 mmol 4-nitro-1,3-diphenylbutan-1-one, 3 mL MeCN and 17 mg catalyst (Ni/SiO_2 after leaching with 2M HCl). The vial was placed in a 300 mL high-pressure autoclave (Parr Instruments). The autoclave was flushed with 20 bar hydrogen three times before the final pressure (20 bar) was applied. The reaction was stirred at 120 °C for 20 h. Afterwards the autoclave was cooled to room temperature and the hydrogen was released. For quantitative GC analysis *n*-dodecane was added as an internal standard.

GC analysis showed that the catalyst was no longer active after acid leaching as no conversion of the substrate occurred.

Reusability of the Ni/SiO_2 catalyst

The reusability of the Ni/SiO_2 catalyst was investigated using the hydrogenation of 4-nitro-1,3-diphenylbutan-1-one to 3,5-diphenyl-3,4-dihydro-2*H*-pyrrole. For this study, the optimized reaction conditions (4 mol% Ni, 120 °C, 20 bar H_2, 3 mL MeCN) were used for 16 h. The yield of ,5-diphenyl-3,4-dihydro-2*H*-pyrrole was determined by GC using *n*-dodecane as an internal standard. After each run, the reaction mixture was separated using Pasteur pipettes. The catalyst was washed several times with ethyl acetate and used directly for the subsequent run. This procedure was carried out for 5 times without a significant decrease of activity (see figure below).

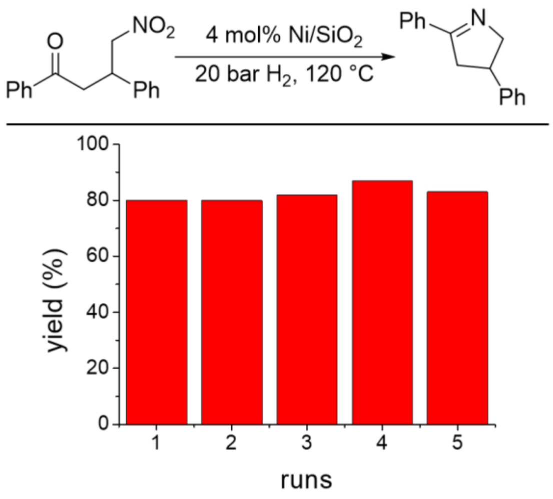

Figure S 20: Reusability of the Ni/SiO_2 catalyst using the hydrogenation of 4-nitro-1,3-diphenylbutan-1-one.

Upscaling

4-nitro-1,3-diphenylbutan-1-one was used as model substrate to perform an upscaling experiment. For the reaction, 1.35 g educt (5 mmol), 419 mg Ni/SiO_2 (2.8 wt%, 4 mol%) and 15 mL Acetonitrile were stirred in a 250 mL high pressure autoclave (Parr Instruments) at 120 °C and 20 bar H_2 for 20 h. The workup was carried out analogously to the 0.2 mmol reactions. The isolated yield on 3,5-diphenyl-3,4-dihydro-2*H*-pyrrole was 90 % (944 mg).

8. Characterization of the nitro ketones

4-nitro-1,3-diphenylbutan-1-one

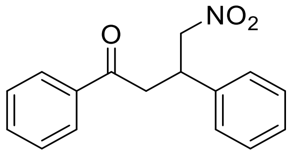

MW ($C_{16}H_{15}NO_3$) = 269.30 g mol^{-1}

^{1}H NMR (500 MHz, CD_2Cl_2): δ = 7.92 (dd, *J* = 8.4, 1.2 Hz, 2H), 7.62 – 7.57 (m, 1H), 7.50 – 7.45 (m, 2H), 7.38 – 7.25 (m, 5H), 4.84 (dd, *J* = 12.5, 6.3 Hz, 1H), 4.69 (dd, *J* = 12.5, 8.5 Hz, 1H), 4.24 – 4.16 (m, 1H), 3.46 (ddd, *J* = 25.3, 17.8, 7.0 Hz, 2H) ppm.

^{13}C NMR (125.7 MHz, CD_2Cl_2): δ = 197.13, 139.81, 136.83, 133.87, 129.31, 129.09, 128.30, 128.13, 127.92, 80.14, 41.91, 39.70 ppm.

Yield: 70 % (13.9 mmol, 3.74 g), white solid.

4-nitro-3-phenyl-1-(*p*-tolyl)butan-1-one

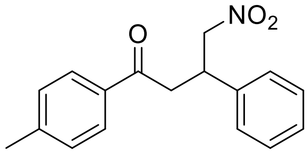

MW ($C_{17}H_{17}NO_3$) = 283.33 g mol^{-1}

^{1}H NMR (500 MHz, CD_2Cl_2): δ = 7.82 (d, *J* = 8.2 Hz, 2H), 7.37 – 7.25 (m, 7H), 4.84 (dd, *J* = 12.5, 6.3 Hz, 1H), 4.67 (dd, *J* = 12.5, 8.6 Hz, 1H), 4.23 – 4.15 (m, 1H), 3.42 (ddd, *J* = 25.3, 17.7, 7.0 Hz, 2H), 2.40 (s, 3H). ppm.

^{13}C NMR (100 MHz, CD_2Cl_2): δ = 197.28, 139.89, 139.09, 136.85, 134.59, 129.29, 128.91, 128.84, 128.09, 127.92, 125.47, 80.14, 41.97, 39.73, 21.43 ppm.

Yield: 53 % (0.79 mmol, 225 mg), white solid.

4-nitro-3-phenyl-1-(*m*-tolyl)butan-1-one

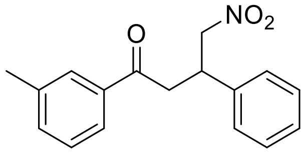

MW ($C_{17}H_{17}NO_3$) = 283.33 g mol^{-1}

^{1}H NMR (400 MHz, CD_2Cl_2): δ = 7.72 (d, *J* = 12.3 Hz, 2H), 7.43 – 7.27 (m, 7H), 4.84 (dd, *J* = 12.5, 6.3 Hz, 1H), 4.69 (dd, *J* = 12.5, 8.5 Hz, 1H), 4.24 – 4.16 (m, 1H), 3.44 (qd, *J* = 17.8, 7.0 Hz, 2H), 2.40 (s, 3H).

^{13}C NMR (100 MHz, CD_2Cl_2): δ = 196.70, 144.97, 139.92, 134.37, 129.74, 129.29, 128.40, 128.09, 127.92, 80.19, 41.79, 39.77, 21.78 ppm.

Yield: 92 % (1.38 mmol, 392 mg), white solid.

4-nitro-3-phenyl-1-(*o*-tolyl)butan-1-one

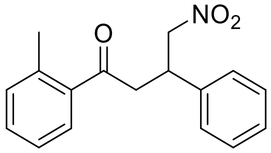

MW ($C_{17}H_{17}NO_3$) = 283.33 g mol^{-1}

^{1}H NMR (400 MHz, CD_2Cl_2): δ = 7.59 (d, *J* = 7.6 Hz, 1H), 7.42 – 7.21 (m, 8H), 4.80 (dd, *J* = 12.4, 6.6 Hz, 1H), 4.68 (dd, *J* = 12.4, 8.3 Hz, 1H), 4.20 – 4.11 (m, 1H), 3.44 – 3.29 (m, 2H), 2.33 (s, 3H) ppm.

^{13}C NMR (100 MHz, CD_2Cl_2): δ = 201.01, 139.59, 138.70, 137.69, 132.38, 132.00, 128.68, 128.15, 127.97, 126.11, 80.24, 44.61, 40.10, 21.22 ppm.

Yield: 49 % (0.74 mmol, 209 mg), white solid.

1-(4-chlorophenyl)-4-nitro-3-phenylbutan-1-one

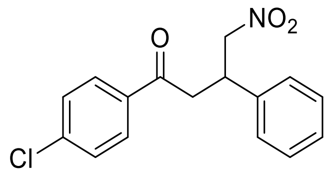

MW ($C_{16}H_{14}ClNO_3$) = 303.74 g mol^{-1}

^{1}H NMR (500 MHz, CD_2Cl_2): δ = 7.89 – 7.84 (m, 2H), 7.47 – 7.43 (m, 2H), 7.37 – 7.25 (m, 5H), 4.82 (dd, *J* = 12.5, 6.5 Hz, 1H), 4.68 (dd, *J* = 12.5, 8.3 Hz, 1H), 4.24 – 4.13 (m, 1H), 3.43 (qd, *J* = 17.8, 6.9 Hz, 2H) ppm.

^{13}C NMR (100 MHz, CD_2Cl_2): δ = 195.54, 139.72, 139.16, 134.76, 129.34, 128.92 128.89, 127.73, 127.44, 79.61, 41.44, 39.21 ppm.

Yield: 68 % (2.04 mmol, 620 mg), white solid.

1-(3-chlorophenyl)-4-nitro-3-phenylbutan-1-one

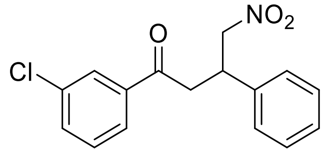

MW ($C_{16}H_{14}ClNO_3$) = 303.74 g mol^{-1}

^{1}H NMR (400 MHz, CD_2Cl_2): δ = 7.90 – 7.89 (m, 1H), 7.82 – 7.79 (m, 1H), 7.57 (ddd, *J* = 8.0, 2.1, 1.1 Hz, 1H), 7.43 (t, *J* = 7.9 Hz, 1H), 7.38 – 7.27 (m, 5H), 4.82 (dd, *J* = 12.5, 6.5 Hz, 1H), 4.69 (dd, *J* = 12.5, 8.2 Hz, 1H), 4.24 – 4.15 (m, 1H), 3.44 (qd, *J* = 18.0, 7.0 Hz, 2H) ppm.

^{13}C NMR (100 MHz, CD_2Cl_2): δ = 195.96, 139.54, 138.36, 135.31, 133.74, 130.54, 129.36, 128.39, 128.23, 127.90, 126.52, 80.03, 42.06, 39.61 ppm.

Yield: 51 % (0.76 mmol, 231 mg), white solid.

1-(4-bromophenyl)-4-nitro-3-phenylbutan-1-one

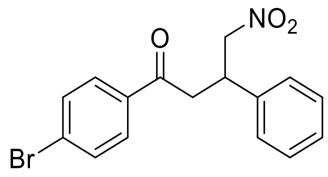

MW ($C_{16}H_{14}BrNO_3$) = 348.20 g mol^{-1}

^{1}H NMR (500 MHz, CD_2Cl_2): δ = 7.81 – 7.77 (m, 2H), 7.65 – 7.60 (m, 2H), 7.37 – 7.25 (m, 5H), 4.82 (dd, *J* = 12.5, 6.5 Hz, 1H), 4.68 (dd, *J* = 12.5, 8.3 Hz, 1H), 4.23 – 4.14 (m, 1H), 3.42 (qd, *J* = 17.8, 6.9 Hz, 2H) ppm.

^{1}H NMR (500 MHz, CD_2Cl_2): δ = 7.81 – 7.77 (m, 2H), 7.65 – 7.60 (m, 2H), 7.37 – 7.25 (m, 5H), 4.82 (dd, *J* = 12.5, 6.5 Hz, 1H), 4.68 (dd, *J* = 12.5, 8.3 Hz, 1H), 4.23 – 4.14 (m, 1H), 3.42 (qd, *J* = 17.8, 6.9 Hz, 2H) ppm.

^{13}C NMR (100 MHz, CD_2Cl_2): δ = 193.21, 139.61, 135.62, 132.40, 129.89, 129.35, 128.94, 128.20, 127.90, 80.06, 41.89, 39.66 ppm.

Yield: 80 % (1.21 mmol, 420 mg), white solid.

1-(4-fluorophenyl)-4-nitro-3-phenylbutan-1-one

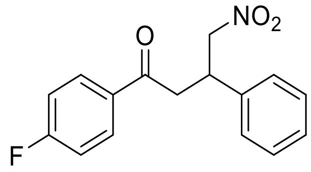

MW ($C_{16}H_{14}FNO_3$) = 387.29 g mol^{-1}

^{1}H NMR (500 MHz, $CDCl_3$): δ = 7.98 – 7.92 (m, 2H), 7.34 (dd, *J* = 10.4, 4.4 Hz, 2H), 7.30 – 7.26 (m, 3H), 7.16 – 7.09 (m, 2H), 4.82 (dd, *J* = 12.5, 6.8 Hz, 1H), 4.69 (dd, *J* = 12.5, 7.8 Hz, 1H), 4.21 (p, *J* = 7.1 Hz, 1H), 3.50 – 3.36 (m, 2H) ppm.

^{13}C NMR (125.7 MHz, $CDCl_3$): δ = 195.38, 167.14, 139.10, 132.93, 130.89, 130.82, 129.26, 128.10, 127.57, 116.13, 115.96, 79.66, 41.56, 39.41 ppm.

Yield: 46 % (0.69 mmol, 190 mg), white solid.

1-(4-methoxyphenyl)-4-nitro-3-phenylbutan-1-one

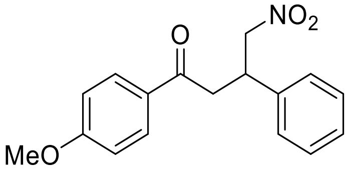

MW ($C_{17}H_{17}NO_4$) = 299.33 g mol^{-1}

^{1}H NMR (500 MHz, CD_2Cl_2): δ = 7.93 – 7.87 (m, 2H), 7.37 – 7.24 (m, 5H), 6.96 – 6.91 (m, 2H), 4.84 (dd, *J* = 12.5, 6.2 Hz, 1H), 4.67 (dd, *J* = 12.5, 8.6 Hz, 1H), 4.22 – 4.14 (m, 1H), 3.86 (s, 3H), 3.39 (ddd, *J* = 25.2, 17.5, 7.0 Hz, 2H) ppm.

^{13}C NMR (100 MHz, CD_2Cl_2): δ = 195.52, 164.23, 139.99, 130.59, 129.88, 129.26, 128.05, 127.91, 114.18, 80.19, 55.92, 41.54, 39.84 ppm.

Yield: 69 % (2.80 mmol, 623 mg), white solid.

1-(4-hydroxyphenyl)-4-nitro-3-phenylbutan-1-one

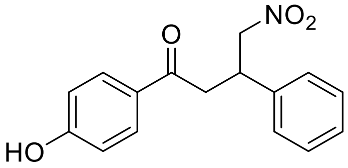

MW ($C_{16}H_{16}NO_4$) = 285.30 g mol^{-1}

^{1}H NMR (400 MHz, DMSO-D_6): δ = 10.35 (s, 1H), 7.75 (d, *J* = 8.5 Hz, 2H), 7.30 (d, *J* = 7.2 Hz, 2H), 7.23 (t, *J* = 7.6 Hz, 2H), 7.15 (t, *J* = 7.5 Hz, 1H), 6.76 (d, *J* = 8.5 Hz, 2H), 4.91 (dd, *J* = 12.8, 5.6 Hz, 1H), 4.79 (dd, *J* = 12.9, 9.9 Hz, 1H), 3.95 (dt, *J* = 13.3, 7.0 Hz, 1H), 3.43 – 3.31 (m, 2H) ppm.

^{13}C NMR (100 MHz, DMSO-D_6): δ = 195.37, 162.22, 140.17, 130.52, 128.43, 128.02, 127.78, 127.14, 115.21, 79.73, 40.56, 39.39 ppm.

Yield: 81 % (1.22 mmol, 348 mg), white solid.

1-(2-hydroxyphenyl)-4-nitro-3-phenylbutan-1-one

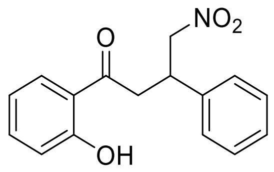

MW ($C_{16}H_{16}NO_4$) = 285.30 g mol^{-1}

^{1}H NMR (400 MHz, CD_2Cl_2): δ = 11.93 (s, 1H), 7.76 (dd, *J* = 8.1, 1.5 Hz, 1H), 7.49 (ddd, *J* = 8.5, 7.2, 1.7 Hz, 1H), 7.39 – 7.26 (m, 5H), 6.93 (dd, *J* = 17.7, 8.5 Hz, 2H), 4.82 (dd, *J* = 12.6, 6.3 Hz, 1H), 4.70 (dd, *J* = 12.6, 8.3 Hz, 1H), 4.27 – 4.16 (m, 1H), 3.50 (ddd, *J* = 40.3, 17.5, 7.2 Hz, 2H) ppm.

^{13}C NMR (100 MHz, CD_2Cl_2): δ = 203.37, 162.80, 139.35, 137.21, 130.13, 129.39, 128.28, 127.89, 119.54, 119.51, 118.85, 80.06, 41.54, 39.51 ppm.

Yield: 33 % (1.00 mmol, 258 mg), white solid.

4-nitro-3-phenyl-1-(4-(trifluoromethyl)phenyl)butan-1-one

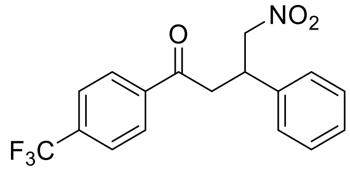

MW ($C_{17}H_{17}F_3NO_3$) = 337.30 g mol^{-1}

^{1}H NMR (400 MHz, CD_2Cl_2): δ = 8.05 – 8.01 (m, 2H), 7.77 – 7.72 (m, 2H), 7.38 – 7.28 (m, 5H), 4.83 (dd, *J* = 12.5, 6.6 Hz, 1H), 4.70 (dd, *J* = 12.5, 8.1 Hz, 1H), 4.27 – 4.17 (m, 1H), 3.56 – 3.42 (m, 2H) ppm.

^{13}C NMR (100 MHz, CD_2Cl_2): δ = 196.38, 139.45, 129.40, 128.76, 118.27, 127.90, 126.18, 126.14, 80.02, 42.25, 39.61 ppm.

Yield: 96 % (1.44 mmol, 487 mg), white solid.

4-nitro-1-phenyl-3-(*p*-tolyl)butan-1-one

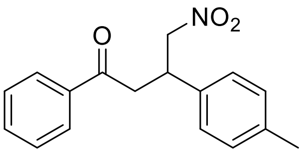

MW ($C_{17}H_{17}NO_3$) = 283.33 g mol^{-1}

^{1}H NMR (400 MHz, CD_2Cl_2): δ = 7.92 (dd, *J* = 8.4, 1.2 Hz, 2H), 7.59 (t, *J* = 7.4 Hz, 1H), 7.47 (t, *J* = 7.6 Hz, 2H), 7.22 – 7.12 (m, 4H), 4.81 (dd, *J* = 12.4, 6.4 Hz, 1H), 4.66 (dd, *J* = 12.4, 8.4 Hz, 1H), 4.22 – 4.11 (m, 1H), 3.43 (qd, *J* = 17.8, 7.0 Hz, 2H), 2.31 (s, 3H) ppm.

^{13}C NMR (100 MHz, CD_2Cl_2): δ = 197.23, 137.99, 136.90, 136.69, 133.83, 130.00, 129.08, 128.31, 127.73, 80.31, 41.97, 39.41, 21.15 ppm.

Yield: 65 % (0.97 mmol, 276 mg), white solid.

4-nitro-1-phenyl-3-(m-tolyl)butan-1-one

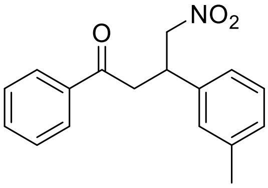

MW ($C_{17}H_{17}NO_3$) = 283.33 g mol^{-1}

^{1}H NMR (400 MHz, $CDCl_3$): δ = 7.95 – 7.90 (m, 2H), 7.61 – 7.55 (m, 1H), 7.46 (t, *J* = 7.7 Hz, 2H), 7.25 – 7.19 (m, 1H), 7.08 (d, *J* = 6.9 Hz, 3H), 4.82 (dd, *J* = 12.4, 6.7 Hz, 1H), 4.68 (dd, *J* = 12.4, 7.9 Hz, 1H), 4.24 – 4.14 (m, 1H), 3.51 – 3.37 (m, 2H), 2.33 (s, 3H) ppm.

^{13}C NMR (100 MHz, $CDCl_3$): δ = 197.03, 139.20, 138.90, 136.53, 133.69, 129.07, 128.87, 128.79, 128.39, 124.47, 79.74, 41.72, 39.36, 21.60 ppm.

Yield: 89 % (1.33 mmol, 378 mg), orange oil.

4-nitro-1-phenyl-3-(o-tolyl)butan-1-one

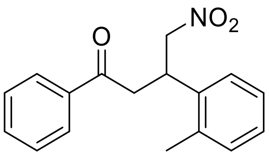

MW ($C_{17}H_{17}NO_3$) = 283.33 g mol^{-1}

^{1}H NMR (400 MHz, $CDCl_3$): δ = 7.92 (dt, *J* = 8.5, 1.6 Hz, 2H), 7.62 – 7.55 (m, 1H), 7.46 (tt, *J* = 6.6, 1.1 Hz, 2H), 7.22 – 7.12 (m, 4H), 4.79 (dd, *J* = 13.3, 6.3 Hz, 1H), 4.66 (dd, *J* = 12.4, 7.6 Hz, 1H), 4.53 (dt, *J* = 14.5, 7.3 Hz, 1H), 3.44 (qd, *J* = 17.8, 6.9 Hz, 2H), 2.47 (s, 3H) ppm.

^{13}C NMR (100 MHz, $CDCl_3$): δ = 197.05, 187.56, 137.45, 136.60, 136.67, 133.69, 131.35, 128.87, 128.14, 127.66, 126.74, 126.61, 125.49, 79.17, 41.70, 34.48, 19.74 ppm.

Yield: 95 % (1.43 mmol, 405 mg), yellow oil.

3-(4-chlorophenyl)-4-nitro-1-phenylbutan-1-one

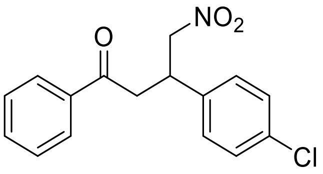

MW ($C_{16}H_{14}ClNO_3$) = 303.74 g mol^{-1}

^{1}H NMR (500 MHz, CD_2Cl_2): δ = 7.91 (dd, *J* = 8.4, 1.2 Hz, 2H), 7.62 – 7.56 (m, 1H), 7.48 (t, *J* = 7.8 Hz, 2H), 7.29 (dd, *J* = 8.7, 5.3 Hz, 2H), 7.04 (t, *J* = 8.7 Hz, 2H), 4.83 (dd, *J* = 12.5, 6.2 Hz, 1H), 4.66 (dd, *J* = 12.5, 8.6 Hz, 1H), 4.25 – 4.17 (m, 1H), 3.43 (qd, *J* = 17.8, 7.0 Hz, 2H) ppm.

^{13}C NMR (100 MHz, CD_2Cl_2): δ = 196.81, 138.40, 136.72, 133.98, 129.46, 129.42, 129.13, 128.29, 79.89, 41.78, 39.11 ppm.

Yield: 74 % (2.22 mmol, 674 mg), white solid.

3-(3-chlorophenyl)-4-nitro-1-phenylbutan-1-one

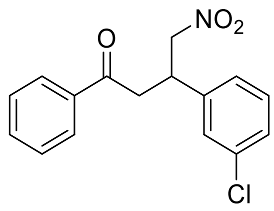

MW ($C_{16}H_{14}ClNO_3$) = 303.74 g mol^{-1}

^{1}H NMR (400 MHz, CD_2Cl_2): δ = 7.95 – 7.89 (m, 2H), 7.63 – 7.56 (m, 1H), 7.51 – 7.45 (m, 2H), 7.33 – 7.25 (m, 3H), 7.24 – 7.20 (m, 1H), 4.84 (dd, *J* = 12.7, 6.2 Hz, 1H), 4.68 (dd, *J* = 12.7, 8.6 Hz, 1H), 4.21 (ddd, *J* = 13.4, 8.5, 6.6 Hz, 1H), 3.45 (qd, *J* = 18.0, 6.9 Hz, 2H) ppm.

^{13}C NMR (100 MHz, CD_2Cl_2): δ = 196.69, 141.97, 136.65, 134.95, 134.00, 130.68, 129.13, 128.30, 128.13, 126.40, 79.72, 41.71, 39.30 ppm.

Yield: 87 % (1.31 mmol, 398 mg), white solid.

3-(2-chlorophenyl)-4-nitro-1-phenylbutan-1-one

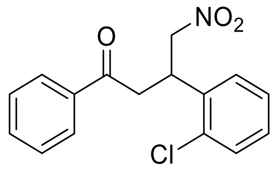

MW ($C_{16}H_{14}ClNO_3$) = 303.74 g mol^{-1}

^{1}H NMR (400 MHz, CD_2Cl_2): δ = 7.97 – 7.92 (m, 2H), 7.63 – 7.57 (m, 1H), 7.52 – 7.41 (m, 3H), 7.34 – 7.22 (m, 3H), 4.91 – 4.80 (m, 2H), 4.69 (p, *J* = 6.7 Hz, 1H), 3.58 – 3.51 (m, 2H) ppm.

^{13}C NMR (100 MHz, CD_2Cl_2): δ = 196.97, 136.90, 136.72, 133.94, 130.70, 129.34, 129.11, 128.33, 127.77, 78.16, 40.51, 36.41 ppm.

Yield: 89 % (1.33 mmol, 405 mg), yellow oil.

3-(4-bromophenyl)-4-nitro-1-phenylbutan-1-one

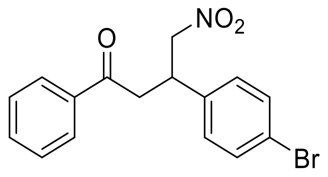

MW ($C_{16}H_{14}BrNO_3$) = 348.20 g mol^{-1}

^{1}H NMR (400 MHz, CD_2Cl_2): δ = 7.91 (dd, *J* = 8.1, 1.1 Hz, 2H), 7.60 (t, *J* = 7.3 Hz, 1H), 7.48 (ddd, *J* = 7.6, 3.6, 2.2 Hz, 4H), 7.20 (d, *J* = 8.4 Hz, 2H), 4.83 (dd, *J* = 12.7, 6.3 Hz, 1H), 4.67 (dd, *J* = 12.6, 8.6 Hz, 1H), 4.24 – 4.14 (m, 1H), 3.50 – 3.36 (m, 2H) ppm.

^{13}C NMR (100 MHz, CD_2Cl_2): δ = 166.79, 138.93, 136.74, 133.98, 132.41, 129.80, 129.14, 128.30, 121.94, 79.82, 41.73, 39.19 ppm.

Yield: 79 % (1.18 mmol, 411 mg), white solid.

3-(4-fluorophenyl)-4-nitro-1-phenylbutan-1-one

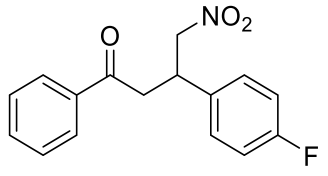

MW ($C_{16}H_{14}FNO_3$) = 387.29 g mol^{-1}

^{1}H NMR (500 MHz, CD_2Cl_2): δ = 7.91 (dd, *J* = 8.4, 1.2 Hz, 2H), 7.62 – 7.56 (m, 1H), 7.48 (t, *J* = 7.8 Hz, 2H), 7.29 (dd, *J* = 8.7, 5.3 Hz, 2H), 7.04 (t, *J* = 8.7 Hz, 2H), 4.83 (dd, *J* = 12.5, 6.2 Hz, 1H), 4.66 (dd, *J* = 12.5, 8.6 Hz, 1H), 4.25 – 4.17 (m, 1H), 3.43 (qd, *J* = 17.8, 7.0 Hz, 2H) ppm.

^{13}C NMR (100 MHz, $CDCl_3$): δ = 196.75, 163.56, 136.40, 133.82, 129.30, 129.22, 128.93, 128.14, 116.26, 116.04, 79.72, 41.67, 38.75 ppm.

Yield: 64 % (1.92 mmol, 552 mg), white solid.

3-(4-(*tert*-butyl)phenyl)-4-nitro-1-phenylbutan-1-one

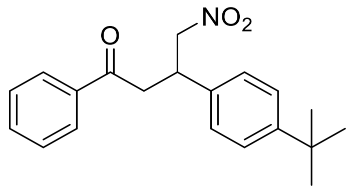

MW ($C_{20}H_{23}NO_3$) = 325.41 g mol^{-1}

^{1}H NMR (400 MHz, CD_2Cl_2): δ = 7.92 (dd, *J* = 8.3, 1.0 Hz, 2H), 7.59 (t, *J* = 7.6 Hz, 1H), 7.47 (t, *J* = 7.7 Hz, 2H), 7.37 (d, *J* = 8.3 Hz, 2H), 7.22 (d, *J* = 8.3 Hz, 2H), 4.82 (dd, *J* = 12.5, 6.5 Hz, 1H), 4.68 (dd, *J* = 12.5, 8.2 Hz, 1H), 4.22 – 4.13 (m, 1H), 3.44 (qd, *J* = 17.8, 6.8 Hz, 2H), 1.29 (s, 9H) ppm.

^{13}C NMR (100 MHz, $CDCl_3$): δ = 197.26, 151.12, 136.89, 136.71, 133.83, 129.08, 128.31, 127.51, 126.24, 80.16, 42.03, 39.21, 34.78, 31.40 ppm.

Yield: 60 % (0.90 mmol, 293 mg), white solid.

3-(4-methoxyphenyl)-4-nitro-1-phenylbutan-1-one

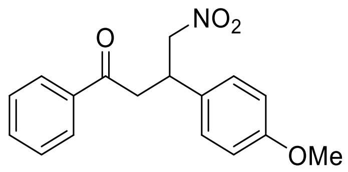

MW ($C_{17}H_{17}NO_4$) = 299.33 g mol^{-1}

^{1}H NMR (500 MHz, CD_2Cl_2): δ = 7.92 (dd, *J* = 8.3, 1.2 Hz, 2H), 7.61 – 7.56 (m, 1H), 7.47 (t, *J* = 7.8 Hz, 2H), 7.24 – 7.18 (m, 2H), 6.86 (d, *J* = 8.7 Hz, 2H), 4.80 (dd, *J* = 12.3, 6.3 Hz, 1H), 4.64 (dd, *J* = 12.3, 8.5 Hz, 1H), 4.19 – 4.10 (m, 1H), 3.77 (s, 3H), 3.42 (qd, *J* = 17.7, 7.0 Hz, 2H) ppm.

^{13}C NMR (100 MHz, CD_2Cl_2): δ = 197.26, 159.53, 136.91, 133.83, 131.57, 129.08, 128.96, 128.30, 114.60, 80.42, 55.59, 42.06, 39.07 ppm.

Yield: 77 % (2.30 mmol, 689 mg), white solid.

3-(4-(benzyloxy)phenyl)-4-nitro-1-phenylbutan-1-one

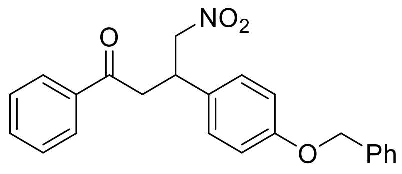

MW ($C_{23}H_{21}NO_4$) = 375.42 g mol^{-1}

^{1}H NMR (400 MHz, CD_2Cl_2): δ = 7.92 (dd, *J* = 8.2, 0.9 Hz, 2H), 7.59 (t, *J* = 7.4 Hz, 1H), 7.32-7.51 (m, 7H), 7.22 (d, *J* = 8.6 Hz, 2H), 6.94 (d, *J* = 8.7 Hz, 2H), 5.03 (s, 2H), 4.81 (dd, *J* = 12.4, 6.4 Hz, 1H), 4.64 (dd, *J* = 12.3, 8.5 Hz, 1H), 4.10-4.21 (m, 1H), 3.77 (s, 3H), 3.34-3.50 (m, 2H) ppm.

^{13}C NMR (100 MHz, CD_2Cl_2): δ = 197.25, 158.71, 137.43, 136.91, 133.83, 131.94, 129.09, 128.91, 128.31, 127.94, 115.50, 80.38, 70.37, 42.06, 39.08 ppm.

Yield: 91 % (1.36 mmol, 512 mg), white solid.

3-(4-(methylthio)phenyl)-4-nitro-1-phenylbutan-1-one

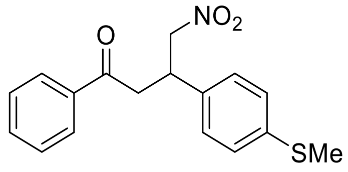

MW ($C_{17}H_{17}NO_3S$) = 315.39 g mol^{-1}

^{1}H NMR (400 MHz, $CDCl_3$): δ = 8.01 – 7.94 (m, 2H), 7.63 (t, *J* = 7.4 Hz, 1H), 7.57 – 7.48 (m, 2H), 7.35 – 7.30 (m, 1H), 7.28 (dd, *J* = 3.8, 1.2 Hz, 3H), 4.86 (dd, *J* = 12.5, 6.6 Hz, 1H), 4.77 – 4.67 (m, 1H), 4.24 (dd, *J* = 14.7, 7.0 Hz, 1H), 3.58 – 3.49 (m, 2H), 2.51 (s, 3H) ppm.

^{13}C NMR (100 MHz, $CDCl_3$): δ = 196.86, 138.38, 136.45, 135.87, 133.74, 128.89, 128.14, 128.06, 127.15, 79.64, 41.58, 38.94, 15.80 ppm.

Yield: 76 % (1.14 mmol, 359 mg), yellow solid.

4-nitro-1-phenyl-3-(4-(trifluoromethyl)phenyl)butan-1-one

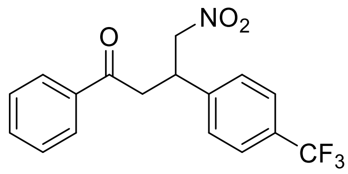

MW ($C_{17}H_{17}F_3NO_3$) = 337.30 g mol^{-1}

^{1}H NMR (400 MHz, CD_2Cl_2): δ = 7.94 – 7.90 (m, 2H), 7.64 – 7.58 (m, 3H), 7.47 (dd, *J* = 12.5, 5.0 Hz, 4H), 4.87 (dd, *J* = 12.8, 6.2 Hz, 1H), 4.72 (dd, *J* = 12.8, 8.6 Hz, 1H), 4.31 (dt, *J* = 13.5, 6.7 Hz, 1H), 3.55 – 3.40 (m, 2H).

^{13}C NMR (100 MHz, CD_2Cl_2): δ = 196.60, 144.07, 136.63, 134.06, 129.15, 128.59, 128.30, 126.22, 124.95, 79.60, 41.67, 39.41 ppm.

Yield: 96 % (1.43 mmol, 484 mg), white solid.

N-(4-(1-nitro-4-oxo-4-phenylbutan-2-yl)phenyl)acetamide

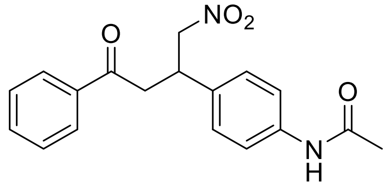

MW ($C_{18}H_{18}N_2O_4$) = 326.35 g mol^{-1}

^{1}H NMR (400 MHz, $CDCl_3$): δ = 7.91 (dd, *J* = 8.3, 1.0 Hz, 2H), 7.58 (t, *J* = 7.5 Hz, 1H), 7.49 – 7.43 (m, 4H), 7.24 (d, *J* = 8.5 Hz, 2H), 7.17 (s, 1H), 4.81 (dd, *J* = 12.5, 6.5 Hz, 1H), 4.66 (dd, *J* = 12.5, 8.1 Hz, 1H), 4.24 – 4.14 (m, 1H), 3.51 – 3.38 (m, 2H), 2.16 (s, 3H) ppm.

^{13}C NMR (100 MHz, $CDCl_3$): δ = 196.97, 168.47, 137.58, 136.44, 134.98, 133.76, 128.91, 128.23, 128.15, 120.54, 79.72, 41.63, 38.94, 24.71 ppm.

Yield: 71 % (1.06 mmol, 345 mg), yellow solid.

4-nitro-3-(4-nitrophenyl)-1-phenylbutan-1-one

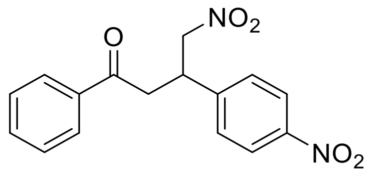

MW ($C_{16}H_{14}N_2O_5$) = 314.30 g mol^{-1}

^{1}H NMR (400 MHz, $CDCl_3$): δ = 8.21 (d, *J* = 8.8 Hz, 2H), 7.91 (dd, *J* = 8.4, 1.3 Hz, 2H), 7.63 – 7.57 (m, 1H), 7.51 – 7.45 (m, 4H), 4.88 (dd, *J* = 12.9, 6.3 Hz, 1H), 4.74 (dd, *J* = 12.9, 8.3 Hz, 1H), 4.43 – 4.32 (m, 1H), 3.50 (dd, *J* = 6.9, 1.4 Hz, 2H) ppm.

^{13}C NMR (100 MHz, $CDCl_3$): δ = 195.98, 146.65, 136.07, 134.10, 129.03, 128.78, 128.13, 124.42, 78.94, 41.20, 39.11 ppm.

Yield: 78 % (2.34 mmol, 735 mg), yellow solid.

3-(naphthalen-1-yl)-4-nitro-1-phenylbutan-1-one

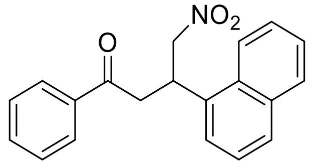

MW ($C_{20}H_{17}NO_3$) = 319.36 g mol^{-1}

^{1}H NMR (400 MHz, $CDCl_3$): δ = 8.23 (d, *J* = 8.6 Hz, 1H), 7.94 (dt, *J* = 8.5, 1.6 Hz, 2H), 7.91 – 7.87 (m, 1H), 7.79 (dd, *J* = 7.2, 2.1 Hz, 1H), 7.64 – 7.51 (m, 3H), 7.48 – 7.41 (m, 4H), 5.19 (p, *J* = 6.9 Hz, 1H), 4.98 – 4.85 (m, 2H), 3.66 – 3.57 (m, 2H) ppm.

^{3}C NMR (100 MHz, $CDCl_3$): δ = 196.98, 136.37, 135.15, 134.25, 133.65, 131.02, 129.31, 128.79, 128.19, 128.10, 127.01, 126.14, 125.33, 122.45, 78.87, 47.47, 41.49 ppm.

Yield: 97 % (1.48 mmol, 473 mg), brown oil.

4-nitro-1-phenyl-3-(thiophen-2-yl)butan-1-one

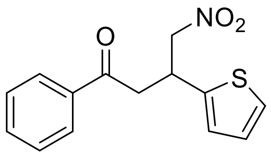

MW ($C_{14}H_{13}NO_3S$) = 275.32 g mol^{-1}

^{1}H NMR (400 MHz, $CDCl_3$): δ = 7.97 – 7.91 (m, 2H), 7.63 – 7.56 (m, 1H), 7.51 – 7.45 (m, 2H), 7.21 (dd, *J* = 5.0, 1.3 Hz, 1H), 6.99 – 6.92 (m, 2H), 4.85 (dd, *J* = 12.6, 6.3 Hz, 1H), 4.71 (dd, *J* = 12.6, 7.5 Hz, 1H), 4.61 – 4.51 (m, 1H), 3.59 – 3.43 (m, 2H) ppm.

^{3}C NMR (100 MHz, $CDCl_3$): δ = 196.57, 142.00, 136.37, 133.84, 128.92, 128.19, 127.29, 125.73, 124.86, 80.00, 42.44, 34.90 ppm.

Yield: 55 % (0.82 mmol, 226 mg), pink solid.

4-nitro-1,3,4-triphenylbutan-1-one

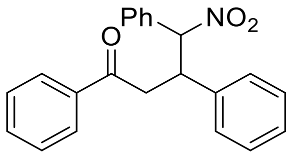

MW ($C_{22}H_{19}NO_3$) = 345.40 g mol^{-1}

^{1}H NMR (500 MHz, CD_2Cl_2): δ = 7.74 – 7.64 (m, 4H), 7.51 – 7.41 (m, 6H), 7.38 – 7.30 (m, 4H), 7.28 – 7.22 (m, 1H), 5.92 (d, *J* = 11.9 Hz, 1H), 4.60 – 4.51 (m, 1H), 3.35 (dd, *J* = 17.2, 10.1 Hz, 1H), 2.88 (dd, *J* = 17.2, 3.1 Hz, 1H) ppm.

^{13}C NMR (125.7 MHz, CD_2Cl_2): δ = 196.65, 139.51, 136.91, 133.59, 133.27, 130.85, 129.75, 129.07, 128.88, 128.83, 128.60, 128.12, 96.30, 45.14, 41.36 ppm.

Yield: 57 % (0.85 mmol, 296 mg), white solid.

4-(4-chlorophenyl)-4-nitro-1,3-diphenylbutan-1-one

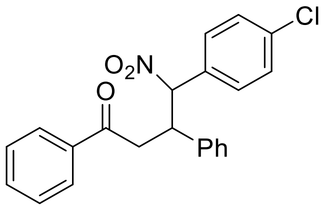

MW ($C_{22}H_{18}ClNO_3$) = 379.84 g mol^{-1}

^{1}H NMR (400 MHz, CD_2Cl_2): δ = 7.70 – 7.63 (m, 4H), 7.53 – 7.30 (m, 10H), 5.93 (d, *J* = 11.9 Hz, 1H), 4.55 – 4.47 (m, 1H), 3.34 (dd, *J* = 17.2, 9.8 Hz, 1H), 2.90 (dd, *J* = 17.2, 3.3 Hz, 1H) ppm.

^{13}C NMR (100 MHz, CD_2Cl_2): δ = 196.51, 139.21, 136.86, 136.84, 133.68, 131.74, 130.34, 129.93, 129.13, 128.91, 128.56, 128.23, 128.12, 95.43, 45.24, 41.25 ppm.

Yield: 23 % (0.35 mmol, 138 mg), white solid.

4-(4-fluorophenyl)-4-nitro-1,3-diphenylbutan-1-one

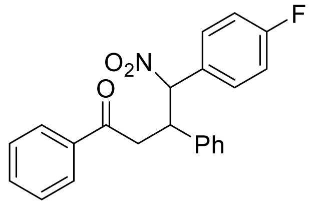

MW ($C_{22}H_{18}FNO_3$) = 363.39 g mol^{-1}

^{1}H NMR (400 MHz, $CDCl_3$): δ = 7.71 – 7.64 (m, 4H), 7.52 – 7.47 (m, 1H), 7.42 – 7.28 (m, 6H), 7.23 (ddd, *J* = 7.2, 3.8, 1.3 Hz, 1H), 7.16 – 7.09 (m, 2H), 5.94 (d, *J* = 11.9 Hz, 1H), 4.59 – 4.51 (m, 1H), 3.31 (dd, *J* = 17.1, 9.4 Hz, 1H), 2.91 (dd, *J* = 17.1, 3.4 Hz, 1H) ppm.

^{13}C NMR (100 MHz, $CDCl_3$): δ = 196.53, 138.71, 136.62, 133.49, 130.69, 130.60, 129.04, 128.69, 128.21, 128.07, 127.97, 116.70, 116.48, 94.98, 44.99, 41.18 ppm.

Yield: 48 % (0.63 mmol, 228 mg), white solid.

4-nitro-1,3-diphenyl-4-(4-(trifluoromethyl)phenyl)butan-1-one

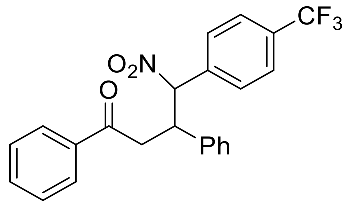

MW ($C_{23}H_{18}F_3NO_3$) = 413.40 g mol^{-1}

^{1}H NMR (400 MHz, CD_2Cl_2): δ = 7.81 (d, *J* = 8.1 Hz, 2H), 7.69 (d, *J* = 8.2 Hz, 2H), 7.63 (dd, *J* = 8.5, 1.1 Hz, 2H), 7.46 (t, *J* = 7.4 Hz, 1H), 7.42 – 7.37 (m, 2H), 7.31 (dd, *J* = 17.2, 7.9 Hz, 4H), 7.22 (t, *J* = 7.3 Hz, 1H), 5.99 (d, *J* = 11.8 Hz, 1H), 4.52 (dd, *J* = 17.0, 7.8 Hz, 1H), 3.32 (dd, *J* = 17.3, 9.6 Hz, 1H), 2.85 (dd, *J* = 17.3, 3.4 Hz, 1H) ppm.

^{13}C NMR (100 MHz, CD_2Cl_2): δ = 196.41, 139.02, 136.94, 136.79, 133.74, 129.54, 129.18, 128.92, 128.56, 128.33, 128.11, 126.67, 95.47, 45.30, 41.12 ppm.

Yield: 37 % (0.56 mmol, 230 mg), white solid.

9. Characterization of the isolated products

pentan-1-amonium chloride (Table 1, Entry 4)[8]

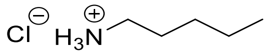

FW ($C_5H_{14}ClN$) = 123.62 g mol^{-1}

^{1}H NMR (300 MHz, DMSO-D$_6$): δ = 8.15 (s, 3H), 2.76 – 2.66 (m, 2H), 1.63 – 1.49 (m, 2H), 1.27 (dq, *J* = 7.1, 3.5 Hz, 4H), 0.92 – 0.79 (m, 3H) ppm.

^{13}C NMR (75 MHz, DMSO-D$_6$): δ = 38.62, 28.00, 26.54, 21.63, 13.73 ppm.

Yield: 99 % (0.495 mmol, 61 mg), white solid.

3-phenyl-5-(*p*-tolyl)-3,4-dihydro-2*H*-pyrrole (**1**)[9]

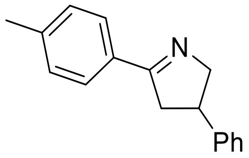

MW ($C_{17}H_{17}N$) = 235.33 g mol^{-1}

^{1}H NMR (400 MHz, CD_2Cl_2): δ = 7.76 (d, *J* = 8.2 Hz, 2H), 7.32 – 7.26 (m, 2H), 7.26 – 7.17 (m, 5H), 4.46 (dd, *J* = 16.1, 8.7 Hz, 1H), 4.01 (dd, *J* = 16.3, 6.2 Hz, 1H), 3.64 (ddd, *J* = 15.2, 8.9, 6.4 Hz, 1H), 3.50 – 3.38 (m, 1H), 3.04 (ddt, *J* = 17.1, 6.7, 2.0 Hz, 1H), 2.39 (s, 3H) ppm.

^{13}C NMR (100 MHz, CD_2Cl_2): δ = 172.16, 145.79, 141.10, 132.30, 129.48, 128.98, 127.93, 127.21, 126.62, 69.96, 44.21, 43.43, 21.56 ppm.

Yield: 89 % (0.178 mmol, 42 mg), white solid.

3-phenyl-5-(*m*-tolyl)-3,4-dihydro-2*H*-pyrrole (**2**)

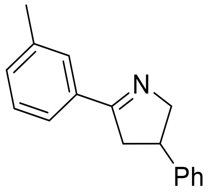

MW ($C_{17}H_{17}N$) = 235.33 g mol^{-1}

^{1}H NMR (400 MHz, $CDCl_3$): δ = 7.75 (s, 1H), 7.63 (d, *J* = 7.6 Hz, 1H), 7.34 – 7.27 (m, 4H), 7.24 – 7.20 (m, 3H), 4.58 – 4.47 (m, 1H), 4.11 (ddt, *J* = 16.4, 5.9, 2.0 Hz, 1H), 3.66 (tt, *J* = 8.6, 6.2 Hz, 1H), 3.53 – 3.42 (m, 1H), 3.09 (ddt, *J* = 17.1, 6.5, 2.0 Hz, 1H), 2.40 (s, 3H).

^{13}C NMR (100 MHz, $CDCl_3$): δ = 172.69, 145.23, 138.33, 134.37, 131.46, 128.85, 128.52, 128.29, 126.93, 126.53, 125.01, 69.68, 44.17, 43.04, 21.49 ppm.

HRMS (ESI+) *m/z* calcd. for [$C_{17}H_{17}N+H^+$] 236.14338, found 236.14274.

Yield: 85 % (0.170mmol, 40 mg), white solid.

3-phenyl-5-(*o*-tolyl)-3,4-dihydro-2*H*-pyrrole (**3**)

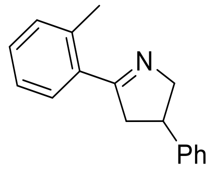

MW ($C_{17}H_{17}N$) = 235.33 g mol^{-1}

^{1}H NMR (400 MHz, CD_2Cl_2): δ = 7.48 (d, *J* = 7.5 Hz, 1H), 7.34 – 7.21 (m, 8H), 4.56 – 4.47 (m, 1H), 4.13 – 4.05 (m, 1H), 3.67 – 3.58 (m, 1H), 3.48 – 3.39 (m, 1H), 3.10 – 3.01 (m, 1H), 2.56 (s, 3H).

^{13}C NMR (100 MHz, CD_2Cl_2): δ = 174.05, 145.64, 137.94, 131.66, 129.42, 129.33, 128.99, 127.19, 126.63, 125.91, 70.33, 47.45, 43.24, 22.06 ppm.

HRMS (ESI+) *m/z* calcd. for [$C_{17}H_{17}N+H^+$] 236.14338, found 236.14288.

Yield: 79 % (0.158 mmol, 37 mg), white solid.

5-(4-chlorophenyl)-3-phenyl-3,4-dihydro-2*H*-pyrrole (**4**)[10]

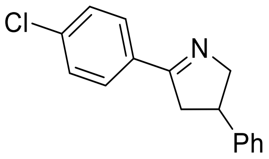

MW ($C_{16}H_{14}NCl$) = 255.75 g mol^{-1}

^{1}H NMR (400 MHz, CD_2Cl_2): δ = 7.82 (d, *J* = 8.6 Hz, 2H), 7.41 (d, *J* = 8.6 Hz, 2H), 7.33 – 7.26 (m, 2H), 7.24 – 7.17 (m, 3H), 4.48 (dd, *J* = 16.8, 8.5 Hz, 1H), 4.03 (d, *J* = 16.6 Hz, 1H), 3.72 – 3.61 (m, 1H), 3.44 (dd, *J* = 17.1, 9.5 Hz, 1H), 3.03 (dd, *J* = 17.2, 6.7 Hz, 1H) ppm.

^{13}C NMR (100 MHz, CD_2Cl_2): δ = 171.29, 145.47, 136.61, 133.56, 129.38, 129.03, 129.00, 127.18, 126.73, 70.08, 44.18, 43.49. ppm.

Yield: 80 % (0.160 mmol, 41 mg), white solid.

5-(3-chlorophenyl)-3-phenyl-3,4-dihydro-2*H*-pyrrole (**5**)

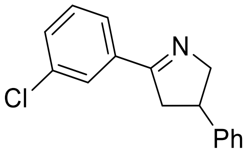

MW ($C_{16}H_{14}NCl$) = 255.75 g mol^{-1}

^{1}H NMR (400 MHz, $CDCl_3$): δ = 7.87 (t, *J* = 1.8 Hz, 1H), 7.72 (dt, *J* = 7.6, 1.4 Hz, 1H), 7.44 – 7.27 (m, 5H), 7.22 – 7.18 (m, 2H), 4.57 – 4.47 (m, 1H), 4.12 (ddt, *J* = 16.6, 6.0, 2.1 Hz, 1H), 3.67 (tt, *J* = 8.9, 6.3 Hz, 1H), 3.48 – 3.38 (m, 1H), 3.09 – 3.00 (m, 1H).

^{13}C NMR (100 MHz, $CDCl_3$): δ = 171.34, 144.85, 136.19, 134.75, 130.59, 129.90, 128.85, 127.86, 126.87, 126.61, 125.85, 69.71, 44.03, 42.99 ppm.

HRMS (ESI+) *m/z* calcd. for [$C_{16}H_{14}NCl+H^+$] 256.08875, found 256.08820.

Yield: 78 % (0.156 mmol, 40 mg), white solid.

5-(4-bromophenyl)-3-phenyl-3,4-dihydro-2*H*-pyrrole (**6**)

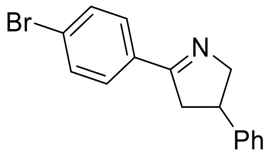

MW ($C_{16}H_{14}NBr$) = 300.20 g mol^{-1}

^{1}H NMR (400 MHz, CD_2Cl_2): δ = 7.76 (d, *J* = 8.5 Hz, 2H), 7.60 – 7.54 (m, 2H), 7.33 – 7.26 (m, 2H), 7.21 (d, *J* = 7.0 Hz, 3H), 4.53 – 4.43 (m, 1H), 4.03 (ddt, *J* = 16.6, 5.9, 2.0 Hz, 1H), 3.71 – 3.62 (m, 1H), 3.49 – 3.39 (m, 1H), 3.03 (dd, *J* = 17.0, 6.8 Hz, 1H).

^{13}C NMR (100 MHz, CD_2Cl_2): δ = 171.40, 145.44, 131.98, 129.61, 129.03, 127.18, 126.73, 70.10, 44.13, 43.48 ppm.

HRMS (ESI+) *m/z* calcd. for [$C_{16}H_{14}NBr+H^+$] 300.03824, found 300.03780.

Yield: 65 % (0.130 mmol, 39 mg), white solid.

5-(4-fluorophenyl)-3-phenyl-3,4-dihydro-2*H*-pyrrole (**7**)

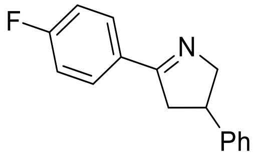

MW ($C_{16}H_{14}NF$) = 239.29 g mol^{-1}

^{1}H NMR (500 MHz, $CDCl_3$): δ = 7.86 (dd, *J* = 8.8, 5.5 Hz, 2H), 7.31 (dd, *J* = 10.2, 5.0 Hz, 2H), 7.23 (dd, *J* = 10.4, 4.6 Hz, 3H), 7.13 – 7.09 (m, 2H), 4.51 (dd, *J* = 16.4, 8.6 Hz, 1H), 4.14 – 4.06 (m, 1H), 3.67 (tt, *J* = 8.8, 6.4 Hz, 1H), 3.49 – 3.40 (m, 1H), 3.06 (ddt, *J* = 17.0, 6.6, 2.0 Hz, 1H) ppm.

^{13}C NMR (125.7 MHz, $CDCl_3$): δ = 171.35, 165.36, 163.37, 145.01, 129.84, 129.77, 128.90, 126.93, 126.63, 115.77, 115.59, 69.71, 44.18, 43.17 ppm.

HRMS (ESI+) *m/z* calcd. for [$C_{16}H_{14}NF+H^+$] 240.11830, found 240.11766.

Yield: 90 % (0.180 mmol, 43 mg), white solid.

5-(4-methoxyphenyl)-3-phenyl-3,4-dihydro-2*H*-pyrrole (**8**)[10]

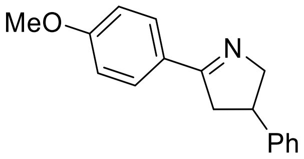

MW ($C_{17}H_{17}NO$) = 251.33 g mol^{-1}

^{1}H NMR (400 MHz, CD_2Cl_2): δ = 7.82 (d, *J* = 8.8 Hz, 2H), 7.29 (t, *J* = 7.4 Hz, 2H), 7.20 (dd, *J* = 12.5, 6.9 Hz, 3H), 6.94 (d, *J* = 8.8 Hz, 2H), 4.44 (dd, *J* = 16.2, 8.4 Hz, 1H), 4.00 (d, *J* = 22.1 Hz, 1H), 3.84 (s, 3H), 3.69 – 3.58 (m, 1H), 3.43 (dd, *J* = 16.3, 10.1 Hz, 1H), 3.03 (dd, *J* = 16.9, 6.7 Hz, 1H) ppm.

^{13}C NMR (100 MHz, CD_2Cl_2): δ = 171.58, 161.85, 145.83, 129.57, 128.99, 127.84, 127.22, 126.61, 114.08, 69.88, 55.73, 44.17, 43.53 ppm.

Yield: 94 % (0.187 mmol, 47 mg), white solid.

4-(3-phenyl-3,4-dihydro-2*H*-pyrrol-5-yl)phenol (**9**)

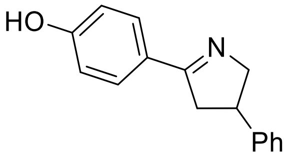

MW ($C_{16}H_{15}ON$) = 237.30 g mol^{-1}

^{1}H NMR (500 MHz, $CDCl_3$): δ = 13.75 (s, 1H), 7.39 – 7.36 (m, 1H), 7.35 – 7.30 (m, 3H), 7.23 (dt, *J* = 15.1, 4.4 Hz, 3H), 7.04 (dd, *J* = 8.3, 0.9 Hz, 1H), 6.87 (td, *J* = 7.6, 1.1 Hz, 1H), 4.55 (ddt, *J* = 16.0, 8.5, 1.5 Hz, 1H), 4.15 (ddt, *J* = 16.0, 5.8, 1.8 Hz, 1H), 3.65 (tt, *J* = 8.5, 6.1 Hz, 1H), 3.59 – 3.52 (m, 1H), 3.17 (ddt, *J* = 16.9, 6.1, 1.7 Hz, 1H) ppm.

^{13}C NMR (125.7 MHz, $CDCl_3$): δ = 176.27, 161.18, 144.47, 132.68, 129.41, 129.00, 126.94, 126.85, 118.47, 117.39, 67.97, 43.88, 41.44 ppm.

HRMS (ESI+) *m/z* calcd. for [$C_{16}H_{15}ON+H^+$] 238.12264, found 238.12213.

Yield: 95 % (0.190 mmol, 45 mg), white solid.

2-(3-phenyl-3,4-dihydro-2*H*-pyrrol-5-yl)phenol (**10**)[11]

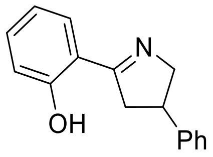

MW ($C_{16}H_{15}ON$) = 237.30 g mol^{-1}

^{1}H NMR (500 MHz, $CDCl_3$): δ = 13.75 (s, 1H), 7.39 – 7.30 (m, 4H), 7.22 (dd, *J* = 9.0, 7.7 Hz, 3H), 7.03 (dd, *J* = 8.3, 0.8 Hz, 1H), 6.87 (td, *J* = 7.7, 1.0 Hz, 1H), 4.55 (dd, *J* = 16.0, 8.5 Hz, 1H), 4.15 (ddt, *J* = 16.0, 5.9, 1.7 Hz, 1H), 3.65 (tt, *J* = 8.5, 6.1 Hz, 1H), 3.59 – 3.52 (m, 1H), 3.17 (ddt, *J* = 16.9, 6.1, 1.7 Hz, 1H) ppm.

^{13}C NMR (125.7 MHz, CD_2Cl_2): δ = 176.28, 161.19, 144.48, 132.69, 129.41, 129.01, 126.95, 126.87, 118.48, 117.40, 117.06, 67.98, 43.90, 41.45 ppm.

Yield: 91 % (0.181 mmol, 43 mg), white solid.

3-phenyl-5-(4-(trifluoromethyl)phenyl)-3,4-dihydro-2*H*-pyrrole (**11**)

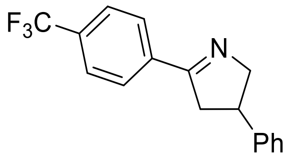

MW ($C_{17}H_{14}NF_3$) = 289.30 g mol^{-1}

^{1}H NMR (400 MHz, $CDCl_3$): δ = 7.98 (d, *J* = 8.5 Hz, 2H), 7.69 (d, *J* = 8.6 Hz, 2H), 7.35 – 7.29 (m, 2H), 7.23 – 7.19 (m, 3H), 4.56 (dd, *J* = 16.8, 8.6 Hz, 1H), 4.20 – 4.10 (m, 1H), 3.70 (tt, *J* = 8.7, 6.4 Hz, 1H), 3.55 – 3.43 (m, 1H), 3.10 (ddt, *J* = 17.1, 6.6, 2.1 Hz, 1H).

^{13}C NMR (100 MHz, CD_2Cl_2): δ = 171.40, 144.74, 137.59, 132.43, 128.94, 128.06, 126.88, 126.72, 125.63, 69.91, 44.19, 43.07 ppm.

HRMS (ESI+) *m/z* calcd. for [$C_{17}H_{14}NF_3+H^+$] 290.11511, found 290.11434.

Yield: 79 % (0.158 mmol, 46 mg), white solid.

5-phenyl-3-(*p*-tolyl)-3,4-dihydro-2*H*-pyrrole (**12**)

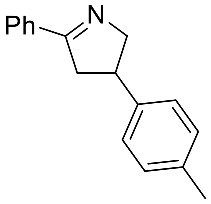

MW ($C_{17}H_{17}N$) = 235.33 g mol^{-1}

^{1}H NMR (400 MHz, CD_2Cl_2): δ = 8.36 (dd, *J* = 7.8, 1.7 Hz, 2H), 7.45 (dd, *J* = 5.9, 4.8 Hz, 3H), 7.22 – 7.15 (m, 4H), 4.50 (dd, *J* = 13.9, 9.4 Hz, 1H), 4.26 (dd, *J* = 14.0, 7.6 Hz, 1H), 3.81 – 3.70 (m, 1H), 3.62 (dd, *J* = 16.4, 9.0 Hz, 1H), 3.21 (dd, *J* = 16.8, 6.2 Hz, 1H), 2.33 (s, 3H) ppm.

^{13}C NMR (100 MHz, CD_2Cl_2): δ = 165.91, 139.04, 136.92, 129.96, 129.51, 128.29, 126.94, 126.53, 71.49, 39.48, 35.69, 20.64 ppm.

HRMS (ESI+) *m/z* calcd. for [$C_{17}H_{17}N+H^{+}$] 236.14338, found 236.14278.

Yield: 87 % (0.170 mmol, 41 mg), white solid.

5-phenyl-3-(*m*-tolyl)-3,4-dihydro-2*H*-pyrrole (**13**)

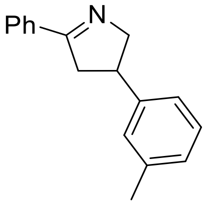

MW ($C_{17}H_{17}N$) = 235.33 g mol^{-1}

^{1}H NMR (500 MHz, $CDCl_3$): δ = 7.90 – 7.85 (m, 2H), 7.48 – 7.40 (m, 4H), 7.20 (t, *J* = 7.7 Hz, 1H), 7.05 – 7.02 (m, 2H), 4.55 – 4.48 (m, 1H), 4.11 (ddt, *J* = 16.4, 6.1, 2.0 Hz, 1H), 3.63 (tt, *J* = 8.7, 6.4 Hz, 1H), 3.50 – 3.43 (m, 1H), 3.09 (ddt, *J* = 17.1, 6.6, 2.0 Hz, 1H), 2.33 (s, 3H).

^{13}C NMR (125.7 MHz, $CDCl_3$): δ = 172.57, 145.13, 138.52, 134.47, 130.68, 128.77, 128.65, 127.79, 127.71, 127.31, 123.98, 69.77, 44.17, 42.96, 25.46 ppm.

HRMS (ESI+) *m/z* calcd. for [$C_{17}H_{17}N+H^+$] 236.14338, found 236.14282.

Yield: 93 % (0.187 mmol, 44 mg), white solid.

5-phenyl-3-(*o*-tolyl)-3,4-dihydro-2*H*-pyrrole (**14**)

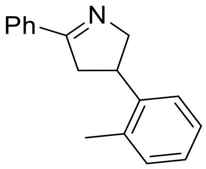

MW ($C_{17}H_{17}N$) = 235.33 g mol^{-1}

^{1}H NMR (500 MHz, $CDCl_3$): δ = 7.87 (dd, *J* = 7.7, 1.8 Hz, 2H), 7.48 – 7.39 (m, 4H), 7.16 (dd, *J* = 10.2, 5.4 Hz, 3H), 4.50 (dd, *J* = 16.4, 8.5 Hz, 1H), 4.13 (ddt, *J* = 16.4, 5.2, 1.8 Hz, 1H), 3.93 – 3.86 (m, 1H), 3.49 – 3.40 (m, 1H), 3.11 – 3.03 (m, 1H), 2.39 (s, 3H) ppm.

^{13}C NMR (125.7 MHz, $CDCl_3$): δ = 172.52, 143.24, 135.50, 134.47, 130.69, 130.49, 128.65, 127.79, 126.75, 126.37, 125.48, 68.78, 43.39, 38.46, 25.45 ppm.

HRMS (ESI+) *m/z* calcd. for [$C_{17}H_{17}N+H^+$] 236.14338, found 236.14287.

Yield: 85 % (0.170 mmol, 40 mg), white solid.

3-(4-chlorophenyl)-5-phenyl-3,4-dihydro-2*H*-pyrrole (**15**)[10]

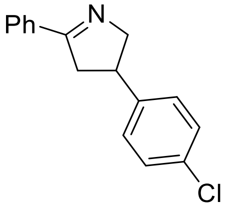

MW ($C_{16}H_{14}NCl$) = 255.75 g mol^{-1}

^{1}H NMR (400 MHz, CD_2Cl_2): δ = 7.89 – 7.84 (m, 2H), 7.44 (t, *J* = 6.2 Hz, 3H), 7.30 – 7.25 (m, 2H), 7.17 (d, *J* = 8.5 Hz, 2H), 4.48 (dd, *J* = 16.3, 8.6 Hz, 1H), 4.06 – 3.97 (m, 1H), 3.70 – 3.60 (m, 1H), 3.52 – 3.41 (m, 1H), 3.08 – 2.98 (m, 1H).

^{13}C NMR (100 MHz, CD_2Cl_2): δ = 172.20, 144.36, 134.85, 132.18, 130.82, 129.03, 128.82, 128.68, 127.99, 69.90, 44.19, 42.81 ppm.

Yield: 82 % (0.164 mmol, 42 mg), white solid.

3-(3-chlorophenyl)-5-phenyl-3,4-dihydro-2*H*-pyrrole (**16**)

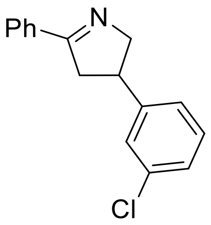

MW ($C_{16}H_{14}NCl$) = 255.75 g mol^{-1}

^{1}H NMR (500 MHz, $CDCl_3$): δ = 7.91 – 7.85 (m, 2H), 7.44 (ddd, *J* = 7.7, 4.5, 1.4 Hz, 3H), 7.32 – 7.28 (m, 2H), 7.22 (d, *J* = 6.9 Hz, 2H), 4.57 – 4.48 (m, 1H), 4.11 (ddt, *J* = 16.4, 6.1, 2.0 Hz, 1H), 3.67 (tt, *J* = 8.8, 6.4 Hz, 1H), 3.53 – 3.43 (m, 1H), 3.09 (ddt, *J* = 17.1, 6.6, 2.0 Hz, 1H).

^{13}C NMR (125.7 MHz, $CDCl_3$): δ = 172.53, 145.16, 134.44, 130.69, 128.86, 128.65, 127.77, 126.94, 126.56, 69.75, 44.15, 43.05 ppm.

HRMS (ESI+) *m/z* calcd. for [$C_{16}H_{14}NCl+H^+$] 256.08875, found 256.28838.

Yield: 86 % (0.172 mmol, 44 mg), white solid.

3-(2-chlorophenyl)-5-phenyl-3,4-dihydro-2*H*-pyrrole (**17**)

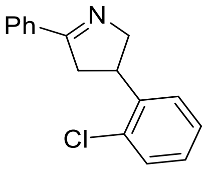

MW ($C_{16}H_{14}NCl$) = 255.75 g mol^{-1}

^{1}H NMR (500 MHz, $CDCl_3$): δ = 7.87 (dt, *J* = 3.8, 2.3 Hz, 2H), 7.47 – 7.41 (m, 4H), 7.38 (d, *J* = 7.6 Hz, 1H), 7.22 – 7.19 (m, 2H), 4.56 – 4.47 (m, 1H), 4.20 – 4.12 (m, 2H), 3.53 – 3.45 (m, 1H), 3.14 – 3.06 (m, 1H) ppm.

^{13}C NMR (100 MHz, CD_2Cl_2): δ = 172.39, 142.25, 134.34, 133.71, 130.78, 129.81, 128.68, 127.81, 127.46, 127.38, 68.08, 42.98, 25.47 ppm.

HRMS (ESI+) *m/z* calcd. for [$C_{16}H_{14}NCl+H^+$] 256.08875, found 256.08841.

Yield: 88 % (0.164 mmol, 42 mg), white solid.

3-(4-bromophenyl)-5-phenyl-3,4-dihydro-2*H*-pyrrole (**18**)

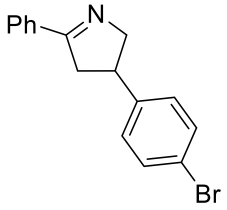

MW ($C_{16}H_{14}NBr$) = 300.20 g mol^{-1}

^{1}H NMR (400 MHz, CD_2Cl_2): δ = 7.86 (dd, *J* = 7.5, 1.8 Hz, 2H), 7.43 (dd, *J* = 7.9, 3.4 Hz, 5H), 7.11 (d, *J* = 8.5 Hz, 2H), 4.52 – 4.43 (m, 1H), 4.06 – 3.97 (m, 1H), 3.66 – 3.59 (m, 1H), 3.52 – 3.41 (m, 1H), 3.03 (dd, *J* = 17.1, 6.5 Hz, 1H) ppm.

^{13}C NMR (100 MHz, CD_2Cl_2): δ = 172.20, 144.86, 134.82, 132.00, 130.83, 129.08, 128.82, 127.99, 120.20, 69.84, 44.15, 42.85 ppm.

HRMS (ESI+) *m/z* calcd. for [$C_{16}H_{14}NBr+H^+$] 300.03824, found 300.03780.

Yield: 73 % (0.150 mmol, 44 mg), white solid.

3-(4-fluorophenyl)-5-phenyl-3,4-dihydro-2*H*-pyrrole (**19**)[12]

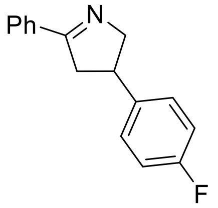

MW ($C_{16}H_{14}NF$) = 239.29 g mol^{-1}

^{1}H NMR (400 MHz, $CDCl_3$): δ = 7.89 – 7.84 (m, 2H), 7.47 – 7.42 (m, 3H), 7.20 – 7.14 (m, 2H), 7.02 – 6.95 (m, 2H), 4.51 (ddt, *J* = 16.4, 8.4, 1.7 Hz, 1H), 4.08 (ddt, *J* = 16.4,

5.9, 2.0 Hz, 1H), 3.71 – 3.61 (m, 1H), 3.52 – 3.42 (m, 1H), 3.05 (ddt, *J* = 17.0, 6.4, 2.0 Hz, 1H) ppm.

^{13}C NMR (100 MHz, $CDCl_3$): δ = 172.46, 162.84, 140.91, 134.36, 130.77, 128.68, 128.37, 128.30, 127.77, 115.72, 115.51, 69.79, 44.27, 42.36 ppm.

Yield: 86 % (0.171 mmol, 41 mg), white solid.

3-(4-methoxyphenyl)-5-phenyl-3,4-dihydro-2*H*-pyrrole (**20**)[10]

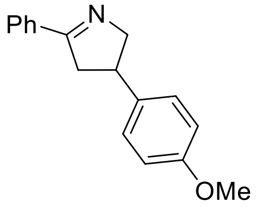

MW ($C_{17}H_{17}NO$) = 251.33 g mol^{-1}

^{1}H NMR (400 MHz, CD_2Cl_2): δ = 7.86 (dd, *J* = 7.4, 2.0 Hz, 2H), 7.43 (d, *J* = 7.2 Hz, 3H), 7.14 (d, *J* = 8.7 Hz, 2H), 6.83 (d, *J* = 8.7 Hz, 2H), 4.45 (dd, *J* = 16.2, 8.4 Hz, 1H), 3.99 (dd, *J* = 15.7, 6.8 Hz, 1H), 3.76 (s, 3H), 3.66 – 3.56 (m, 1H), 3.43 (dd, *J* = 16.9, 9.5 Hz, 1H), 3.01 (dd, *J* = 16.9, 6.8 Hz, 1H) ppm.

^{13}C NMR (100 MHz, CD_2Cl_2): δ = 172.37, 158.58, 137.59, 130.69, 128.78, 128.12, 127.96, 126.17, 114.31, 70.07, 55.59, 44.28, 42.67 ppm.

Yield: 86 % (0.171 mmol, 43 mg), white solid.

3-(4-(benzyloxy)phenyl)-5-phenyl-3,4-dihydro-2*H*-pyrrole (**21**)

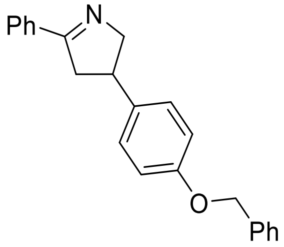

MW ($C_{23}H_{21}ON$) = 327.43 g mol^{-1}

^{1}H NMR (400 MHz, $CDCl_3$): δ = 7.87 (dd, *J* = 7.5, 2.0 Hz, 2H), 7.47 – 7.29 (m, 8H), 7.14 (d, *J* = 8.6 Hz, 2H), 6.92 (d, *J* = 8.7 Hz, 2H), 5.05 (s, 2H), 4.50 (dd, *J* = 16.3, 8.5 Hz, 1H), 4.07 (dd, *J* = 16.4, 6.0 Hz, 1H), 3.63 (dt, *J* = 15.1, 7.7 Hz, 1H), 3.45 (dd, *J* = 16.8, 9.7 Hz, 1H), 3.05 (dd, *J* = 17.1, 6.6 Hz, 1H) ppm.

^{13}C NMR (100 MHz, $CDCl_3$): δ = 172.61, 157.52, 137.48, 137.19, 134.50, 130.67, 128.71, 128.64, 128.08, 127.92, 127.76, 127.59, 115.19, 70.21, 69.78, 44.22, 42.34 ppm.

HRMS (ESI+) *m/z* calcd. for [$C_{23}H_{21}ON+H^+$] 328.16959, found 328.16883.

Yield: 74 % (0.150 mmol, 48 mg), white solid.

3-(4-(methylthio)phenyl)-5-phenyl-3,4-dihydro-2*H*-pyrrole (**22**)

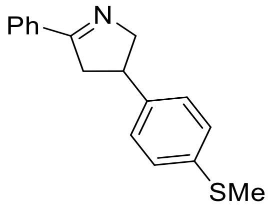

MW ($C_{17}H_{17}NS$) = 267.39 g mol^{-1}

^{1}H NMR (400 MHz, $CDCl_3$): δ = 7.86 (dd, *J* = 7.6, 2.1 Hz, 2H), 7.47 – 7.40 (m, 3H), 7.23 – 7.19 (m, 2H), 7.17 – 7.12 (m, 2H), 4.51 (dd, *J* = 16.4, 8.3 Hz, 1H), 4.08 (ddt, *J* = 16.6, 6.0, 2.0 Hz, 1H), 3.68 – 3.59 (m, 1H), 3.46 (dd, *J* = 17.0, 9.7 Hz, 1H), 3.06 (ddt, *J* = 10.6, 6.5, 1.7 Hz, 1H), 2.47 (s, 3H) ppm.

^{13}C NMR (100 MHz, $CDCl_3$): δ = 172.50, 142.24, 136.33, 134.42, 130.73, 128.67, 127.78, 127.49, 127.45, 69.71, 44.14, 42.61, 16.35 ppm.

HRMS (ESI+) *m/z* calcd. for [$C_{17}H_{17}NS+H^+$] 268.11545, found 268.11491.

Yield: 77 % (0.153 mmol, 41 mg), white solid.

3-(4-(*tert*-butyl)phenyl)-5-phenyl-3,4-dihydro-2*H*-pyrrole (**23**)

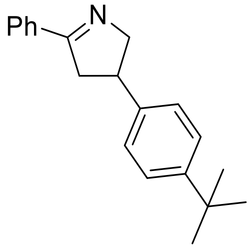

MW ($C_{20}H_{23}N$) = 277.41 g mol^{-1}

^{1}H NMR (400 MHz, $CDCl_3$): δ = 7.87 (dd, *J* = 7.6, 2.1 Hz, 2H), 7.44 (ddd, *J* = 5.2, 4.5, 1.9 Hz, 3H), 7.36 – 7.30 (m, 2H), 7.19 – 7.14 (m, 2H), 4.55 – 4.47 (m, 1H), 4.11 (ddt, *J* = 16.2, 6.1, 2.0 Hz, 1H), 3.65 (tt, *J* = 8.7, 6.6 Hz, 1H), 3.50 – 3.41 (m, 1H), 3.09 (ddt, *J* = 17.0, 6.7, 2.0 Hz, 1H), 1.31 (s, 9H) ppm.

^{13}C NMR (100 MHz, $CDCl_3$): δ = 172.58, 149.44, 141.95, 134.55, 130.64, 128.64, 127.76, 126.66, 125.73, 69.69, 44.09, 42.67, 34.55, 31.51 ppm.

HRMS (ESI+) *m/z* calcd. for [$C_{20}H_{23}N$+H^+] 278.19033, found 278.18975.

Yield: 92 % (0.184 mmol, 51 mg), white solid.

5-phenyl-3-(4-(trifluoromethyl)phenyl)-3,4-dihydro-2*H*-pyrrole (**24**)

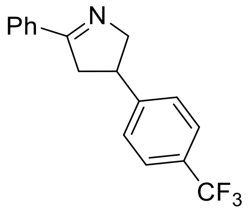

MW ($C_{17}H_{14}NF_3$) = 289.30 g mol^{-1}

^{1}H NMR (400 MHz, $CDCl_3$): δ = 7.87 (dd, *J* = 7.6, 1.7 Hz, 2H), 7.56 (d, *J* = 8.2 Hz, 2H), 7.45 (dd, *J* = 7.3, 5.4 Hz, 3H), 7.33 (d, *J* = 8.5 Hz, 2H), 4.55 (dd, *J* = 16.3, 8.7 Hz, 1H), 4.13 (dd, *J* = 16.7, 5.6 Hz, 1H), 3.77 – 3.69 (m, 1H), 3.52 (dd, *J* = 17.0, 9.7 Hz, 1H), 3.09 (dd, *J* = 17.1, 6.3 Hz, 1H) ppm.

^{13}C NMR (100 MHz, $CDCl_3$): δ = 172.31, 134.18, 130.89, 128.73, 127.80, 127.40, 127.29, 125.87, 125.83, 69.59, 44.13, 42.88 ppm.

HRMS (ESI+) *m/z* calcd. for [$C_{17}H_{14}NF_3+H^+$] 290.11511, found 290.11450.

Yield: 75 % (0.150 mmol, 44 mg), white solid.

N-(4-(5-phenyl-3,4-dihydro-2*H*-pyrrol-3-yl)phenyl)acetamide (**25**)

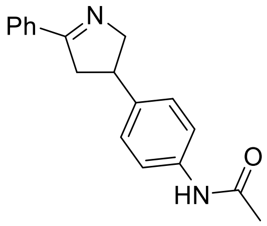

MW ($C_{18}H_{18}ON_2$) = 278.36 g mol^{-1}

^{1}H NMR (400 MHz, CD_2Cl_2): δ = 7.87 (d, *J* = 7.4 Hz, 2H), 7.47 (s, 1H), 7.45 – 7.40 (m, 5H), 7.17 (d, *J* = 7.8 Hz, 2H), 4.46 (dd, *J* = 16.5, 8.4 Hz, 1H), 4.01 (dd, *J* = 15.4, 7.0 Hz, 1H), 3.69 – 3.58 (m, 1H), 3.45 (dd, *J* = 16.0, 10.1 Hz, 1H), 3.03 (dd, *J* = 17.1, 6.8 Hz, 1H), 2.11 (s, 3H) ppm.

^{13}C NMR (100 MHz, CD_2Cl_2): δ = 172.42, 168.56, 141.27, 136.99, 134.93, 130.75, 128.80, 127.97, 127.58, 120.45, 69.90, 44.20, 42.87, 24.62 ppm.

HRMS (ESI+) *m/z* calcd. for [$C_{18}H_{18}ON_2+H^+$] 279.14919, found 279.14859.

Yield: 90 % (0.180 mmol, 50 mg), yellow solid.

4-(5-phenyl-3,4-dihydro-2*H*-pyrrol-3-yl)aniline (**26**)

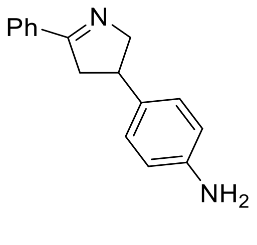

MW ($C_{16}H_{16}N_2$) = 236.32 g mol^{-1}

^{1}H NMR (500 MHz, $CDCl_3$): δ = 7.86 (dd, *J* = 7.6, 1.9 Hz, 2H), 7.45 – 7.41 (m, 3H), 7.01 (d, *J* = 8.4 Hz, 2H), 6.64 (d, *J* = 8.4 Hz, 2H), 4.47 (dd, *J* = 16.3, 8.5 Hz, 1H), 4.04 (ddt, *J* = 16.3, 6.1, 2.0 Hz, 1H), 3.59 (s, 2H), 3.46 – 3.39 (m, 1H), 3.02 (ddt, *J* = 17.0, 6.7, 2.0 Hz, 1H) ppm.

^{13}C NMR (125.7 MHz, $CDCl_3$): δ = 172.59, 144.74, 134.47, 130.49, 128.51, 127.68, 127.63, 115.42, 69.68, 44.07, 42.25 ppm.

HRMS (ESI+) *m/z* calcd. for [$C_{16}H_{16}N_2+H^+$] 237.13863, found 237.13815.

Yield: 85 % (0.170 mmol, 40 mg), white solid.

3-(naphthalen-1-yl)-5-phenyl-3,4-dihydro-2*H*-pyrrole (**27**)

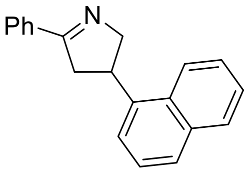

MW ($C_{20}H_{17}N$) = 271.36 g mol^{-1}

^{1}H NMR (400 MHz, $CDCl_3$): δ = 8.08 (d, *J* = 8.3 Hz, 1H), 7.94 – 7.85 (m, 3H), 7.74 (d, *J* = 7.9 Hz, 1H), 7.58 – 7.48 (m, 2H), 7.48 – 7.35 (m, 5H), 4.64 (ddt, *J* = 16.0, 8.0, 1.8 Hz, 1H), 4.51 – 4.41 (m, 1H), 4.40 – 4.31 (m, 1H), 3.59 (ddt, *J* = 17.1, 9.6, 1.9 Hz, 1H), 3.34 – 3.25 (m, 1H) ppm.

^{3}C NMR (100 MHz, $CDCl_3$): δ = 172.70, 140.37, 134.51, 134.24, 131.57, 130.76, 129.25, 128.70, 127.84, 127.27, 126.25, 125.80, 125.77, 123.43, 123.09, 68.42, 43.36, 38.64 ppm.

HRMS (ESI+) *m/z* calcd. for [$C_{20}H_{17}N+H^+$] 272.14338, found 272.14282.

Yield: 77 % (0.155 mmol, 42 mg), white solid.

5-phenyl-3-(thiophen-2-yl)-3,4-dihydro-2*H*-pyrrole (**28**)

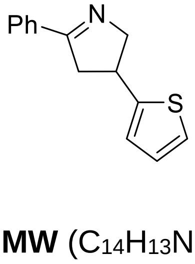

MW ($C_{14}H_{13}NS$) = 227.33 g mol^{-1}

^{1}**H NMR** (400 MHz, $CDCl_3$): δ = 7.88 – 7.83 (m, 2H), 7.47 – 7.40 (m, 3H), 7.15 (dd, *J* = 5.1, 1.2 Hz, 1H), 6.94 (dd, *J* = 5.1, 3.5 Hz, 1H), 6.89 – 6.85 (m, 1H), 4.58 – 4.48 (m, 1H), 4.14 (ddt, *J* = 16.1, 6.5, 2.0 Hz, 1H), 4.00 – 3.90 (m, 1H), 3.50 (dddd, *J* = 16.9, 9.4, 2.0, 1.2 Hz, 1H), 3.14 (ddt, *J* = 16.9, 7.1, 2.0 Hz, 1H) ppm.

^{3}**C NMR** (100 MHz, $CDCl_3$): δ = 172.35, 147.94, 134.29, 130.75, 128.63, 127.70, 127.01, 123.47, 123.34, 69.58, 44.50, 38.70 ppm.

HRMS (ESI+) *m/z* calcd. for [$C_{14}H_{13}NS+H^+$] 228.08415, found 228.08367.

Yield: 73 % (0.145 mmol, 33 mg), white solid.

3,5-diphenyl-3,4-dihydro-2*H*-pyrrole (**29**)[10]

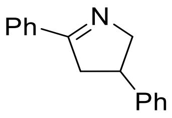

MW ($C_{16}H_{15}N$) = 221.30 g mol^{-1}

^{1}**H NMR** (500 MHz, CD_2Cl_2): δ = 7.89 – 7.85 (m, 2H), 7.47 – 7.40 (m, 3H), 7.30 (dt, *J* = 9.0, 1.6 Hz, 2H), 7.24 – 7.18 (m, 3H), 4.52 – 4.45 (m, 1H), 4.04 (ddt, *J* = 16.3, 6.1, 2.0 Hz, 1H), 3.66 (tt, *J* = 8.7, 6.4 Hz, 1H), 3.47 (dddd, *J* = 17.0, 9.7, 2.2, 1.4 Hz, 1H), 3.07 (ddt, *J* = 17.0, 6.7, 2.0 Hz, 1H) ppm.

^{13}**C NMR** (100 MHz, CD_2Cl_2): δ = 172.31, 145.69, 135.00, 130.73, 129.01, 128.80, 127.98, 127.21, 126.66, 70.04, 44.24, 43.42 ppm.

Yield: 96 % (0.192 mmol, 42m g), white solid.

2,3,5-triphenyl-3,4-dihydro-2*H*-pyrrole (**30**)[13]

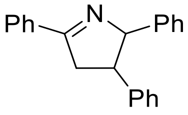

MW ($C_{22}H_{19}N$) = 297.40 g mol^{-1}

^{1}**H NMR** (400 MHz, acetone-D_6): δ = 8.05 – 8.00 (m, 4H), 7.53 – 7.46 (m, 6H), 7.34 – 7.18 (m, 20H), 5.28 (t, *J* = 2.0 Hz, 1H), 5.26 (t, *J* = 2.1 Hz, 1H), 3.74 (dd, *J* = 9.2,

1.9 Hz, 1H), 3.70 (dd, *J* = 9.2, 1.9 Hz, 1H), 3.49 (d, *J* = 8.4 Hz, 1H), 3.45 (d, *J* = 9.0 Hz, 1H), 3.26 (dd, *J* = 8.4, 2.3 Hz, 1H), 3.22 (dd, *J* = 8.4, 2.3 Hz, 1H) ppm.

^{3}C NMR (100 MHz, acetone-D_6): δ = 172.64, 144.90, 144.50, 135.54, 131.43, 129.53, 129.30, 129.06, 128.72, 128.31, 127.64, 127.44, 127.36, 84.70, 54.54, 45.38 ppm.

dr = 50:50 (enantiomeric mixture)

Yield: 84 % (0.168 mmol, 50 mg), white solid.

2-(4-chlorophenyl)-3,5-diphenyl-3,4-dihydro-2*H*-pyrrole (**31**)

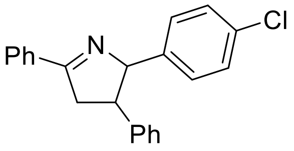

MW ($C_{22}H_{18}NCl$) = 331.84 g mol^{-1}

^{1}H NMR (400 MHz, CD_2Cl_2): δ = 7.99 – 7.94 (m, 2H), 7.52 – 7.45 (m, 3H), 7.34 – 7.23 (m, 7H), 7.14 – 7.10 (m, 2H), 5.25 (t, *J* = 2.0 Hz, 0.52H), 5.23 (t, *J* = 2.1 Hz, 0.48H), 3.67 (dd, *J* = 9.1, 1.9 Hz, 0.52H), 3.63 (dd, *J* = 9.1, 1.9 Hz, 0.60H), 3.40 (d, *J* = 8.5 Hz, 0.52H), 3.36 (d, *J* = 8.8 Hz, 0.52H), 3.25 (dd, *J* = 8.5, 2.3 Hz, 0.51H), 3.21 (dd, *J* = 8.5, 2.2 Hz, 0.59H).

^{3}C NMR (100 MHz, CD_2Cl_2): δ = 173.02, 143.31, 142.71, 134.61, 132.90, 131.23, 130.32, 129.16, 128.91, 128.78, 128.35, 128.23, 127.77, 127.11, 127.00, 83.62, 54.08, 45.22 ppm.

HRMS (ESI+) *m/z* calcd. for [$C_{22}H_{18}NCl+H^+$] 332.12005, found 332.11963.

dr = 52:48 (enantiomeric mixture)

Yield: 81 % (0.163 mmol, 54 mg), white solid.

2-(4-fluorophenyl)-3,5-diphenyl-3,4-dihydro-2*H*-pyrrole (**32**)

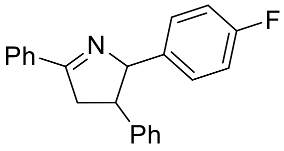

MW ($C_{22}H_{18}NF$) = 315.39 g mol^{-1}

^{1}**H NMR** (300 MHz, CD_2Cl_2): δ = 8.05 – 7.92 (m, 2H), 7.52 – 7.43 (m, 3H), 7.35 – 7.14 (m, 7H), 7.08 – 6.94 (m, 2H), 5.27 (s, 0.48H), 5.24 (s, 0.52H), 3.69 (dd, *J* = 9.1, 1.7 Hz, 0.53H), 3.63 (dd, *J* = 9.1, 1.7 Hz, 0.68H), 3.43 (d, *J* = 8.2 Hz, 0.51H), 3.37 (d, *J* = 8.6 Hz, 0.60H), 3.25 (dd, *J* = 8.3, 2.2 Hz, 0.69H), 3.20 (dd, *J* = 8.3, 2.2 Hz, 0.58H).

^{3}**C NMR** (75 MHz, CD_2Cl_2): δ = 172.72, 163.98, 143.49, 140.00, 134.72, 131.19, 129.16, 128.92, 128.60, 128.49, 128.24, 127.77, 127.09, 115.56, 115.27, 83.69, 54.14, 45.19 ppm.

HRMS (ESI+) *m/z* calcd. for [$C_{22}H_{18}NF+H^{+}$] 316.14960, found 316.14896.

dr = 48:52 (enantiomeric mixture)

Yield: 76 % (0.152 mmol, 48 mg), white solid.

3,5-diphenyl-2-(4-(trifluoromethyl)phenyl)-3,4-dihydro-2*H*-pyrrole (**33**)

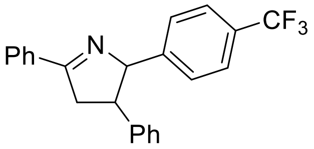

MW ($C_{23}H_{18}NF_3$) = 365.40 g mol^{-1}

^{1}**H NMR** (400 MHz, $CDCl_3$): δ = 8.01 – 7.96 (m, 2H), 7.58 – 7.47 (m, 5H), 7.36 – 7.27 (m, 5H), 7.24 – 7.20 (m, 2H), 5.37 (s, 0.48H), 5.35 (s, 0.52H), 3.70 (dd, *J* = 9.1, 1.9 Hz, 0.48H), 3.66 (dd, *J* = 9.2, 1.9 Hz, 0.58H), 3.42 (d, *J* = 8.1 Hz, 0.49H), 3.37 (d, *J* = 7.6 Hz, 0.54H), 3.29 (dd, *J* = 8.2, 2.1 Hz, 0.63H), 3.25 (dd, *J* = 8.2, 2.1 Hz, 0.49H).

^{13}**C NMR** (75 MHz, $CDCl_3$): δ = 173.40, 147.53, 142.91, 134.02, 131.28, 129.10, 128.81, 128.10, 127.46, 127.11, 126.87, 125.58, 125.53, 83.73, 53.62, 45.16 ppm.

HRMS (ESI+) *m/z* calcd. for [$C_{23}H_{18}NF_3$+H^+] 366.14641, found 366.14576.

dr = 48:52 (enantiomeric mixture)

Yield: 70 % (0.140 mmol, 51 mg), white solid.

10. NMR Spectra

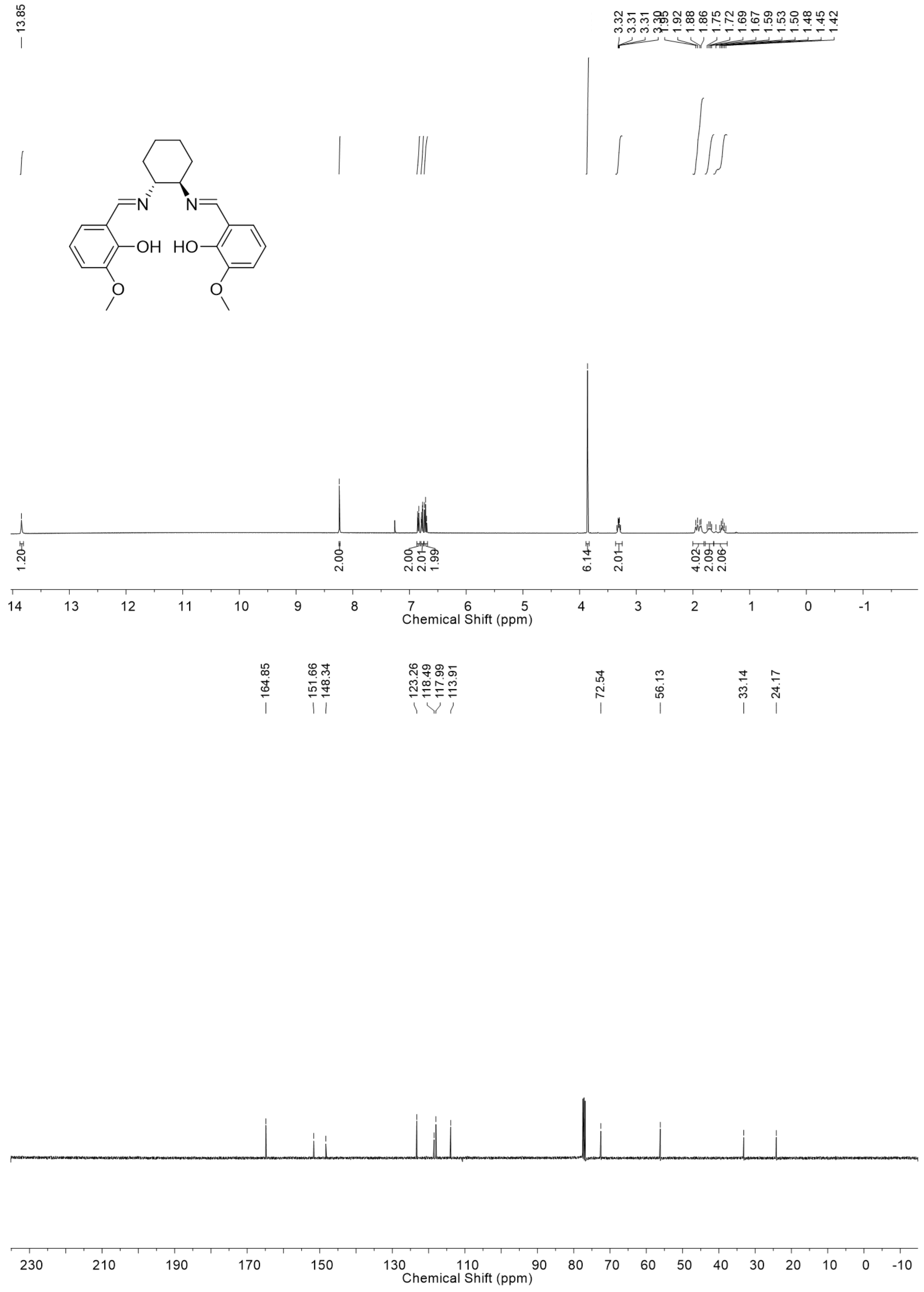

Figure S 21: NMR Spectra of the salen ligand.

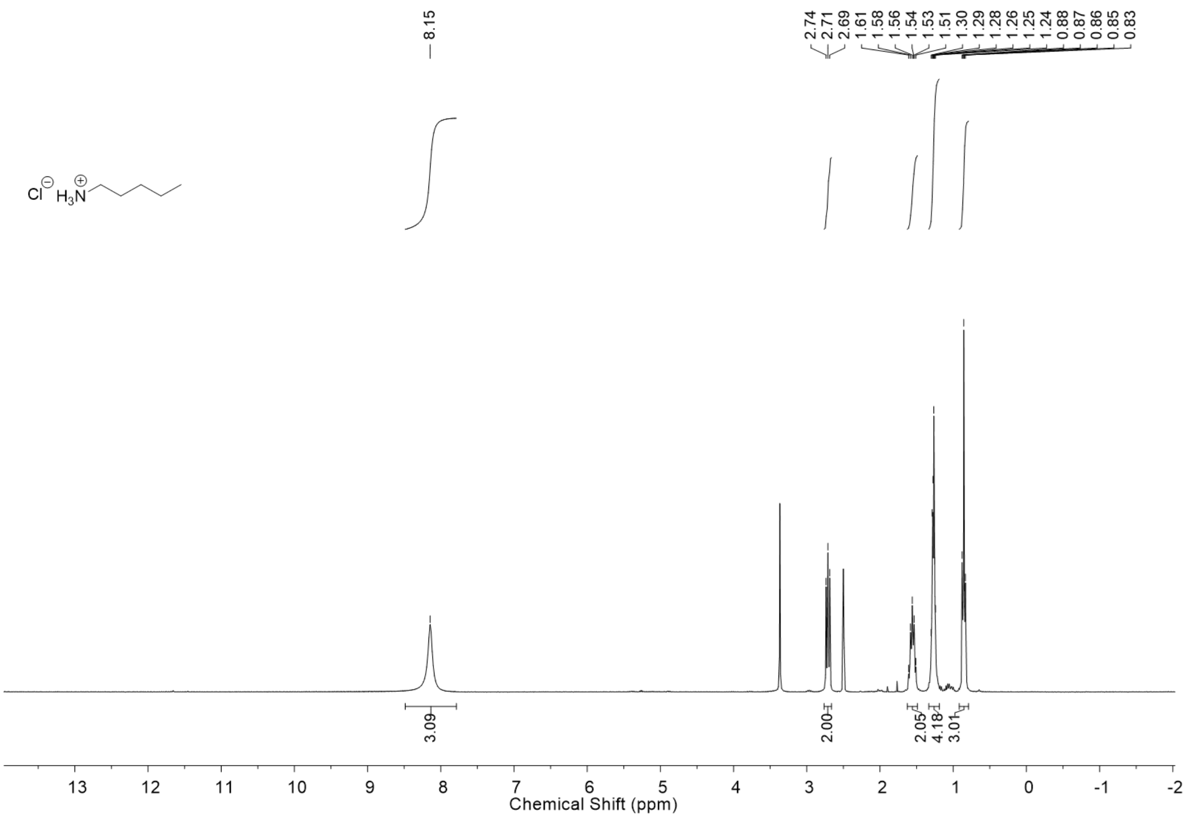

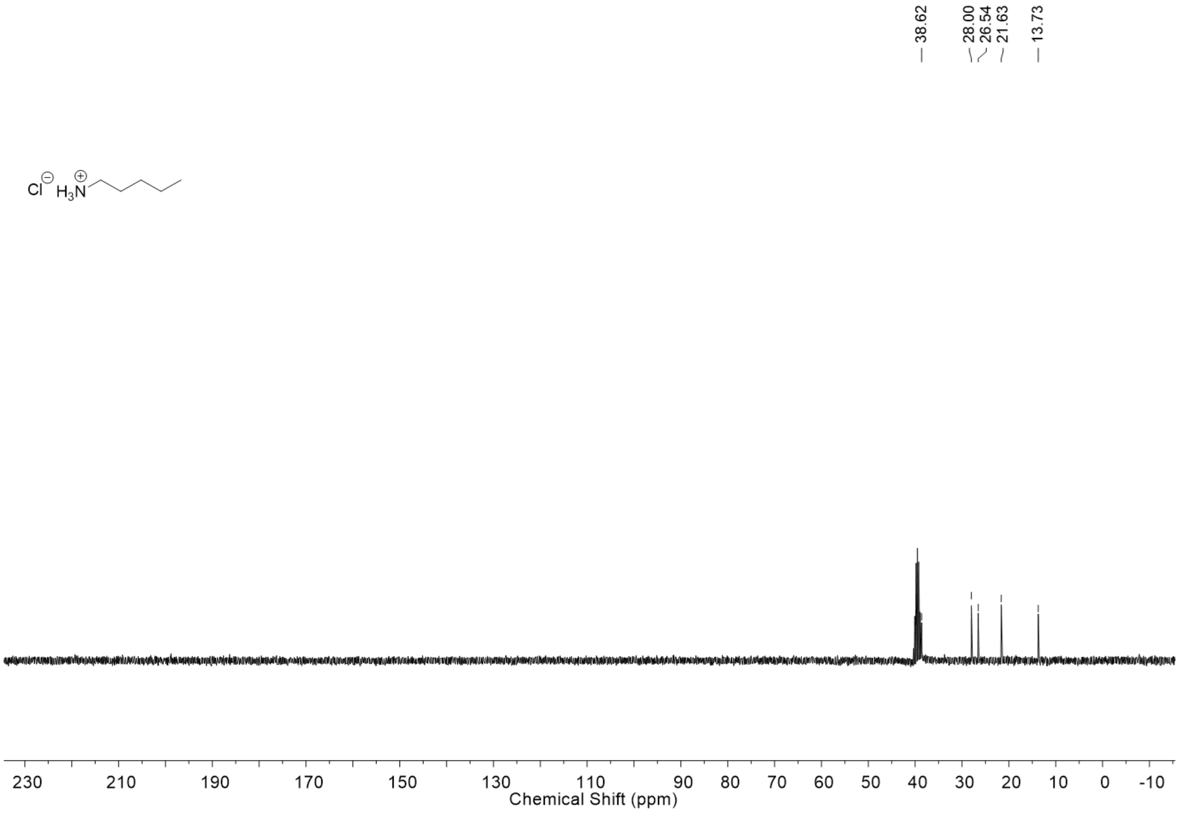

Figure S 22: NMR Spectra of pentan-1-amonium chloride.

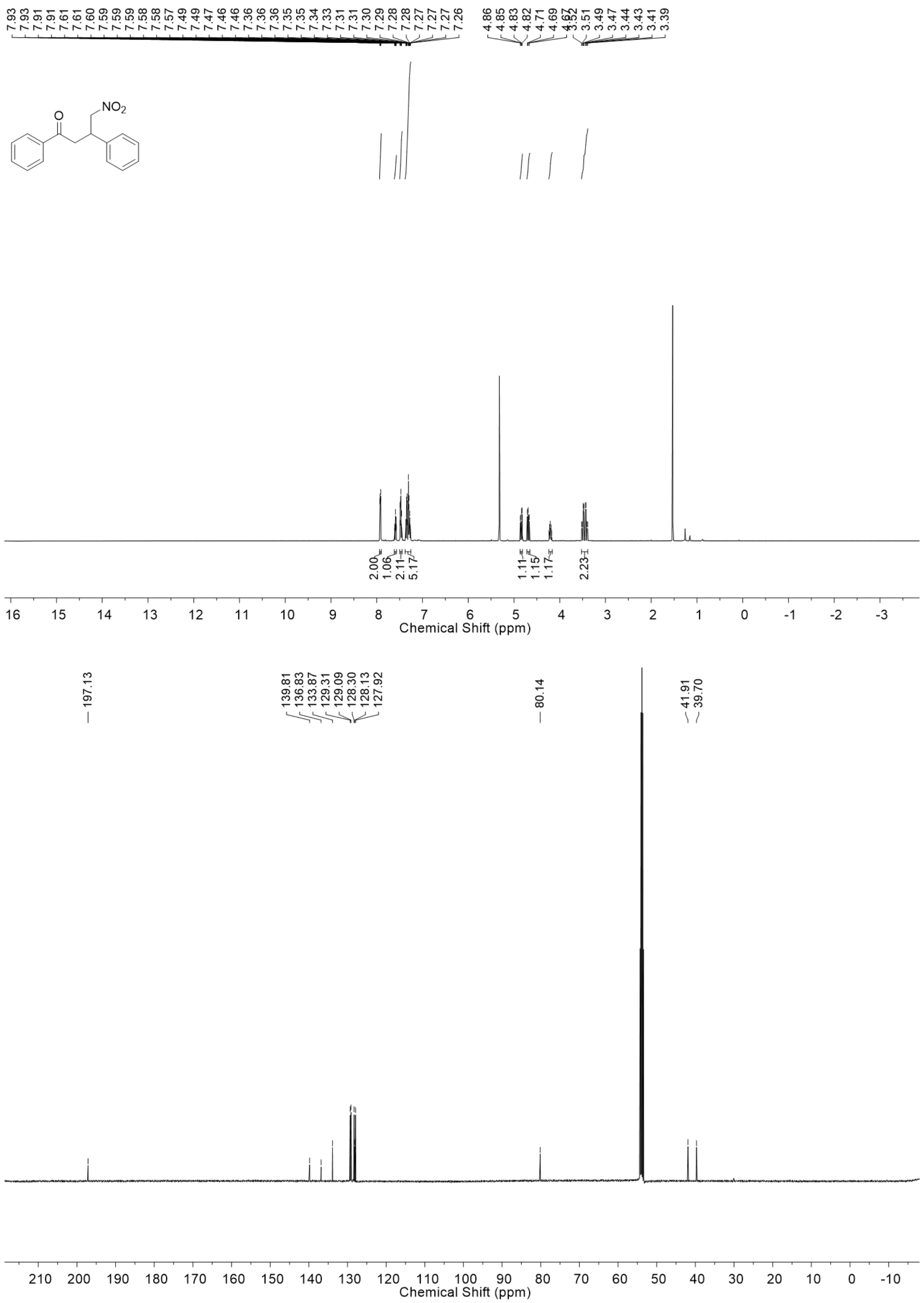

Figure S 23: NMR Spectra of 4-nitro-1,3-diphenylbutan-1-one.

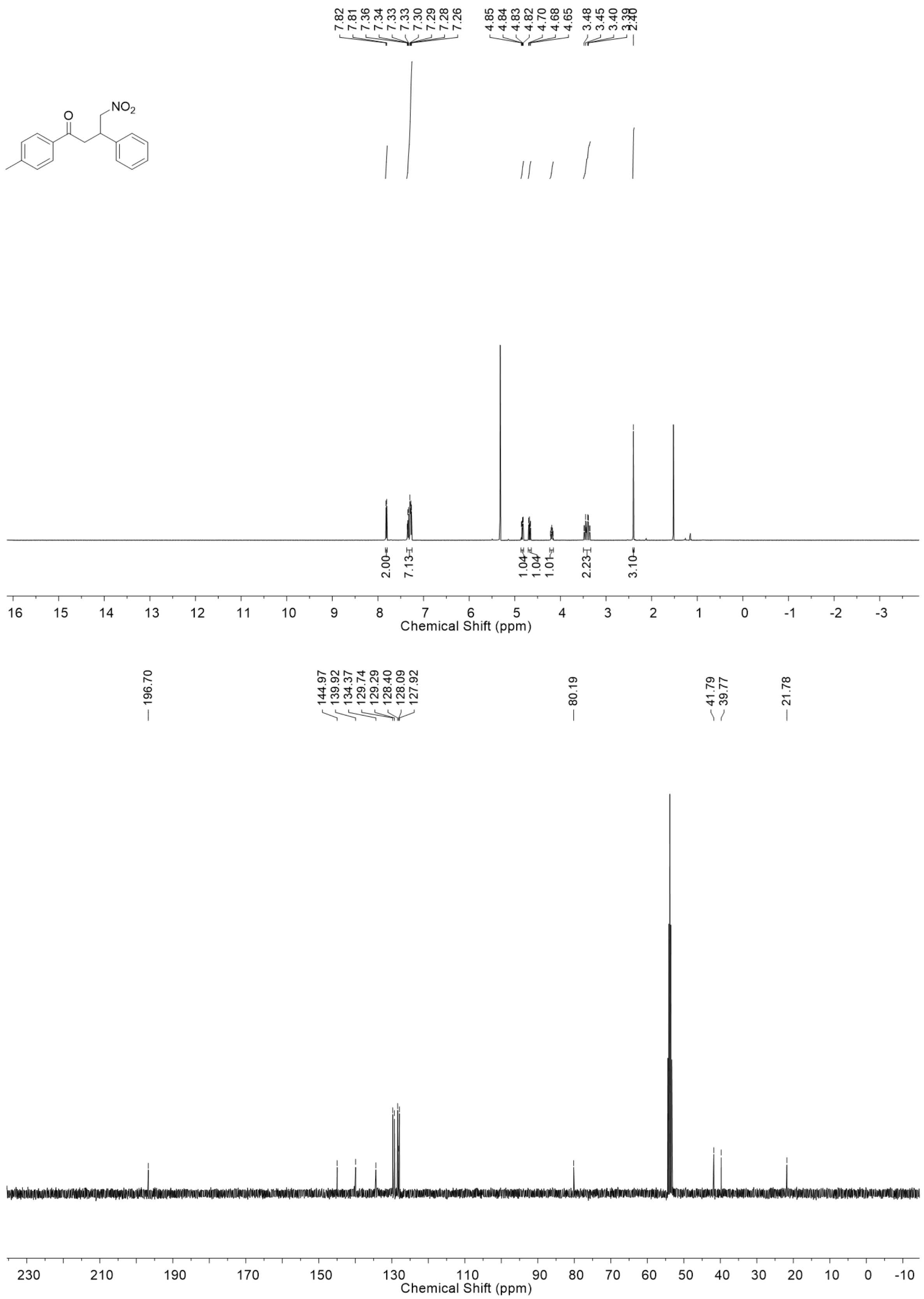

Figure S 24: NMR Spectra of 4-nitro-3-phenyl-1-(*p*-tolyl)butan-1-one.

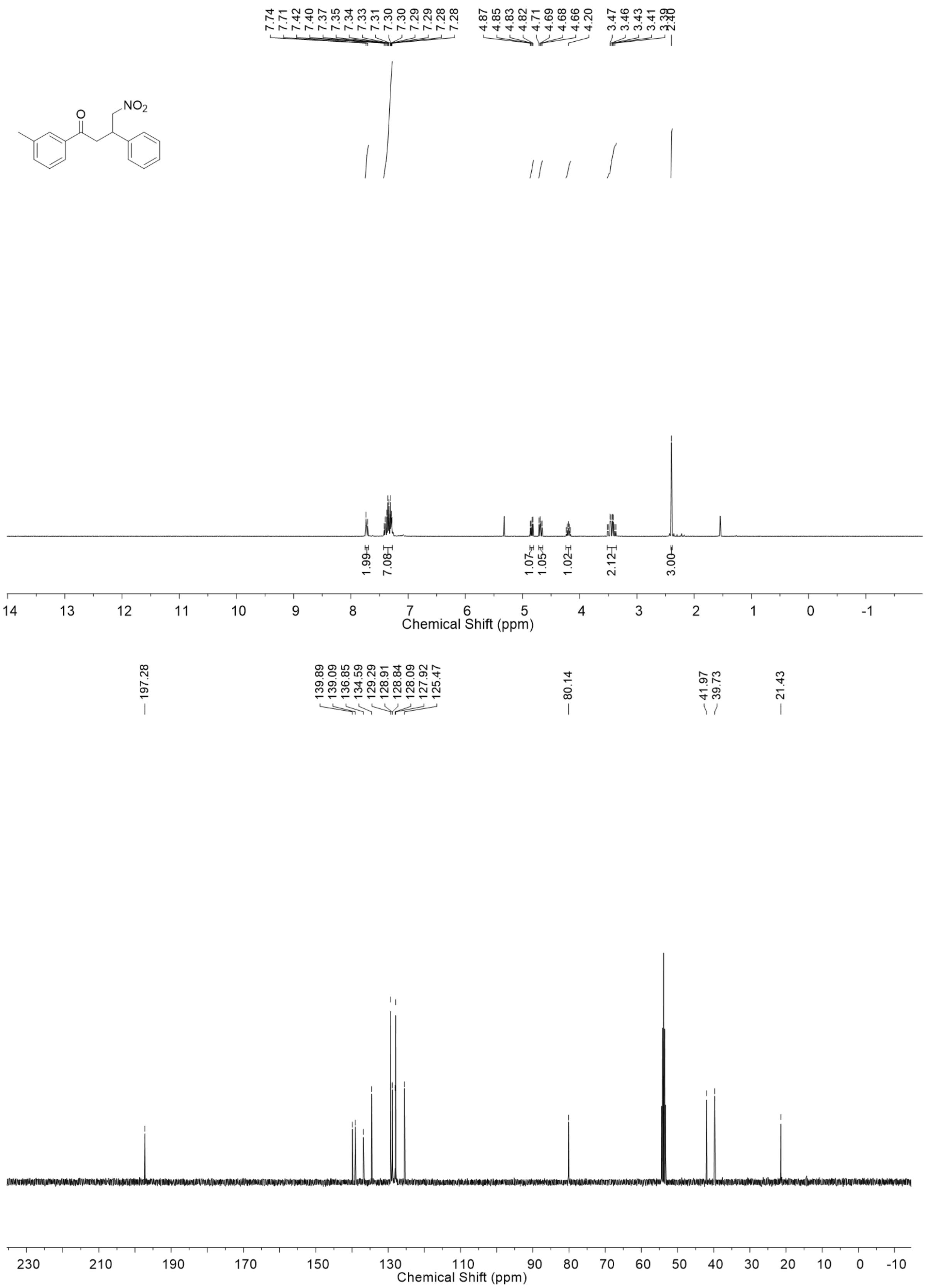

Figure S 25: NMR Spectra of 4-nitro-3-phenyl-1-(*m*-tolyl)butan-1-one.

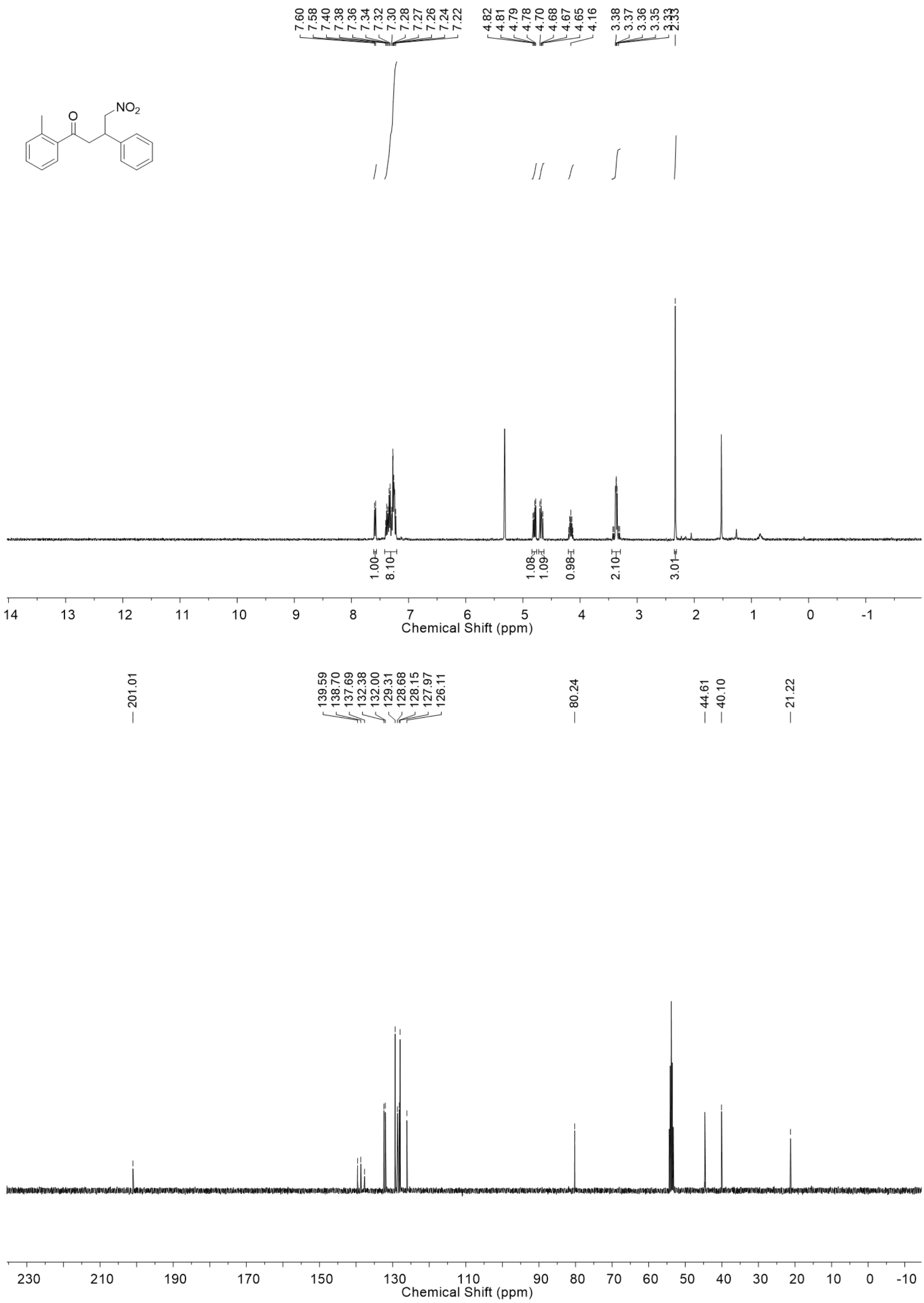

Figure S 26: NMR Spectra of 4-nitro-3-phenyl-1-(*o*-tolyl)butan-1-one.

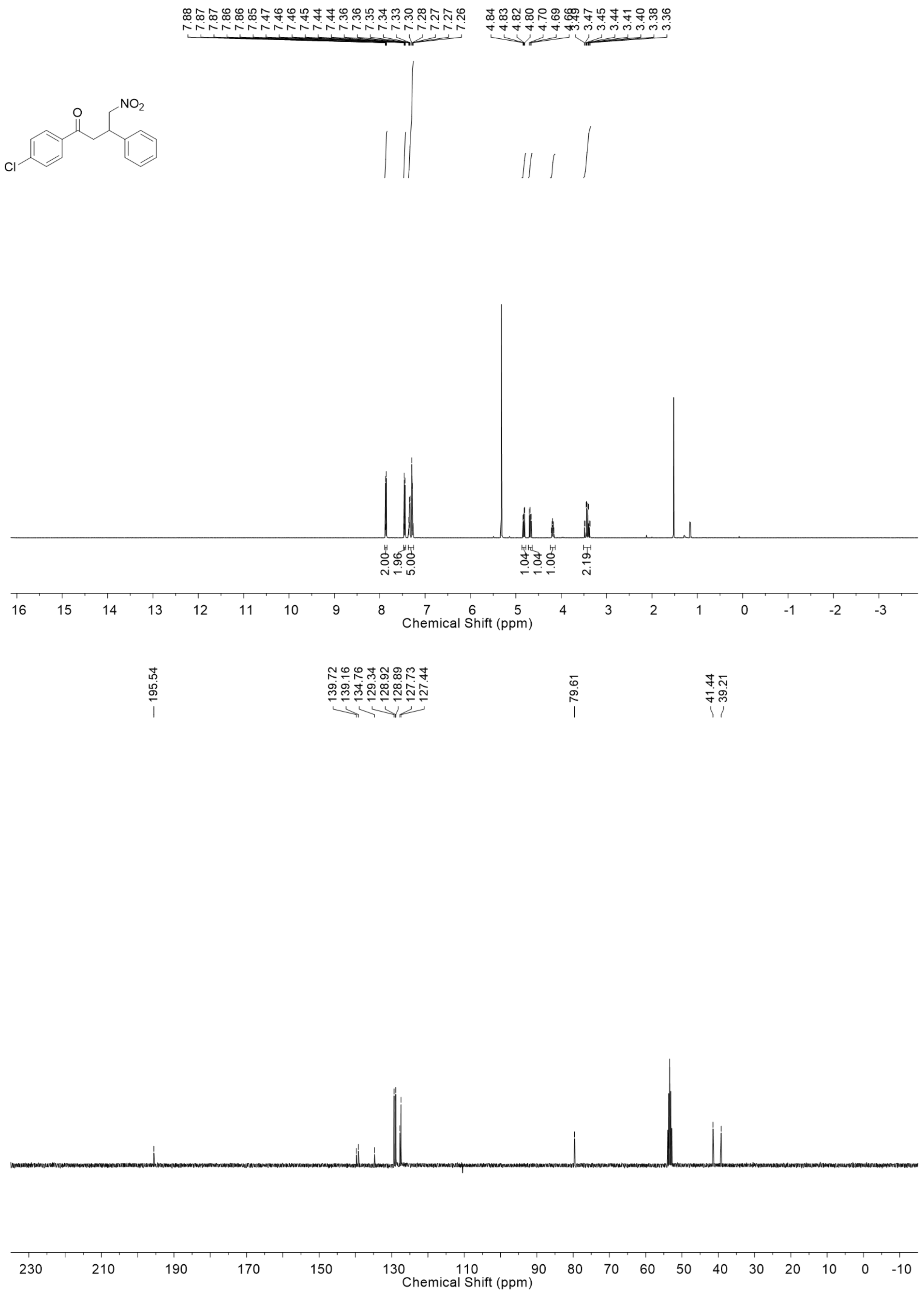

Figure S 27: NMR Spectra of **1**-(4-chlorophenyl)-4-nitro-3-phenylbutan-1-one.

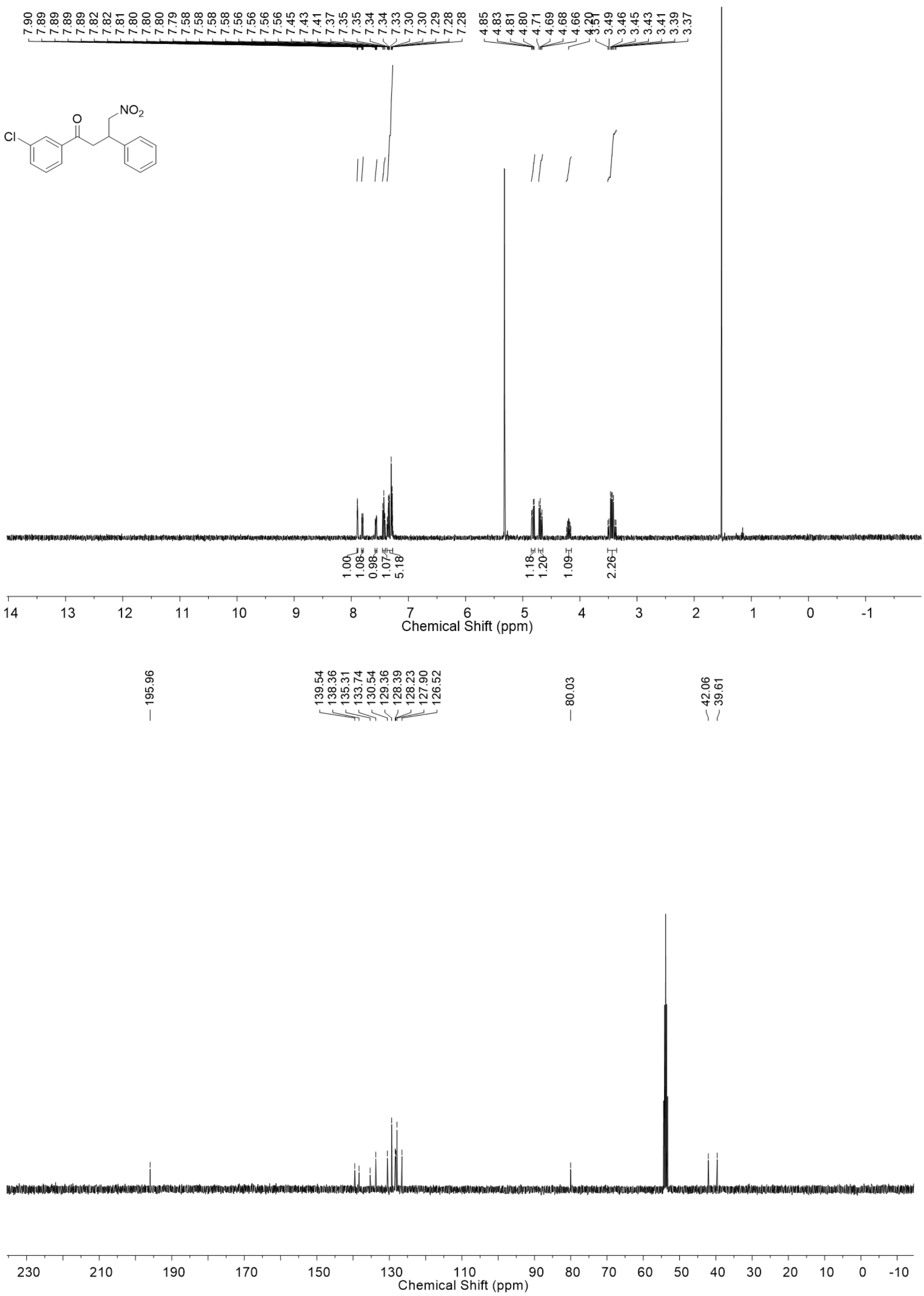

Figure S 28: NMR Spectra of 1-(3-chlorophenyl)-4-nitro-3-phenylbutan-1-one.

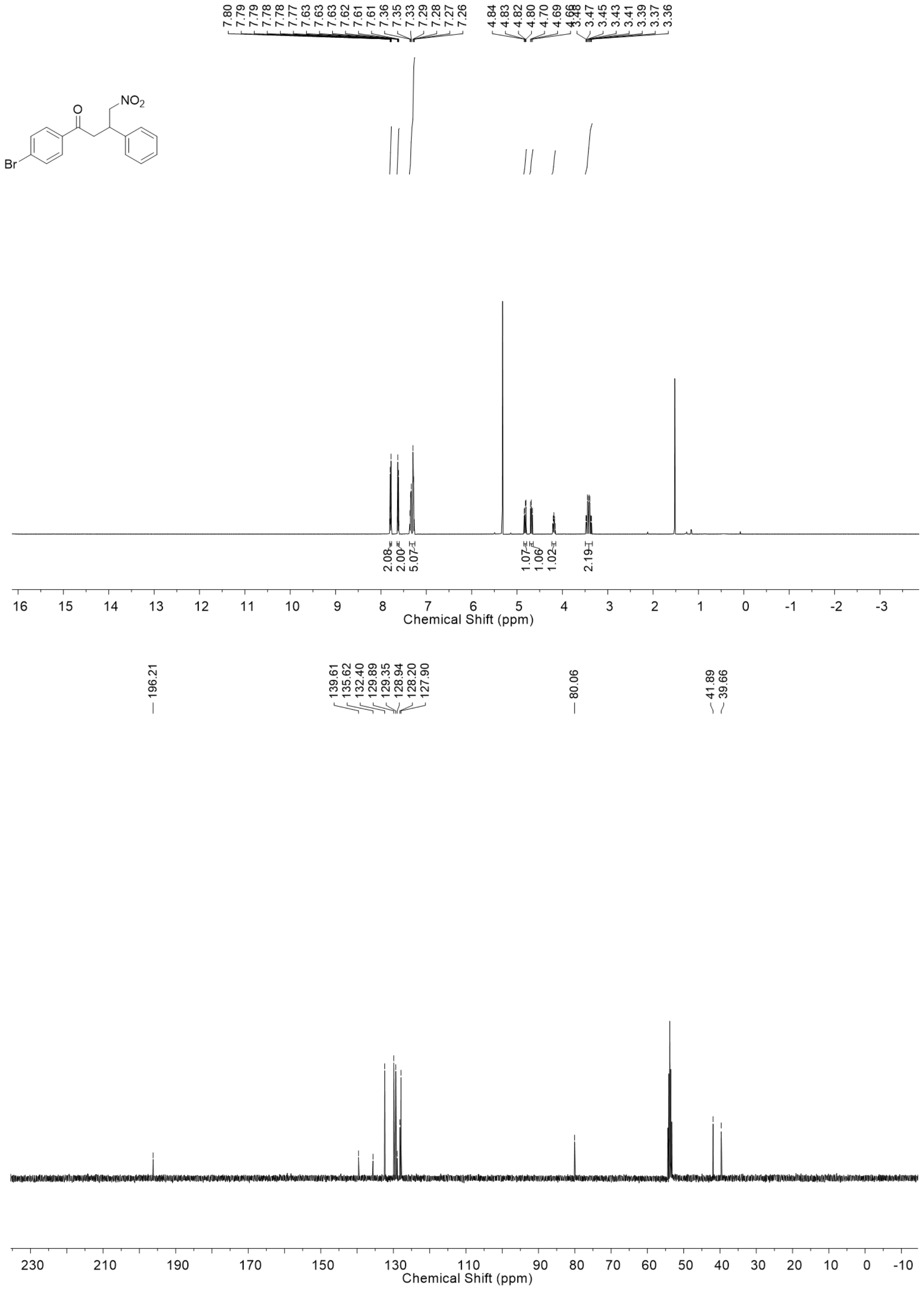

Figure S 29: NMR Spectra of 1-(4-bromophenyl)-4-nitro-3-phenylbutan-1-one.

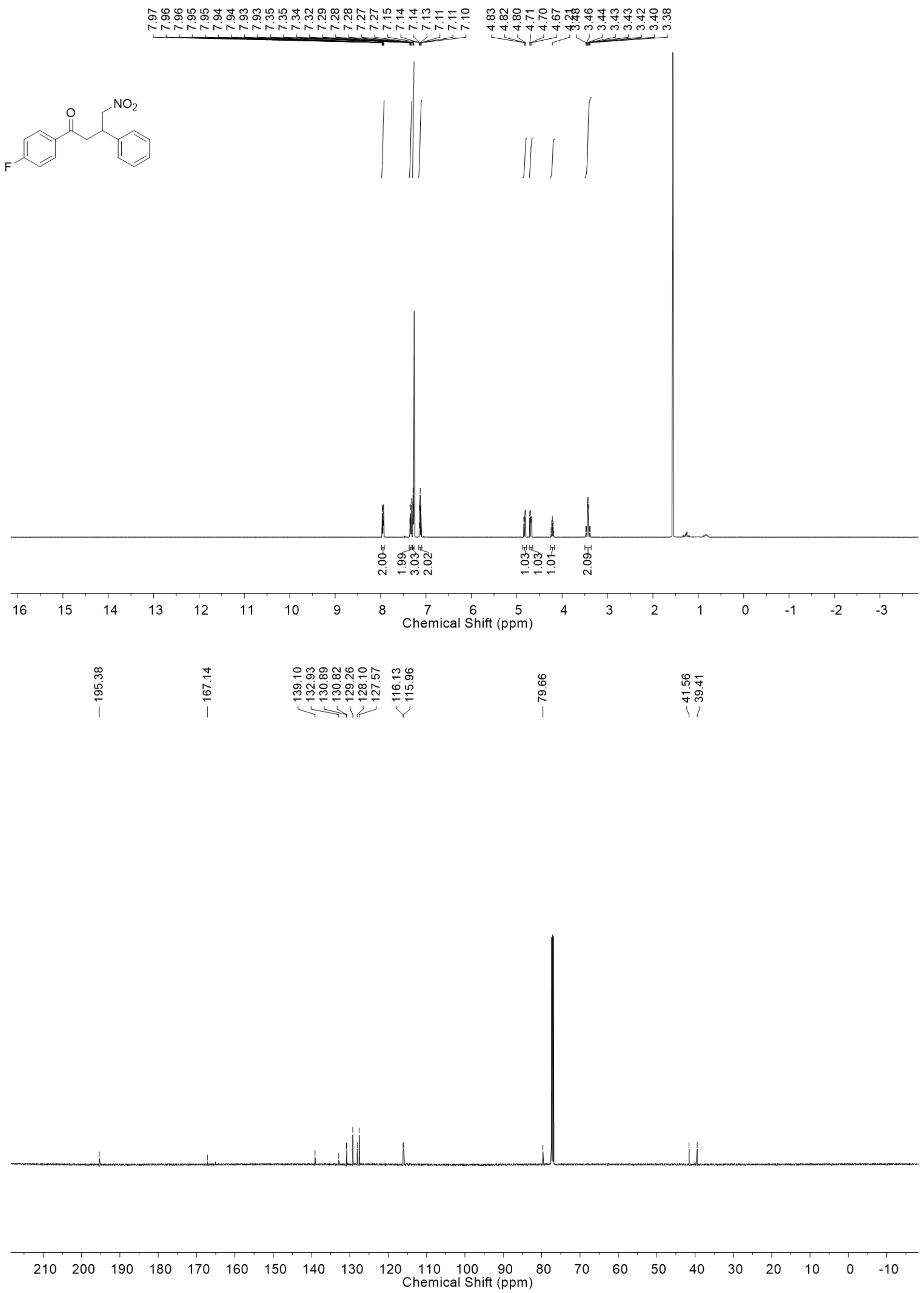

Figure S 30: NMR Spectra of 1-(4-fluorophenyl)-4-nitro-3-phenylbutan-1-one.

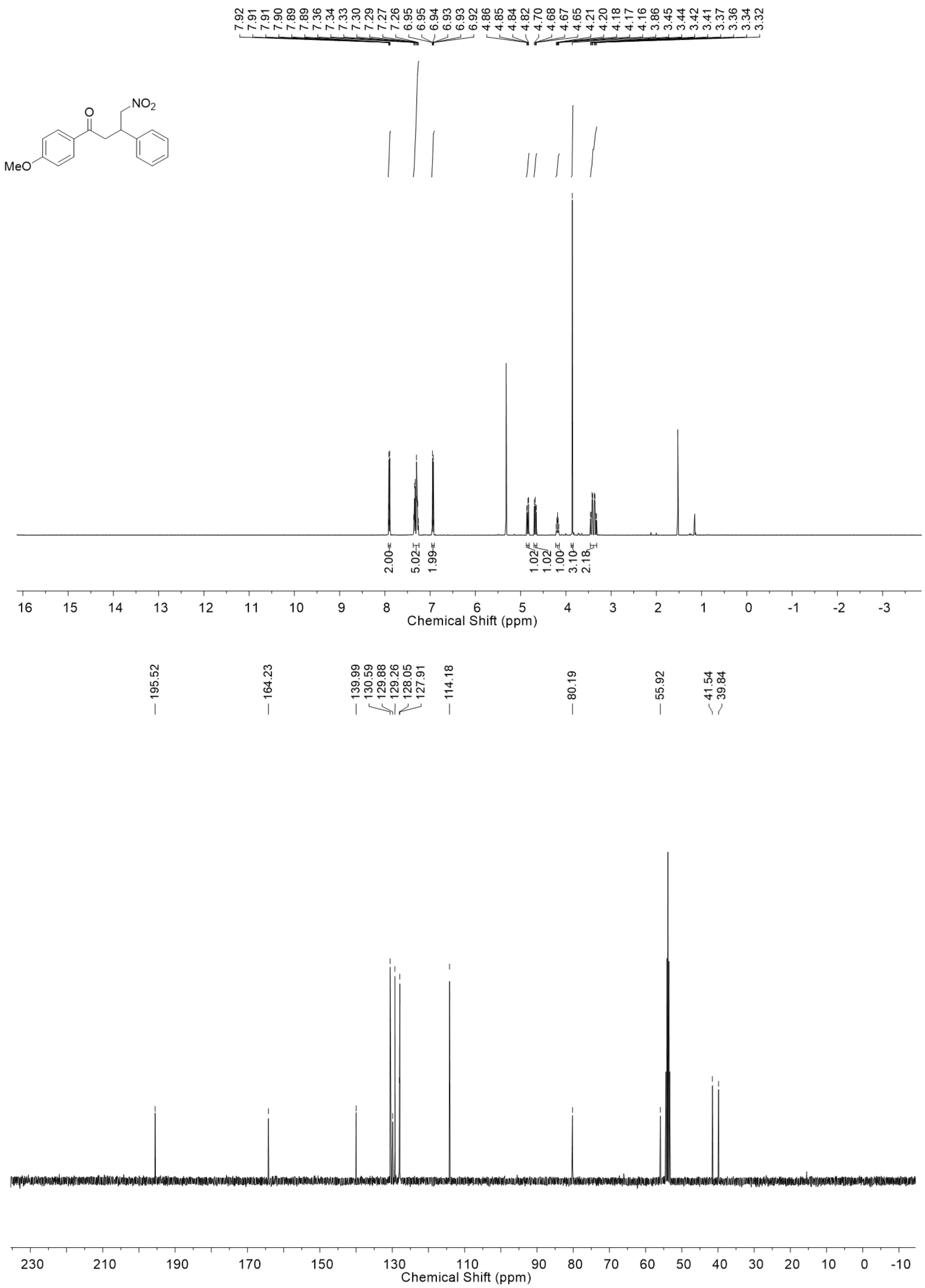

Figure S 31: NMR Spectra of 1-(4-methoxyphenyl)-4-nitro-3-phenylbutan-1-one.

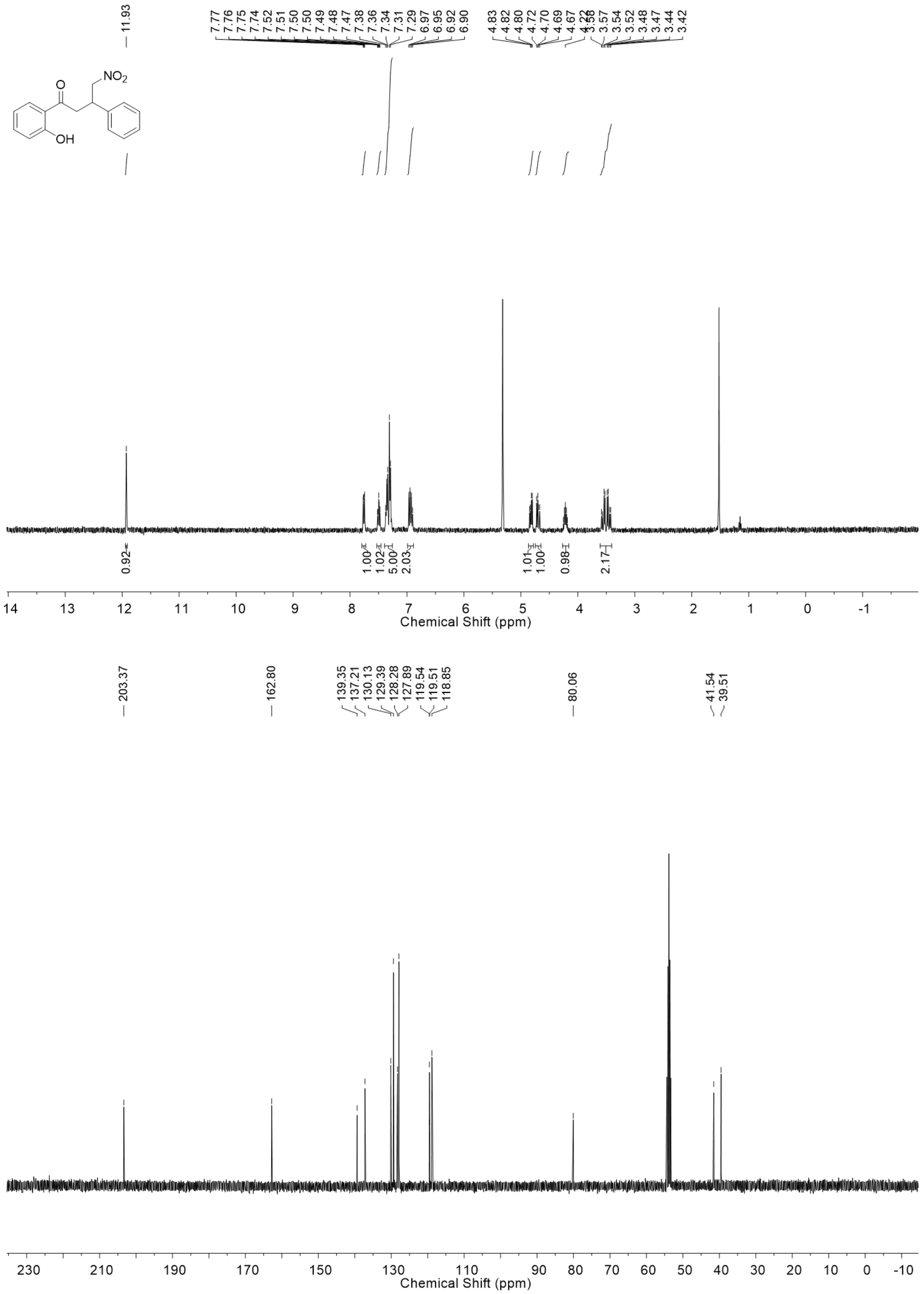

Figure S 32: NMR Spectra of 1-(2-hydroxyphenyl)-4-nitro-3-phenylbutan-1-one.

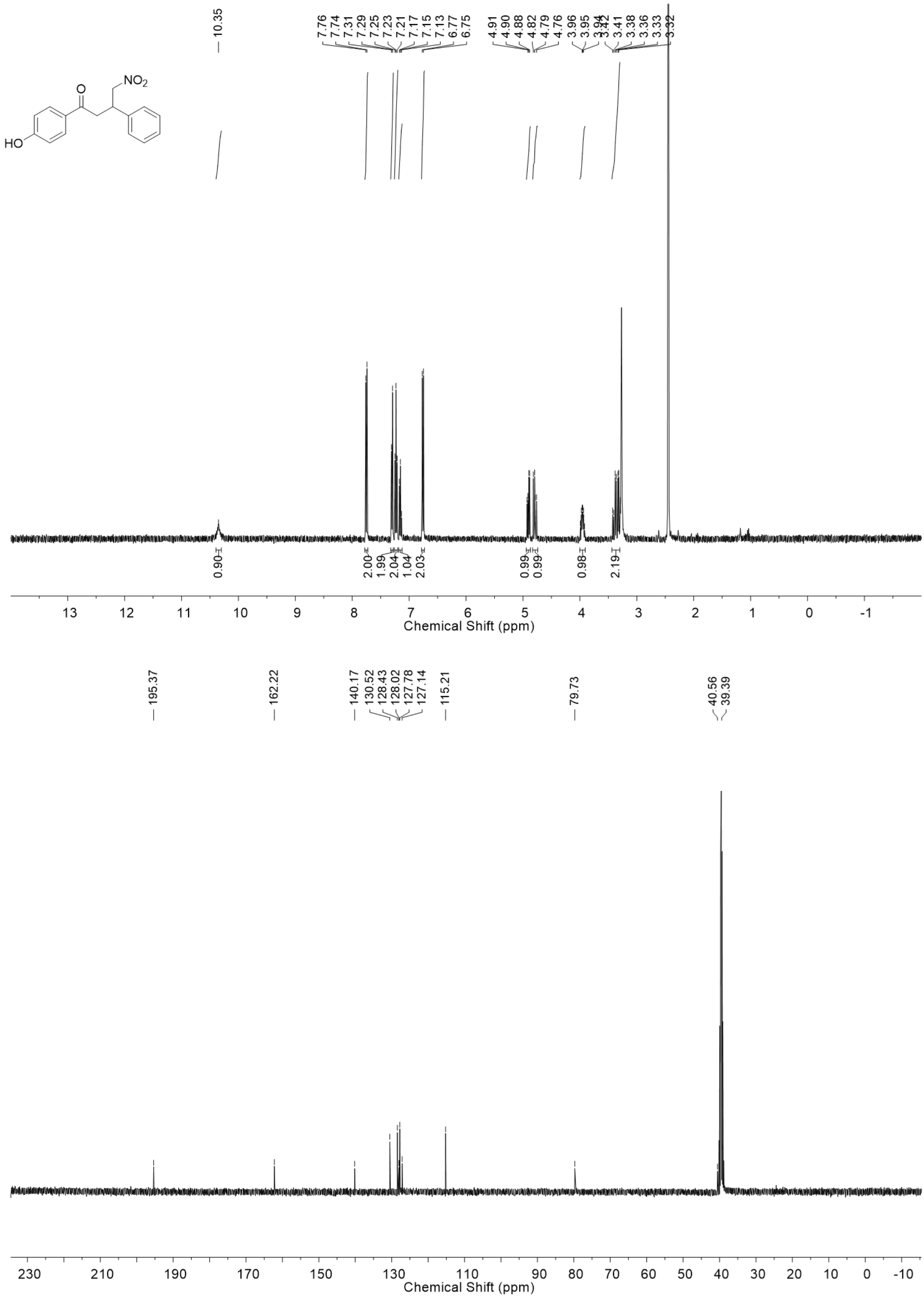

Figure S 33: NMR Spectra of 1-(4-hydroxyphenyl)-4-nitro-3-phenylbutan-1-one.

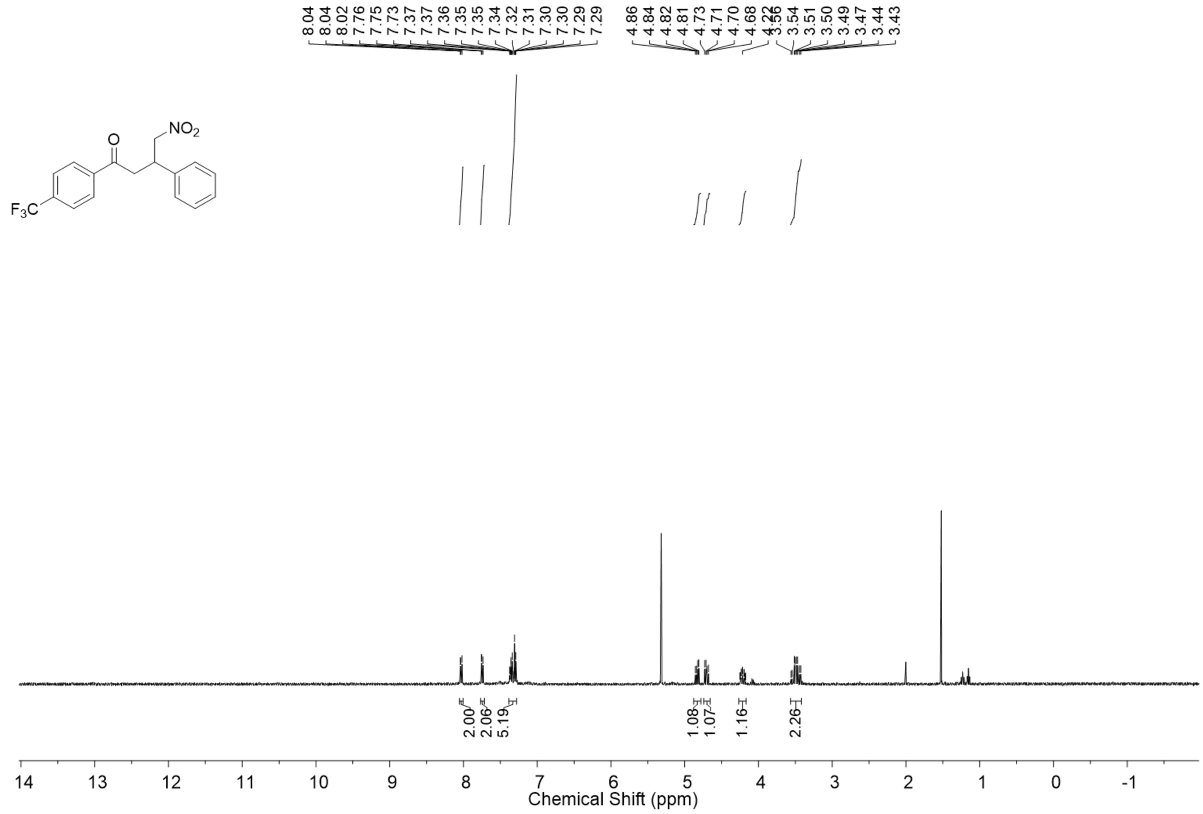

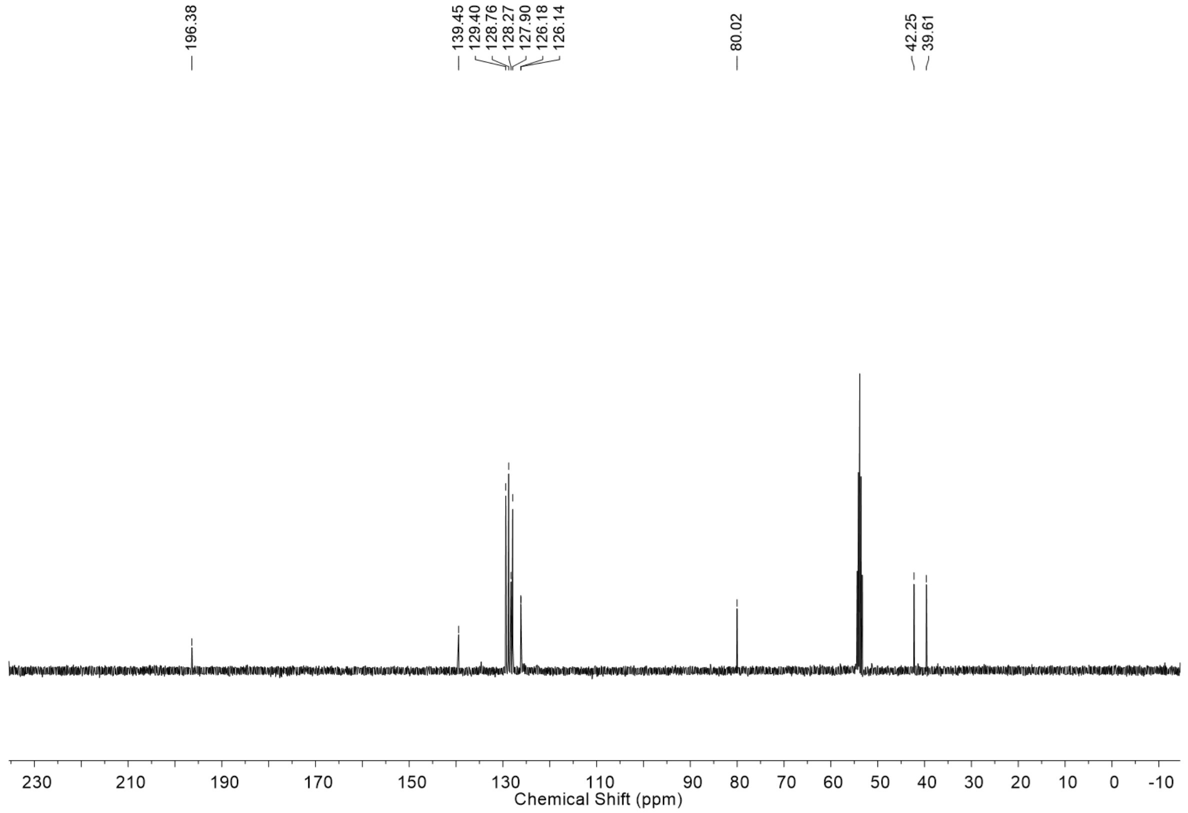

Figure S 34: NMR Spectra of 4-nitro-3-phenyl-1-(4-(trifluoromethyl)phenyl)butan-1-one.

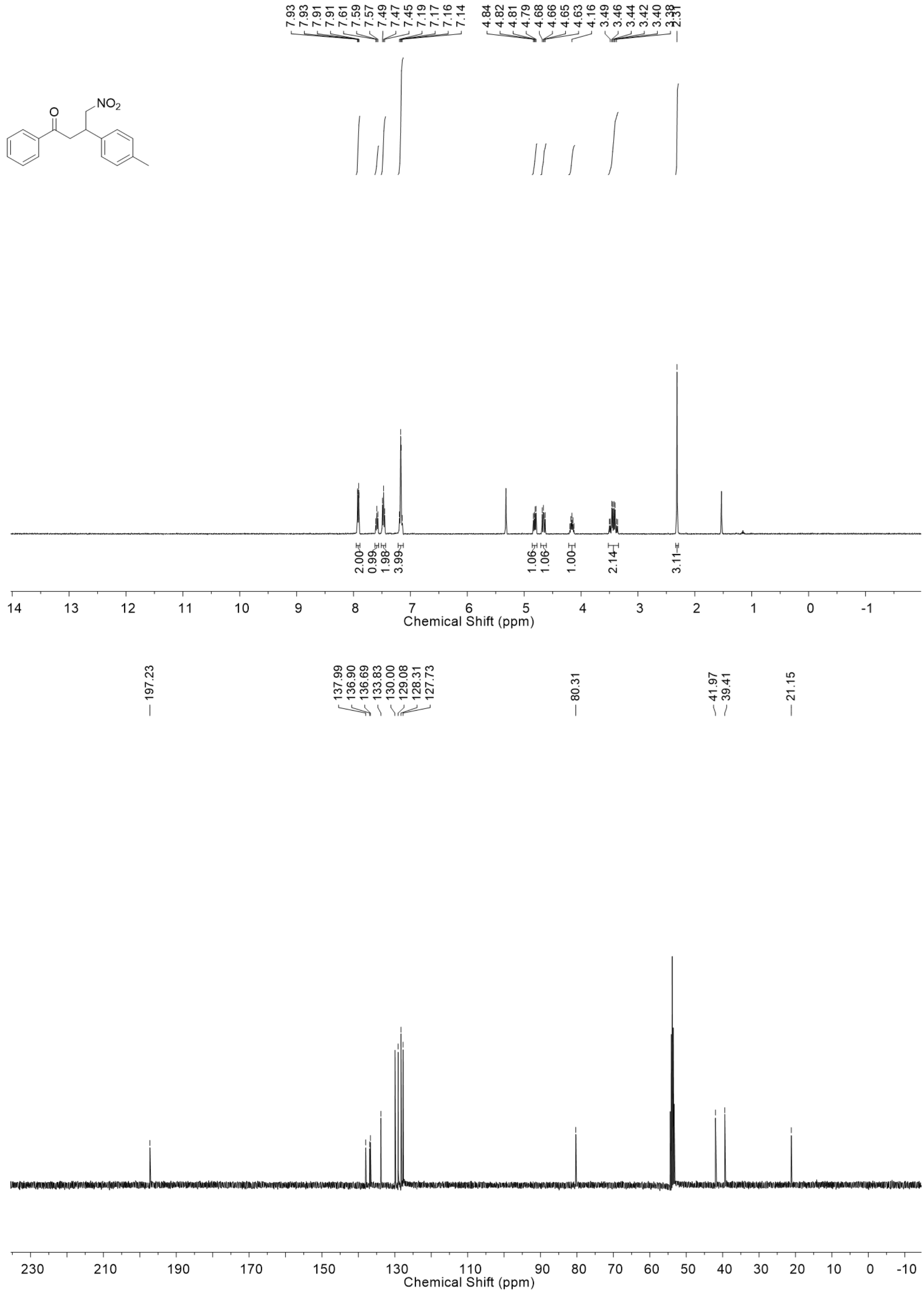

Figure S 35: NMR Spectra of 4-nitro-1-phenyl-3-(*p*-tolyl)butan-1-one.

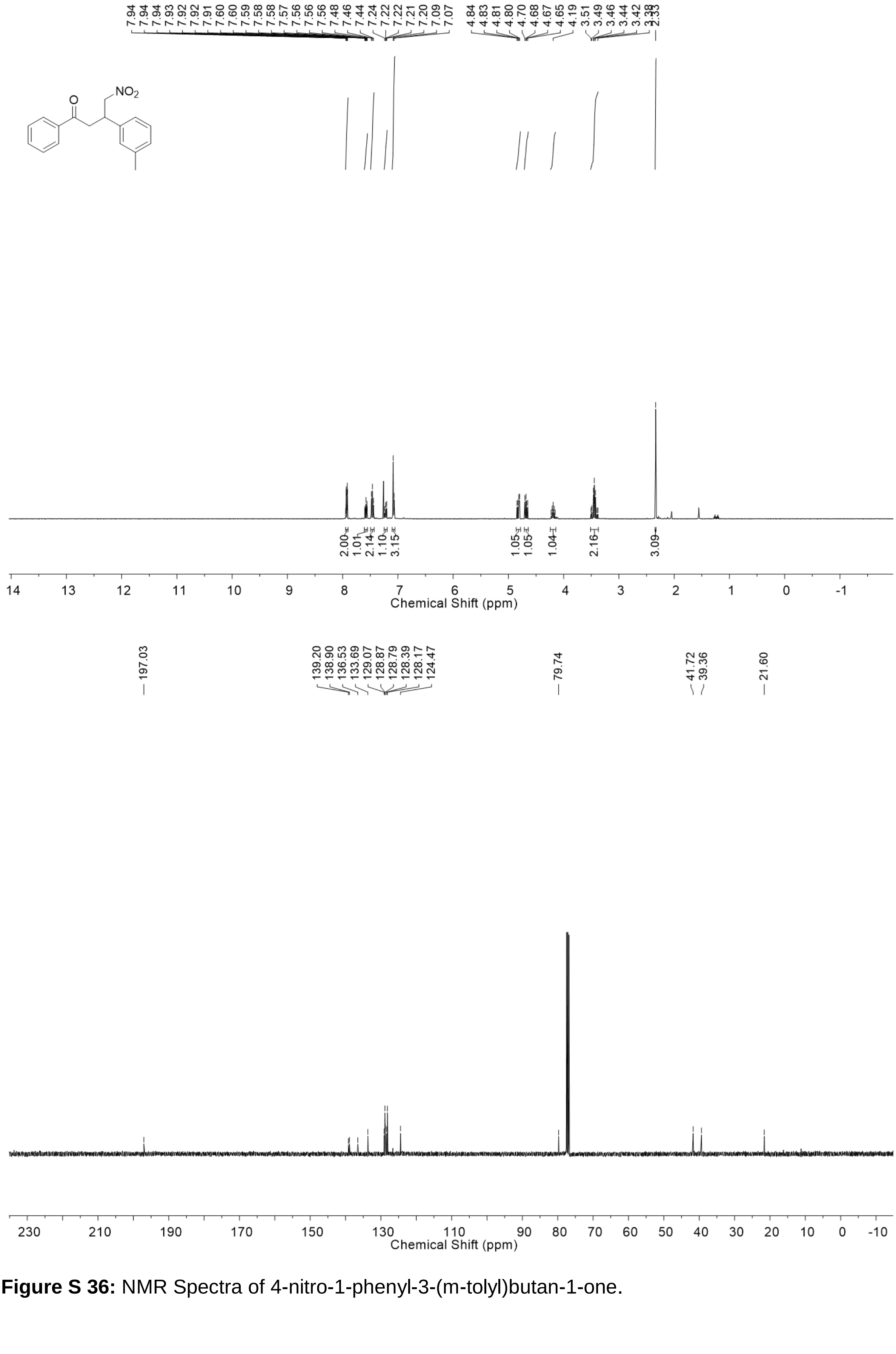

Figure S 36: NMR Spectra of 4-nitro-1-phenyl-3-(m-tolyl)butan-1-one.

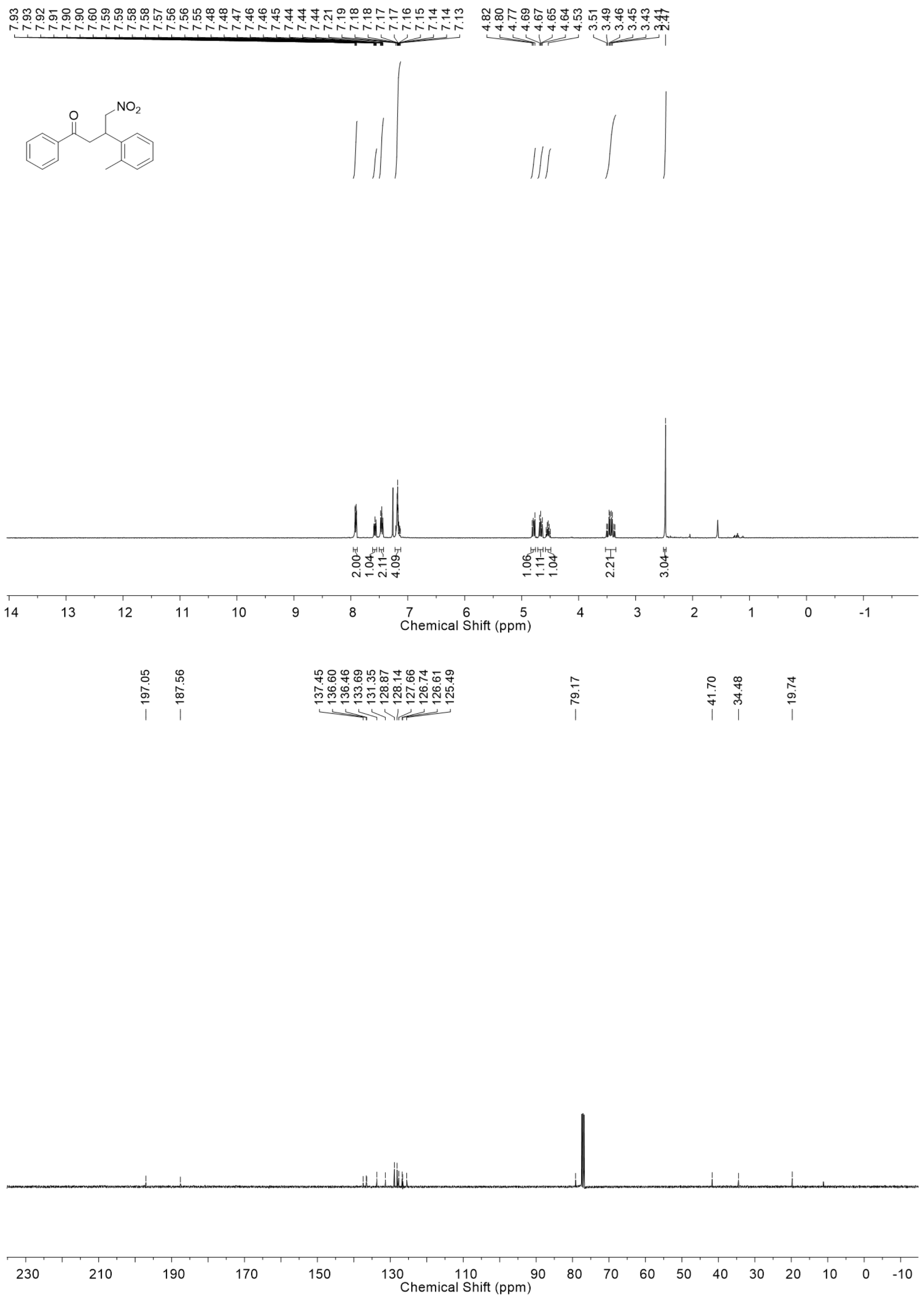

Figure S 37: NMR Spectra of 4-nitro-1-phenyl-3-(o-tolyl)butan-1-one.

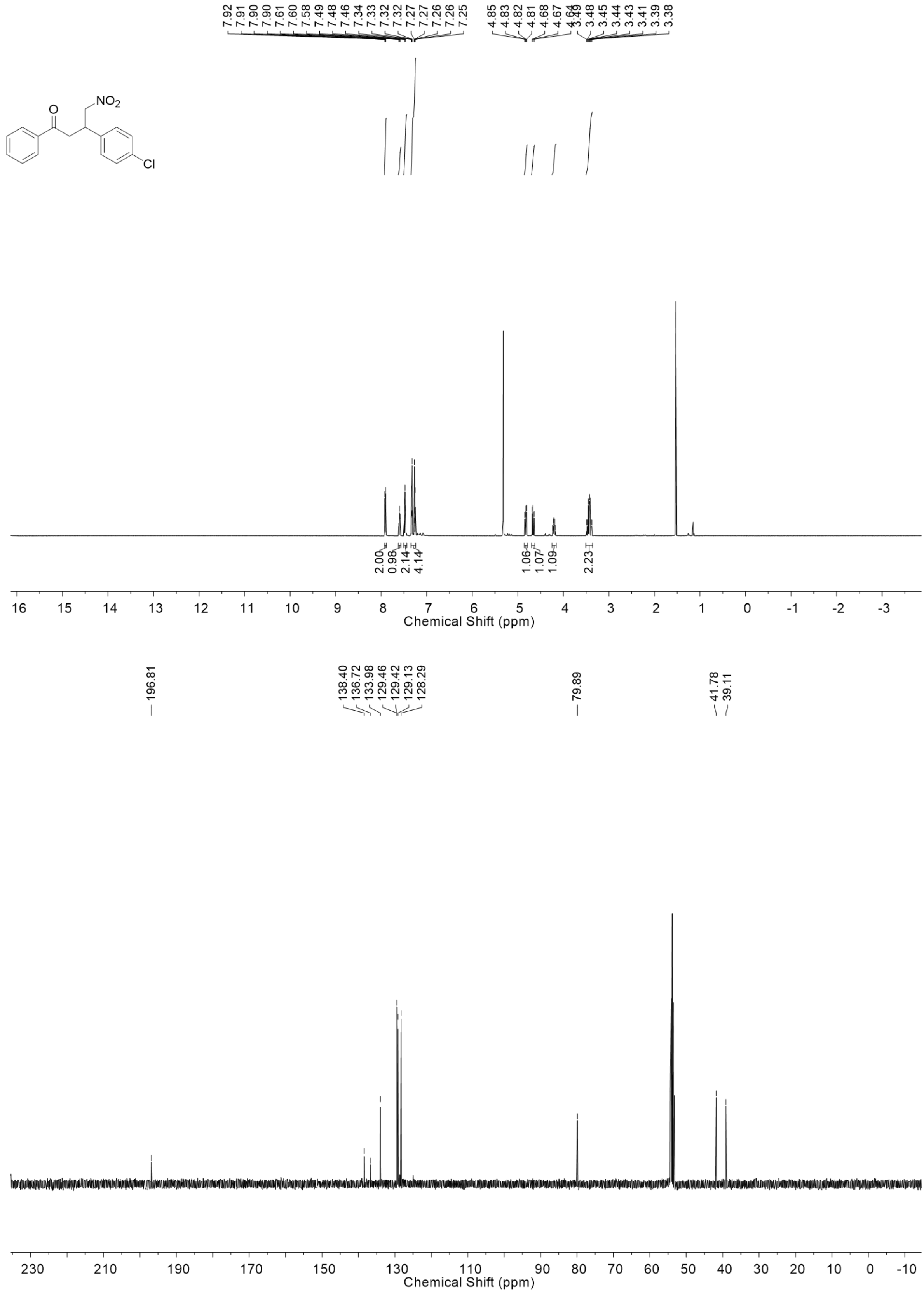

Figure S 38: NMR Spectra of 3-(4-chlorophenyl)-4-nitro-1-phenylbutan-1-one.

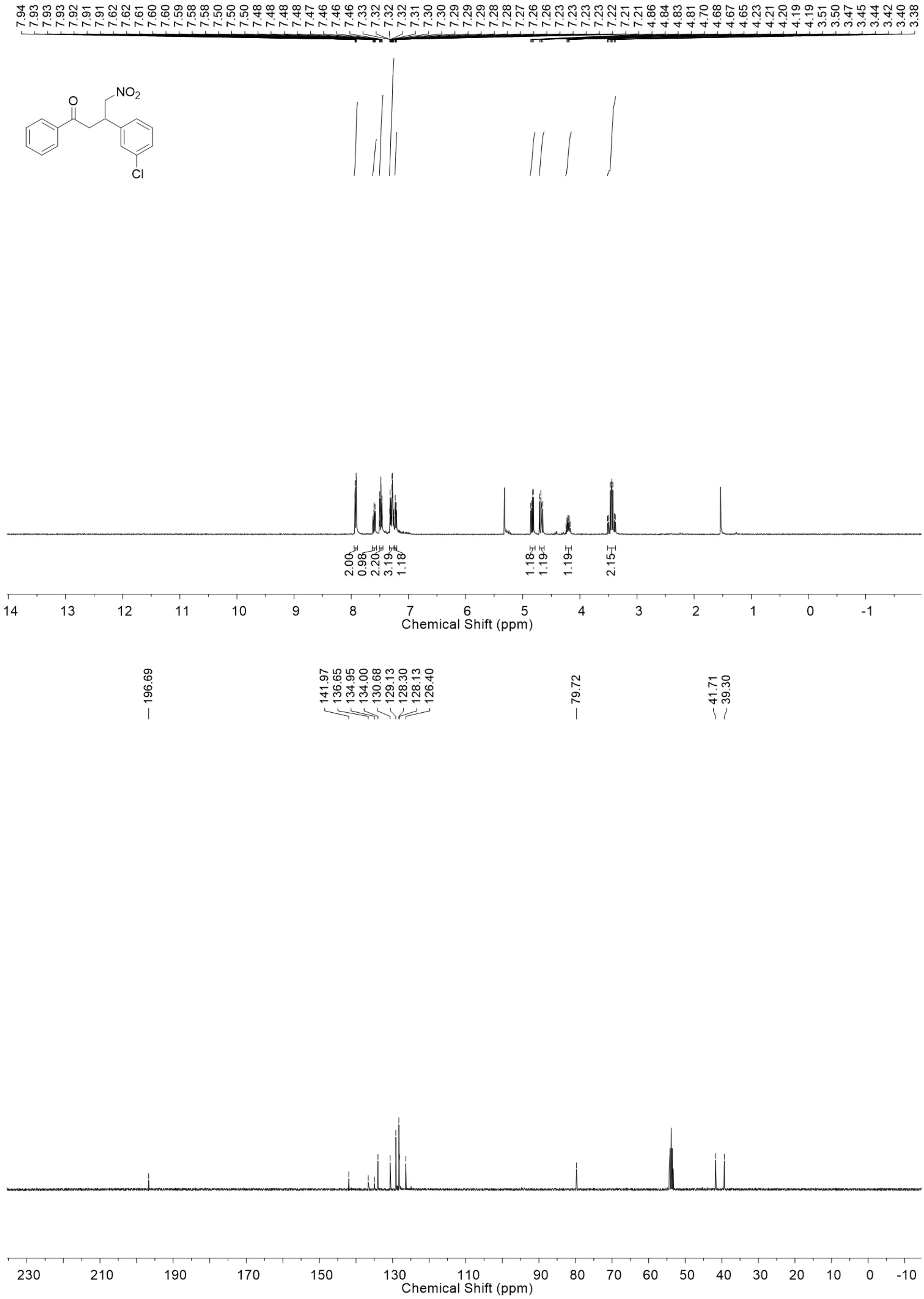

Figure S 39: NMR Spectra of 3-(3-chlorophenyl)-4-nitro-1-phenylbutan-1-one.

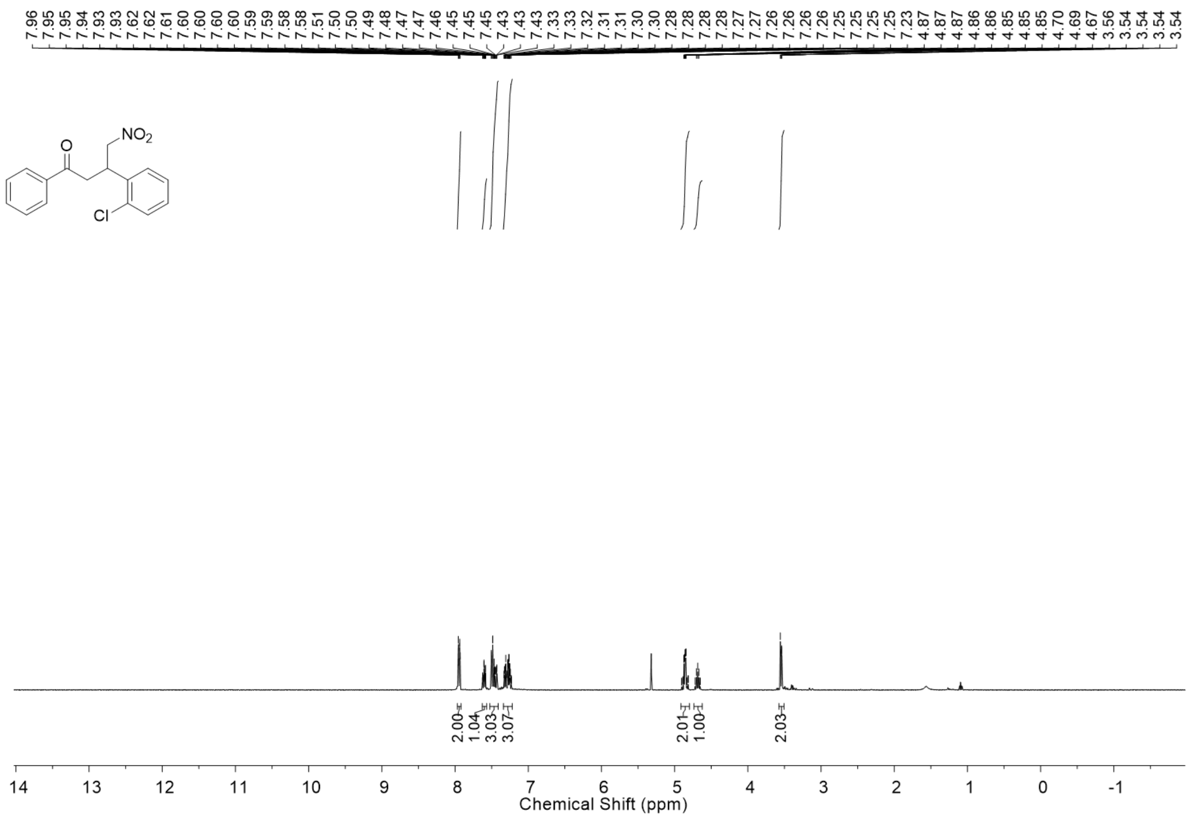

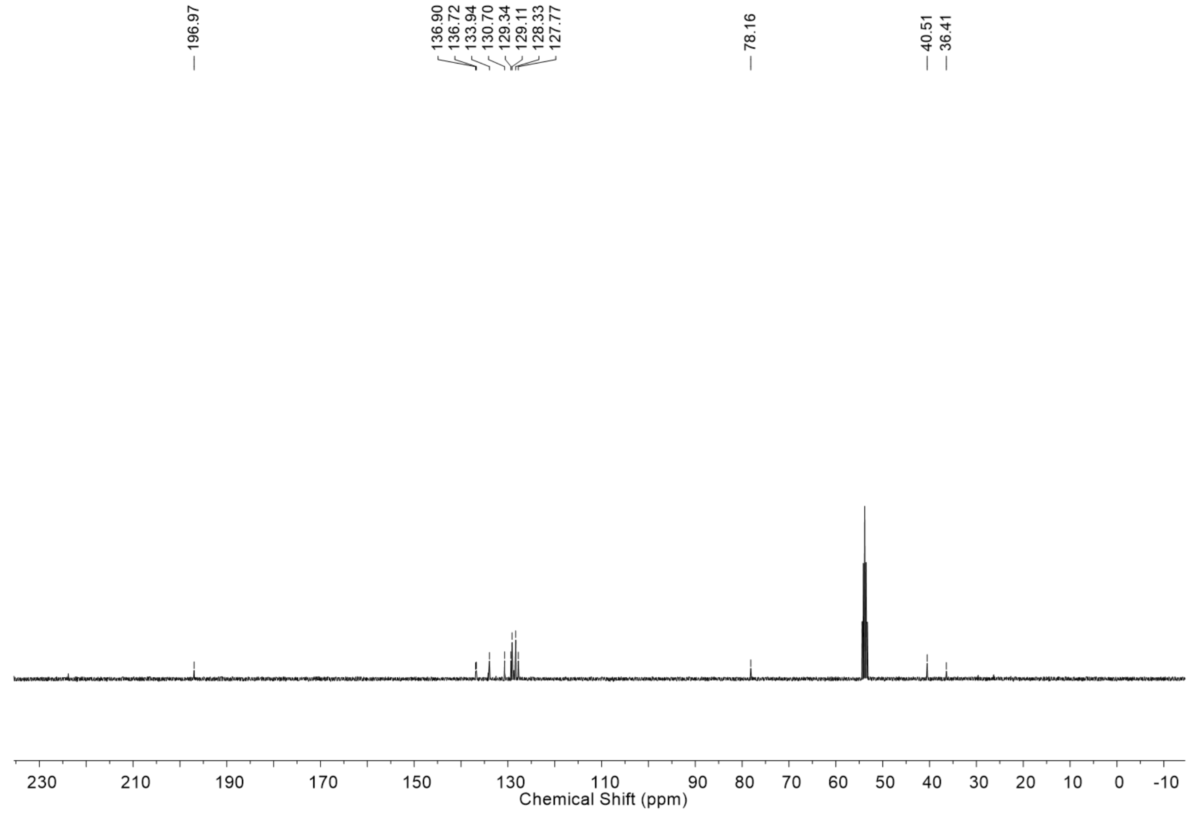

Figure S 40: NMR Spectra of 3-(2-chlorophenyl)-4-nitro-1-phenylbutan-1-one.

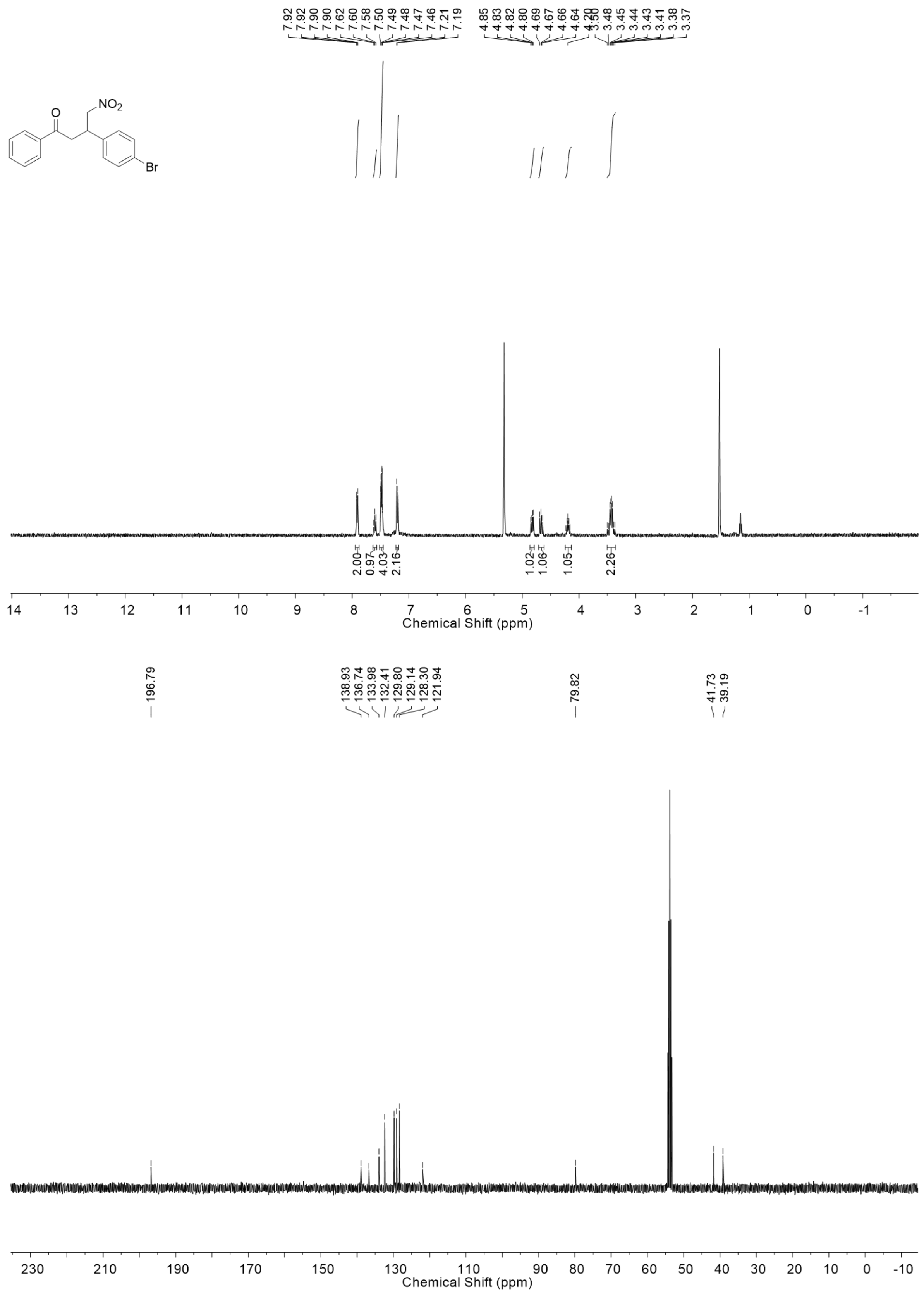

Figure S 41: NMR Spectra of 3-(4-bromophenyl)-4-nitro-1-phenylbutan-1-one.

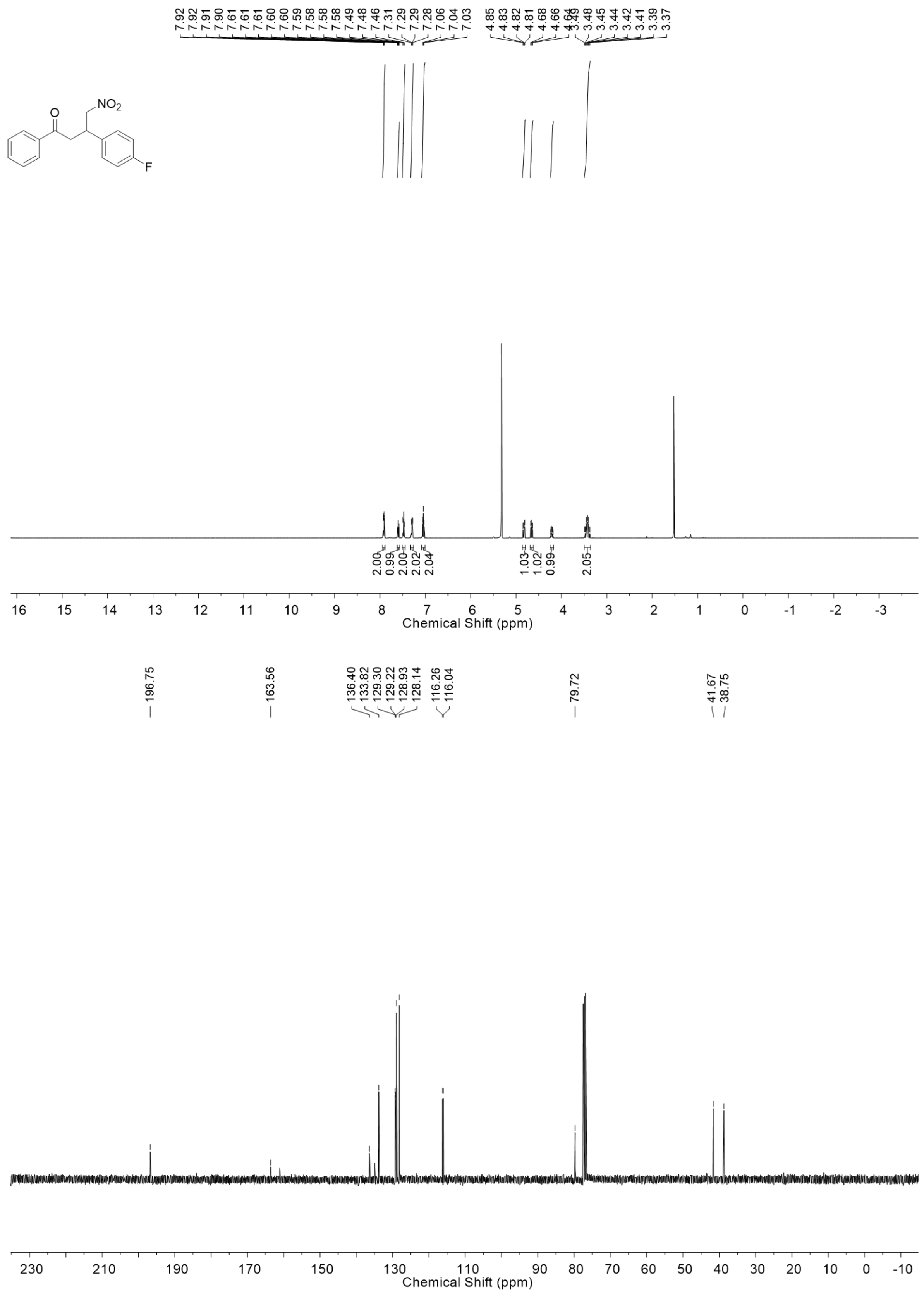

Figure S 42: NMR Spectra of 3-(4-fluorophenyl)-4-nitro-1-phenylbutan-1-one.

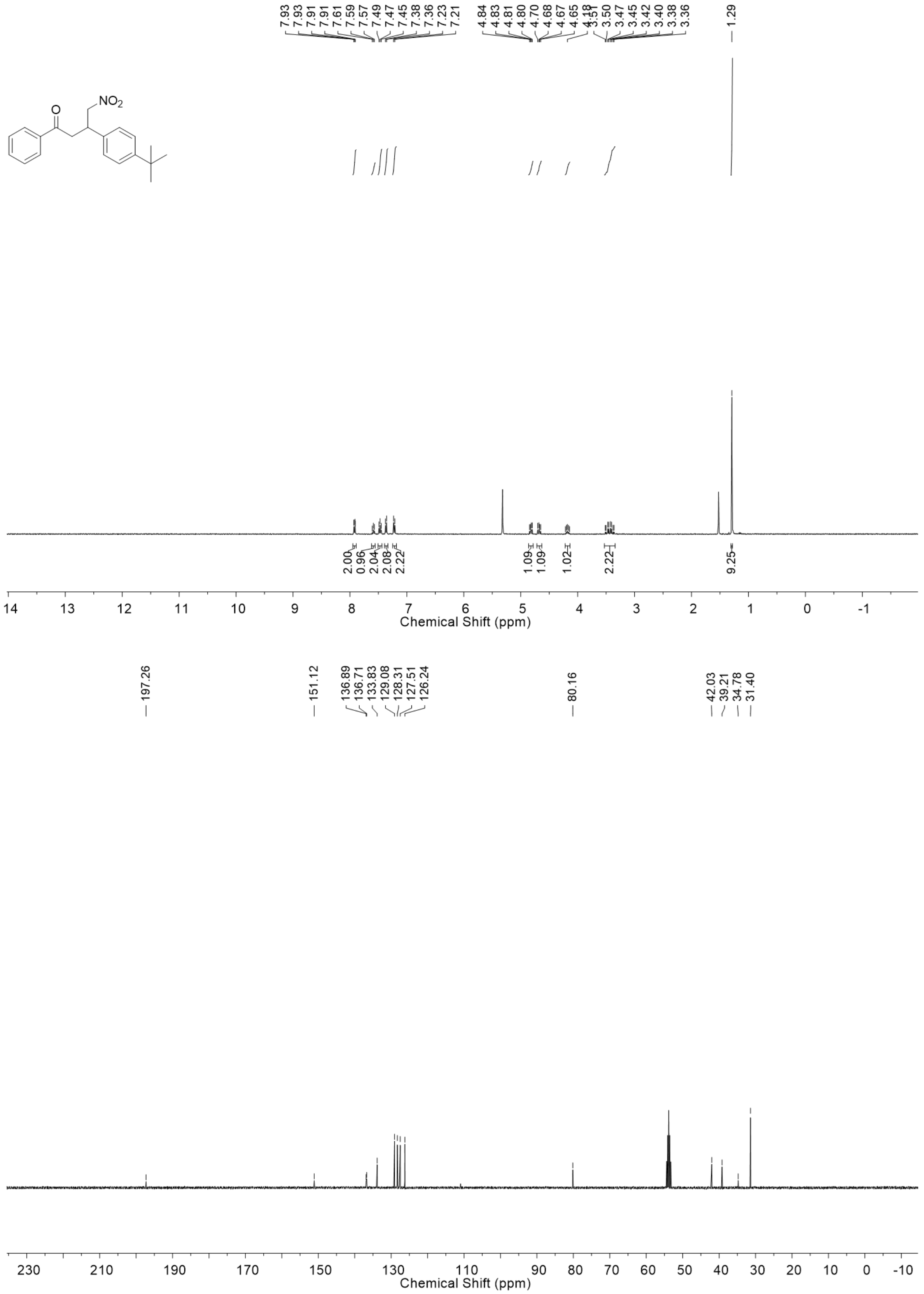

Figure S 43: NMR Spectra of 3-(4-(*tert*-butyl)phenyl)-4-nitro-1-phenylbutan-1-one.

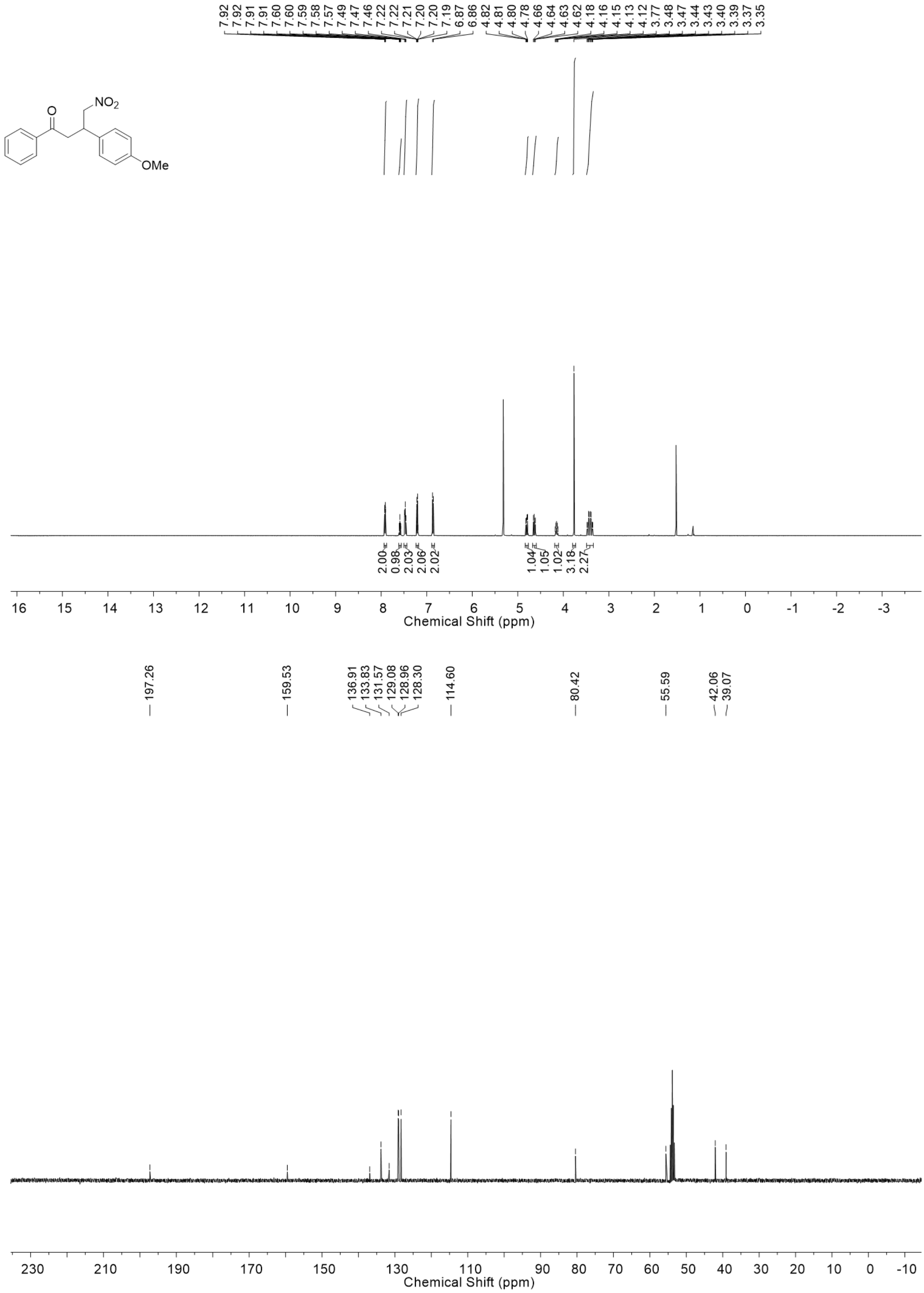

Figure S 44: NMR Spectra of 3-(4-methoxyphenyl)-4-nitro-1-phenylbutan-1-one.

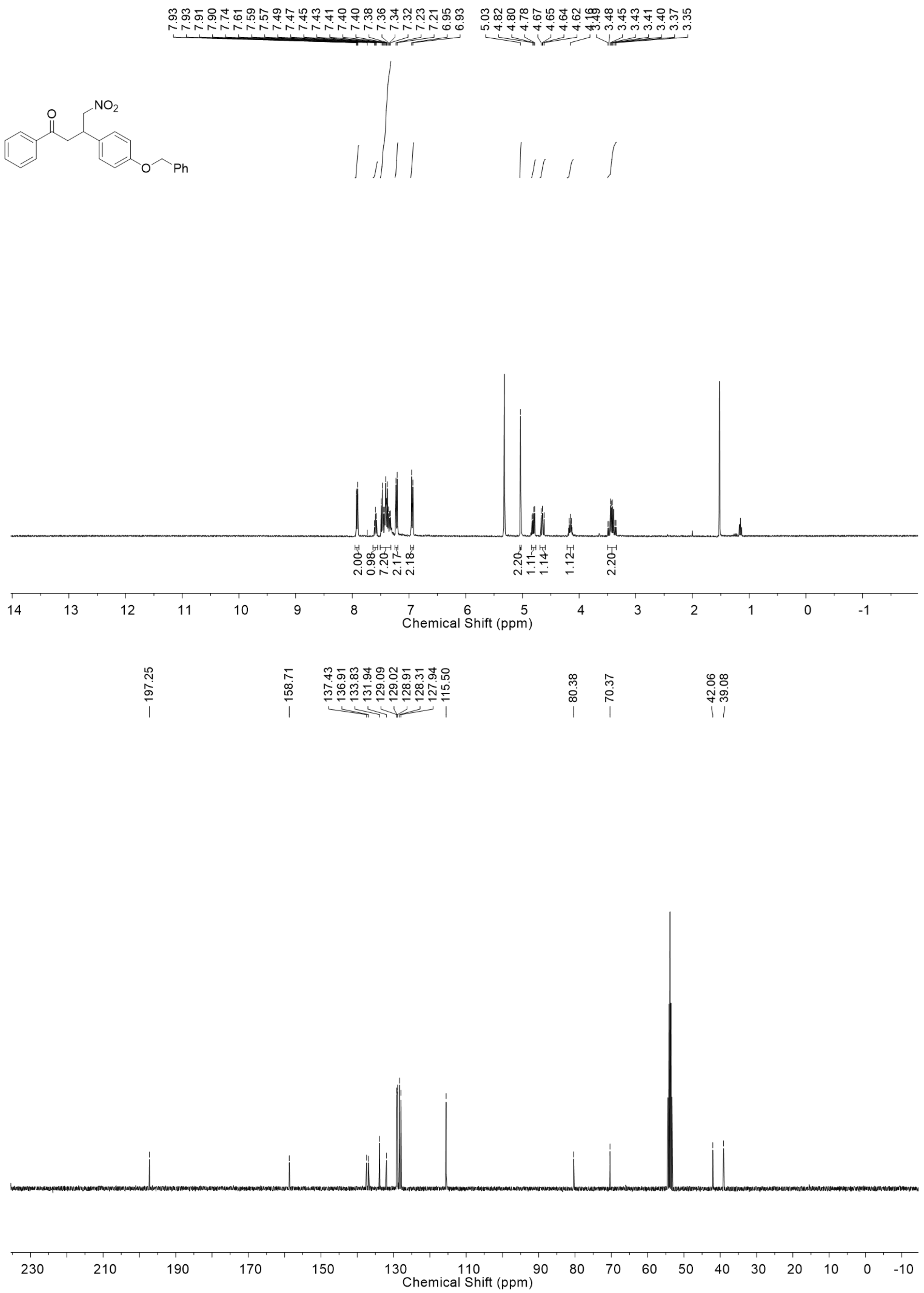

Figure S 45: NMR Spectra of 3-(4-(benzyloxy)phenyl)-4-nitro-1-phenylbutan-1-one.

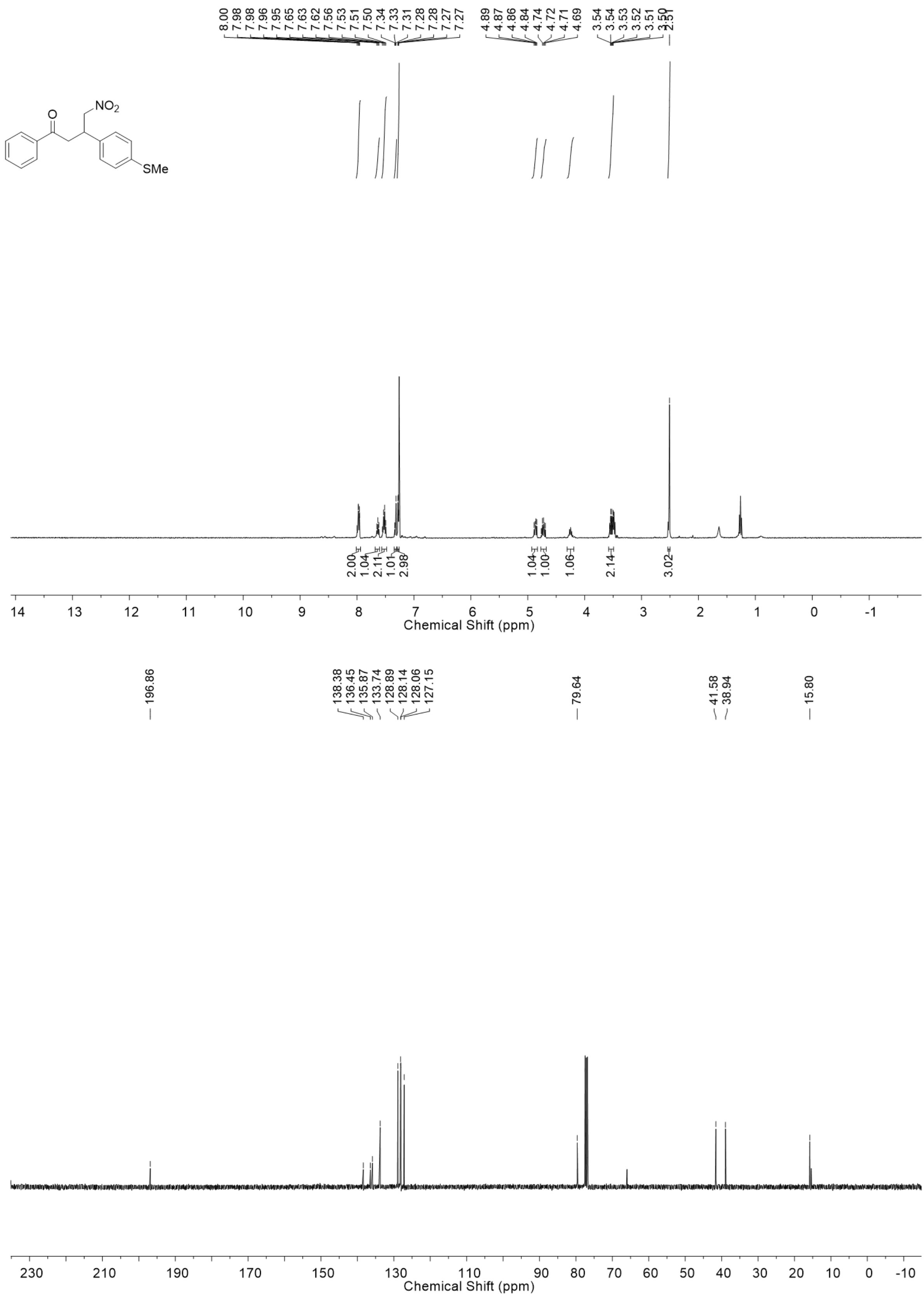

Figure S 46: NMR Spectra of 3-(4-(methylthio)phenyl)-4-nitro-1-phenylbutan-1-one.

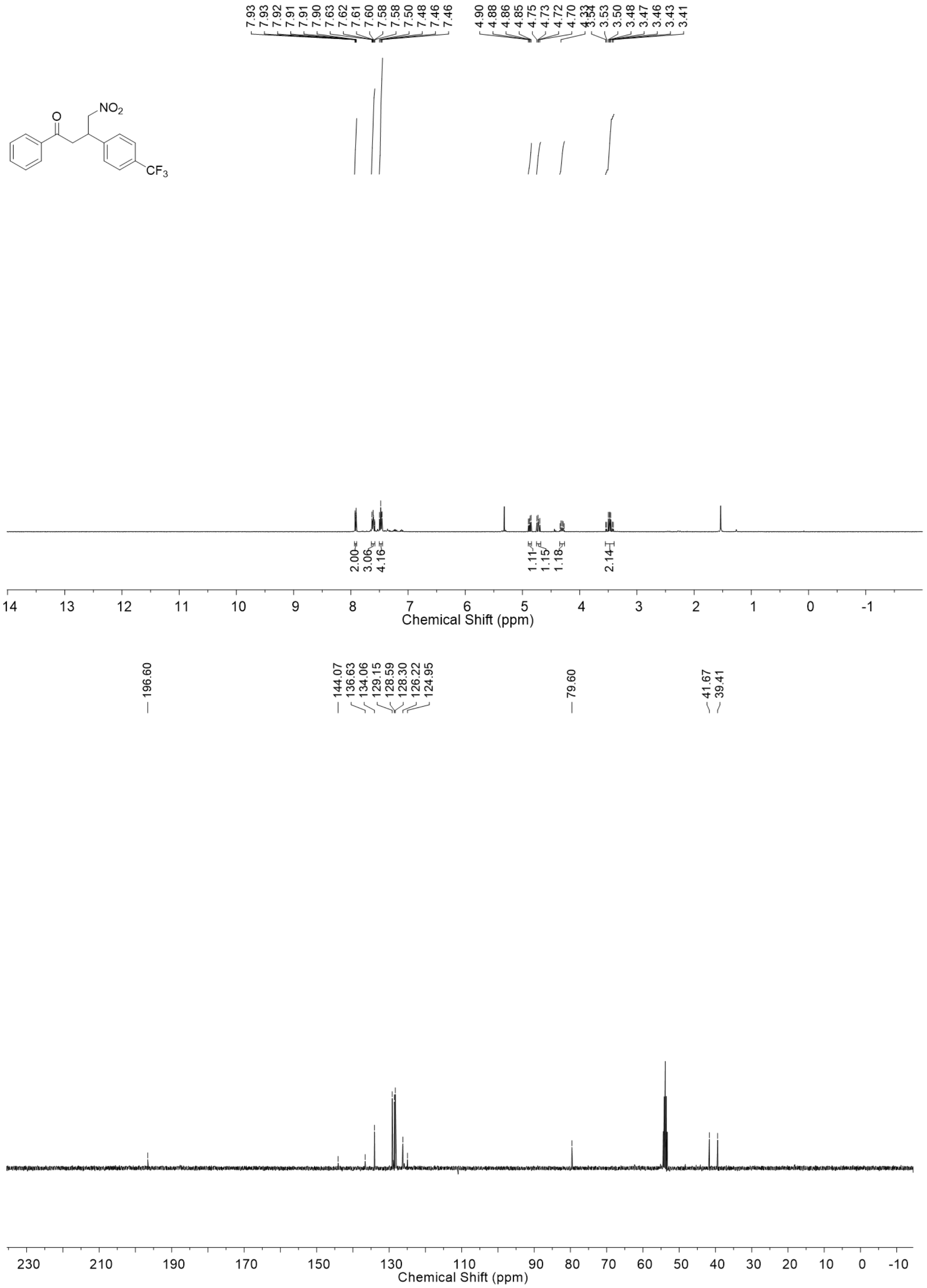

Figure S 47: NMR Spectra of 4-nitro-1-phenyl-3-(4-(trifluoromethyl)phenyl)butan-1-one.

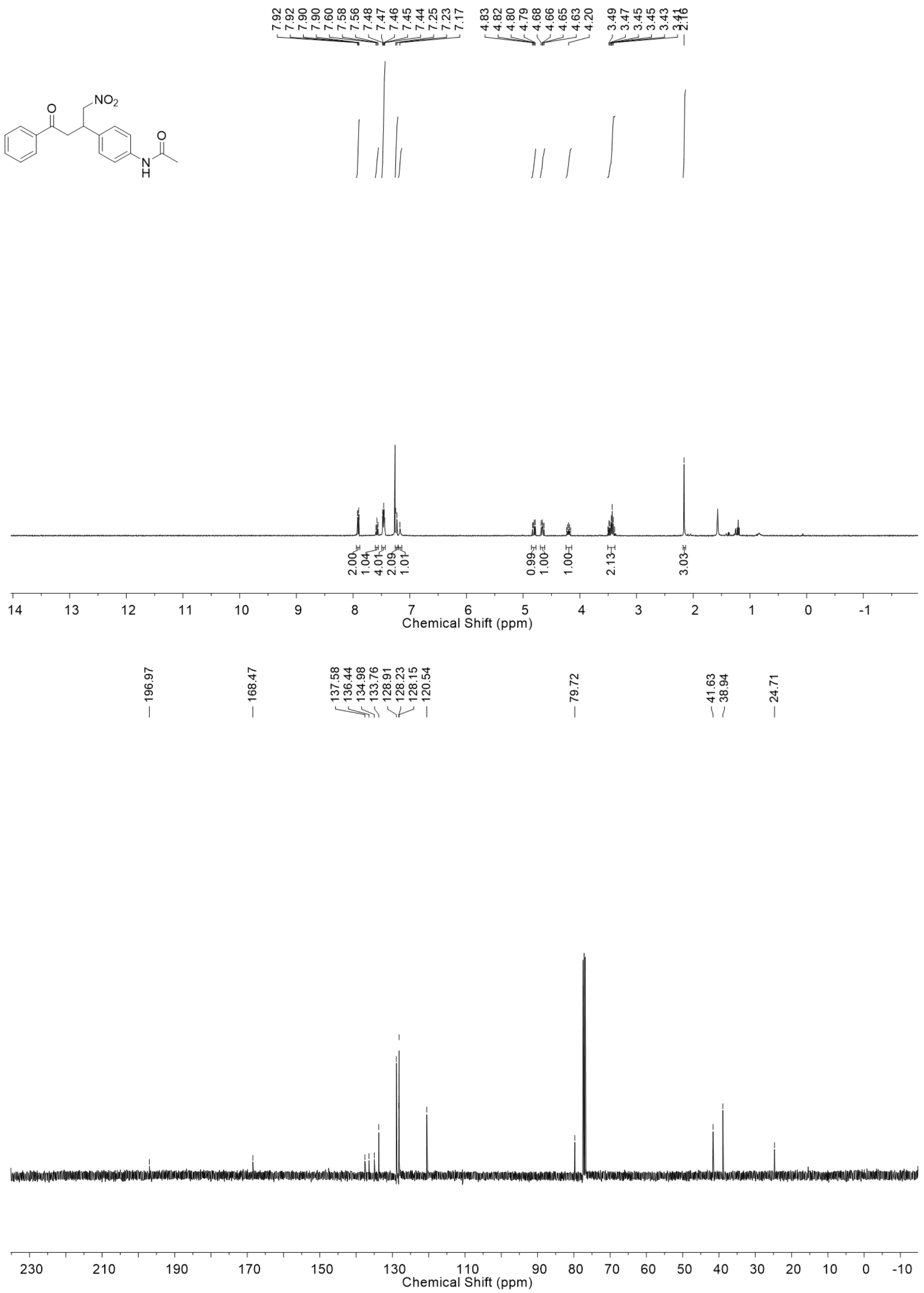

Figure S 48: NMR Spectra of *N*-(4-(1-nitro-4-oxo-4-phenylbutan-2-yl)phenyl)acetamide.

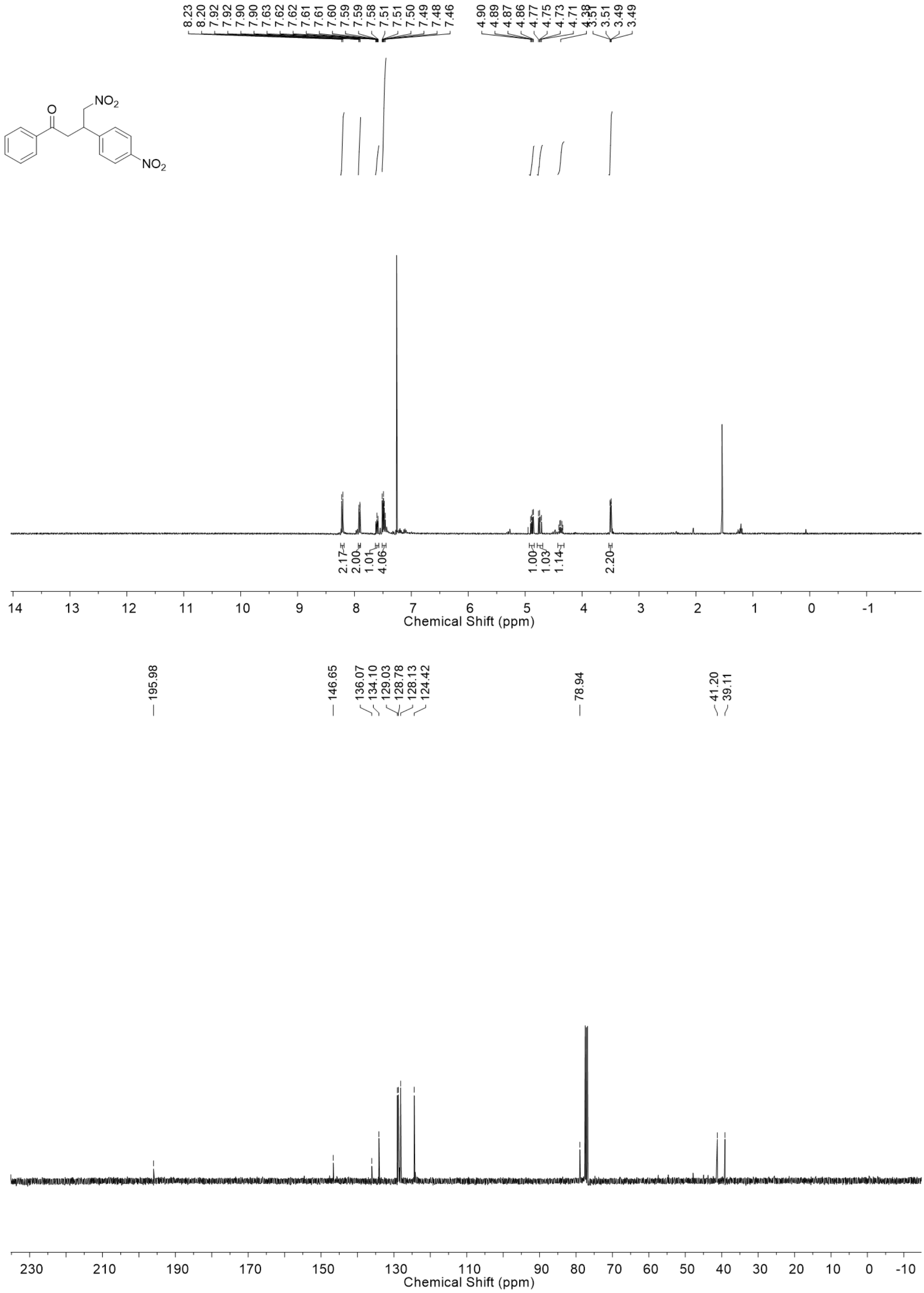

Figure S 49: NMR Spectra of 4-nitro-3-(4-nitrophenyl)-1-phenylbutan-1-one.

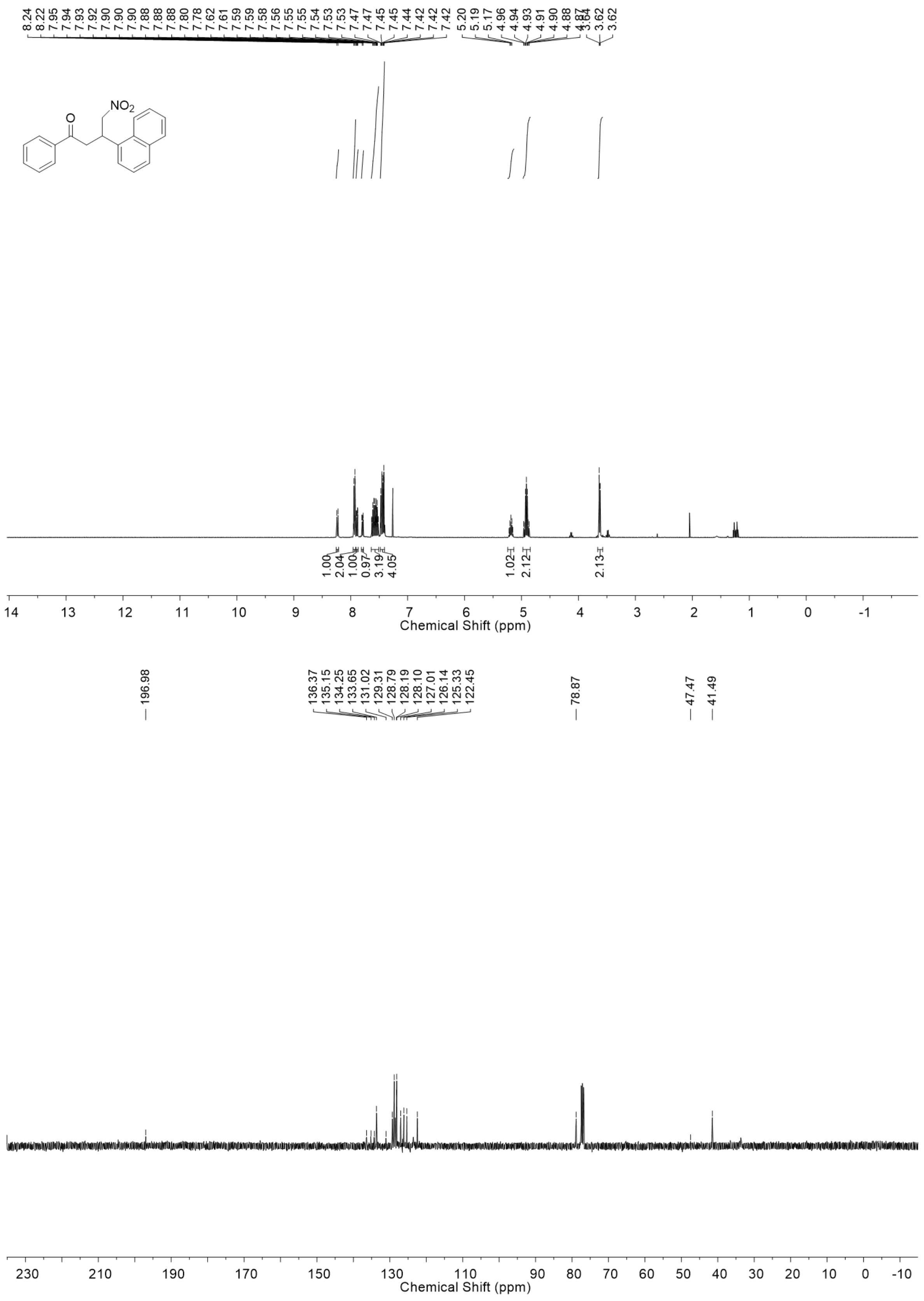

Figure S 50: NMR Spectra of 3-(naphthalen-1-yl)-4-nitro-1-phenylbutan-1-one.

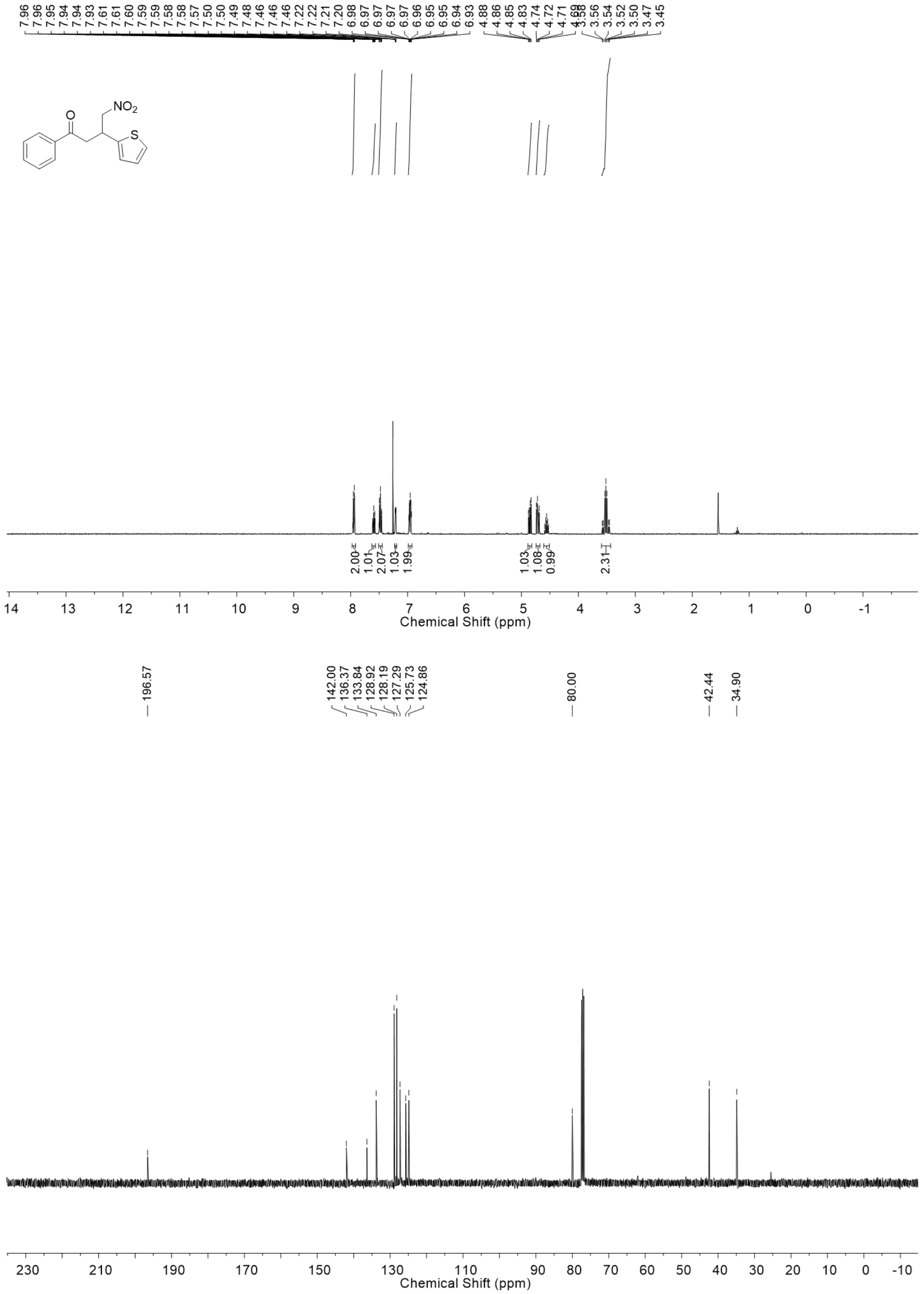

Figure S 51: NMR Spectra of 4-nitro-1-phenyl-3-(thiophen-2-yl)butan-1-one.

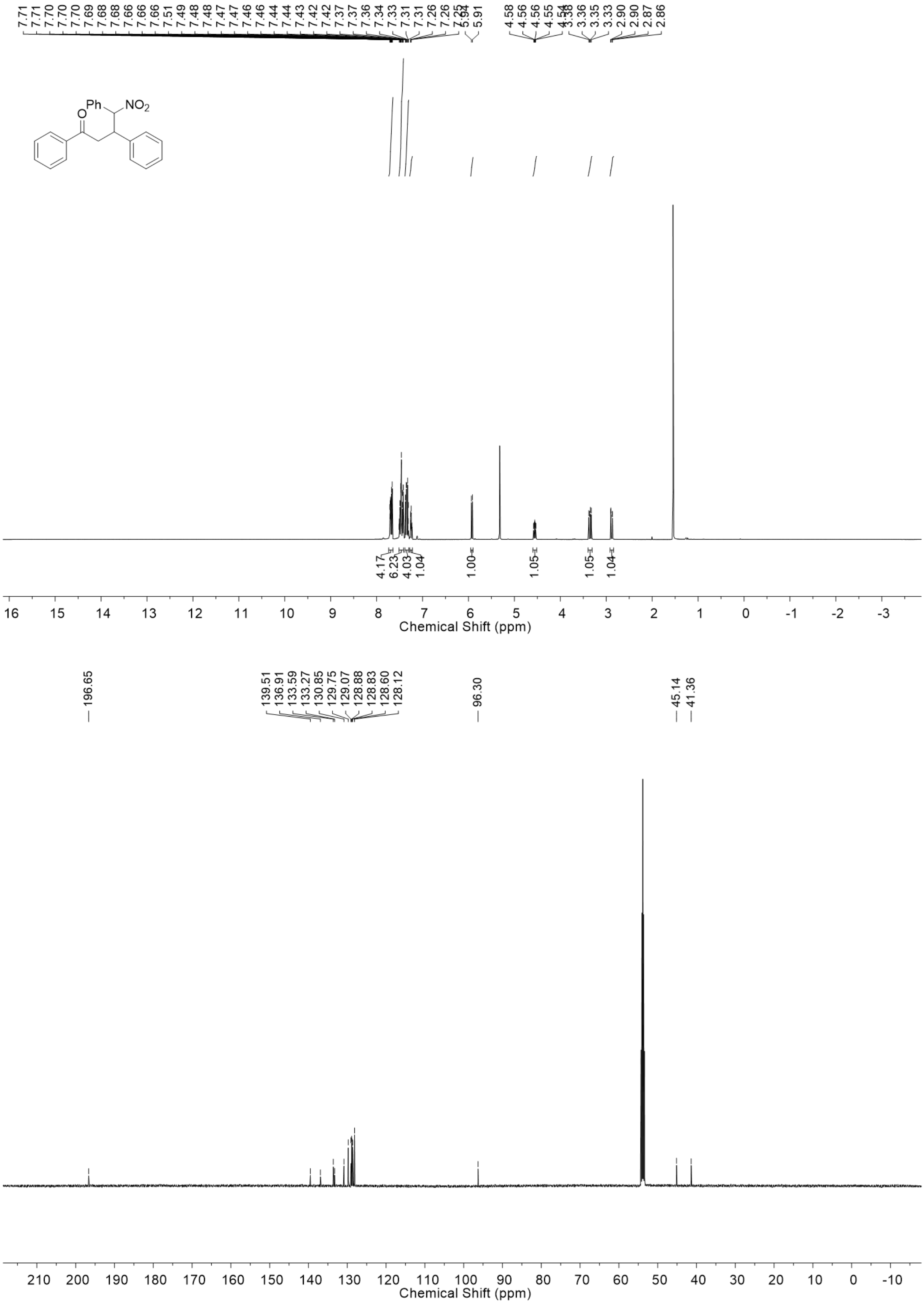

Figure S 52: NMR Spectra of 4-nitro-1,3,4-triphenylbutan-1-one.

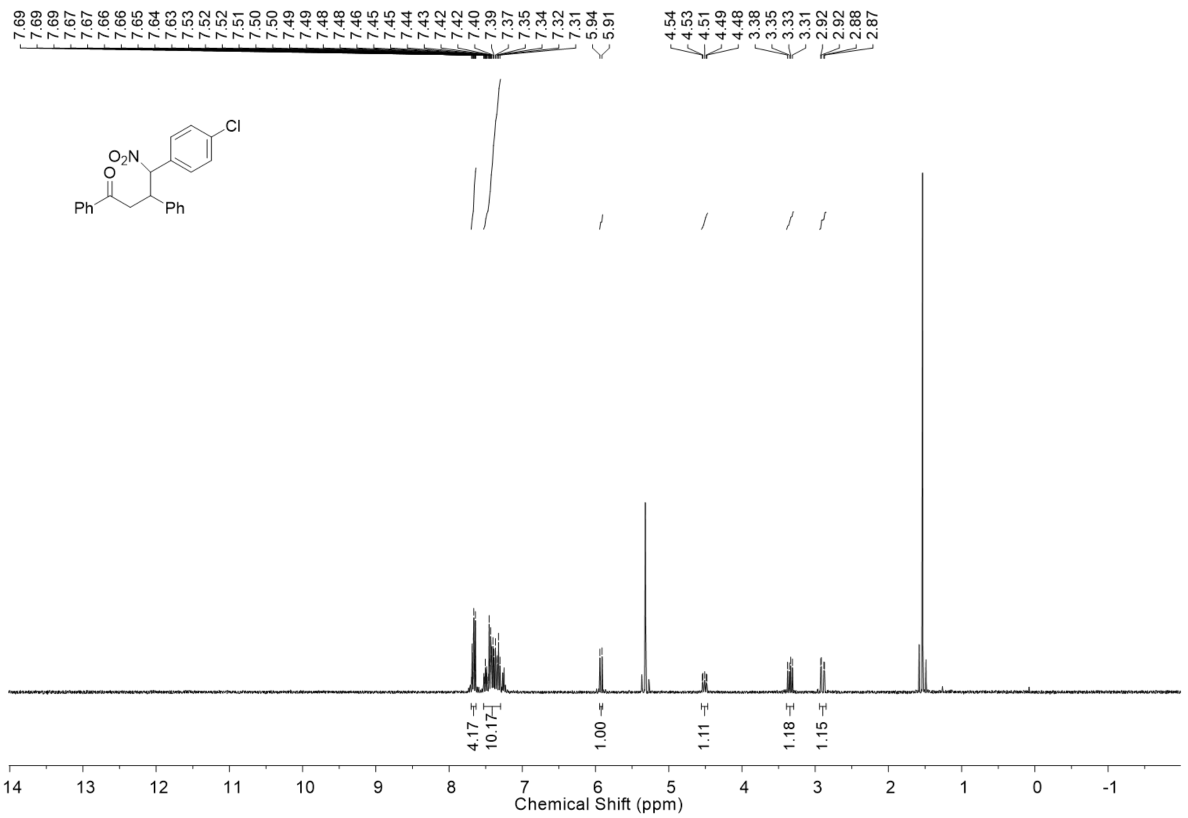

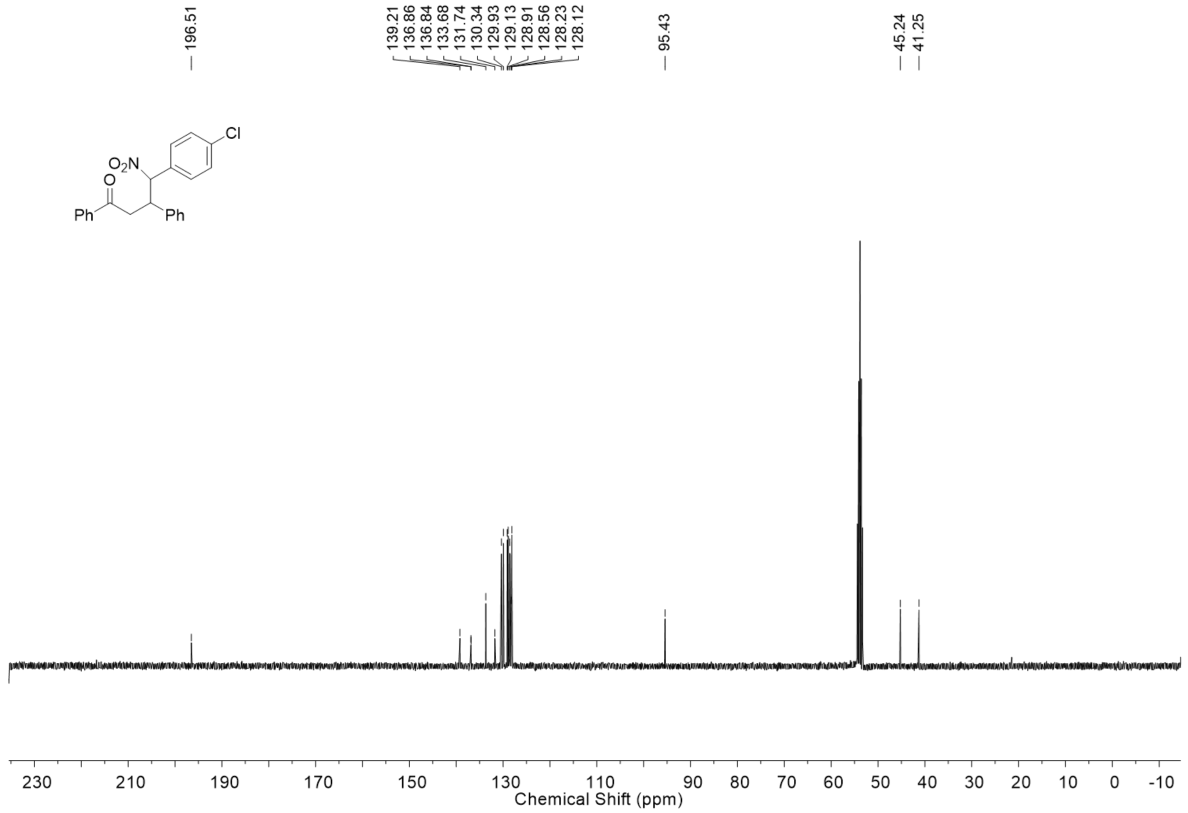

Figure S 53: NMR Spectra of 4-(4-chlorophenyl)-4-nitro-1,3-diphenylbutan-1-one.

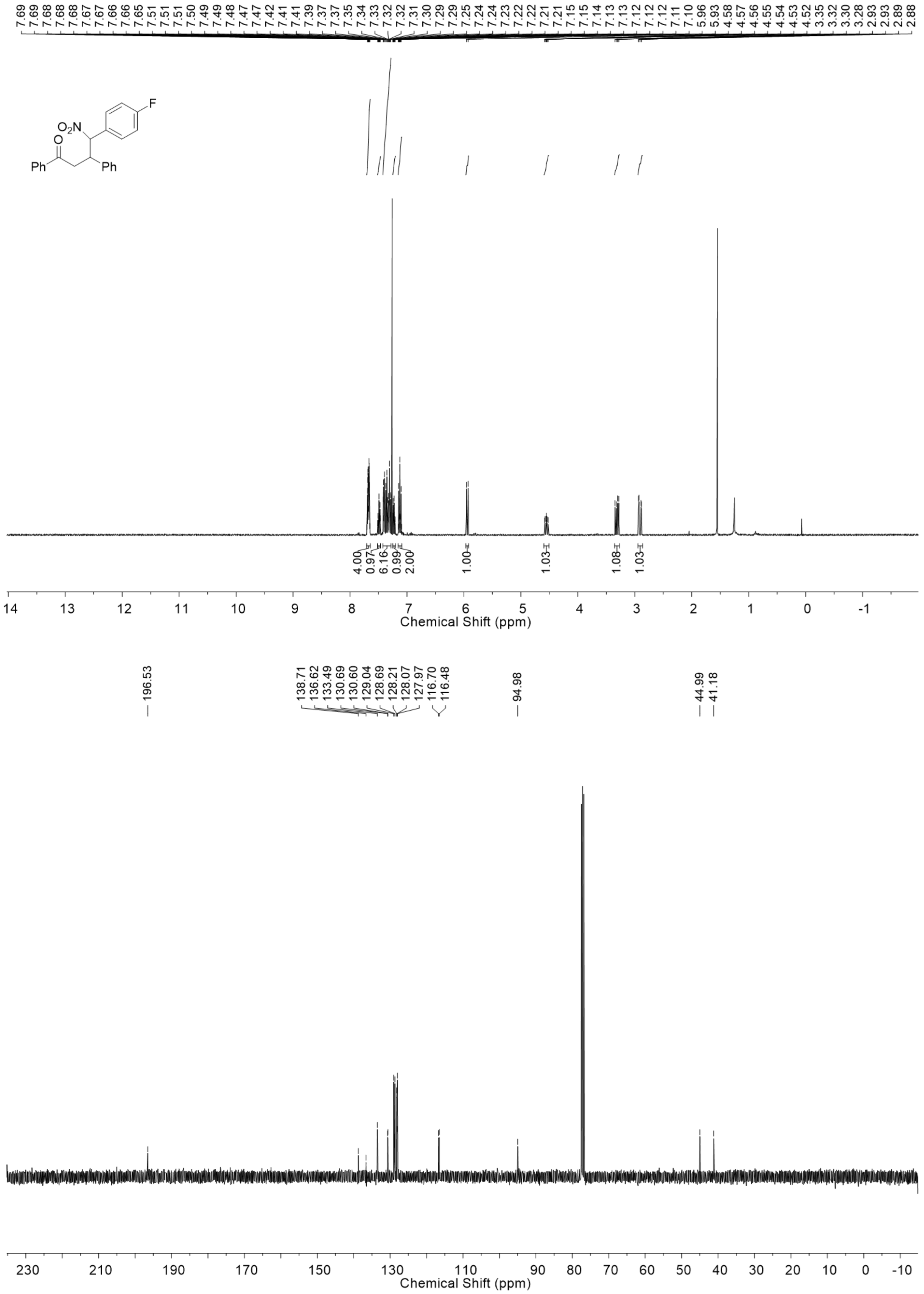

Figure S 54: NMR Spectra of 4-(4-fluorophenyl)-4-nitro-1,3-diphenylbutan-1-one.

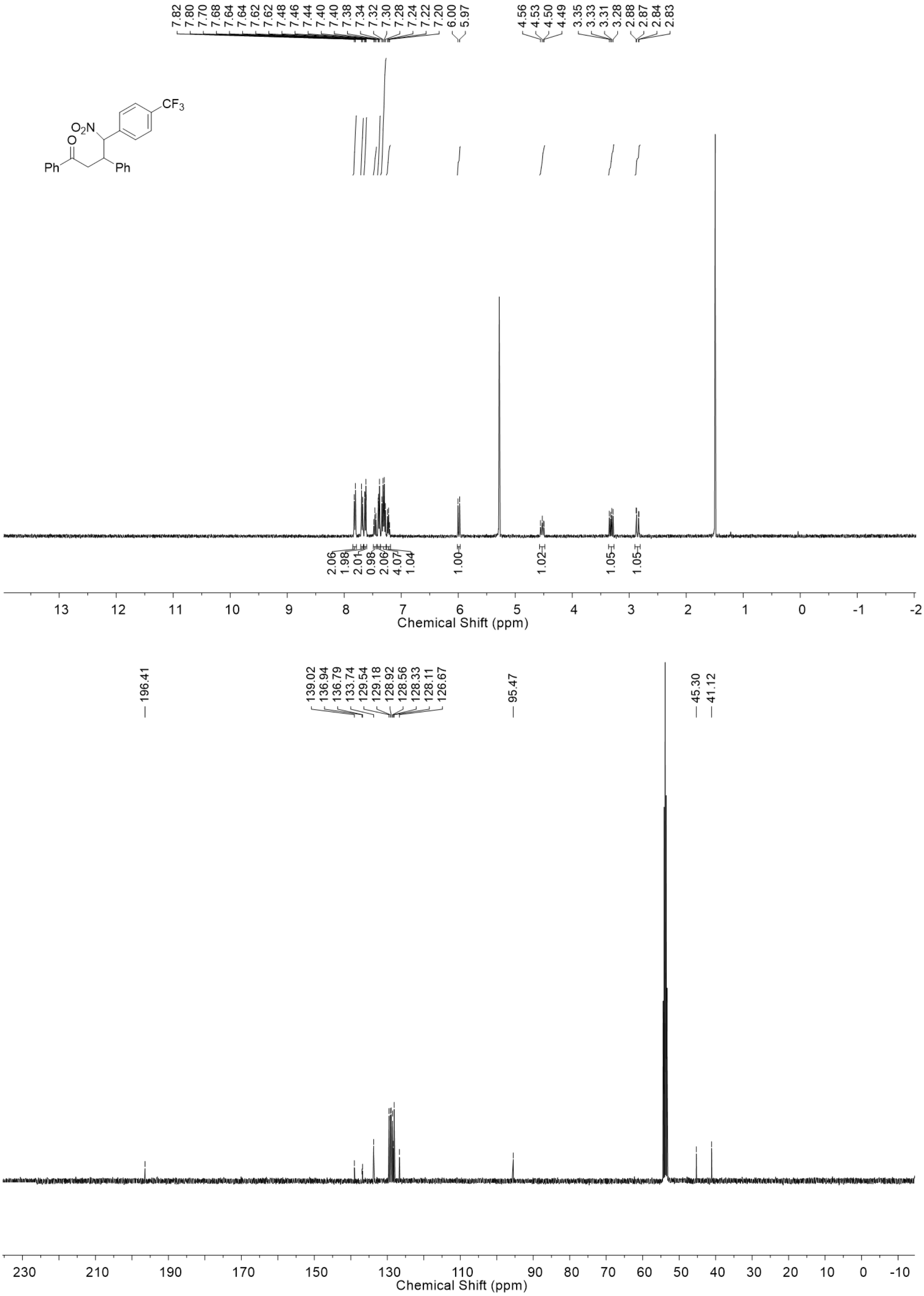

Figure S 55: NMR Spectra of 4-nitro-1,3-diphenyl-4-(4-(trifluoromethyl)phenyl)butan-1-one.

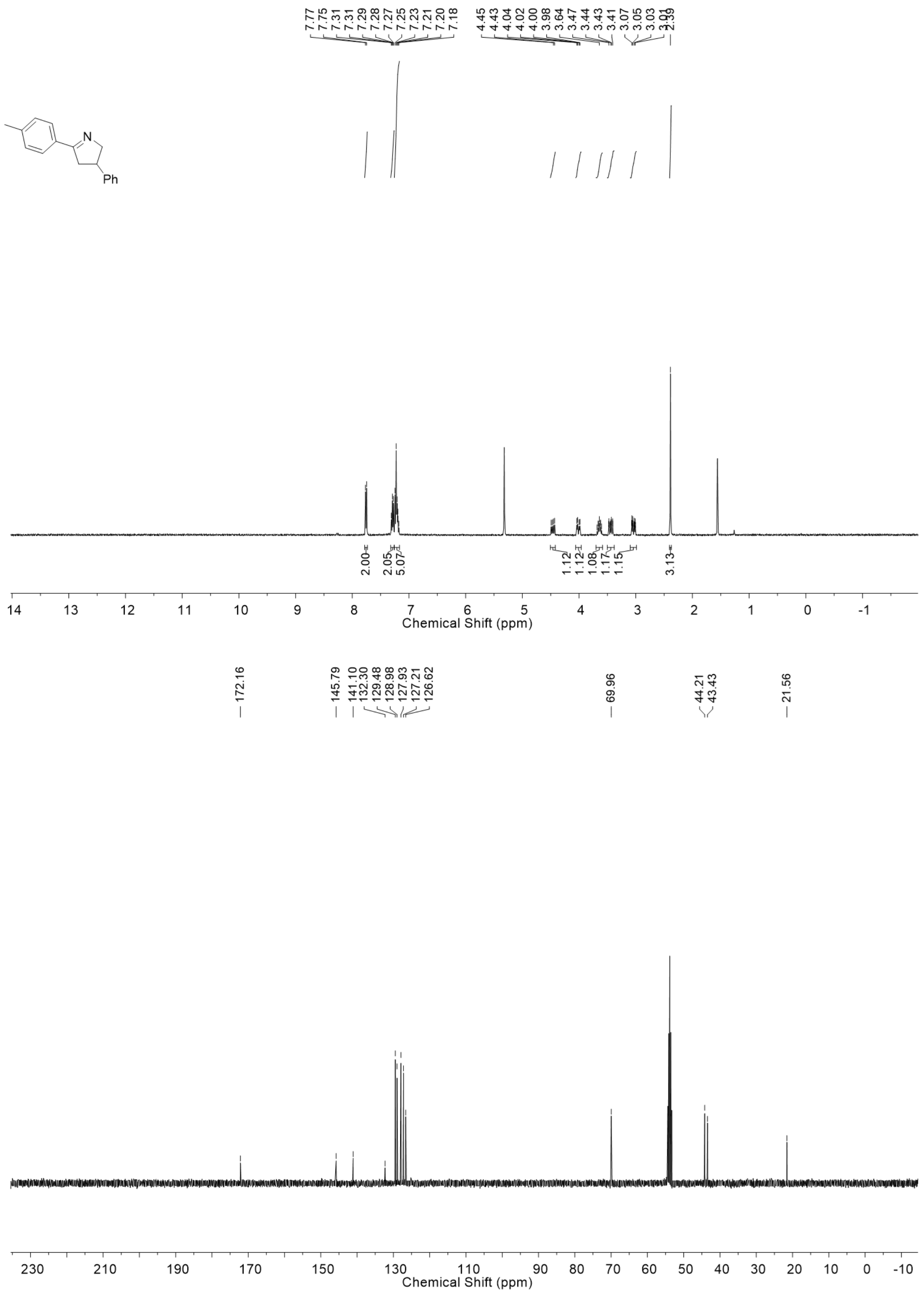

Figure S 56: NMR Spectra of compound (**1**).

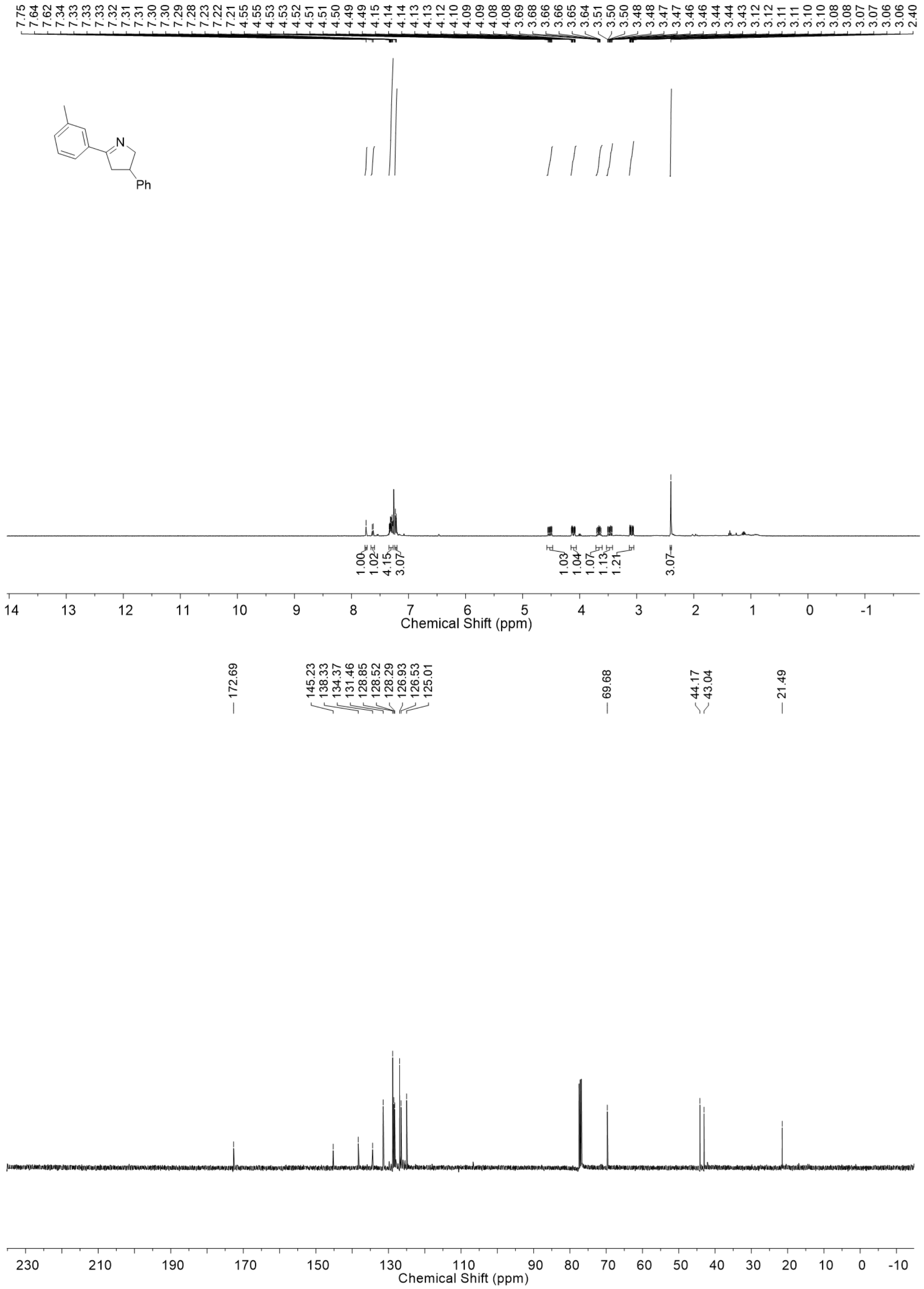

Figure S 57: NMR Spectra of compound (**2**).

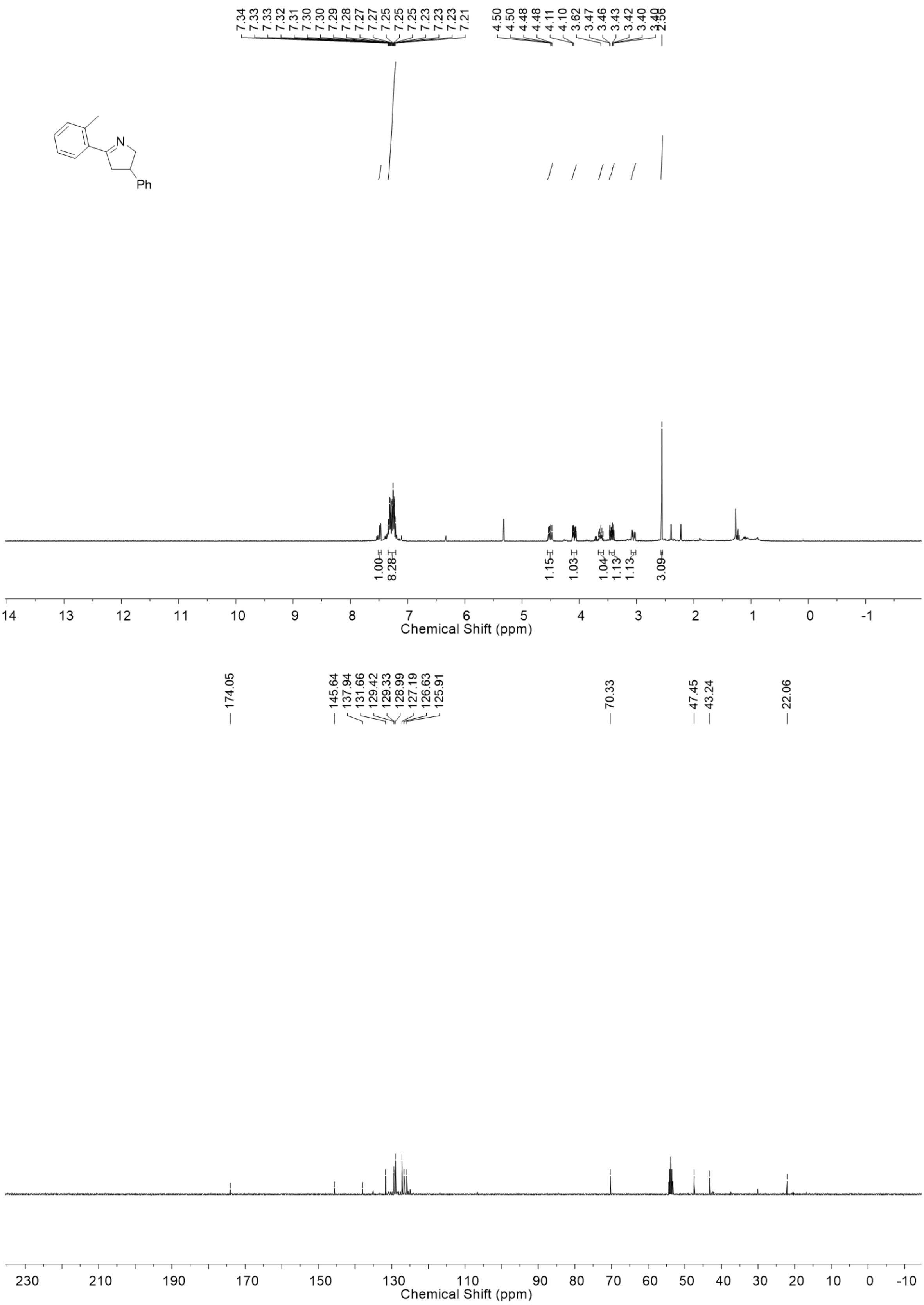

Figure S 58: NMR Spectra of compound (**3**).

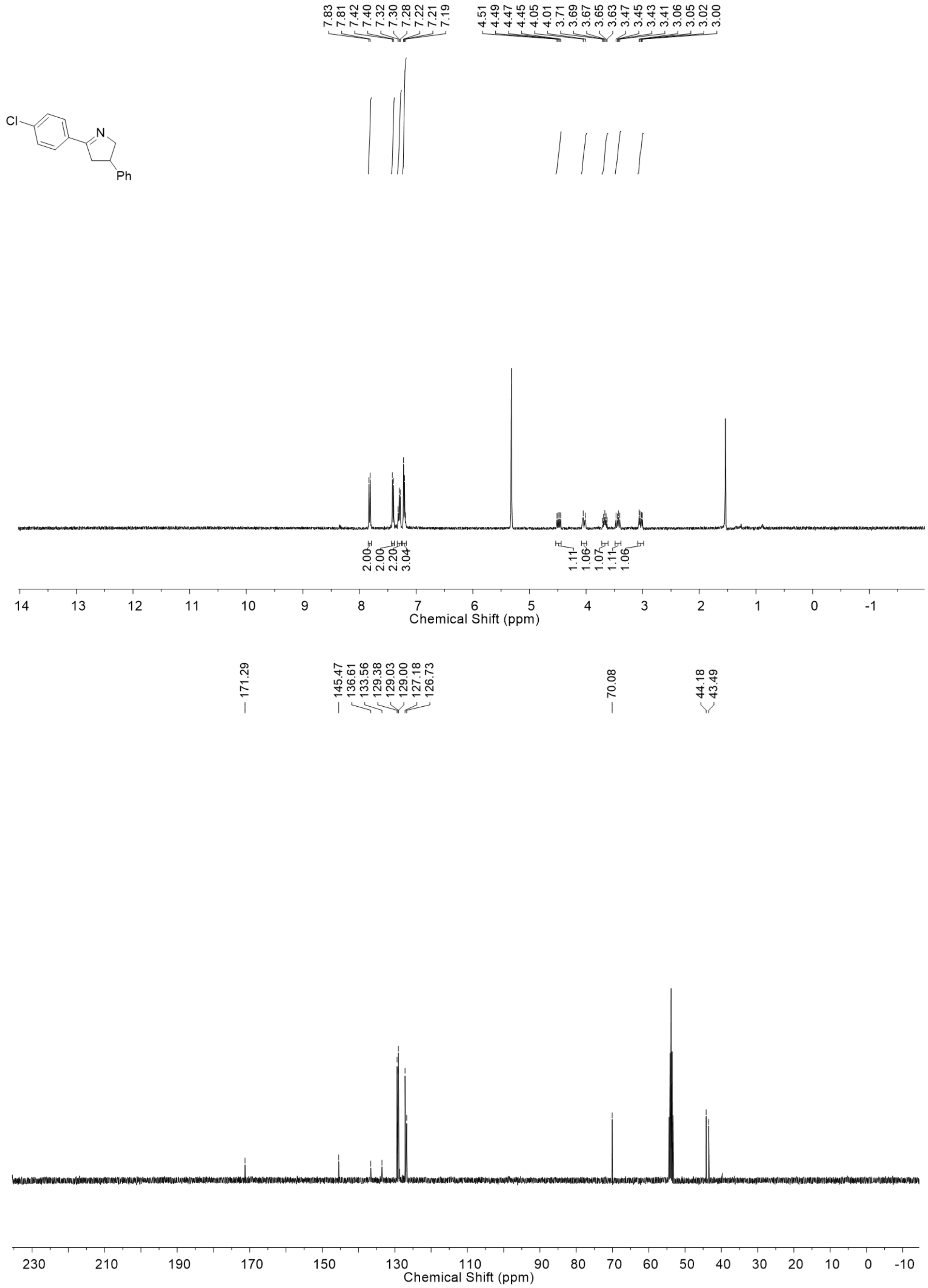

Figure S 59: NMR Spectra of compound (**4**).

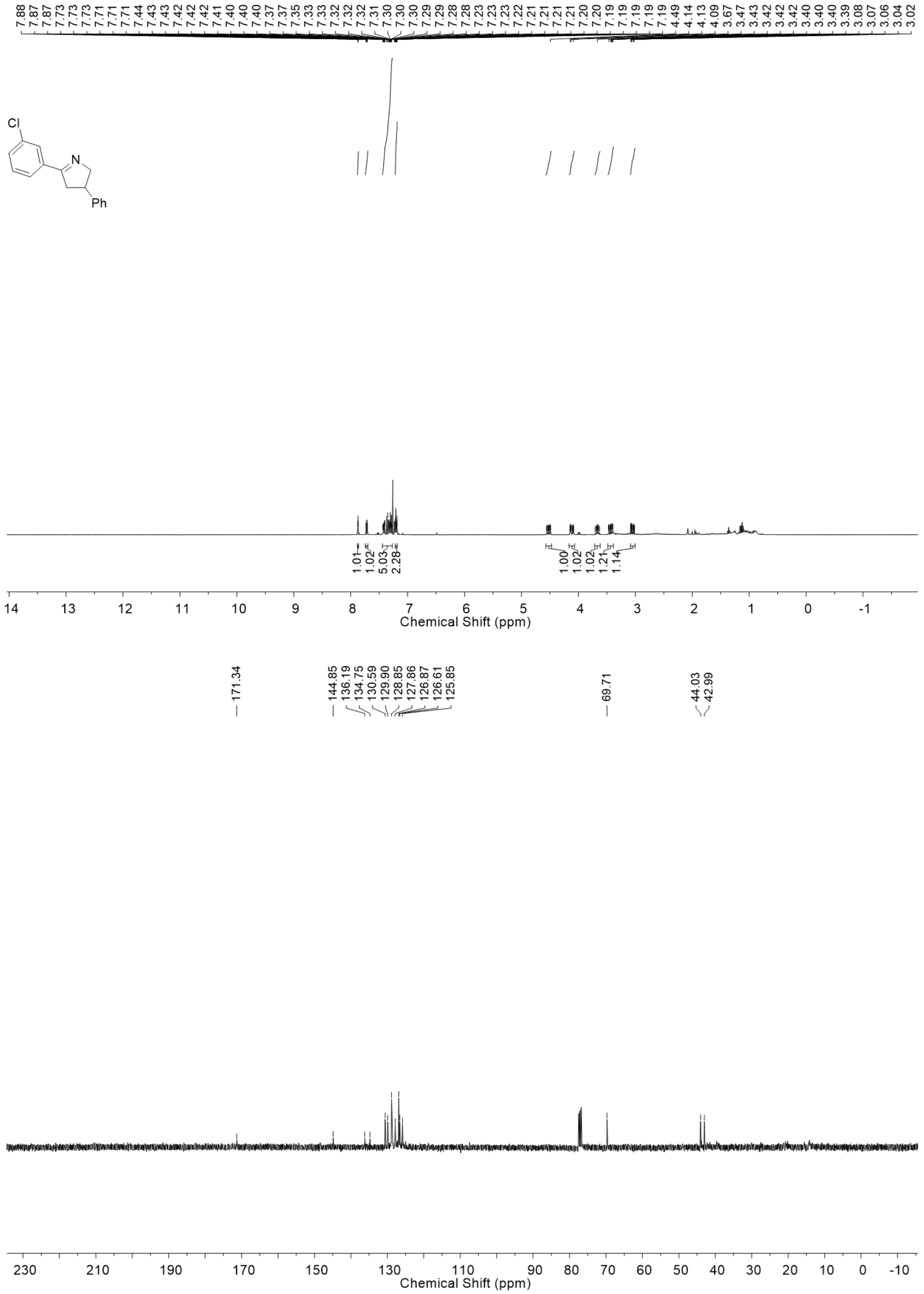

Figure S 60: NMR Spectra of compound (**5**).

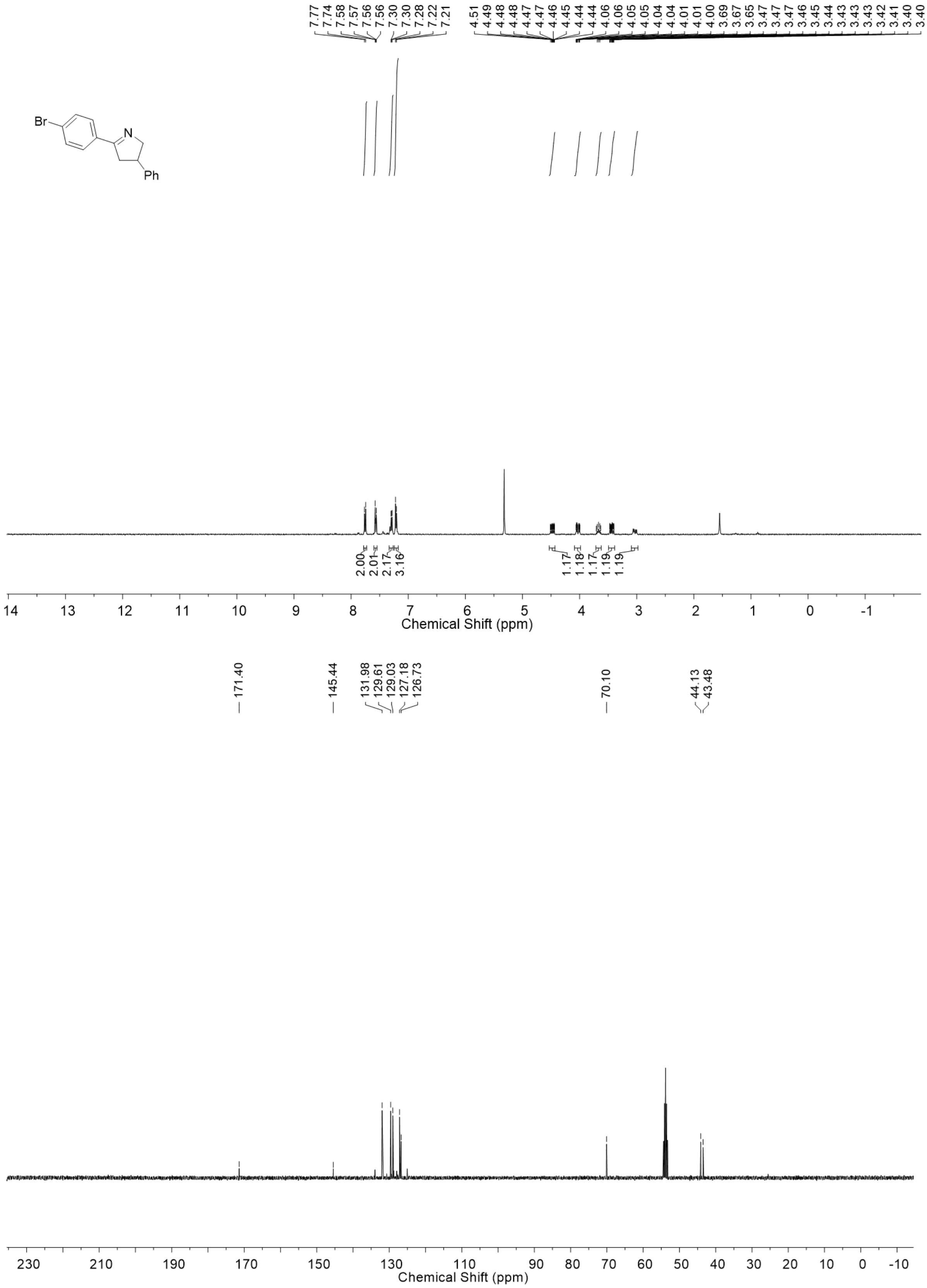

Figure S 61: NMR Spectra of compound (**6**).

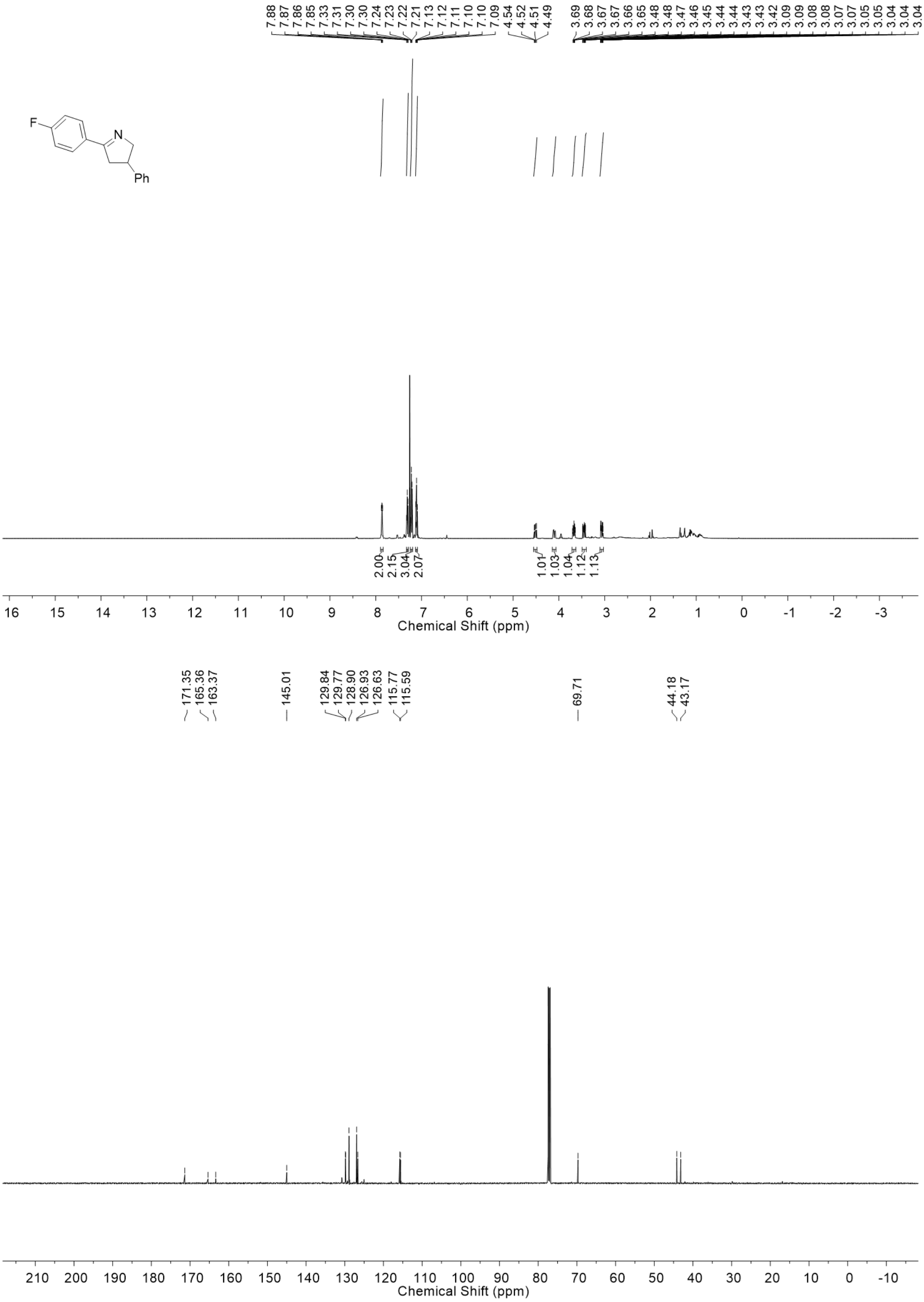

Figure S 62: NMR Spectra of compound (**7**).

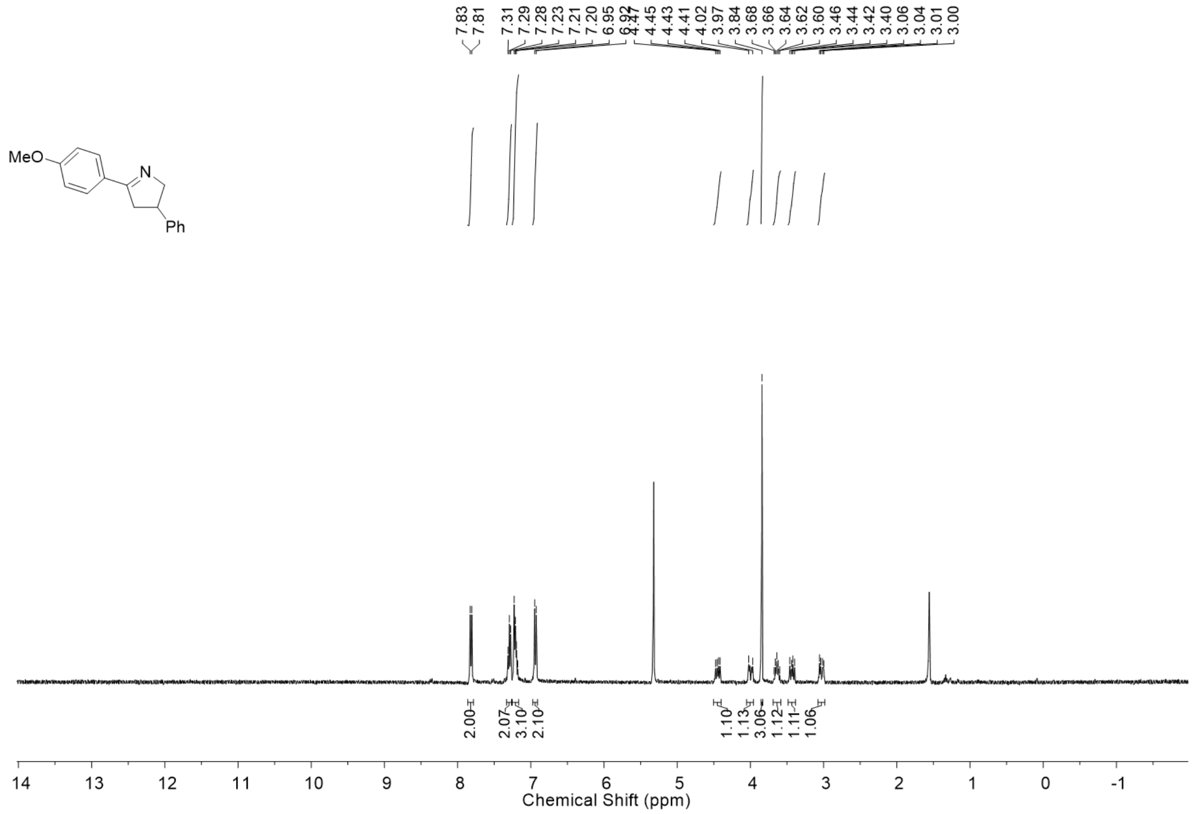

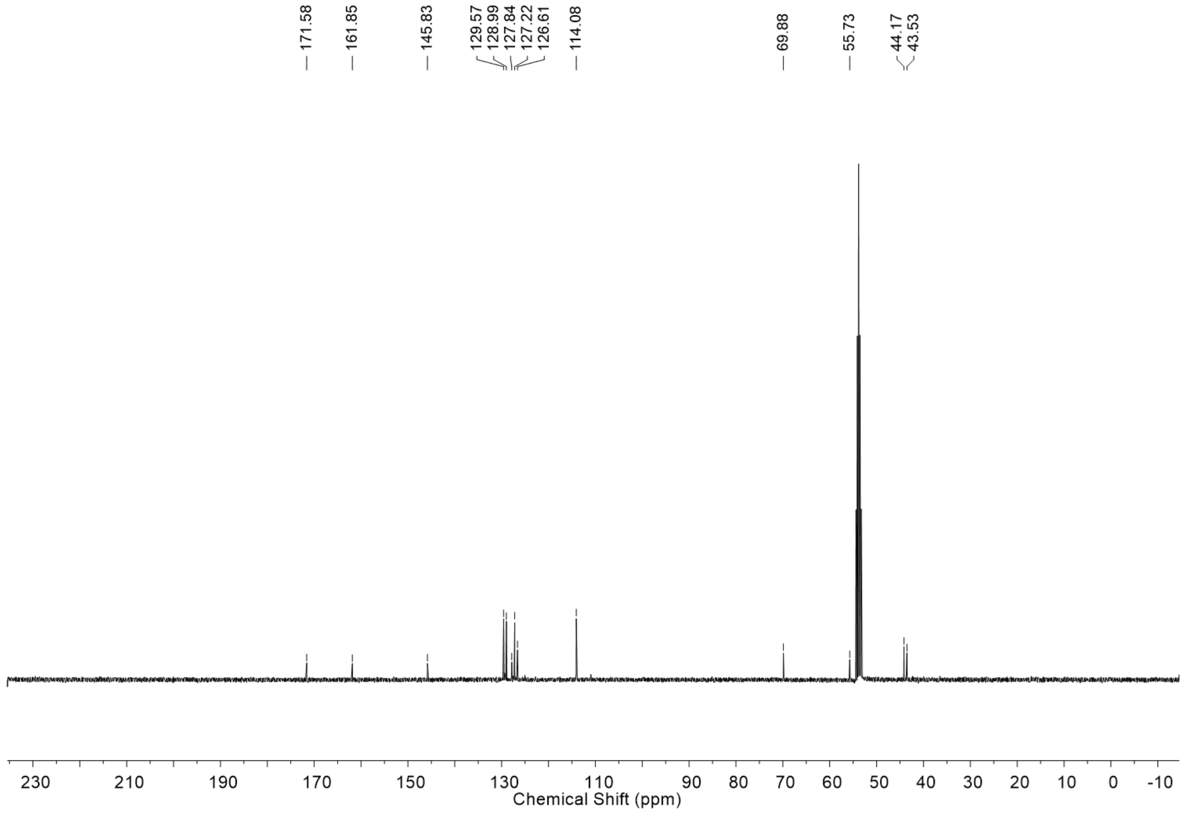

Figure S 63: NMR Spectra of compound (**8**).

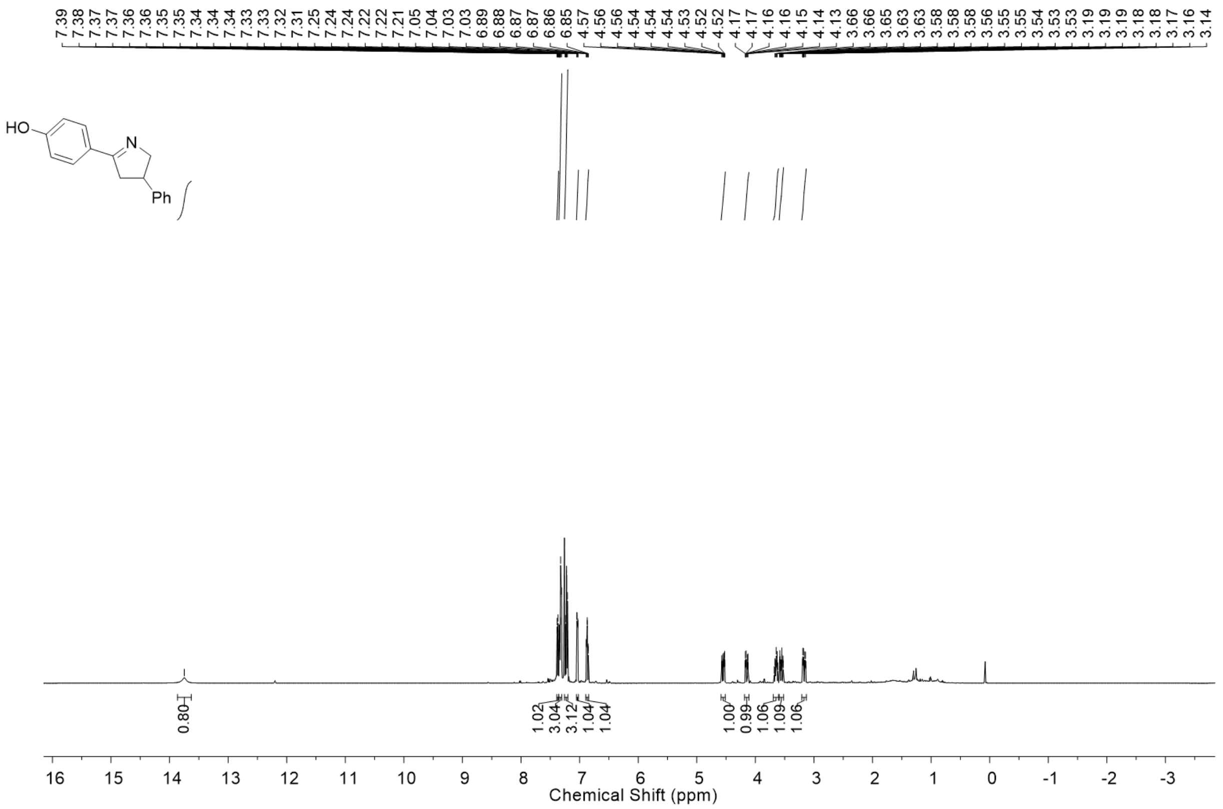

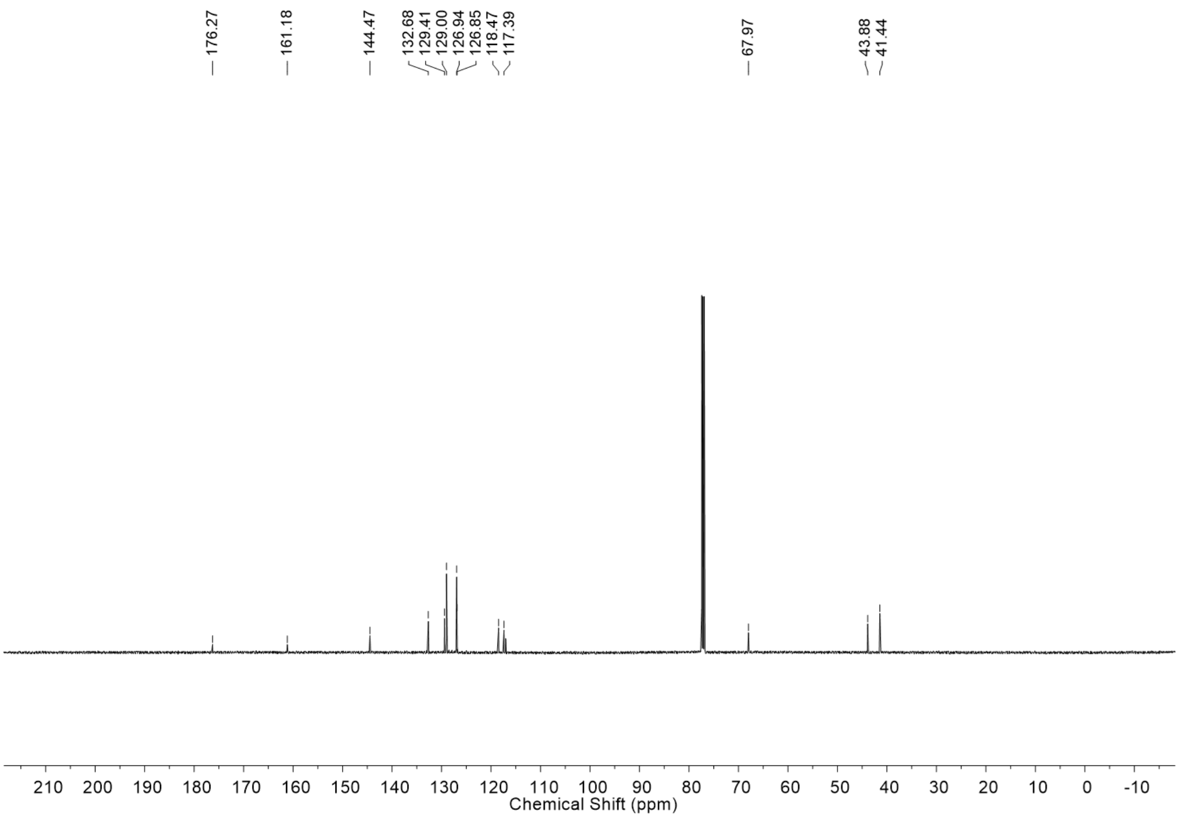

Figure S 64: NMR Spectra of compound (**9**).

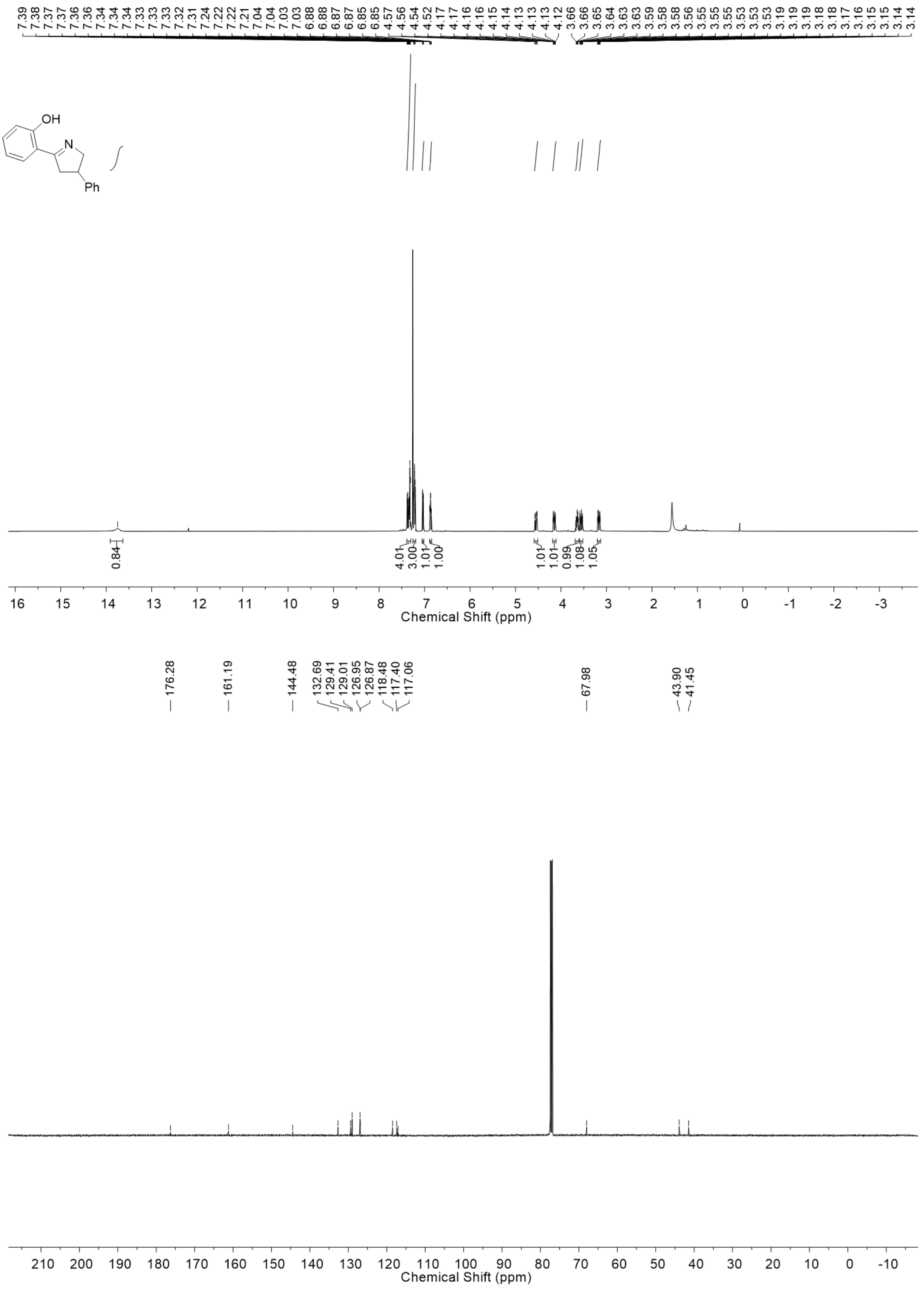

Figure S 65: NMR Spectra of compound (**10**).

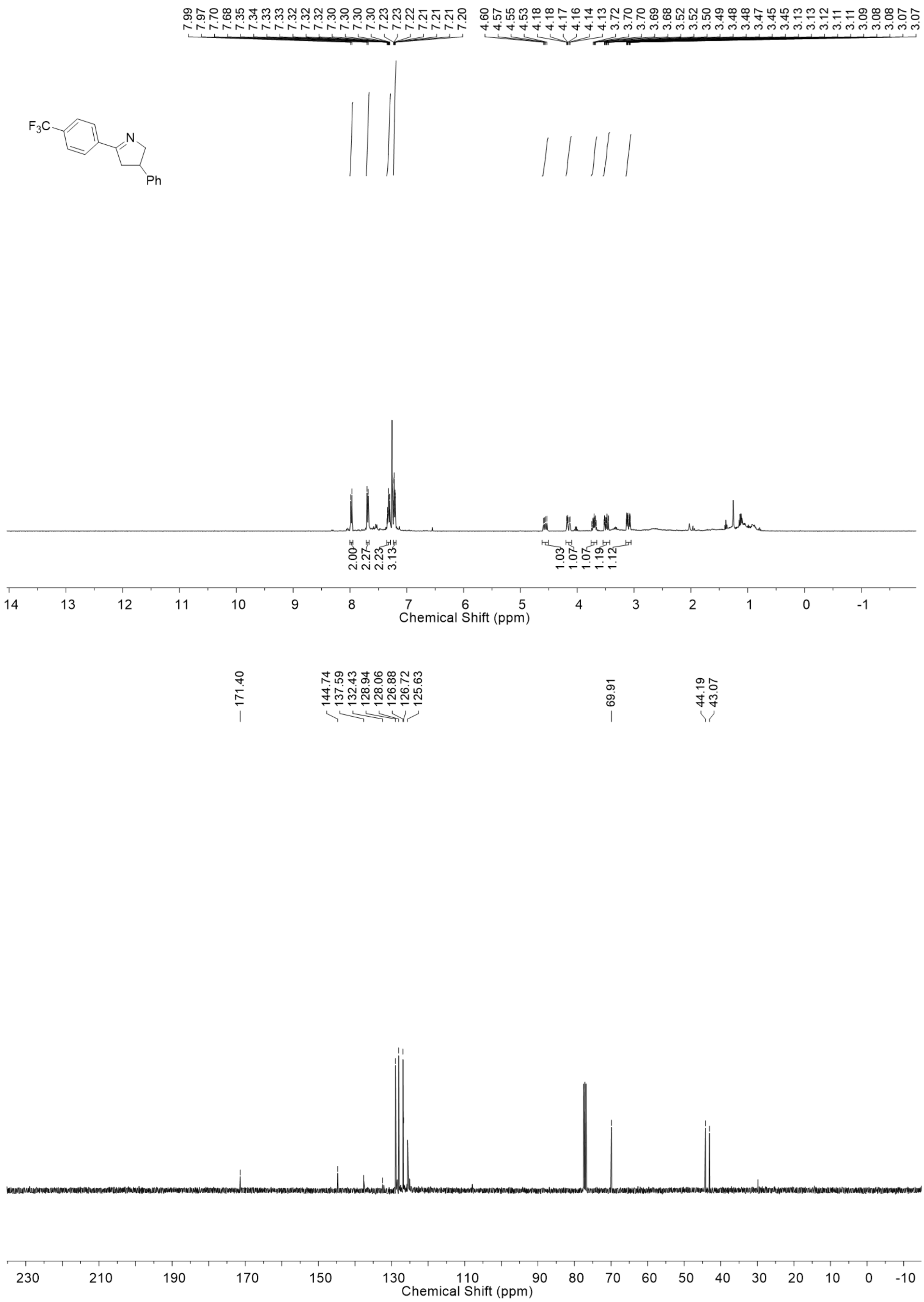

Figure S 66: NMR Spectra of compound (**11**).

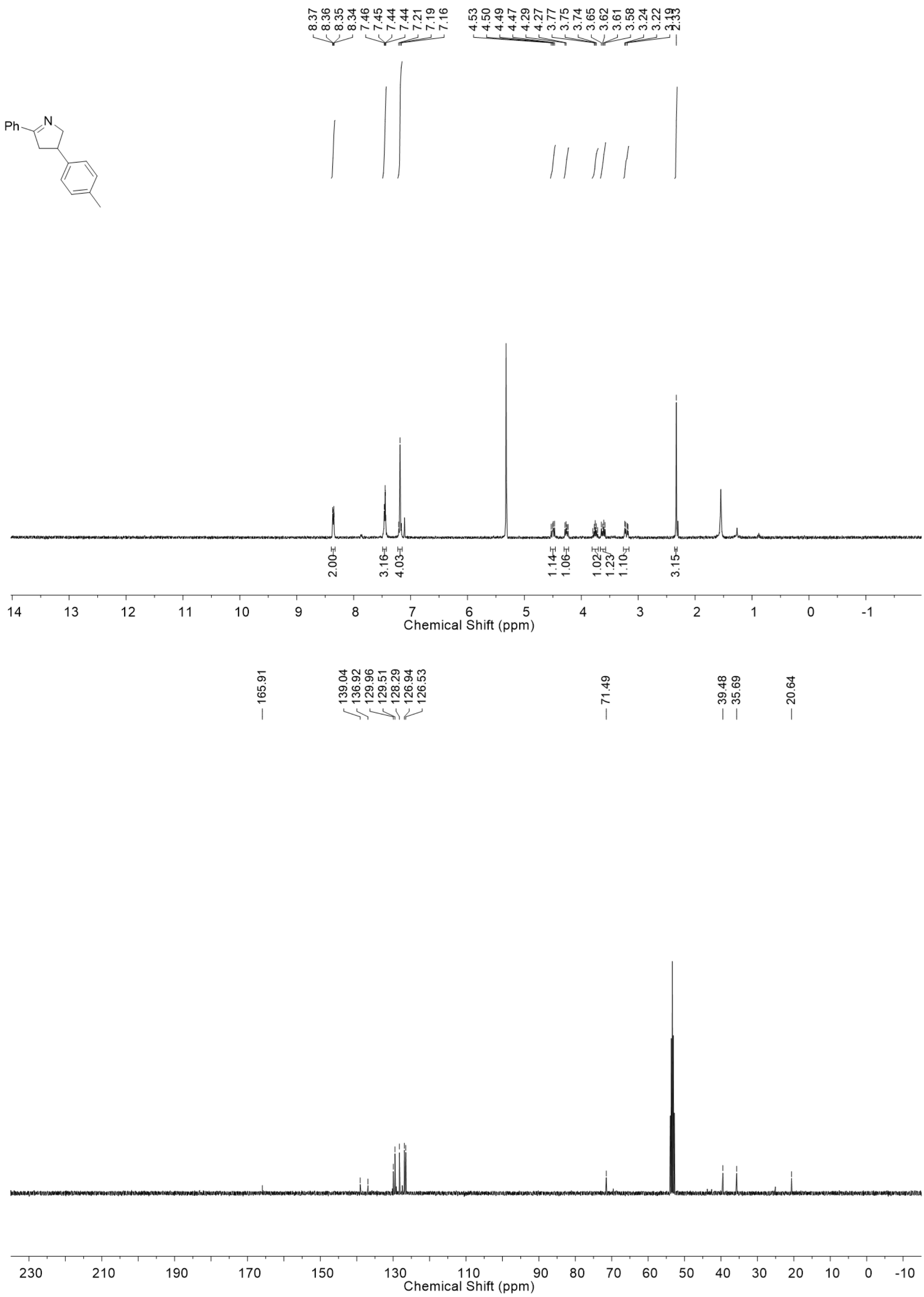

Figure S 67: NMR Spectra of compound (**12**).

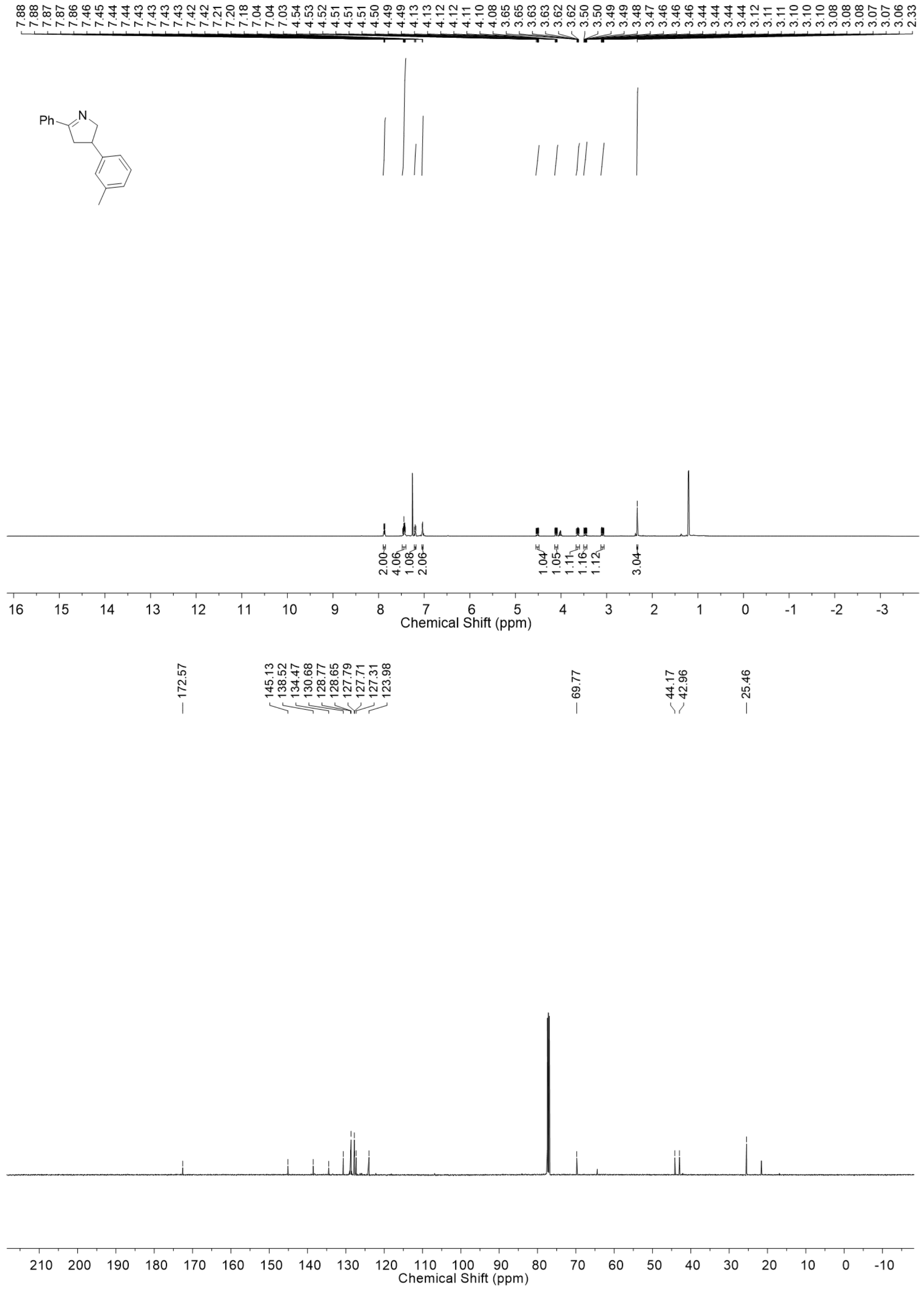

Figure S 68: NMR Spectra of compound (**13**).

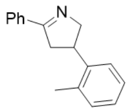

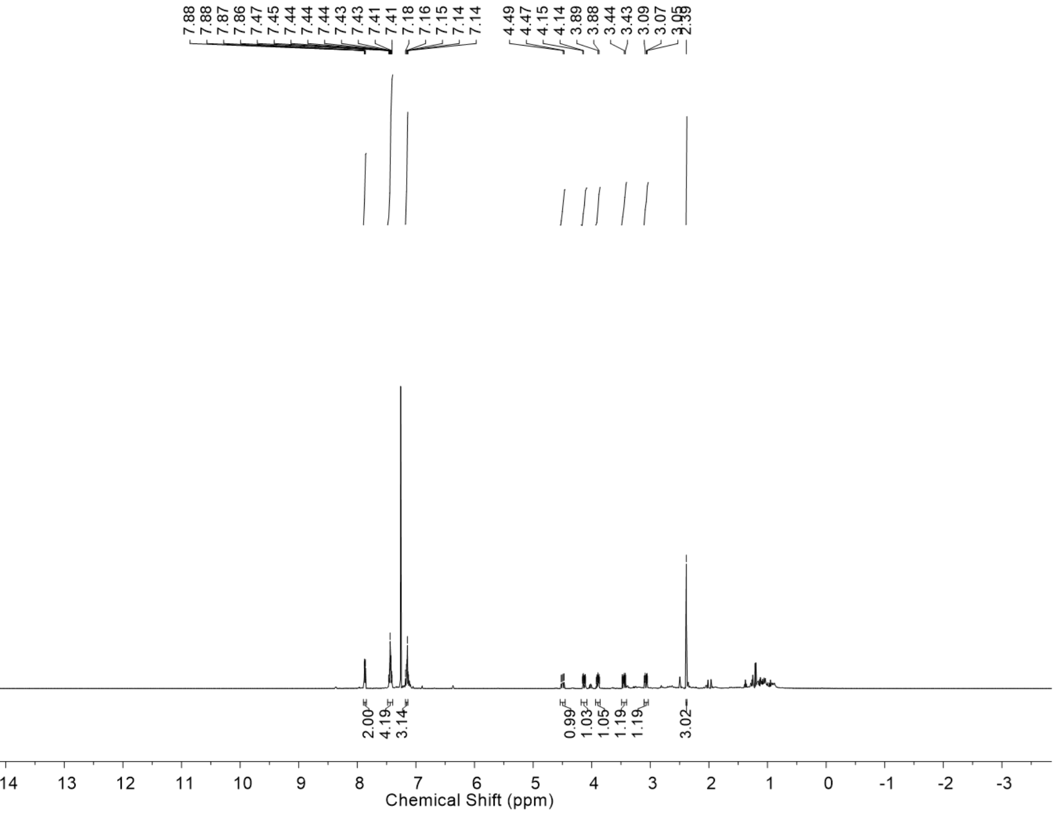

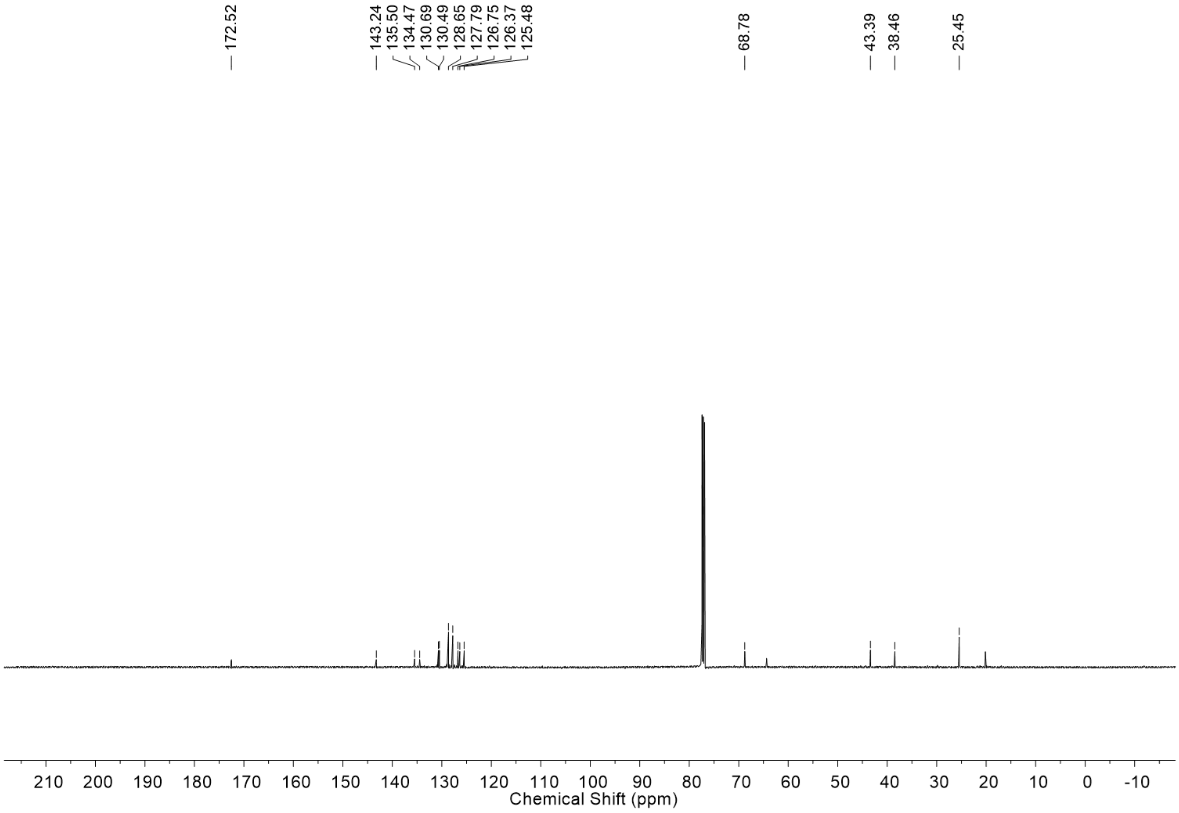

Figure S 69: NMR Spectra of compound (**14**).

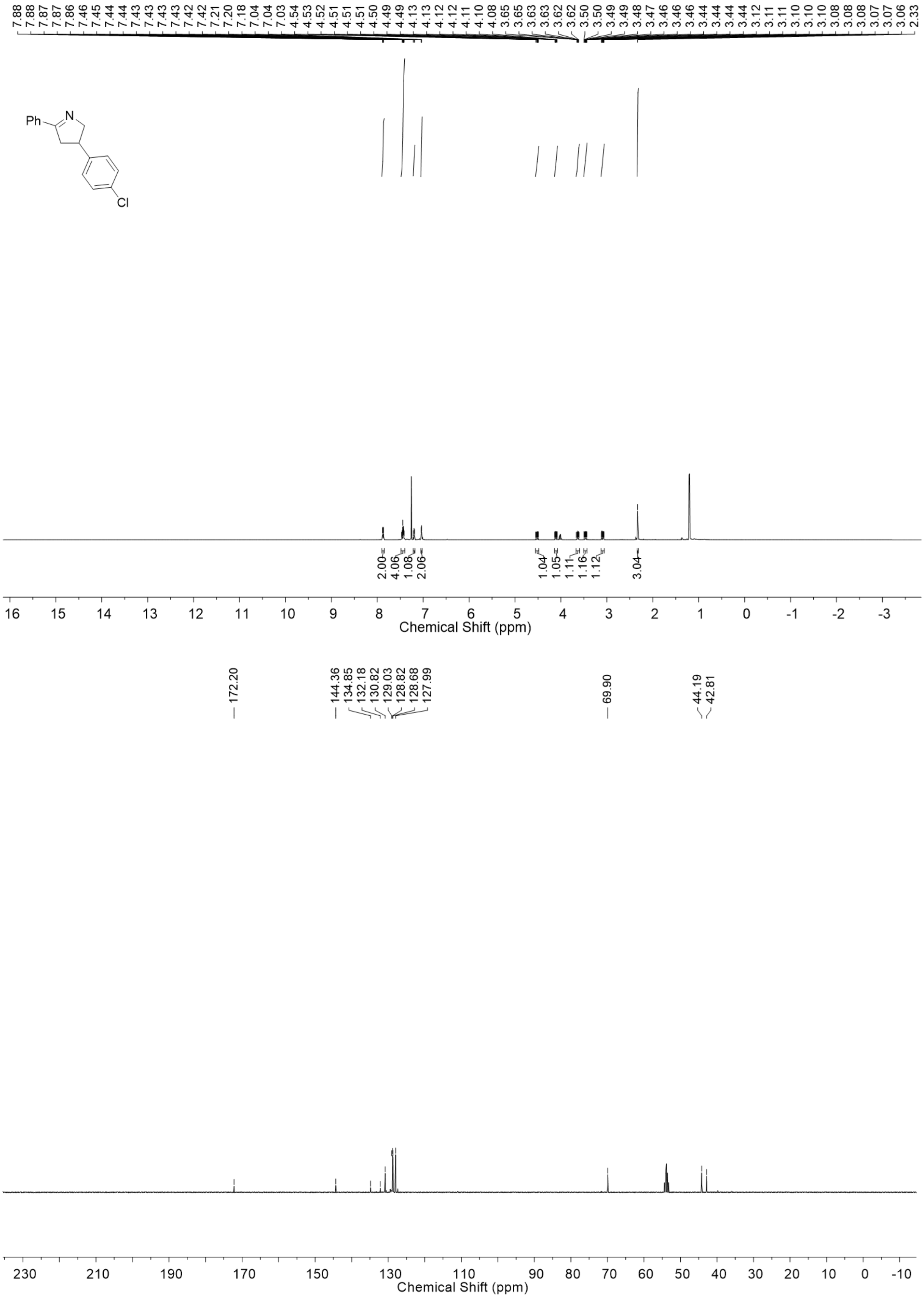

Figure S 70: NMR Spectra of compound (**15**).

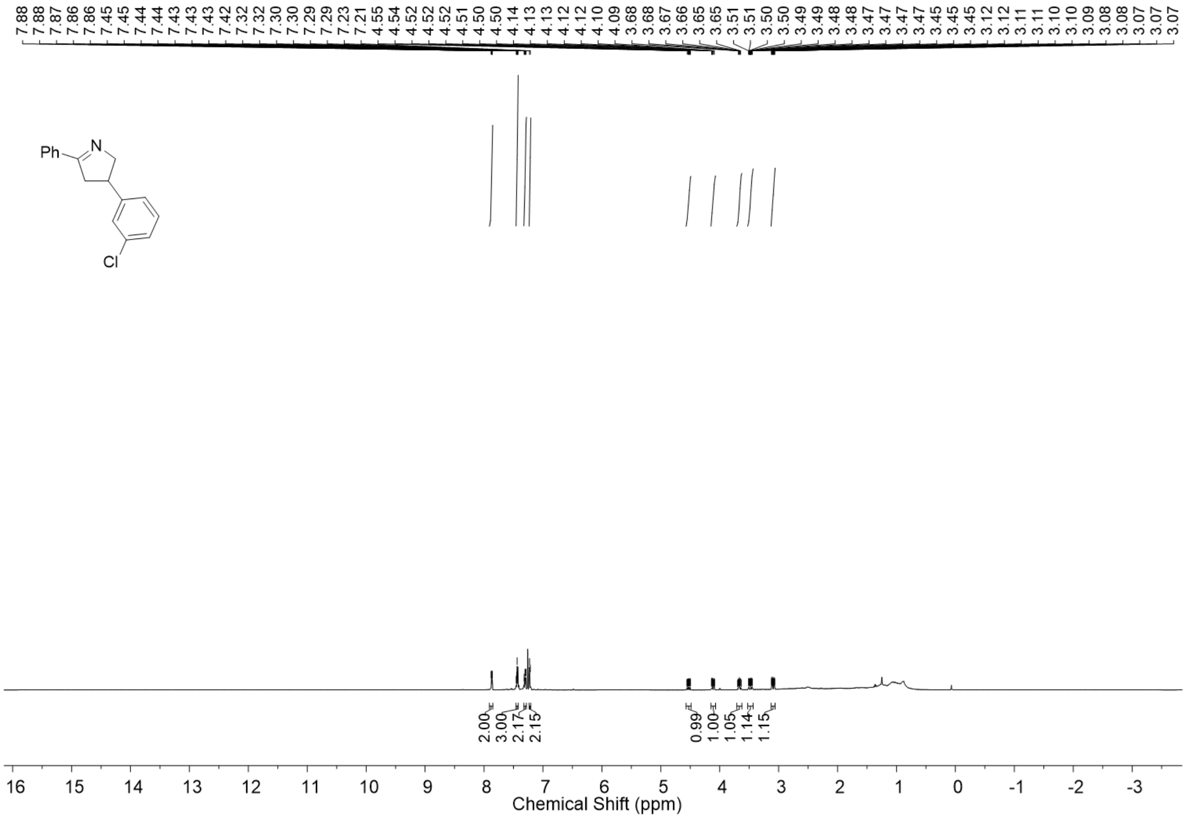

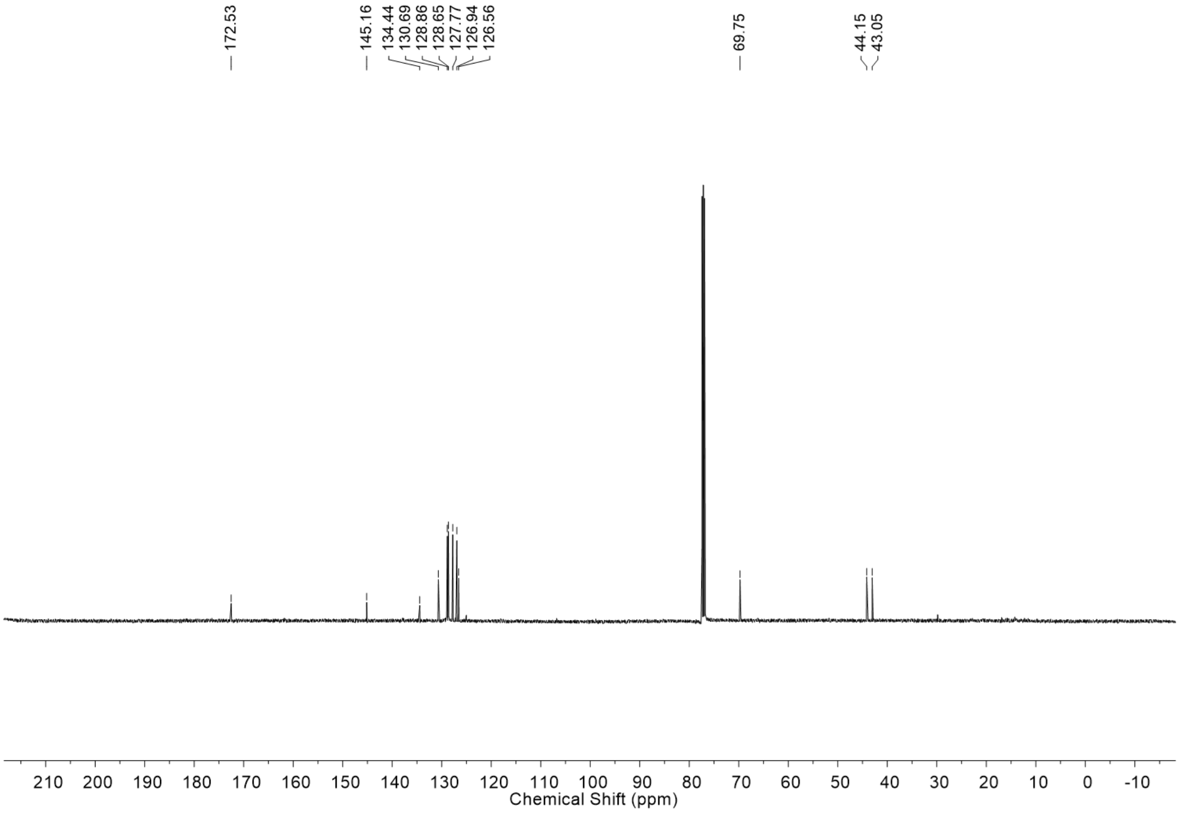

Figure S 71: NMR Spectra of compound (**16**).

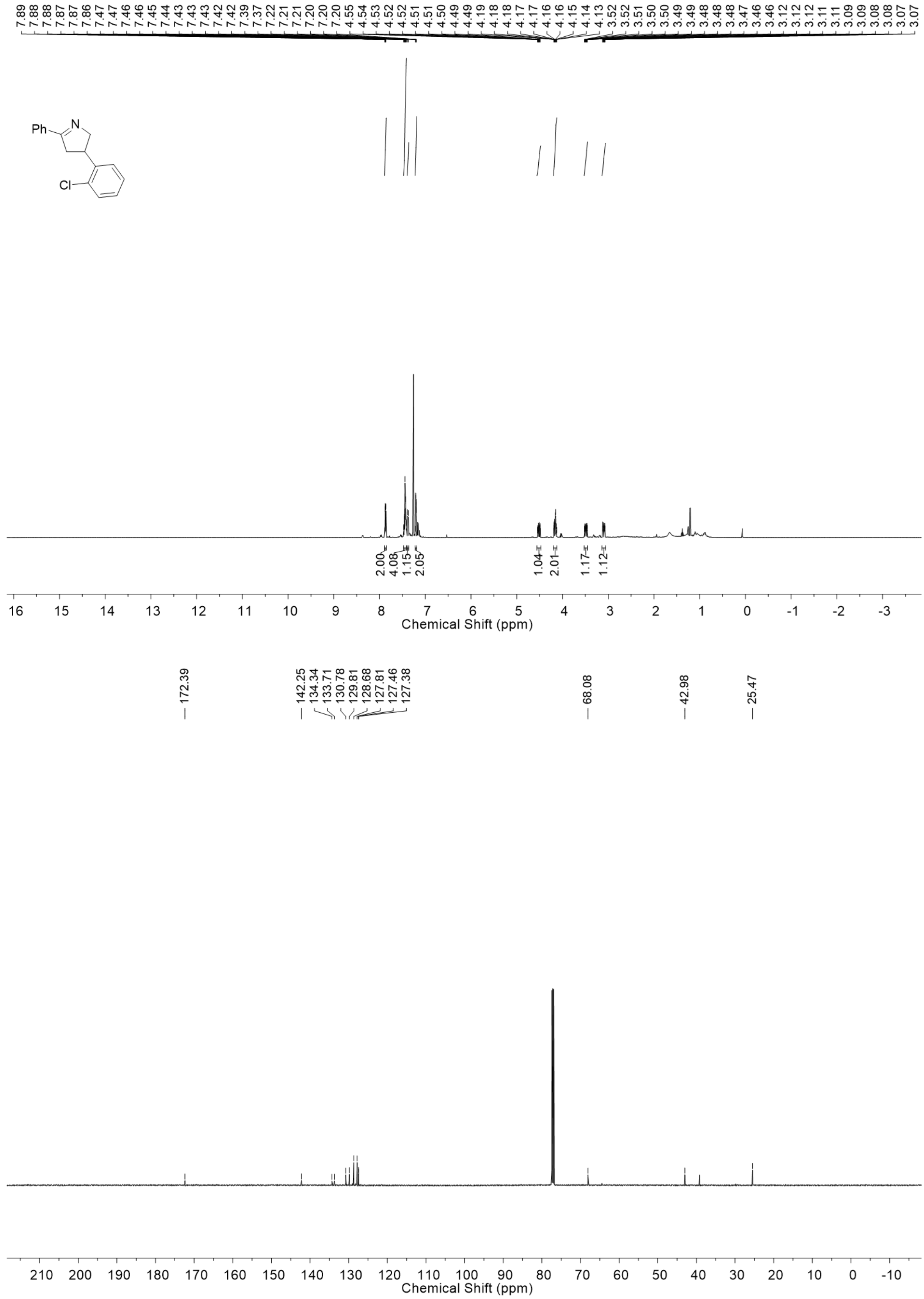

Figure S 72: NMR Spectra of compound (**17**).

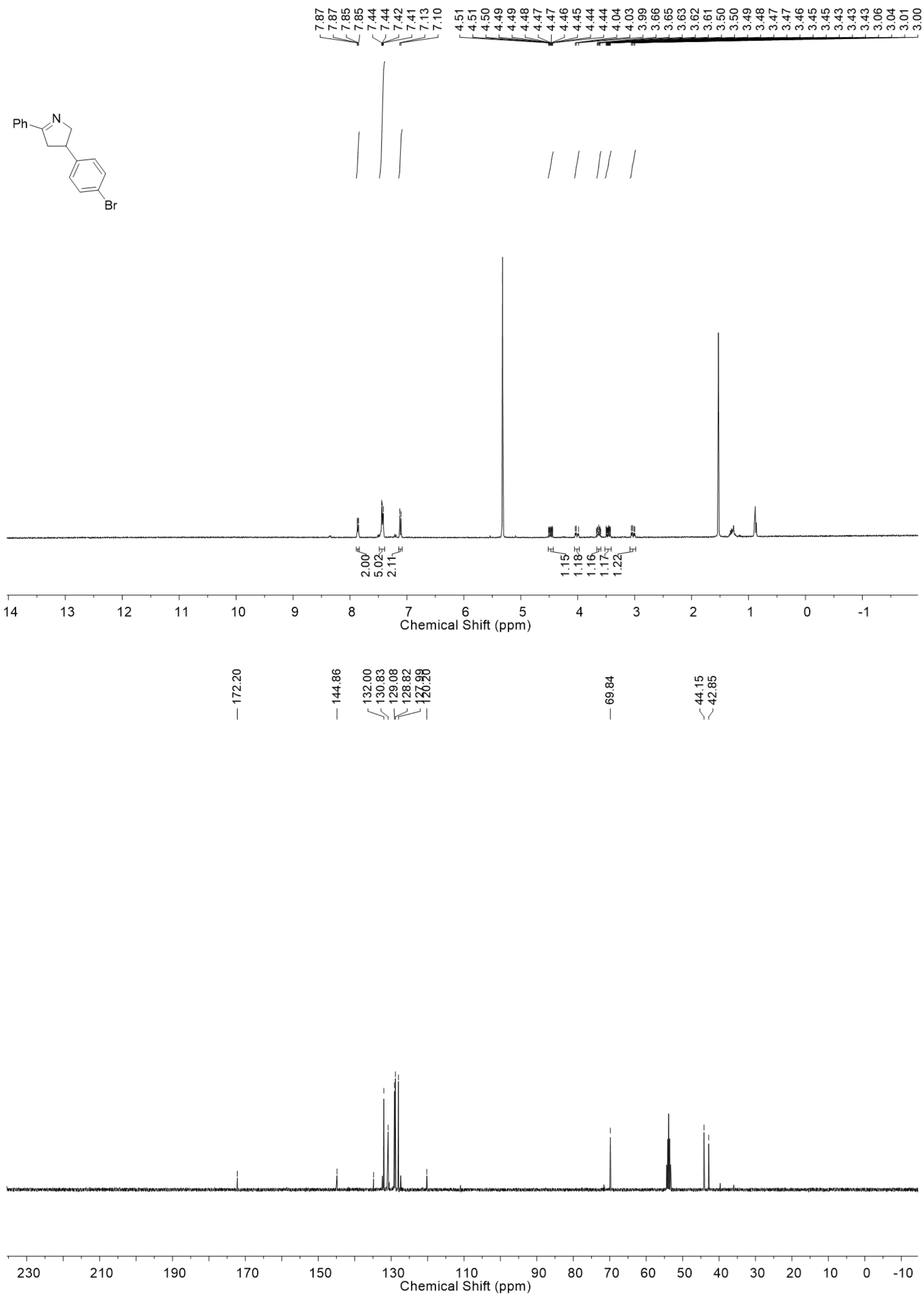

Figure S 73: NMR Spectra of compound (**18**).

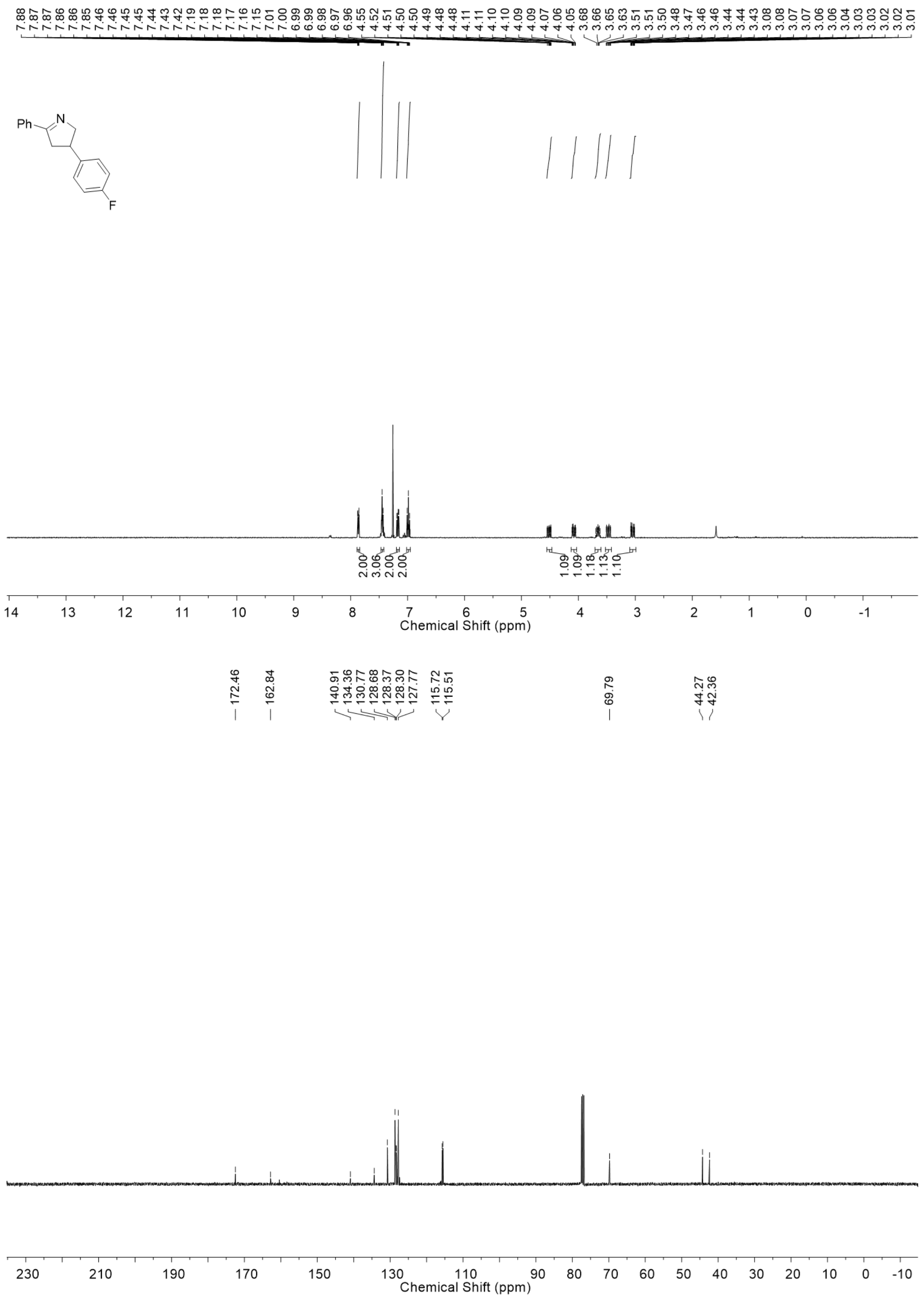

Figure S 74: NMR Spectra of compound (**19**).

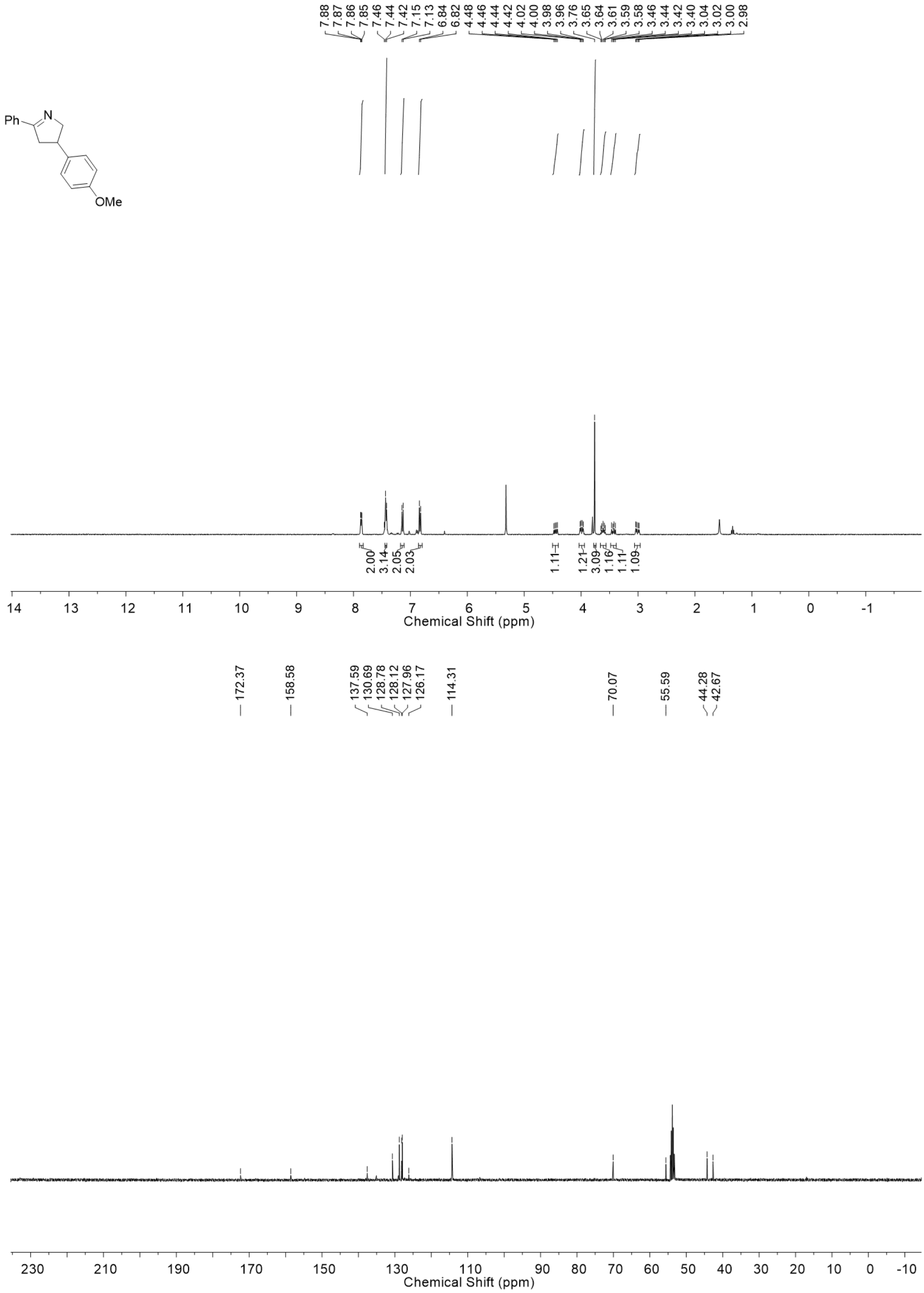

Figure S 75: NMR Spectra of compound (**20**).

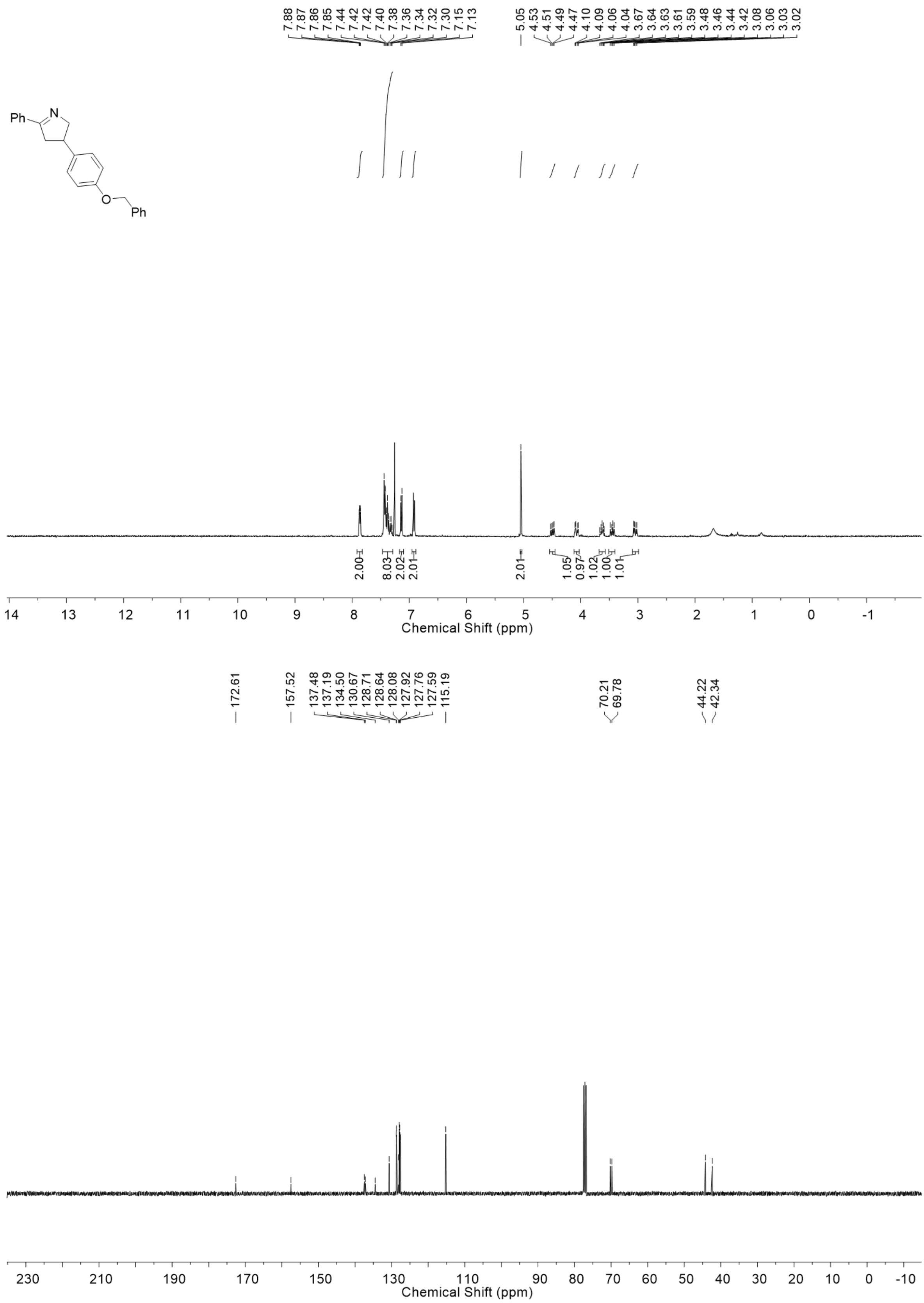

Figure S 76: NMR Spectra of compound (**21**).

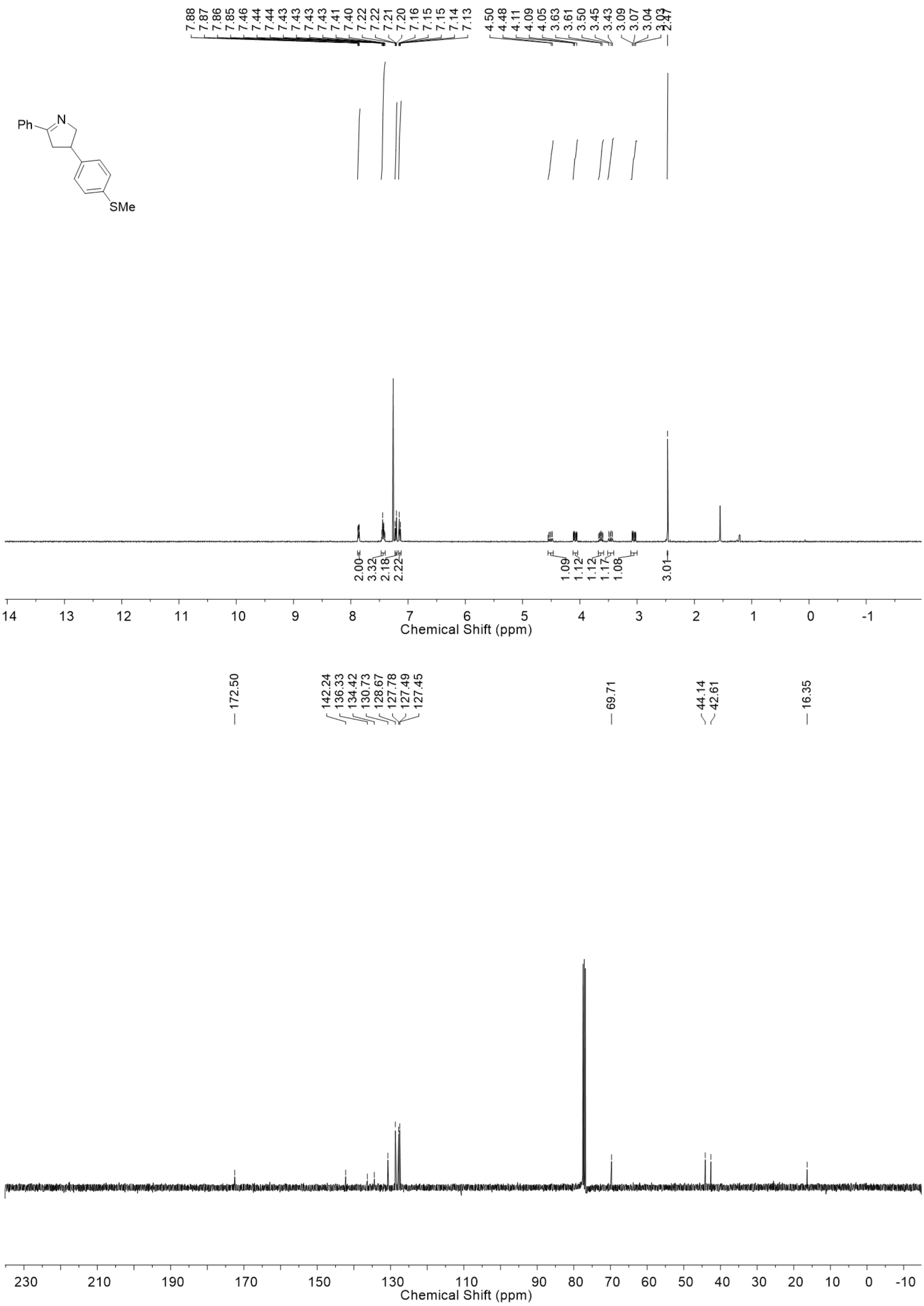

Figure S 77: NMR Spectra of compound (**22**).

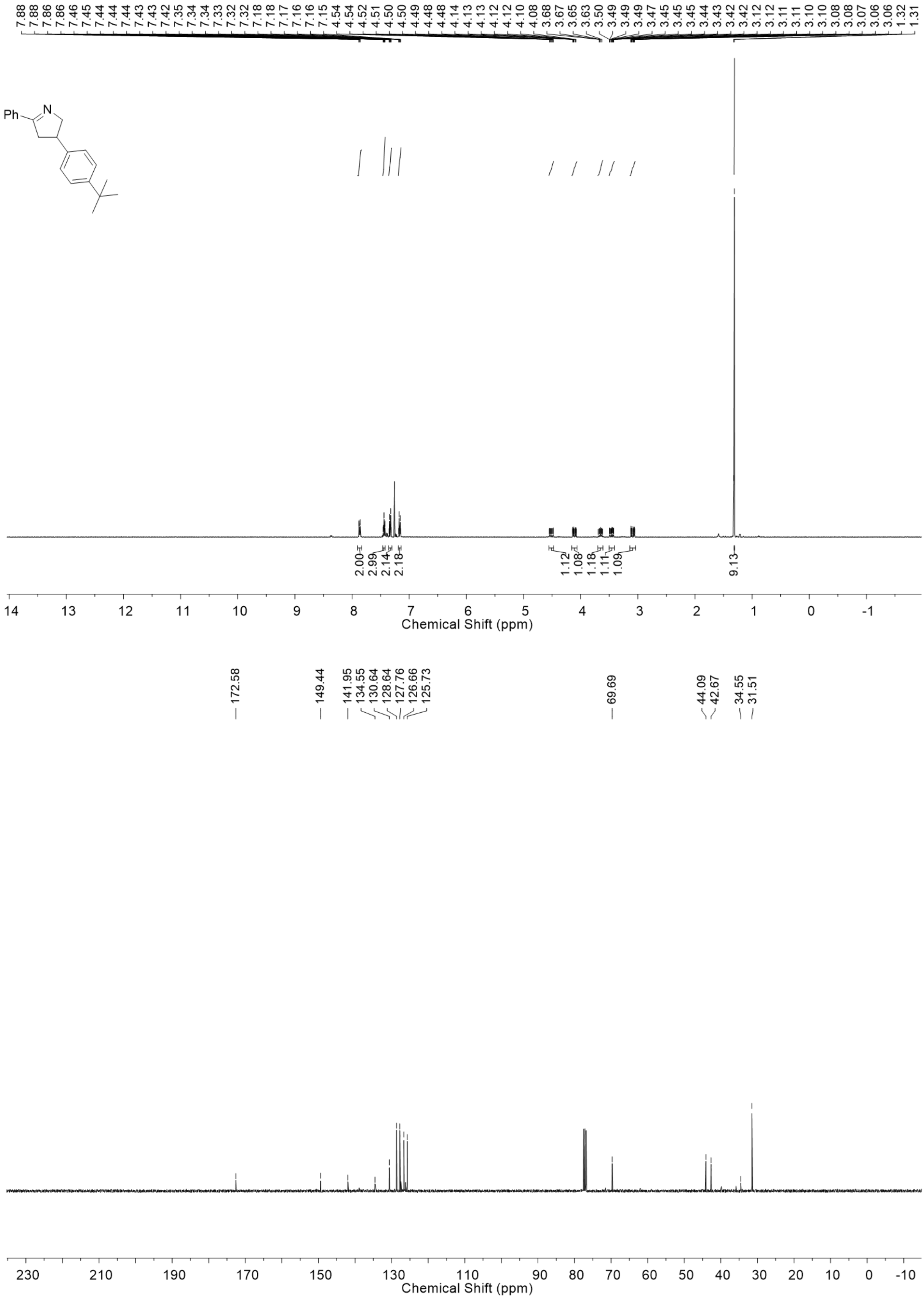

Figure S 78: NMR Spectra of compound (**23**).

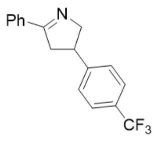

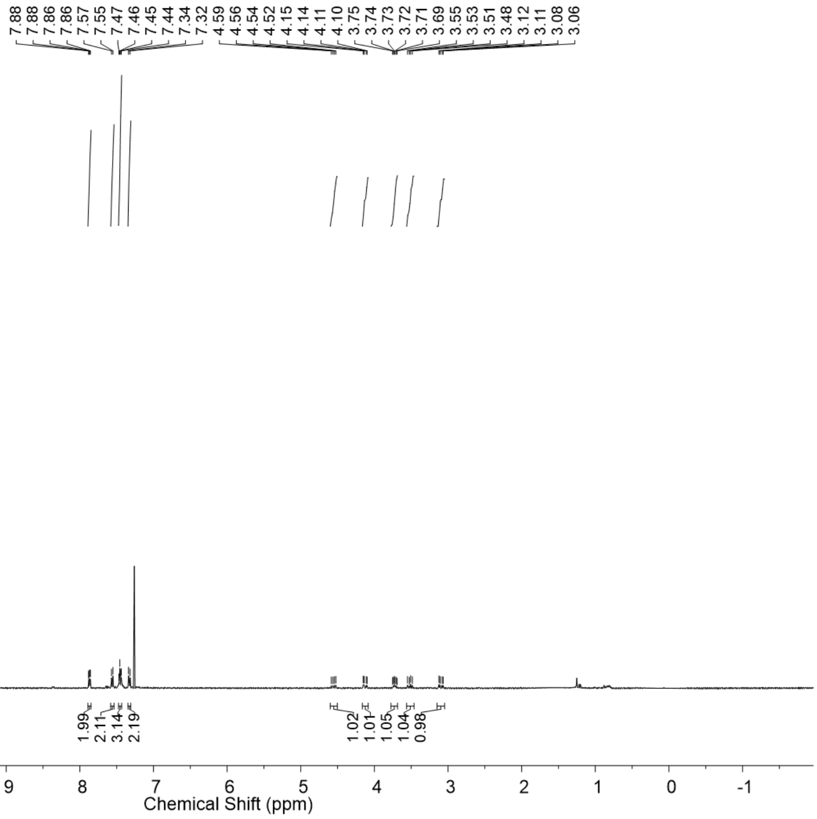

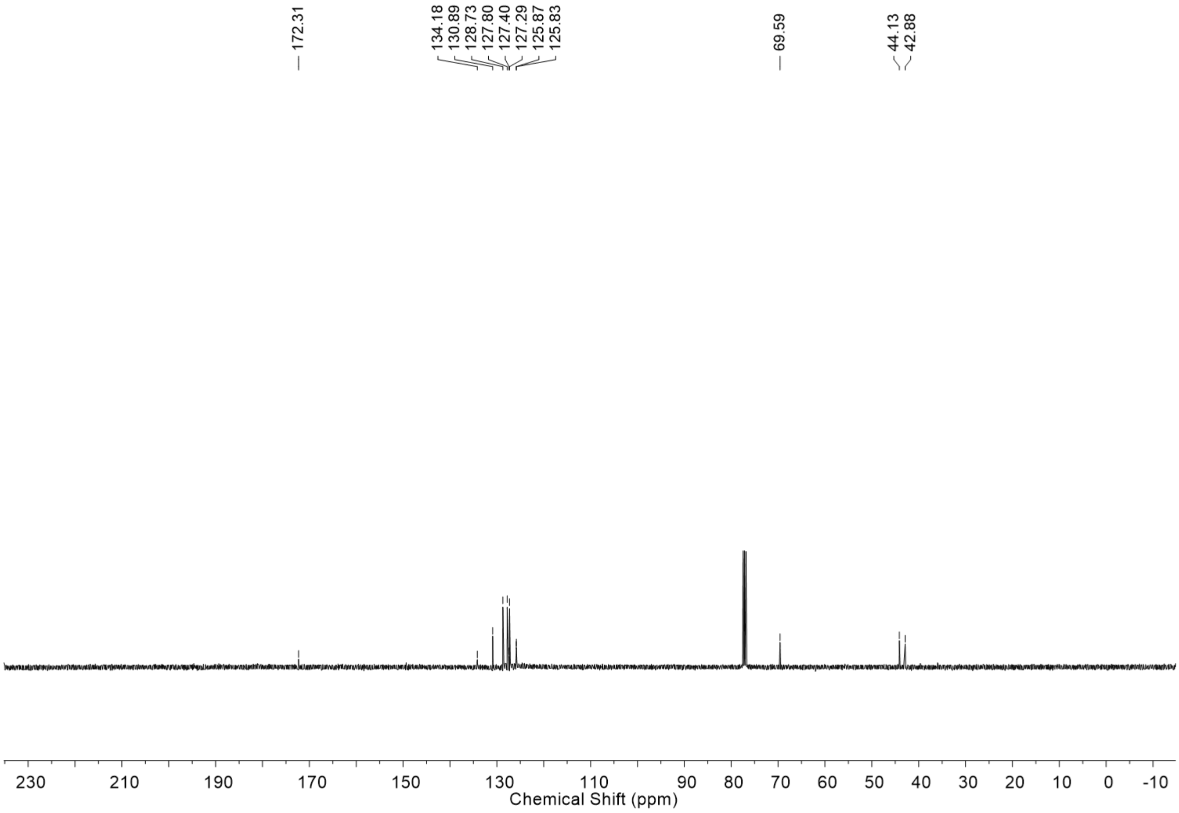

Figure S 79: NMR Spectra of compound (**24**).

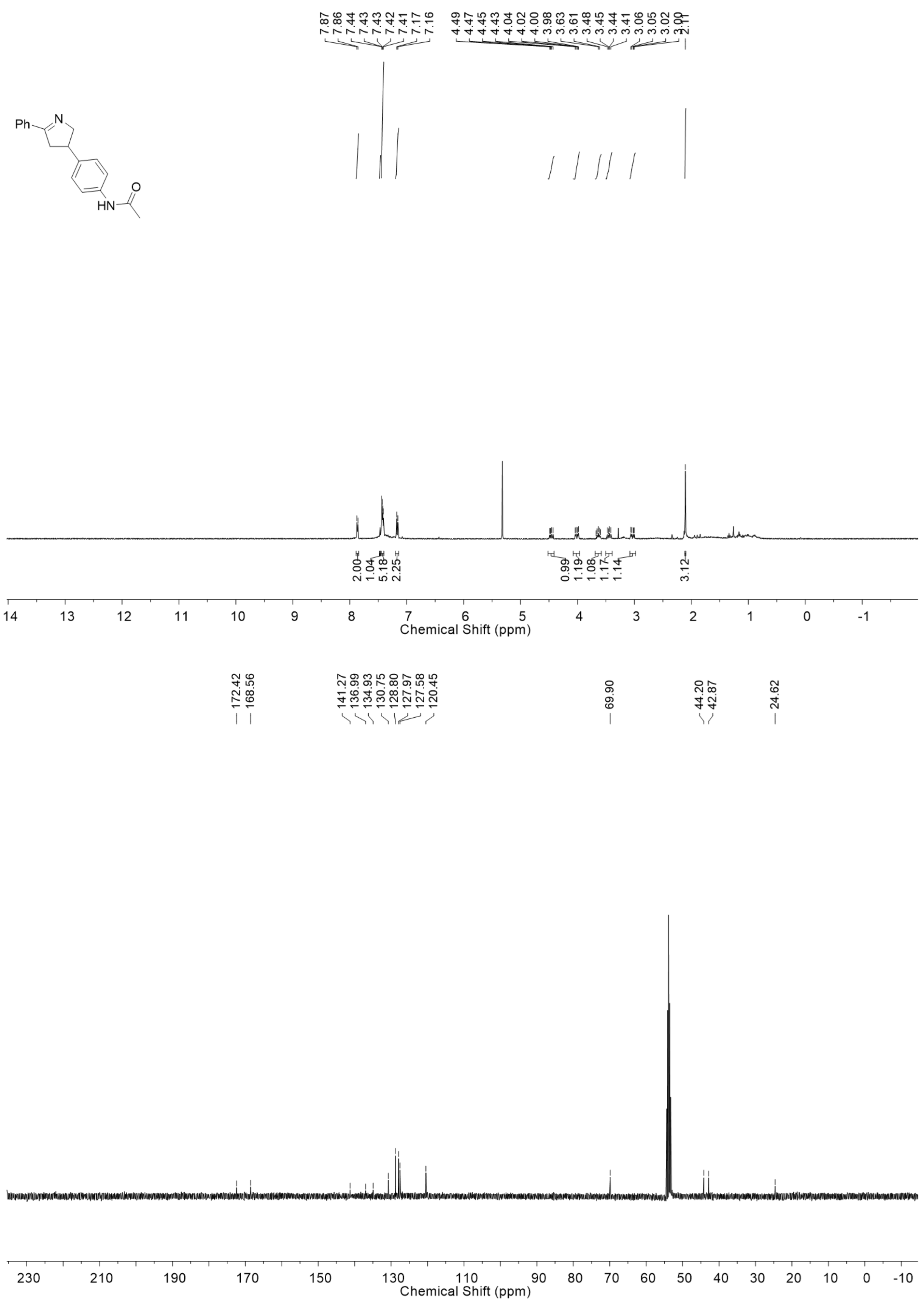

Figure S 80: NMR Spectra of compound (**25**).

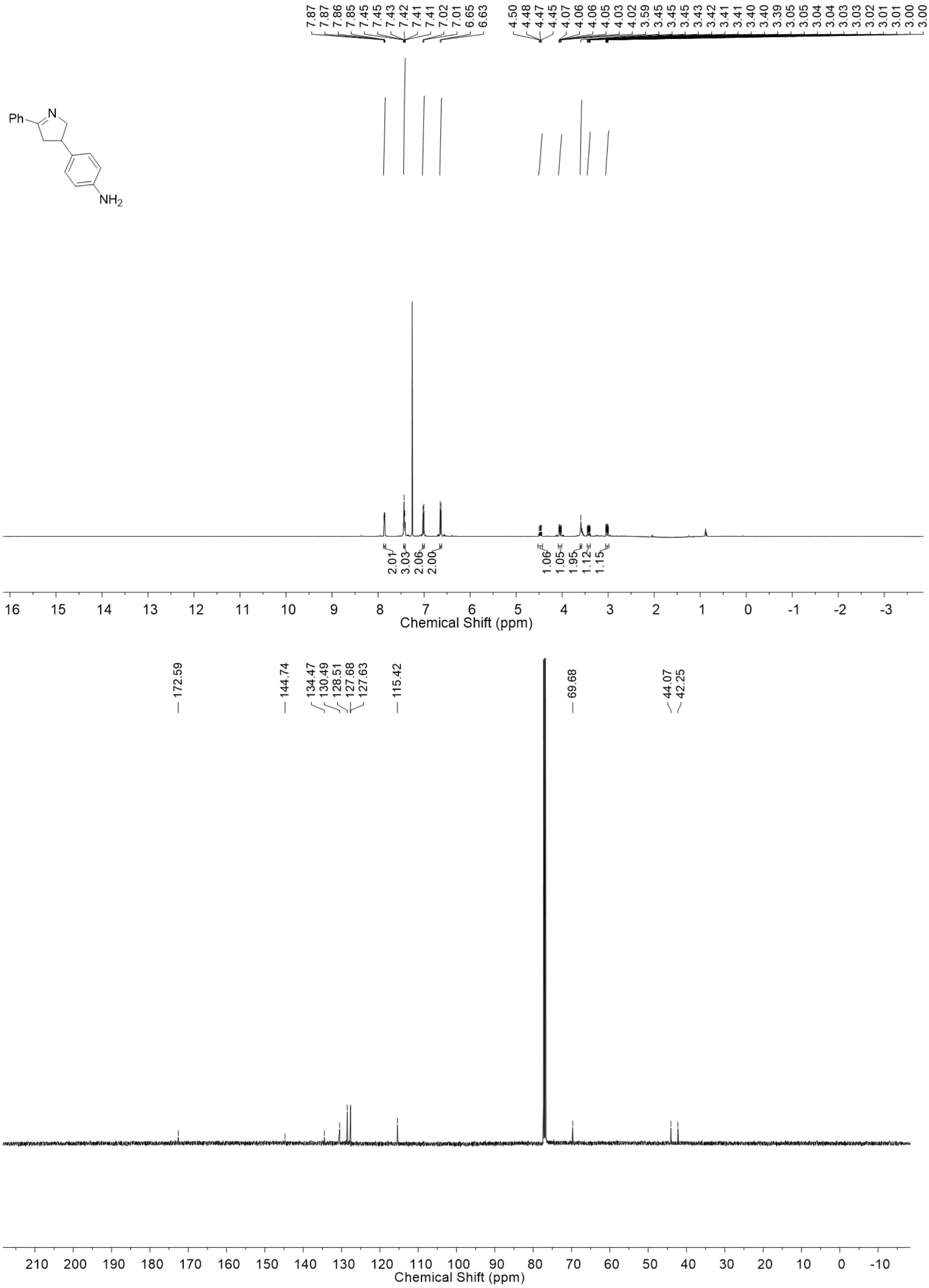

Figure S 81: NMR Spectra of compound (**26**).

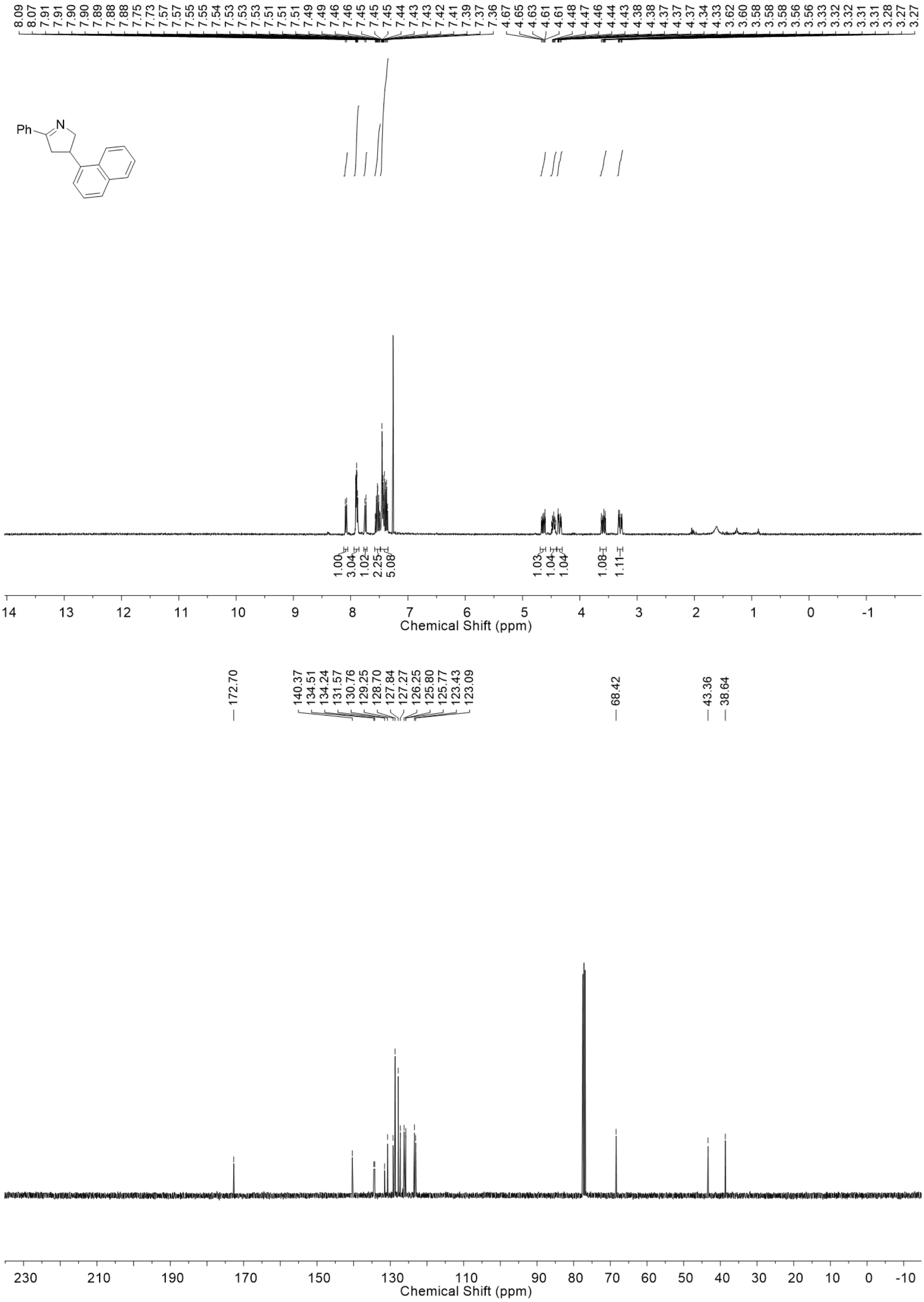

Figure S 82: NMR Spectra of compound (**27**).

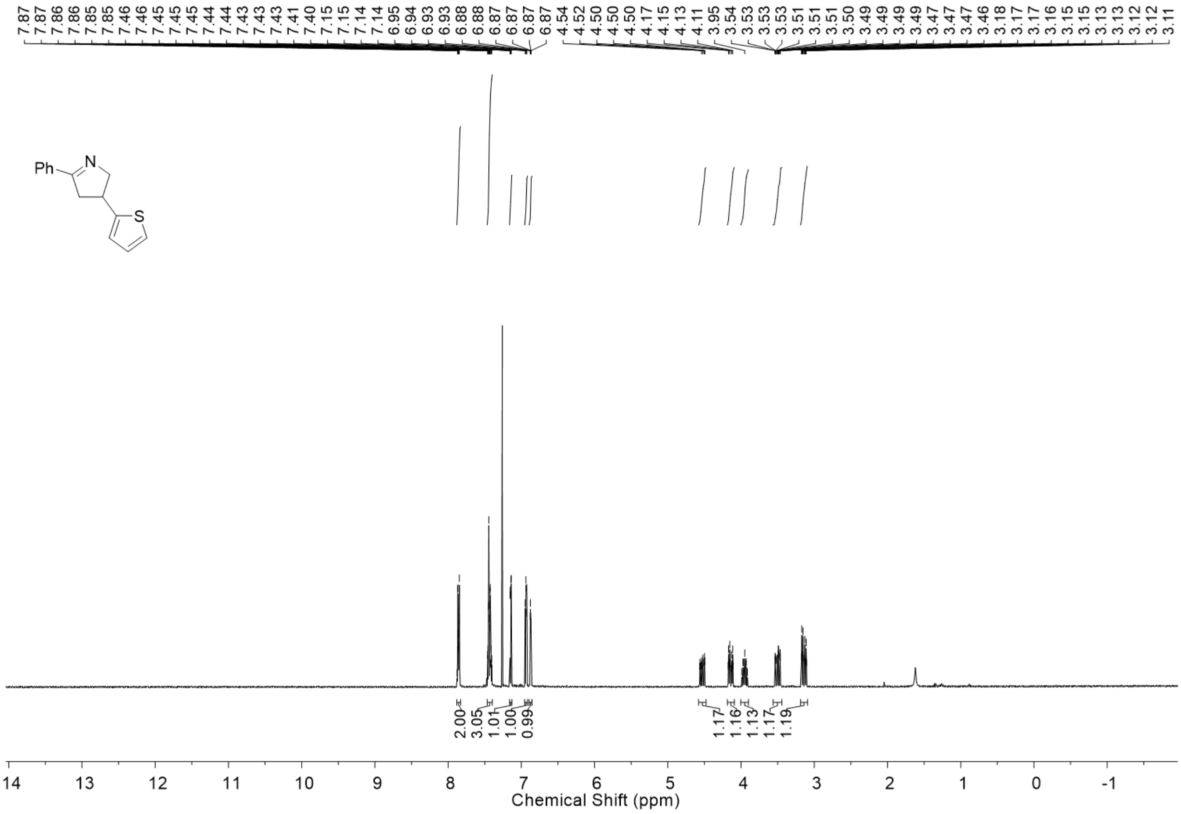

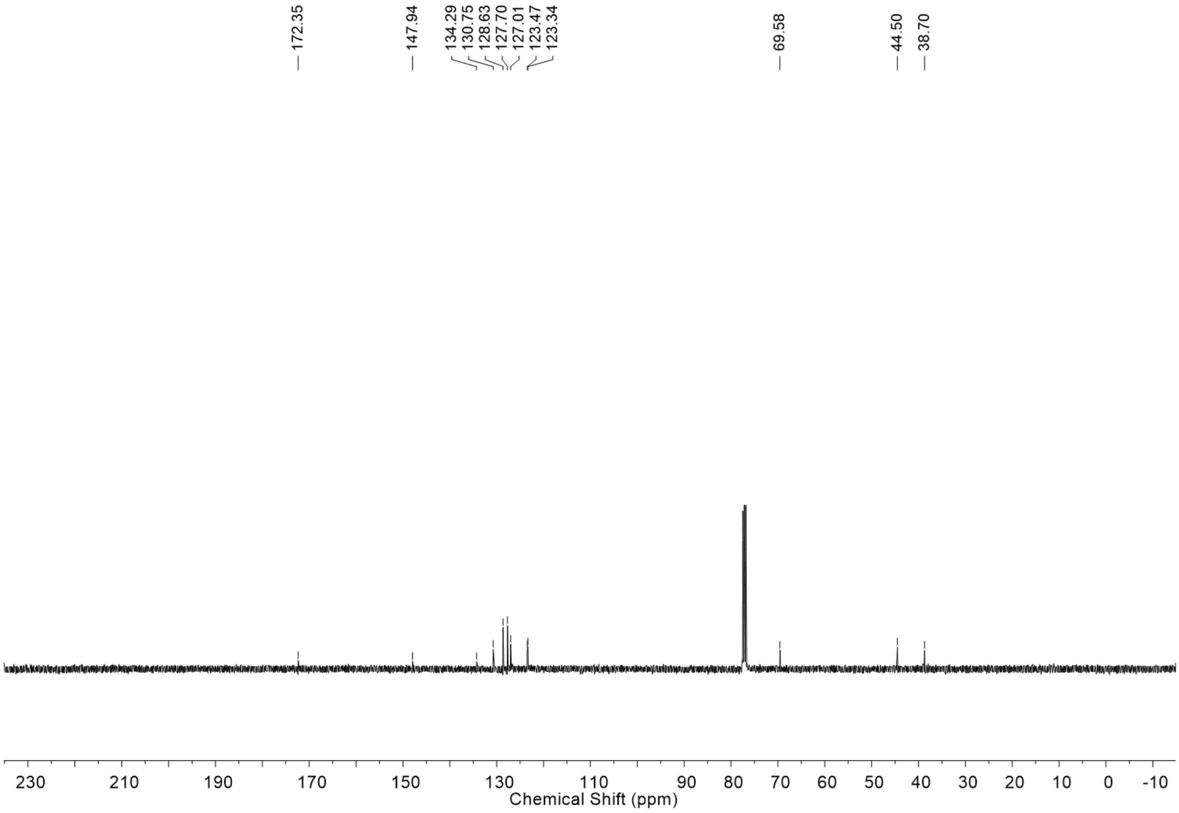

Figure S 83: NMR Spectra of compound (**28**).

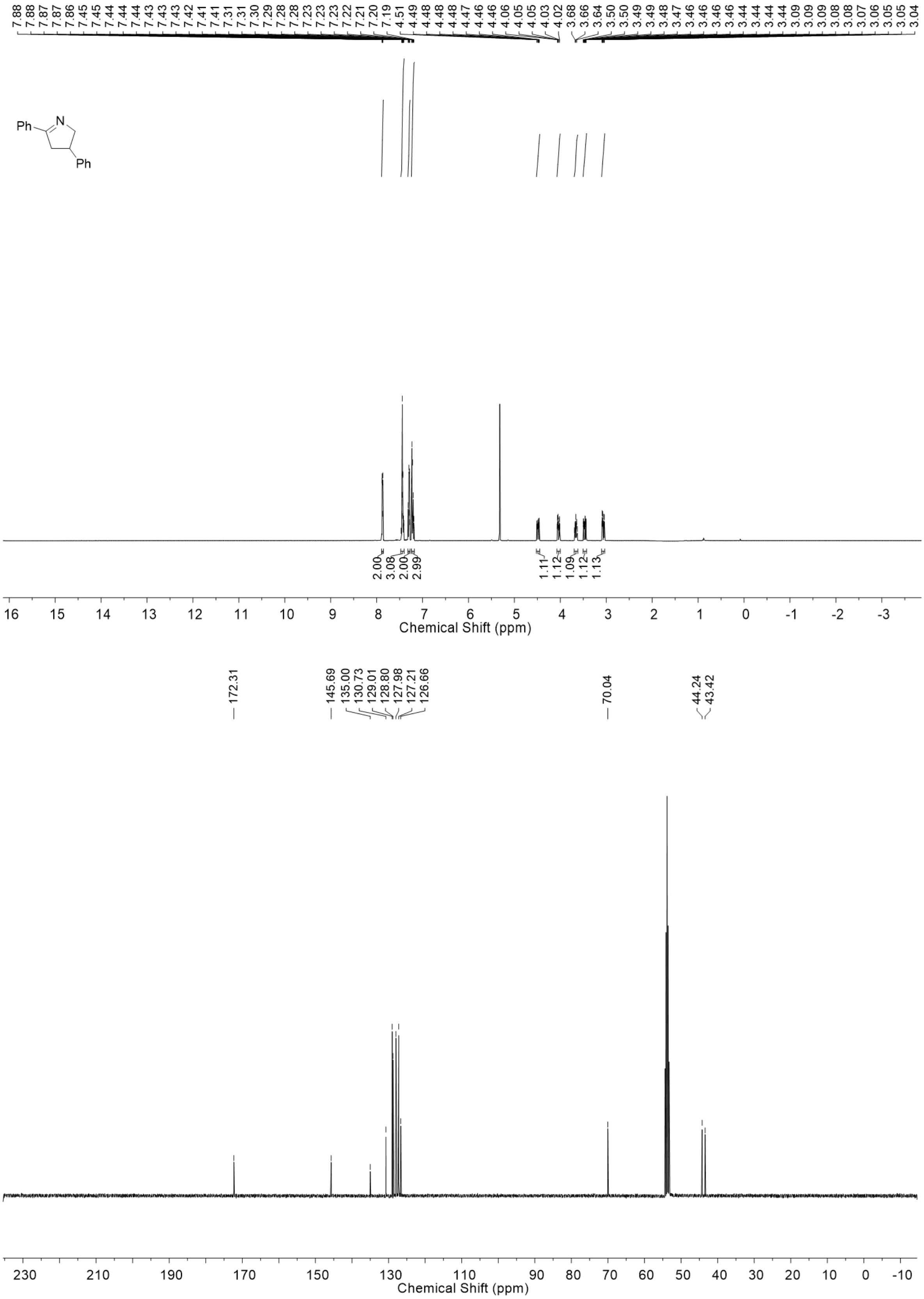

Figure S 84: NMR Spectra of compound (**29**).

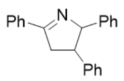

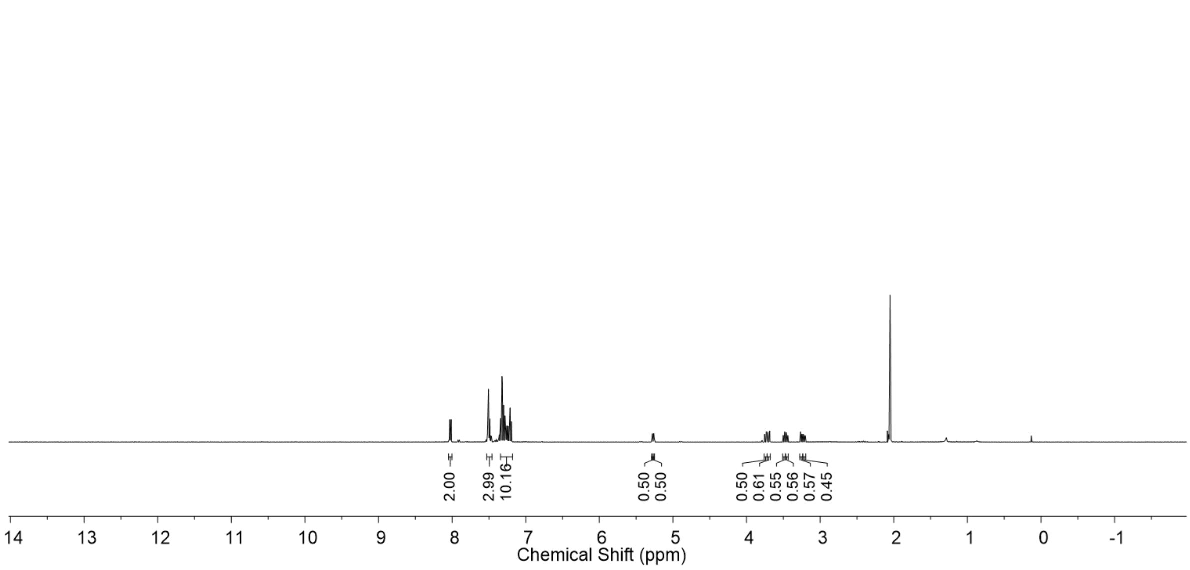

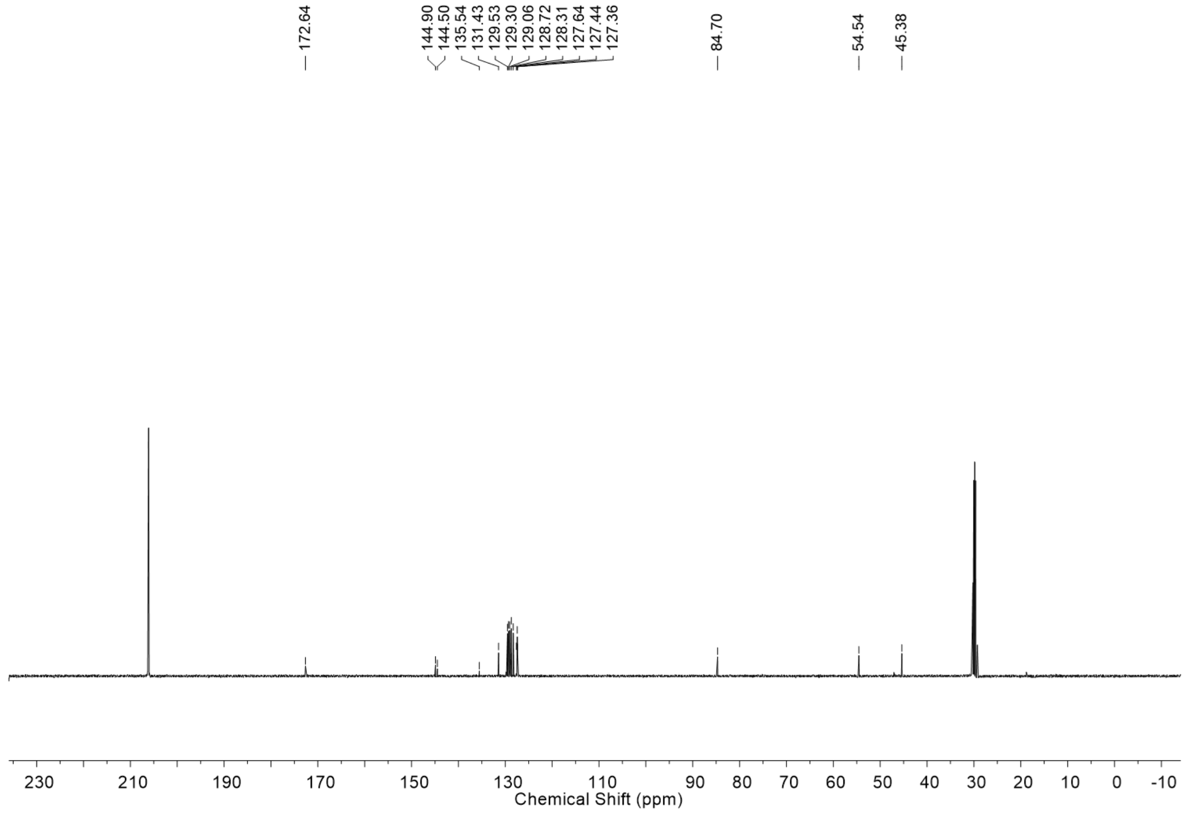

Figure S 85: NMR Spectra of compound (**30**).

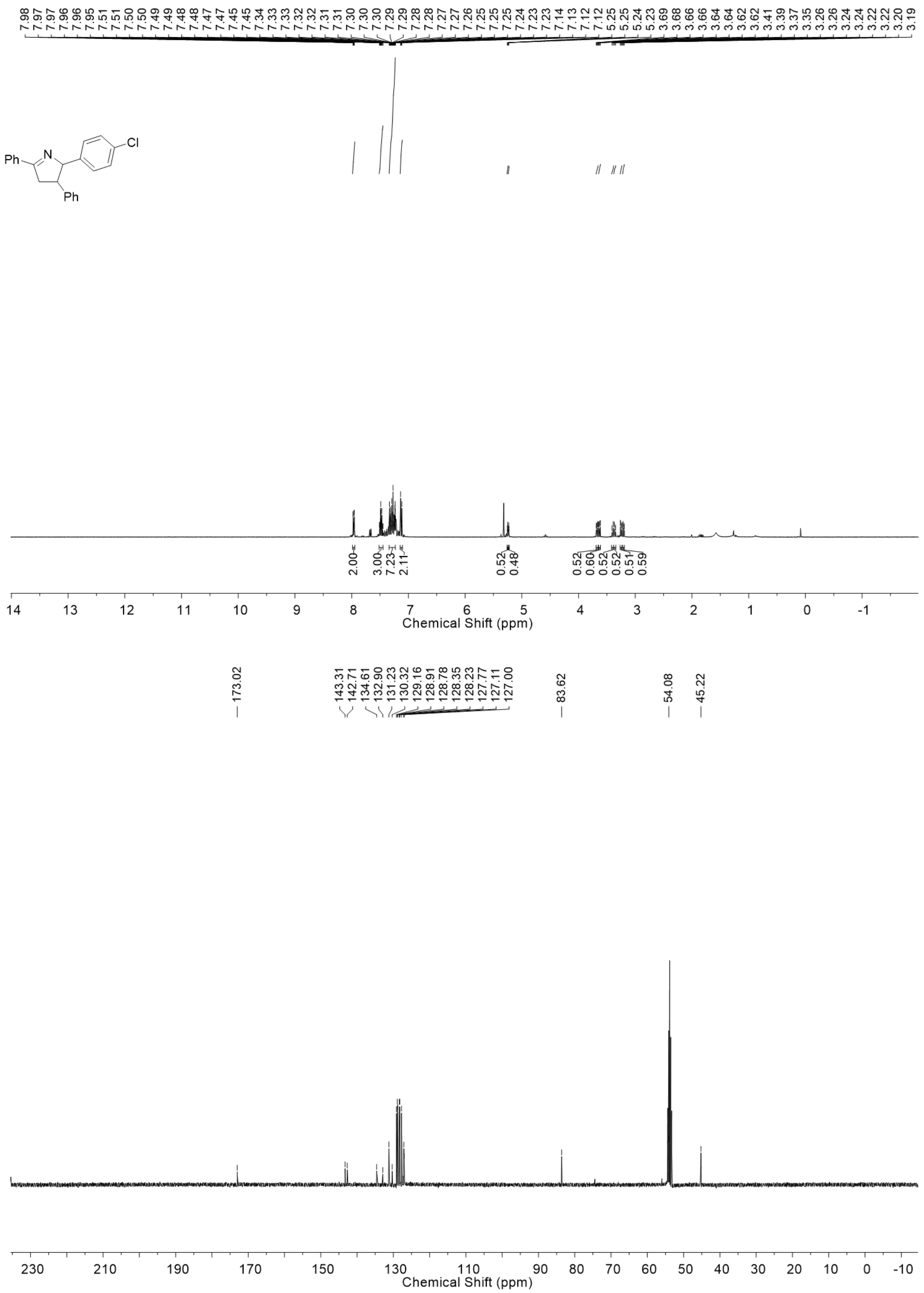

Figure S 86: NMR Spectra of compound (**31**).

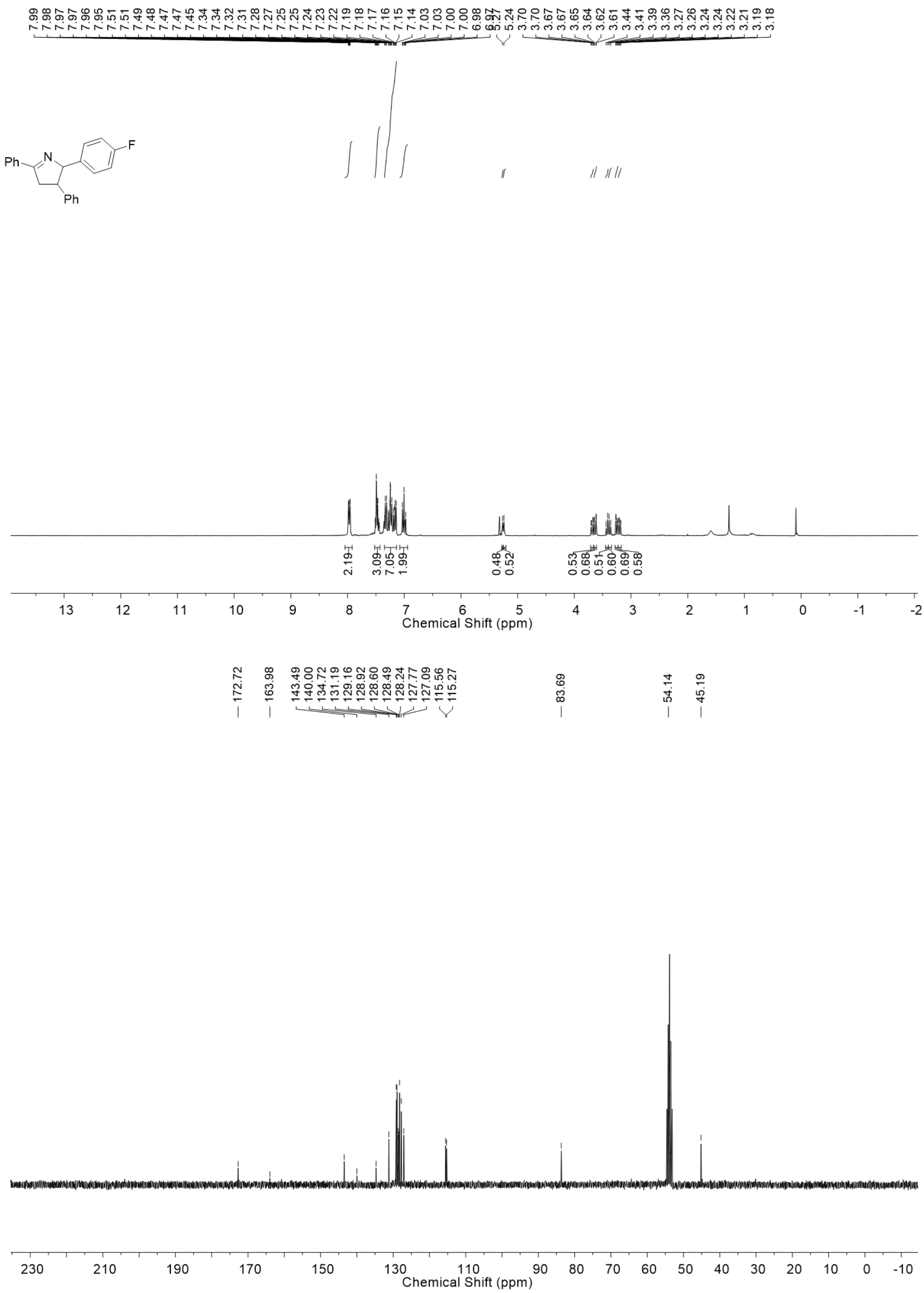

Figure S 87: NMR Spectra of compound (**32**).

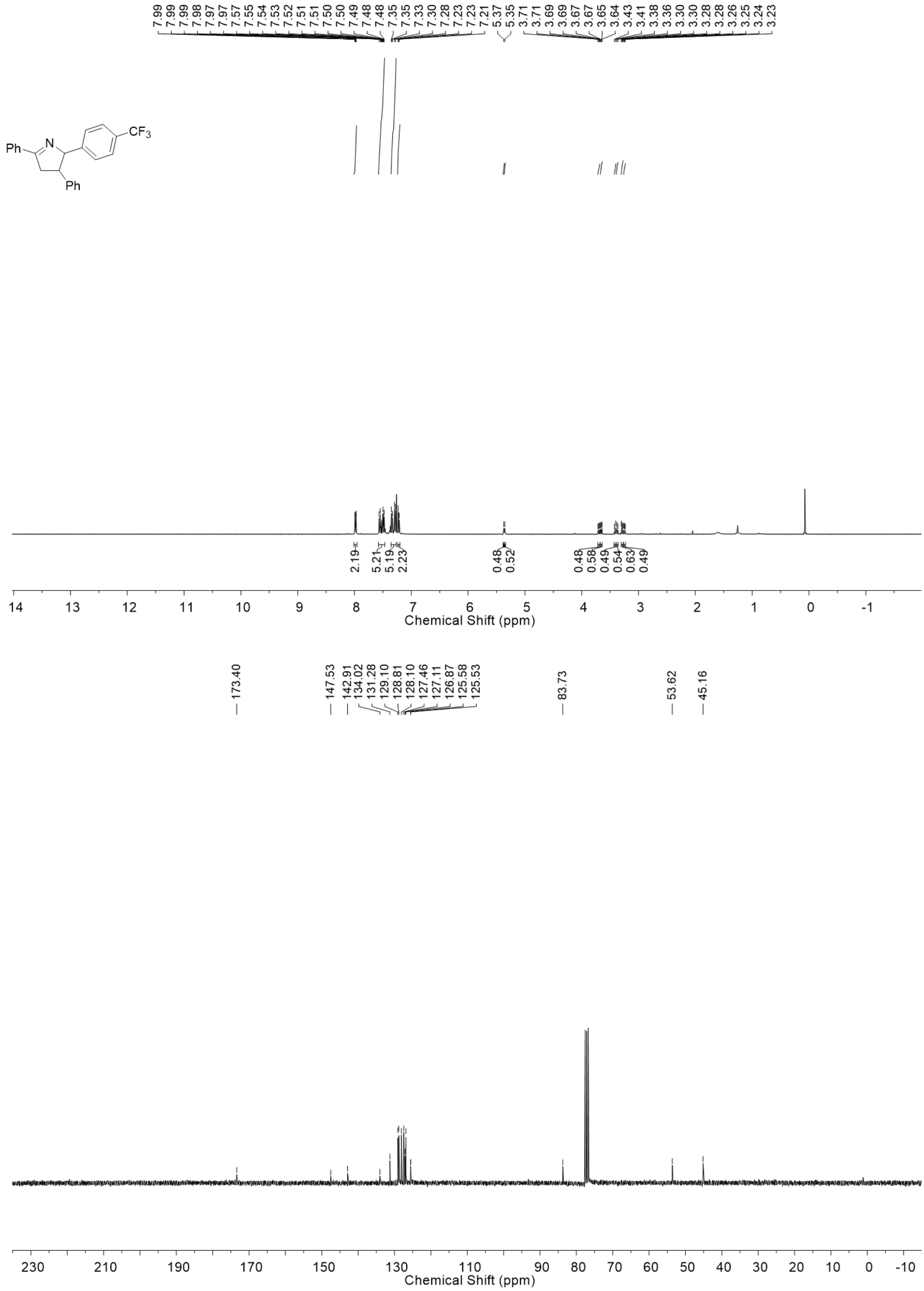

Figure S 88: NMR Spectra of compound (**33**).

11. HRMS Spectra

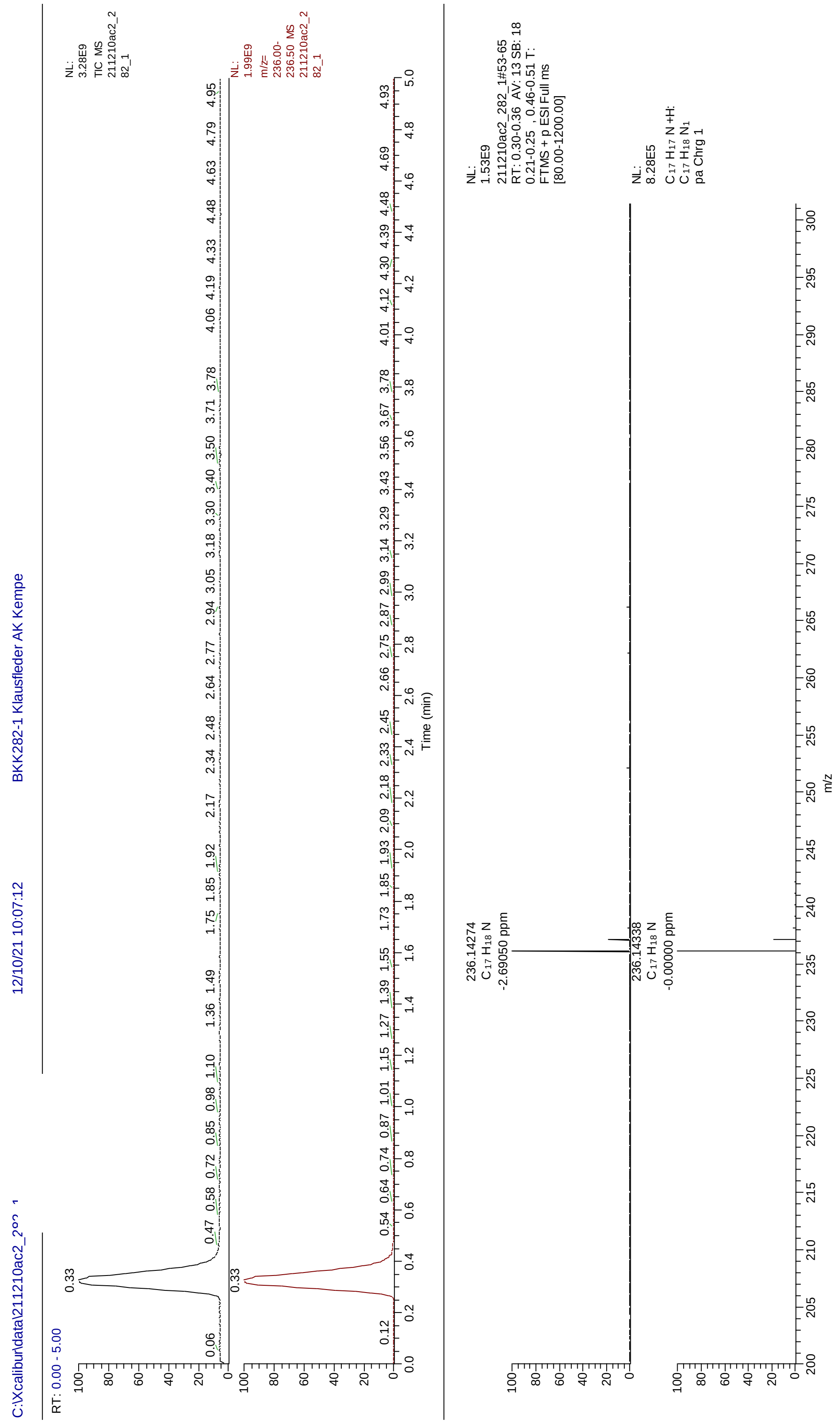

Figure S 89: HRMS-Spectra of compound (**2**).

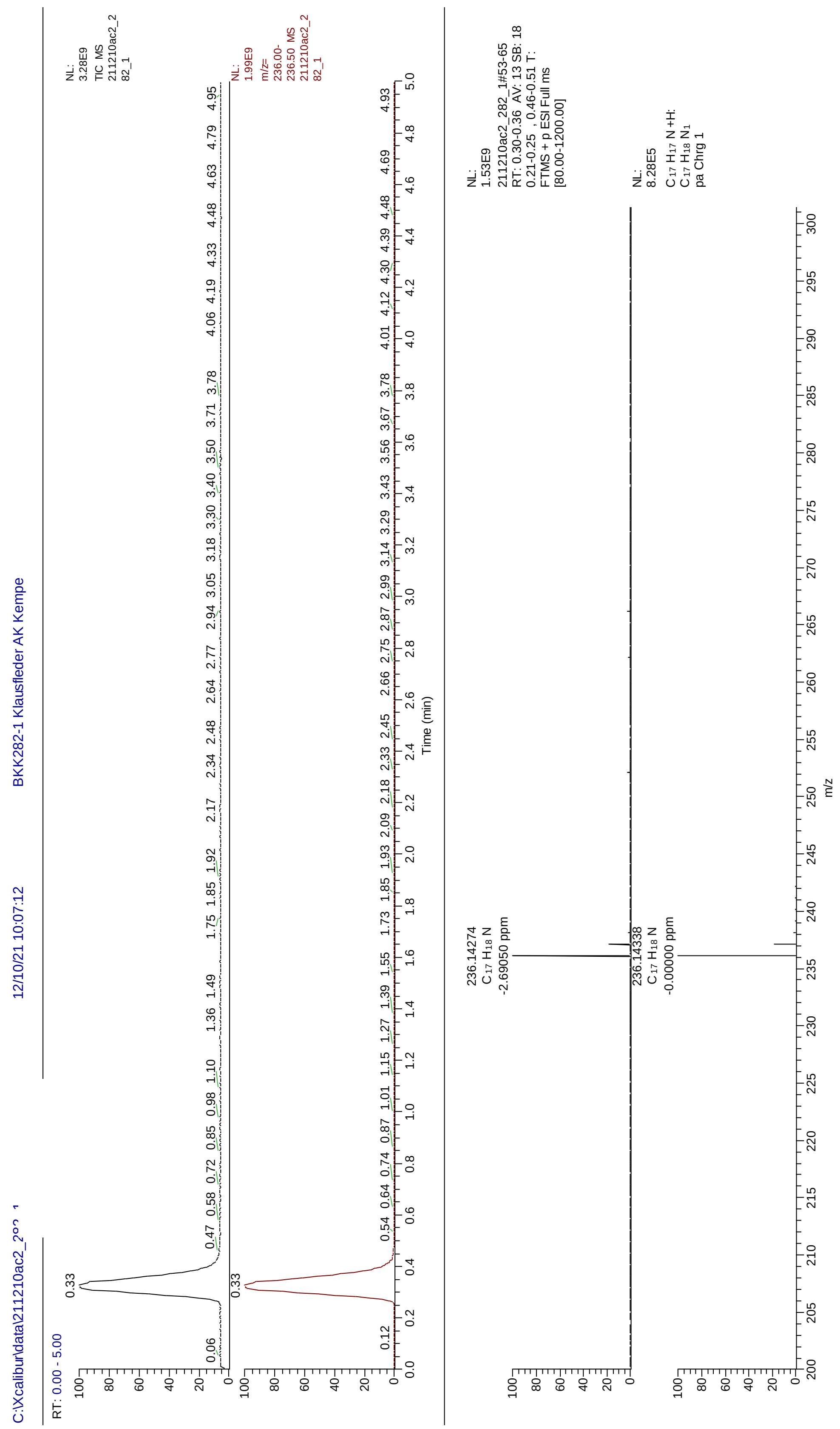

Figure S 90: HRMS-Spectra of compound (**3**).

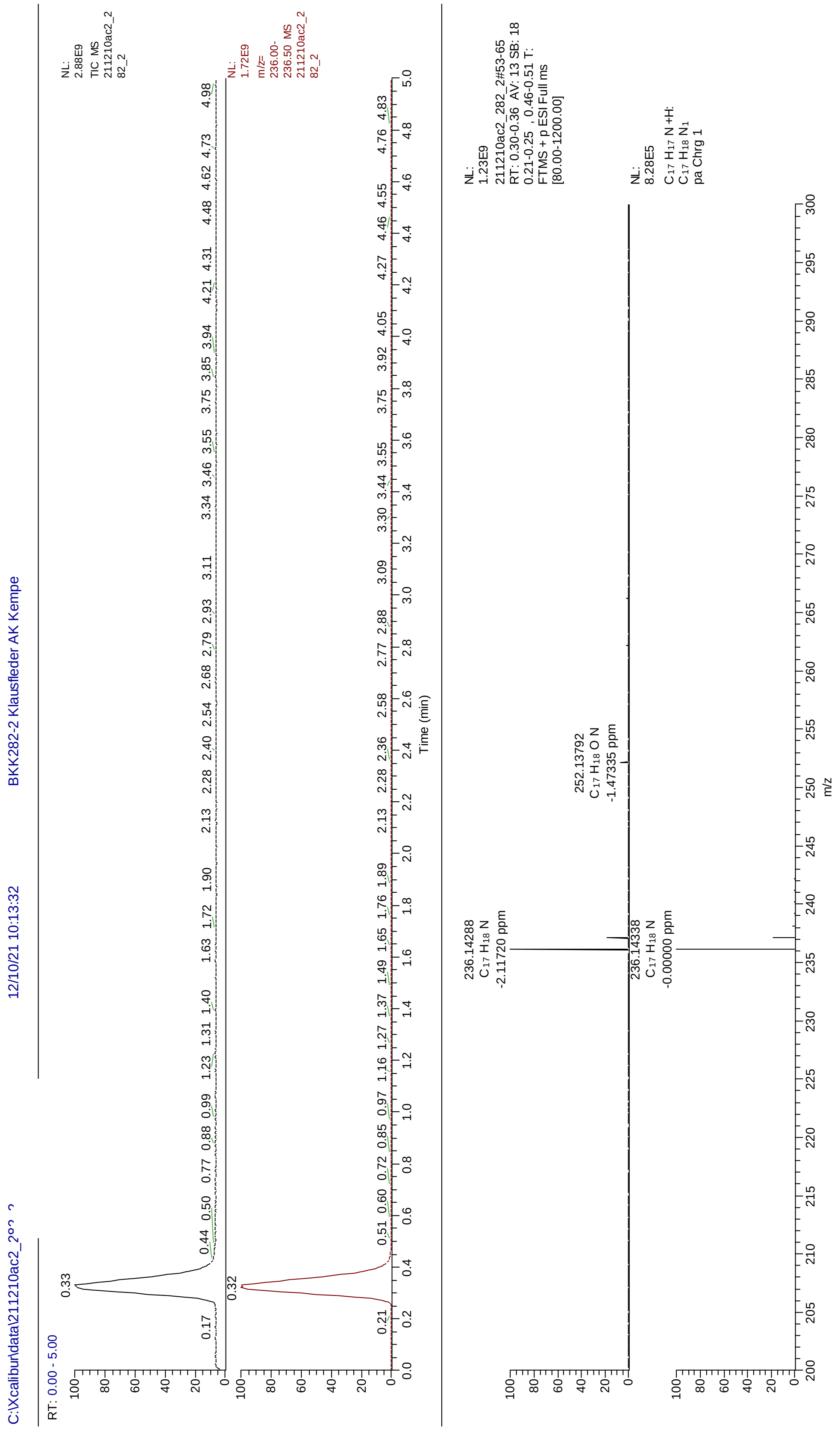

Figure S 91: HRMS-Spectra of compound (**5**).

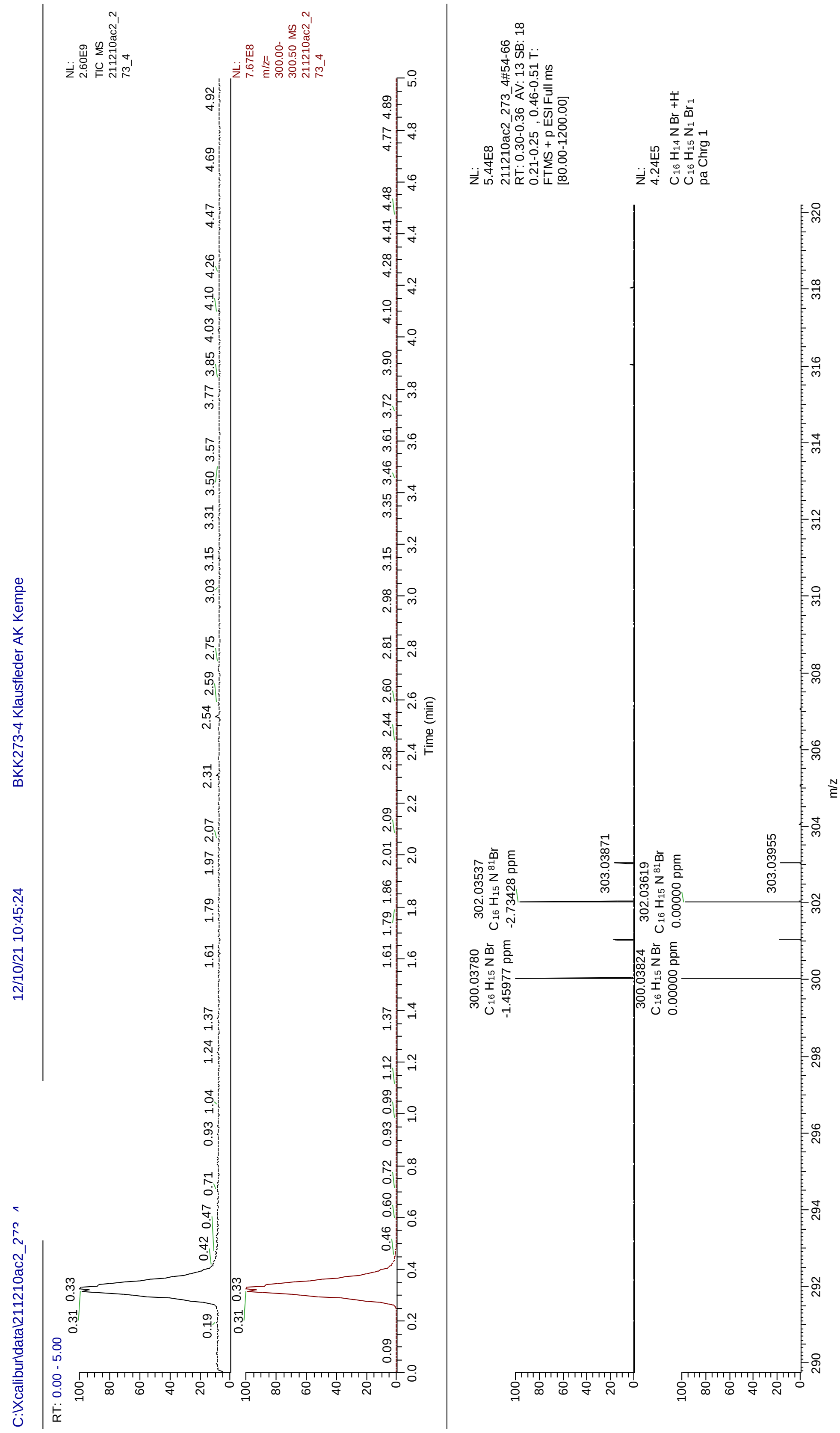

Figure S 92: HRMS-Spectra of compound (**6**).

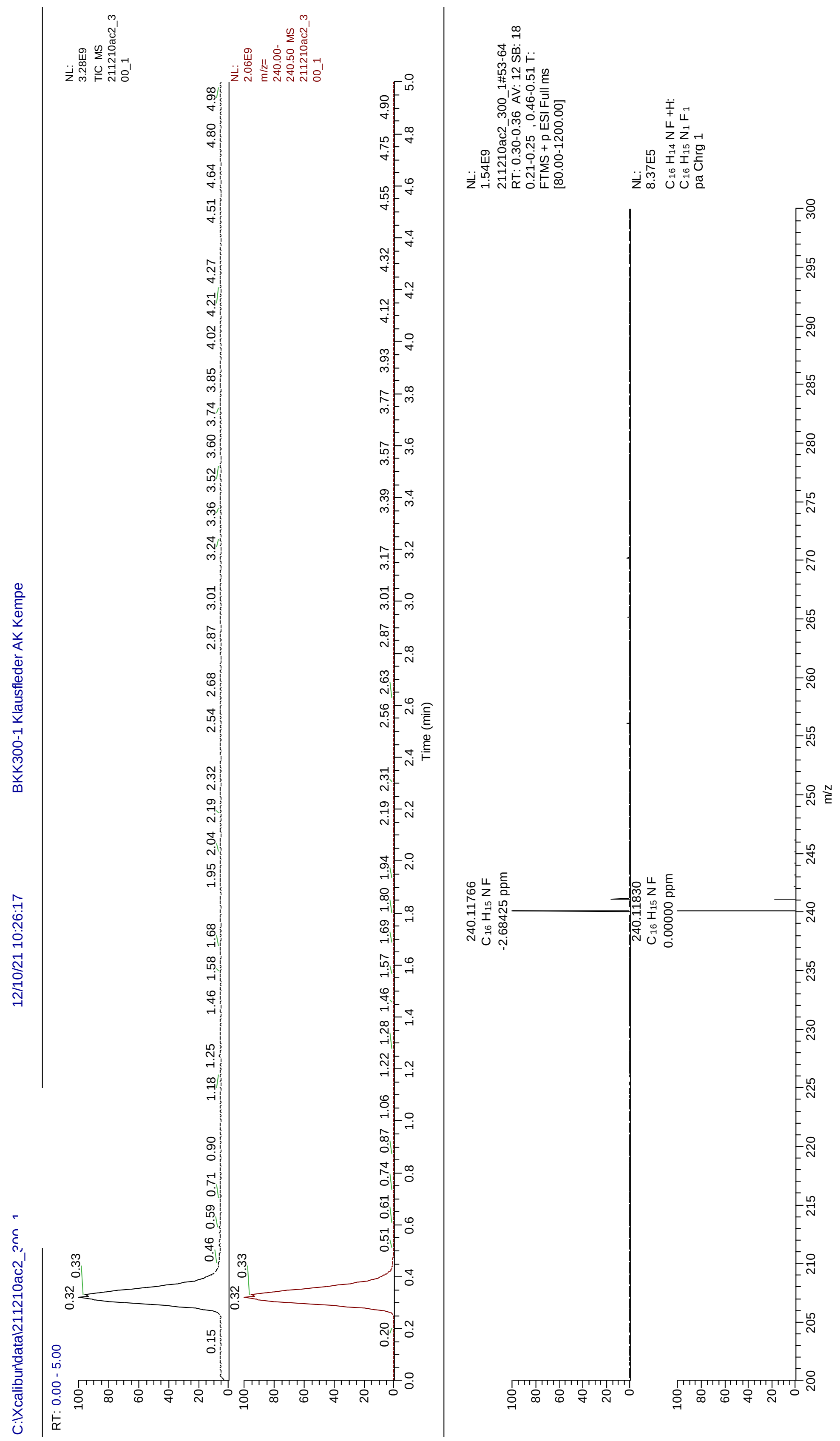

Figure S 93: HRMS-Spectra of compound (**7**).

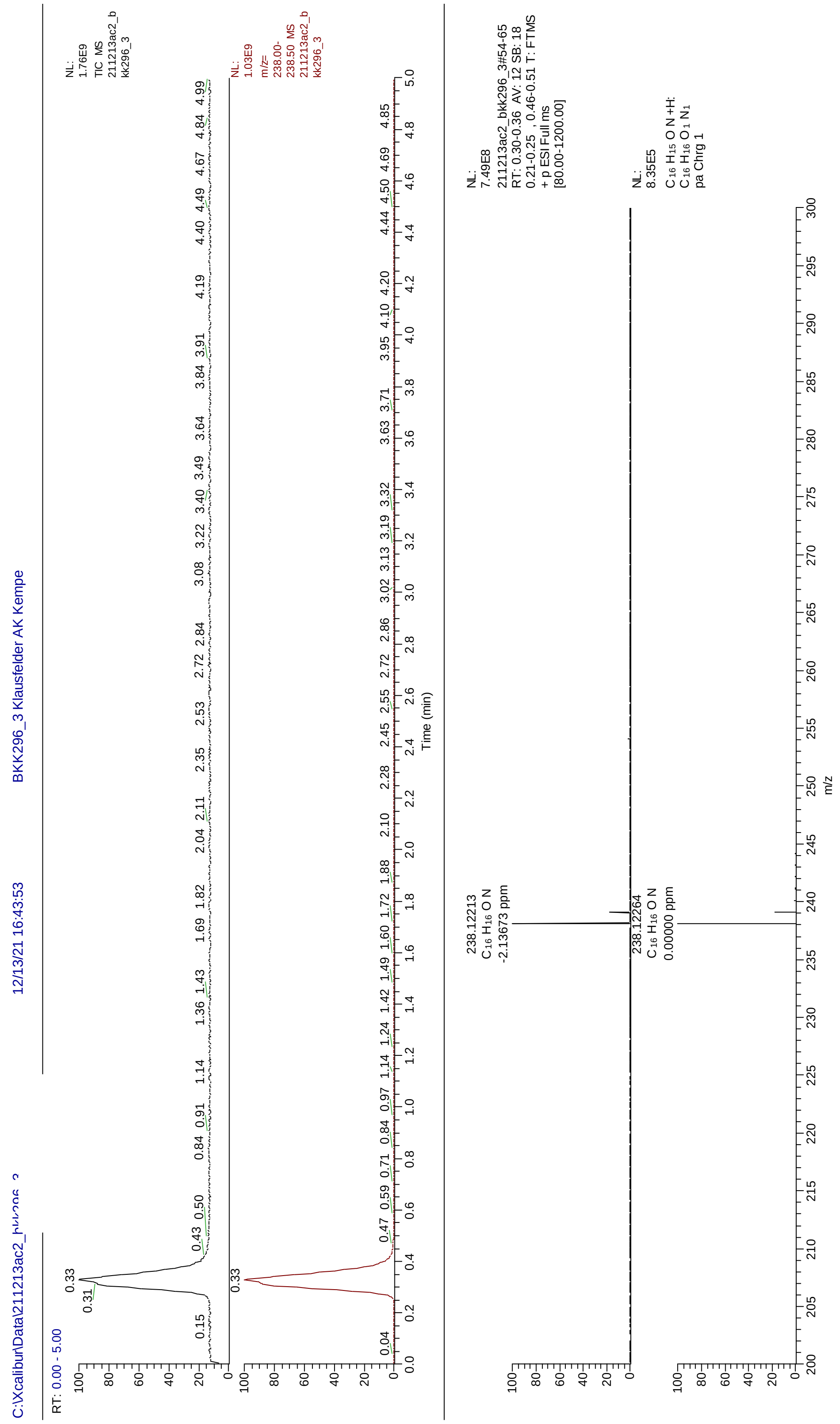

Figure S 94: HRMS-Spectra of compound (**9**).

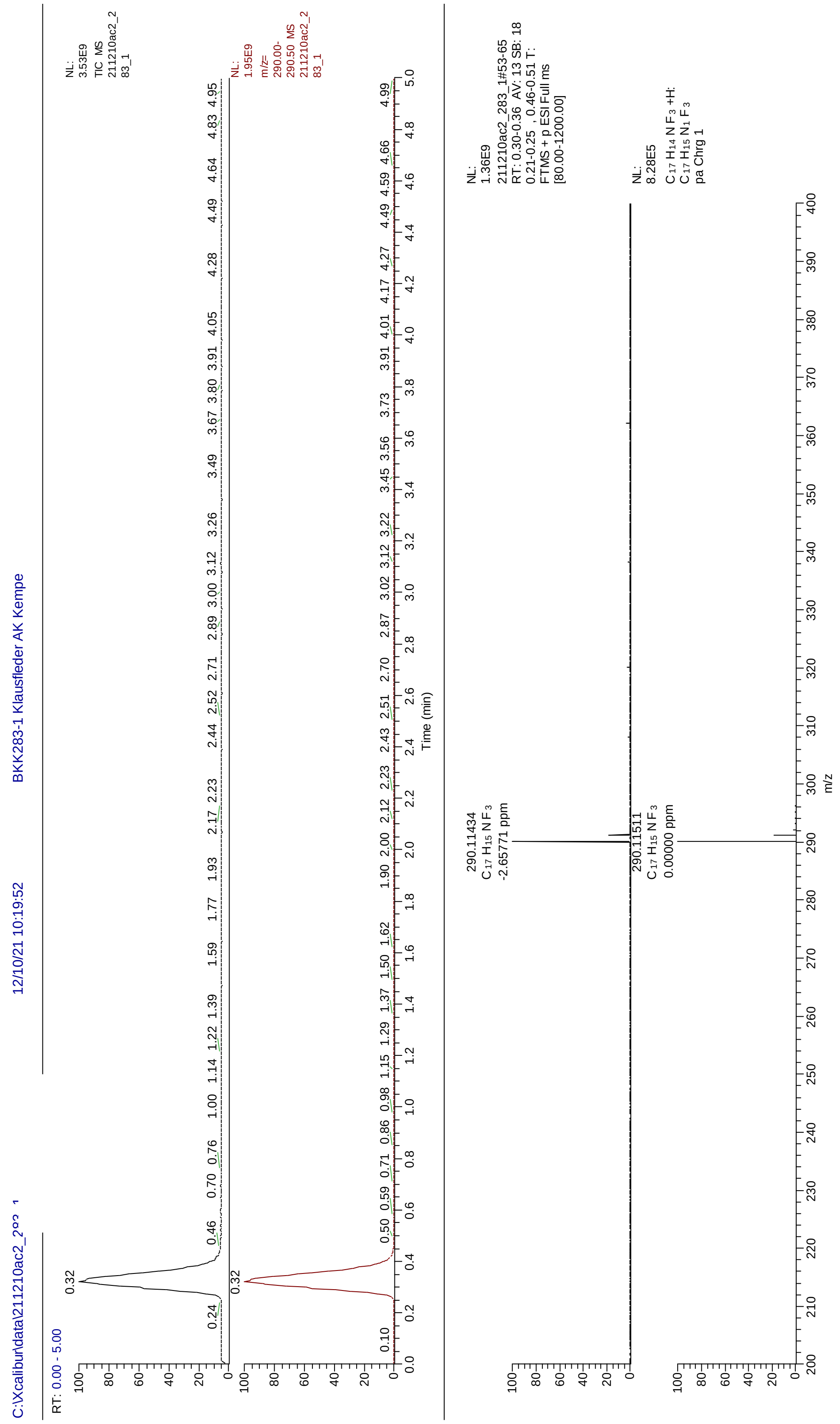

Figure S 95: HRMS-Spectra of compound (**11**).

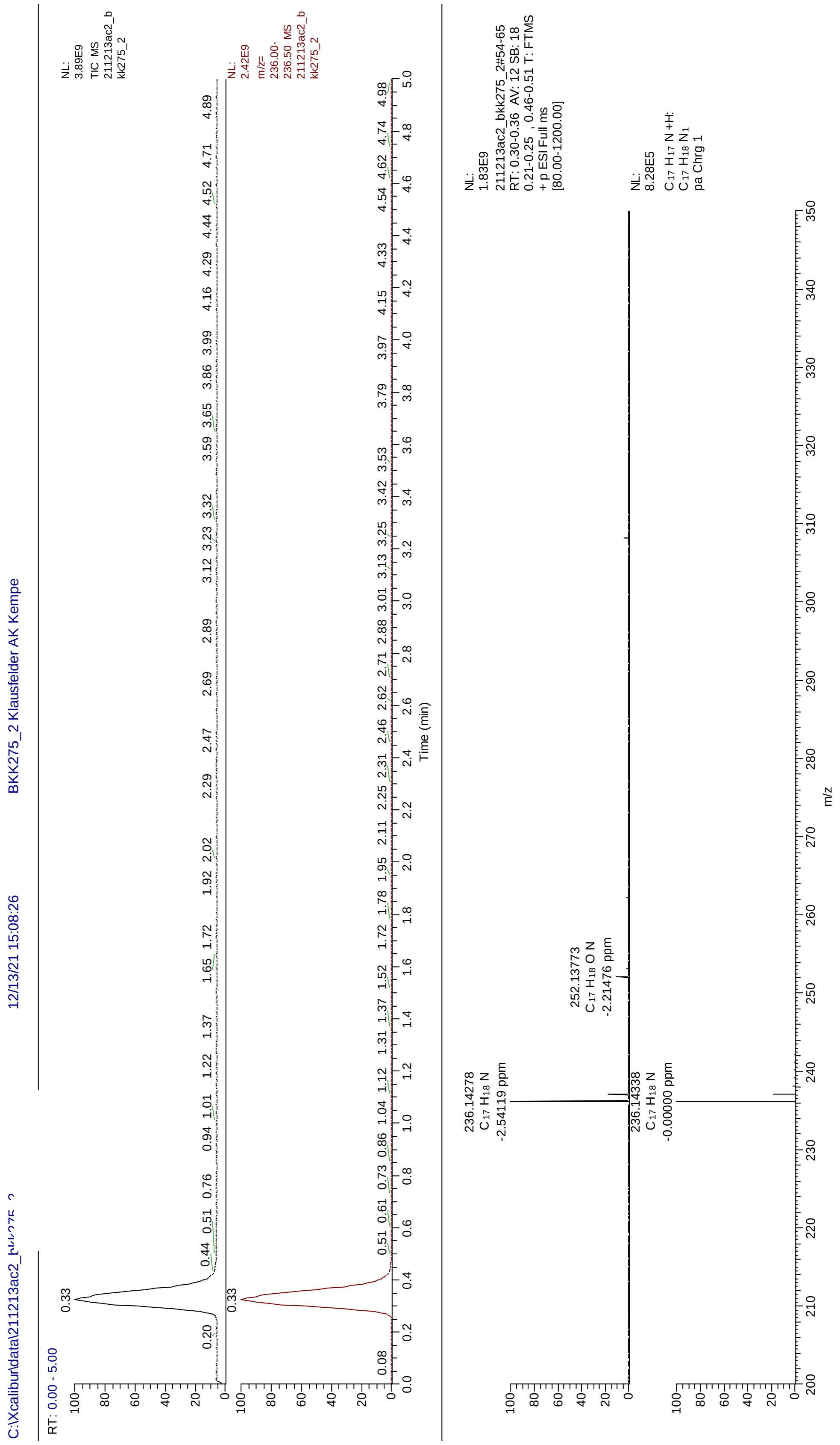

Figure S 96: HRMS-Spectra of compound (**12**).

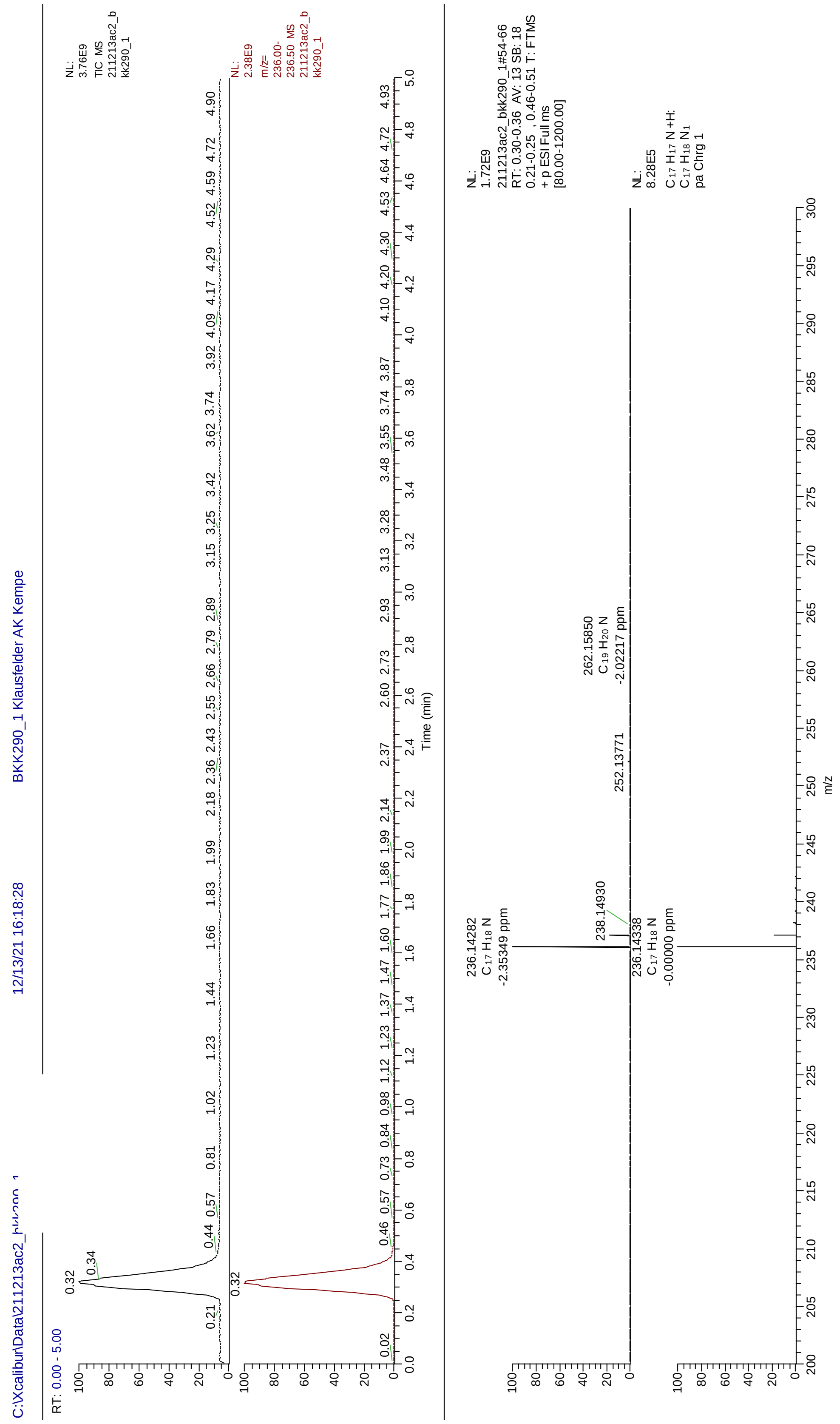

Figure S 97: HRMS-Spectra of compound (**13**).

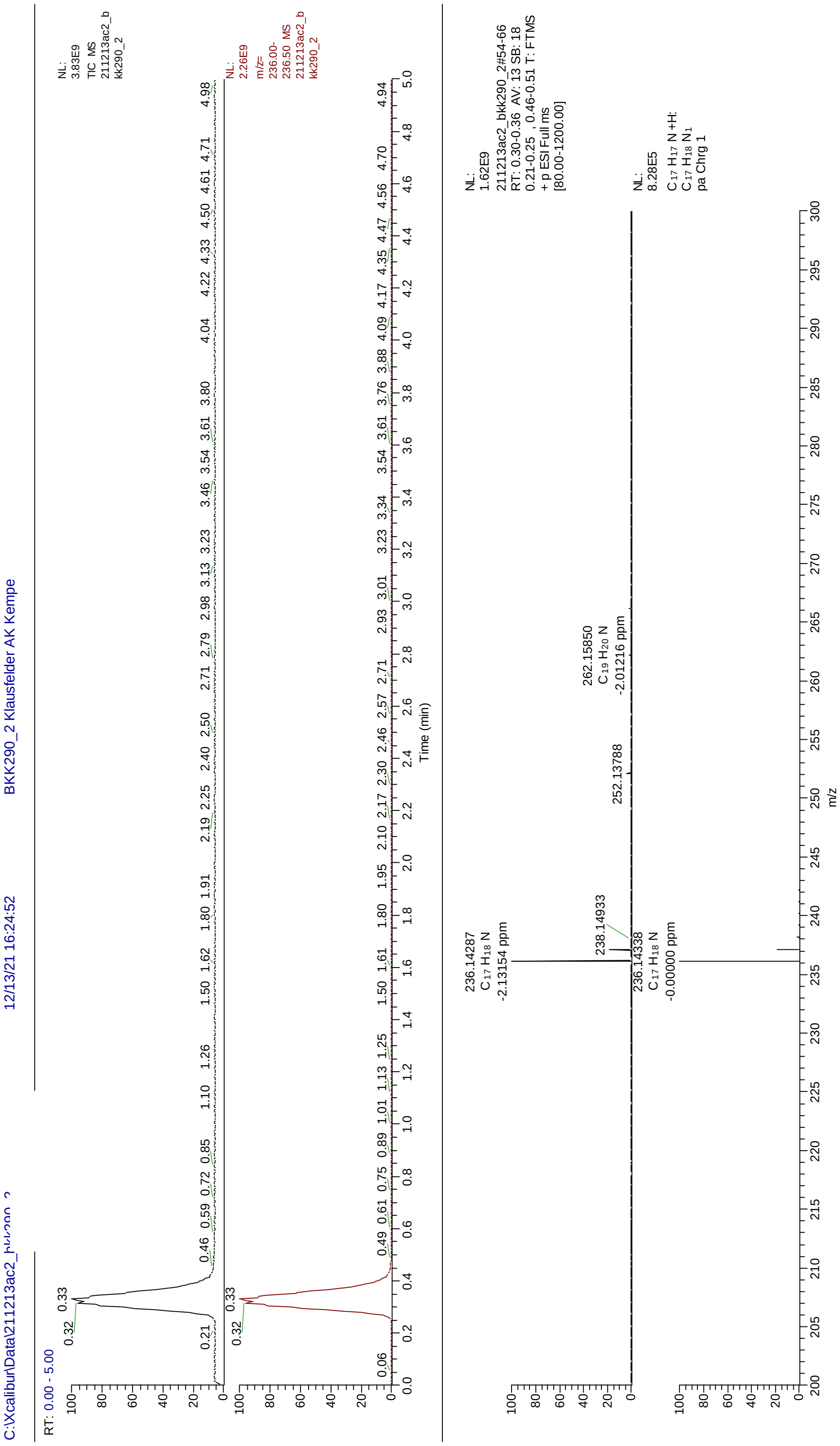

Figure S 98: HRMS-Spectra of compound (**14**).

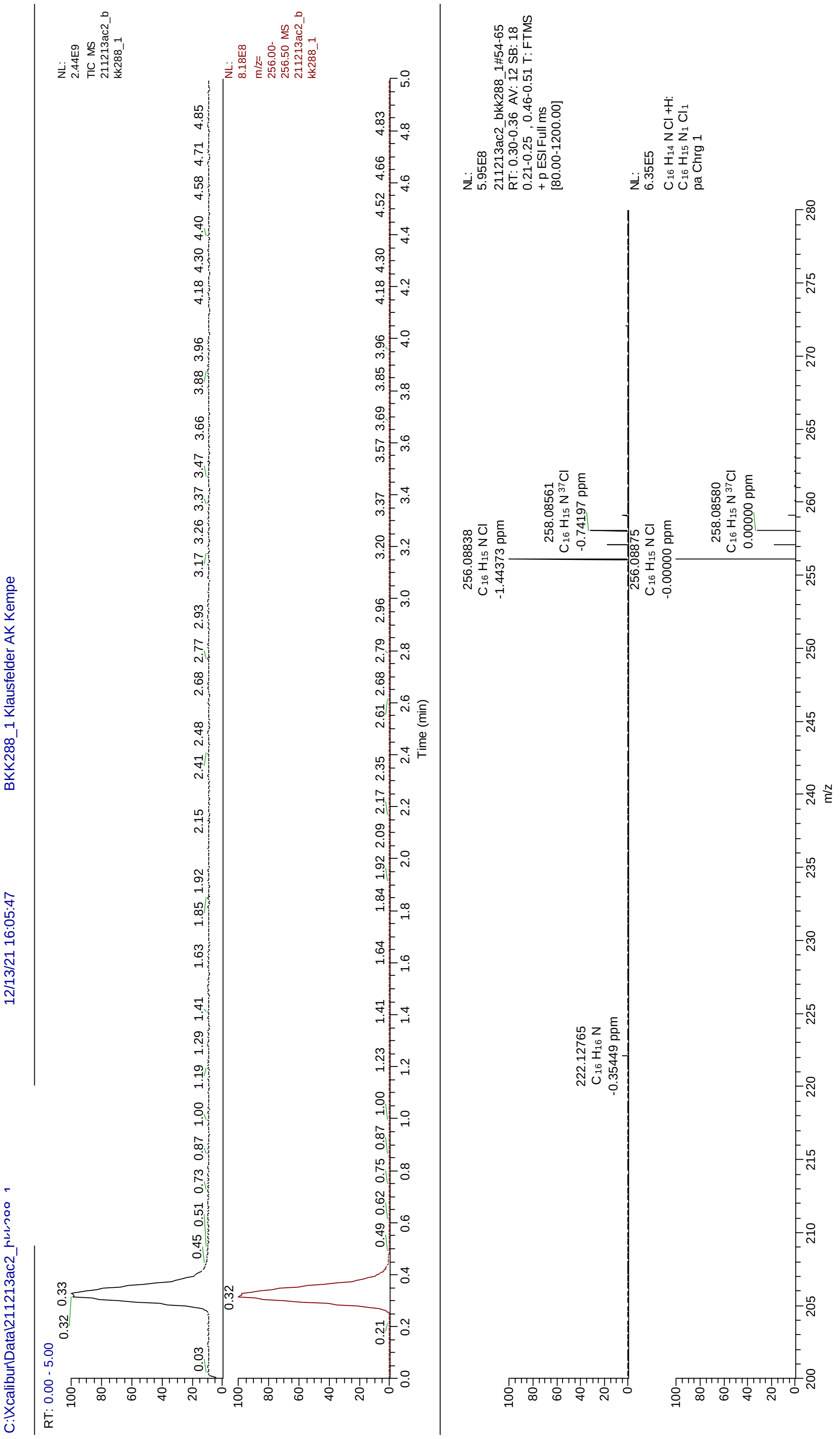

Figure S 99: HRMS-Spectra of compound (**15**).

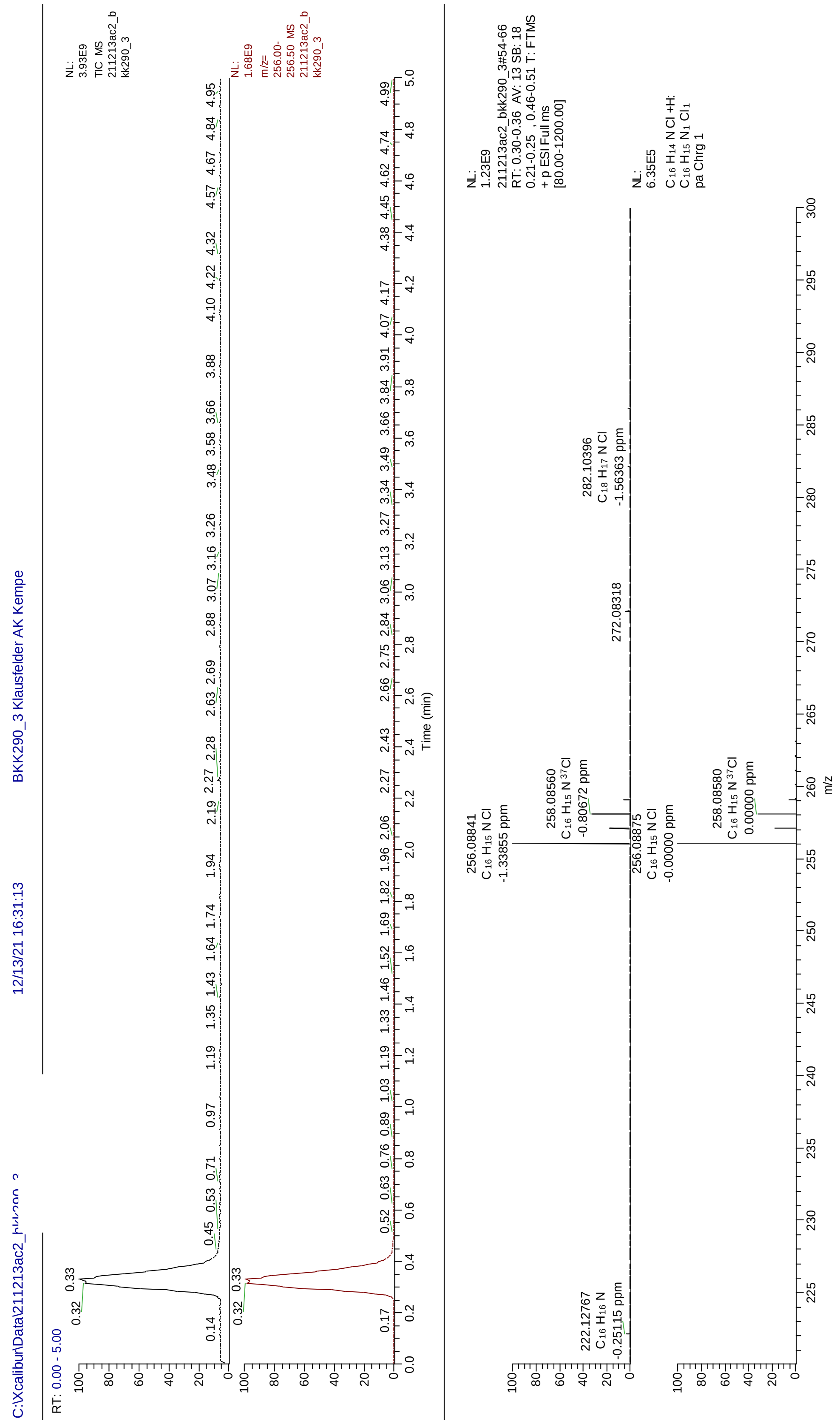

Figure S 100: HRMS-Spectra of compound (**17**).

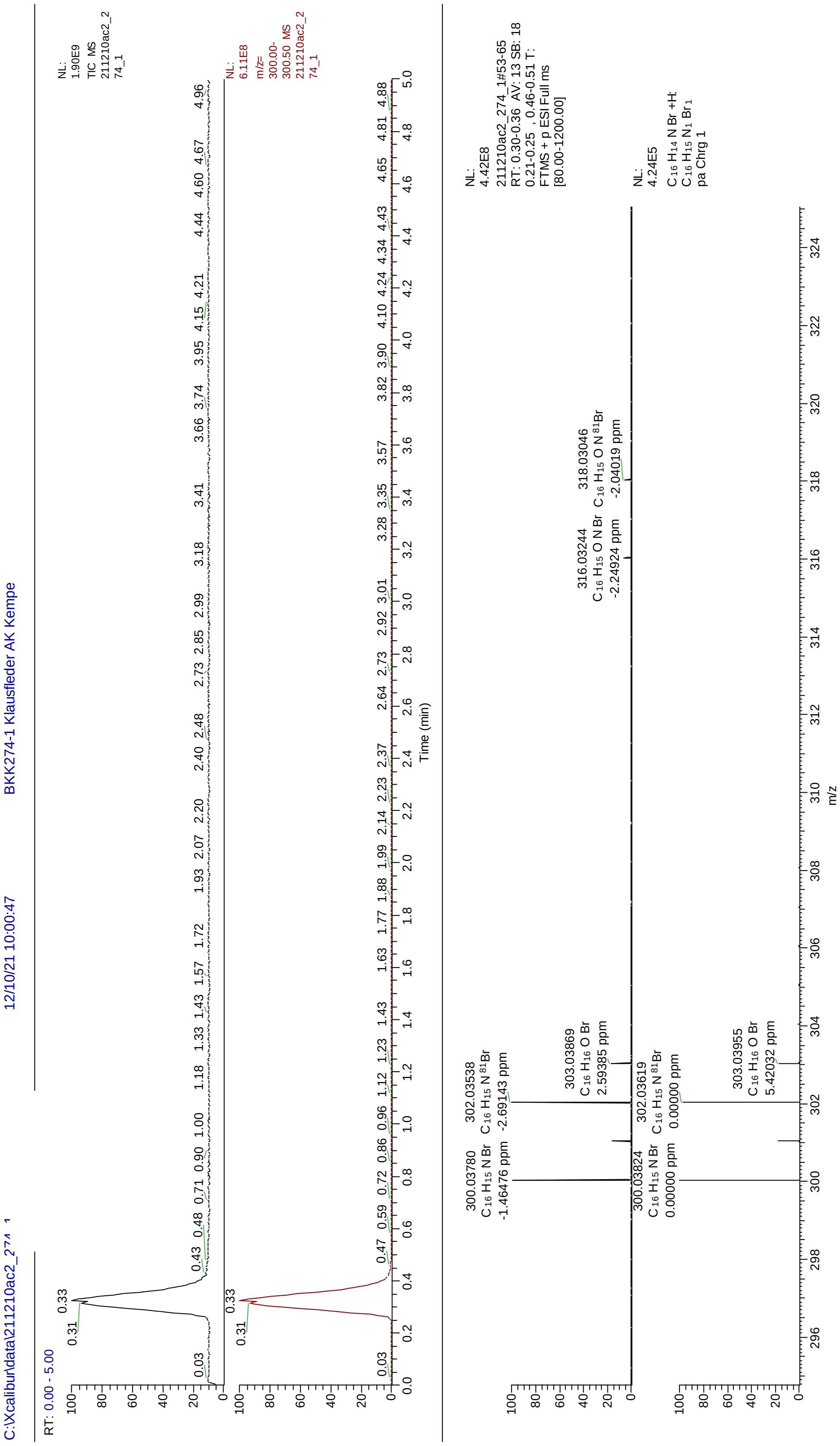

Figure S101: HRMS-Spectra of compound (**18**).

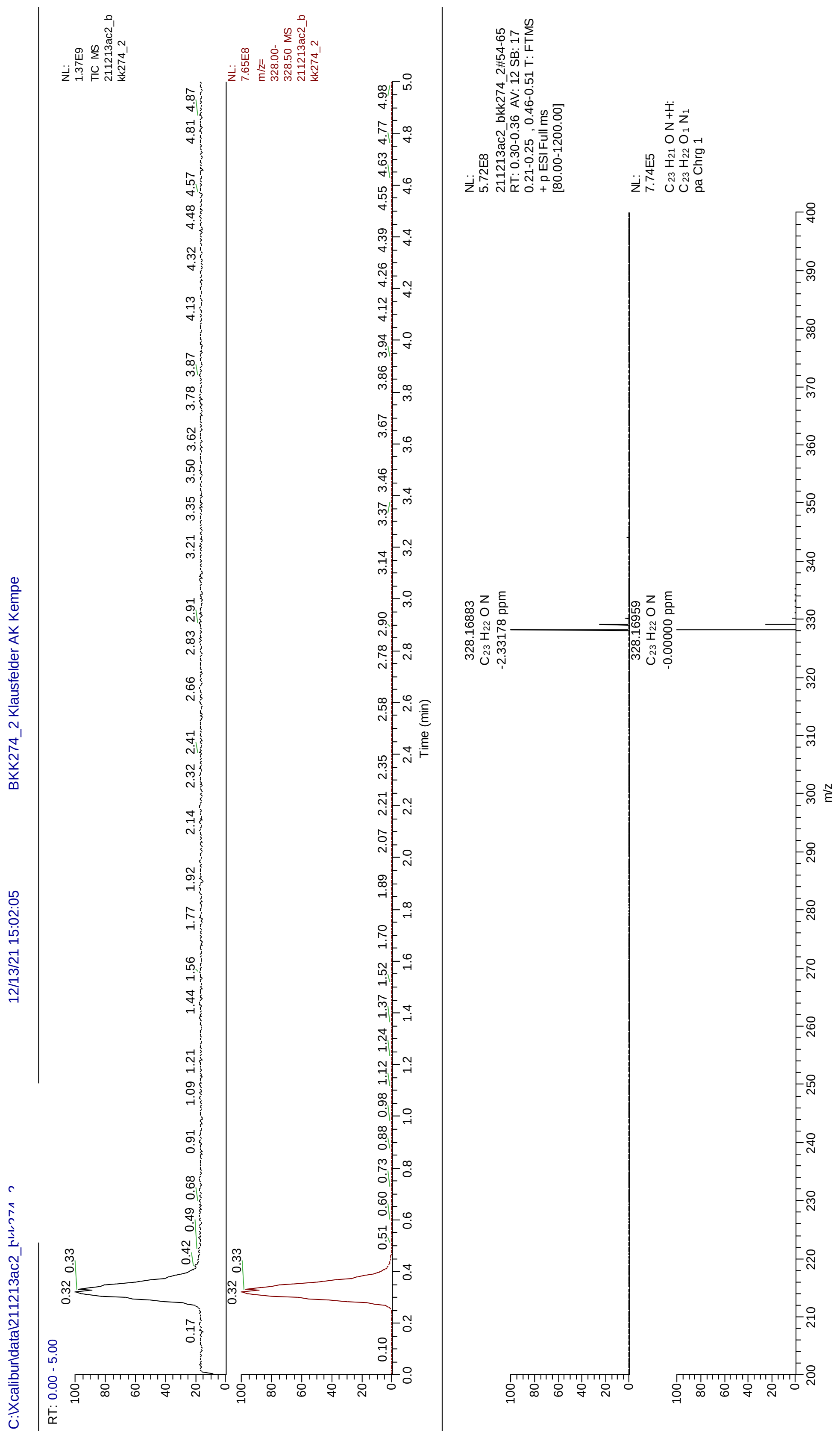

Figure S 102: HRMS-Spectra of compound (**21**).

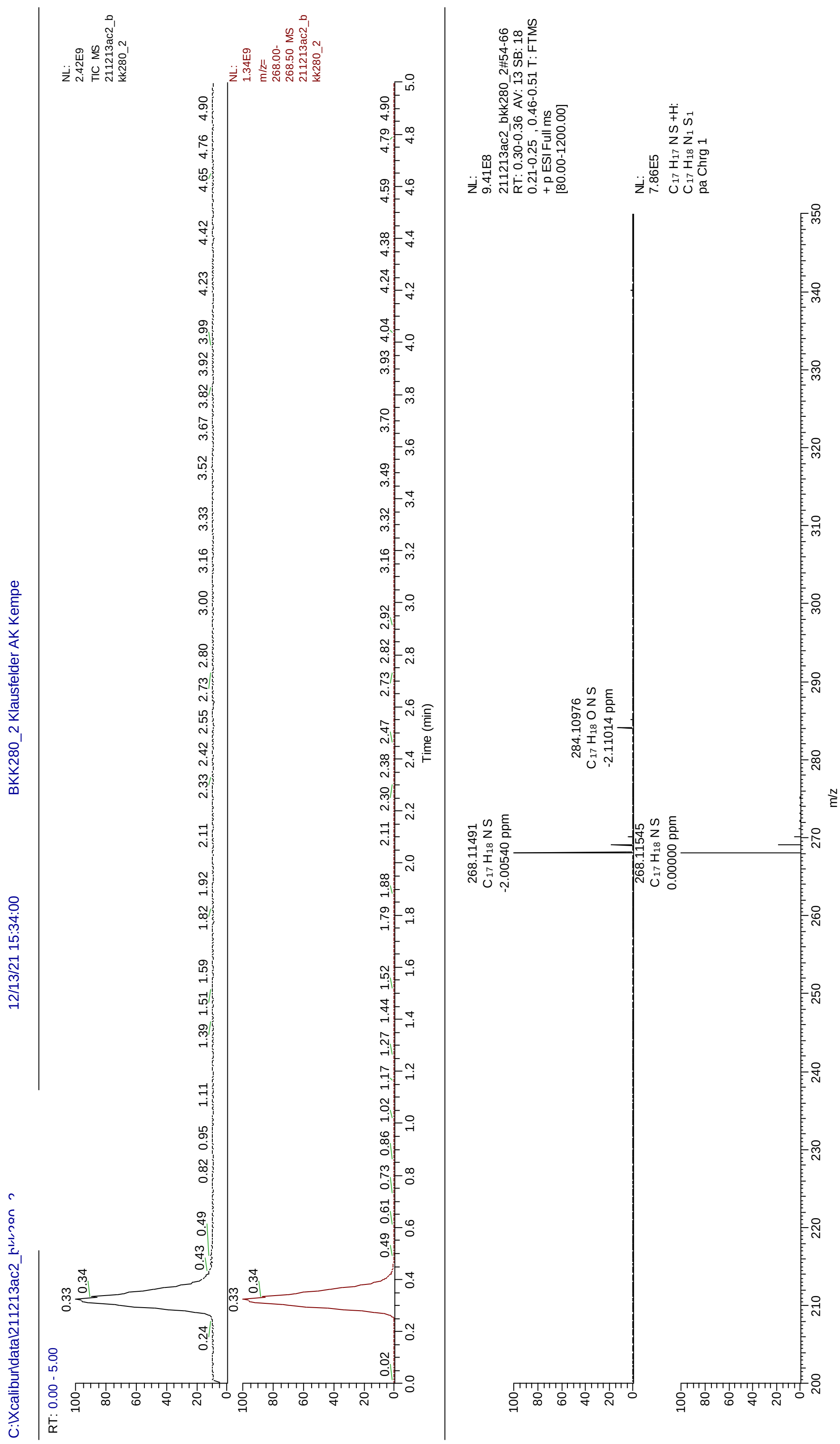

Figure S 103: HRMS-Spectra of compound (**22**).

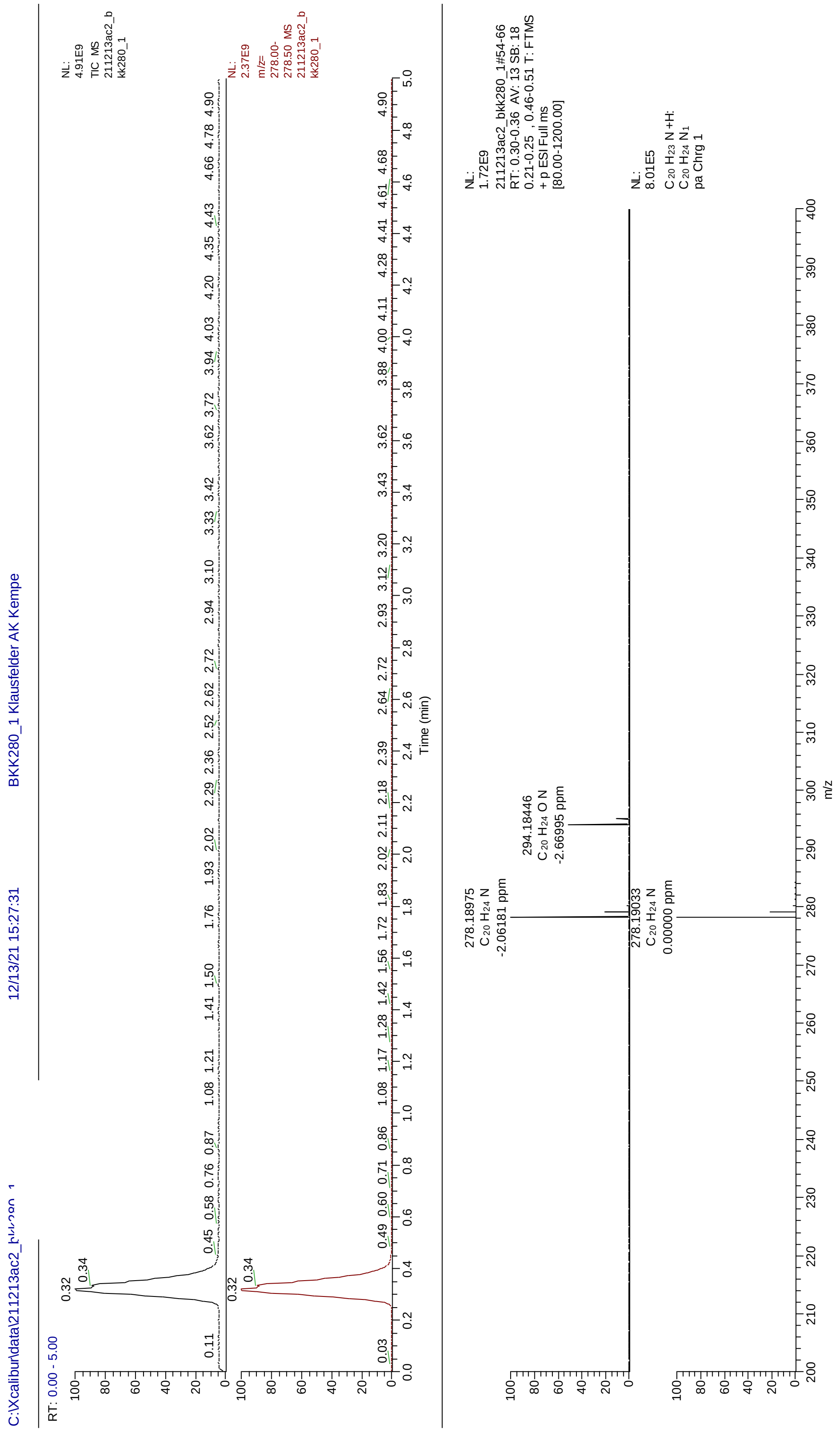

Figure S 104: HRMS-Spectra of compound (**23**).

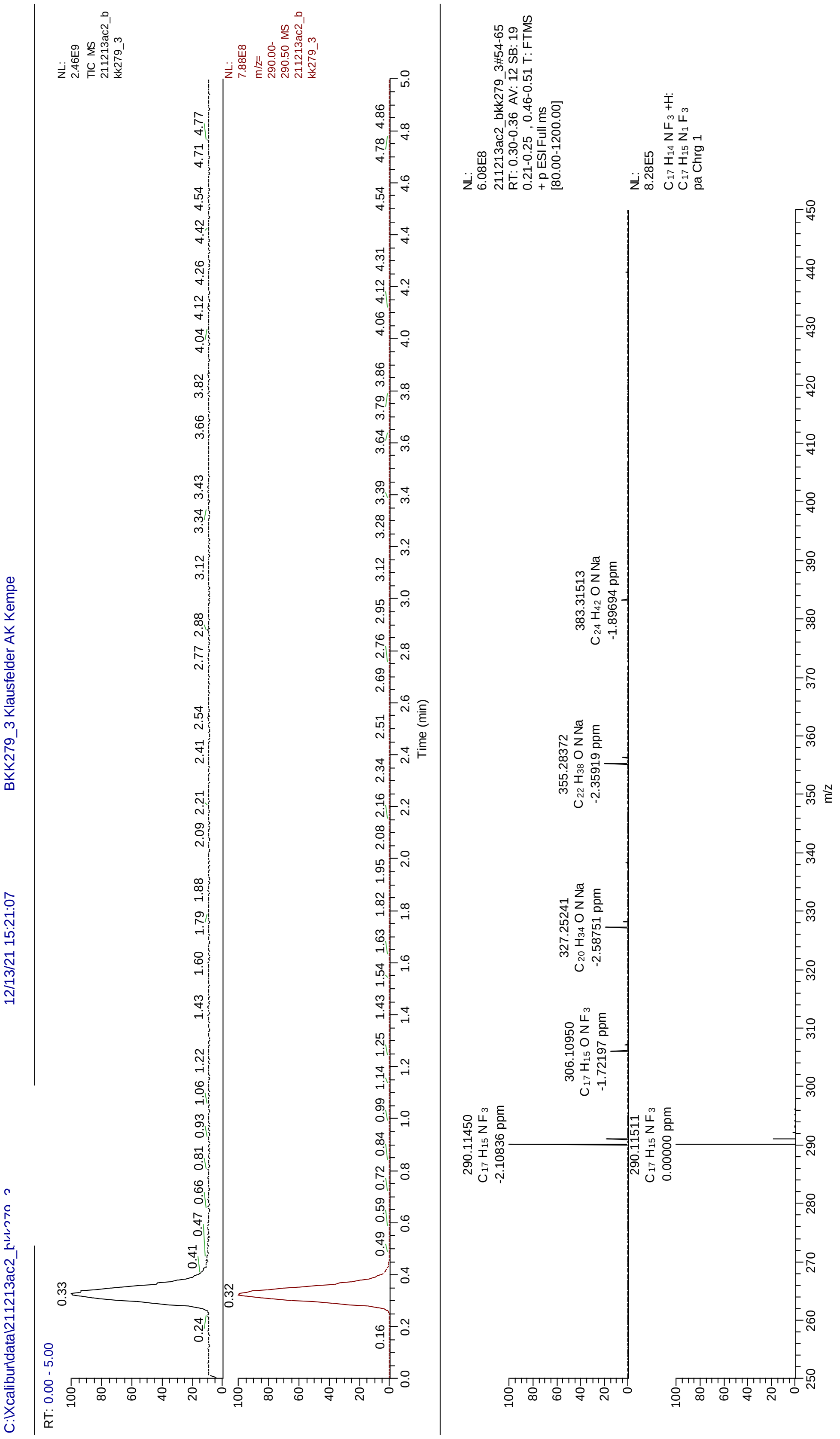

Figure S 105: HRMS-Spectra of compound (**24**).

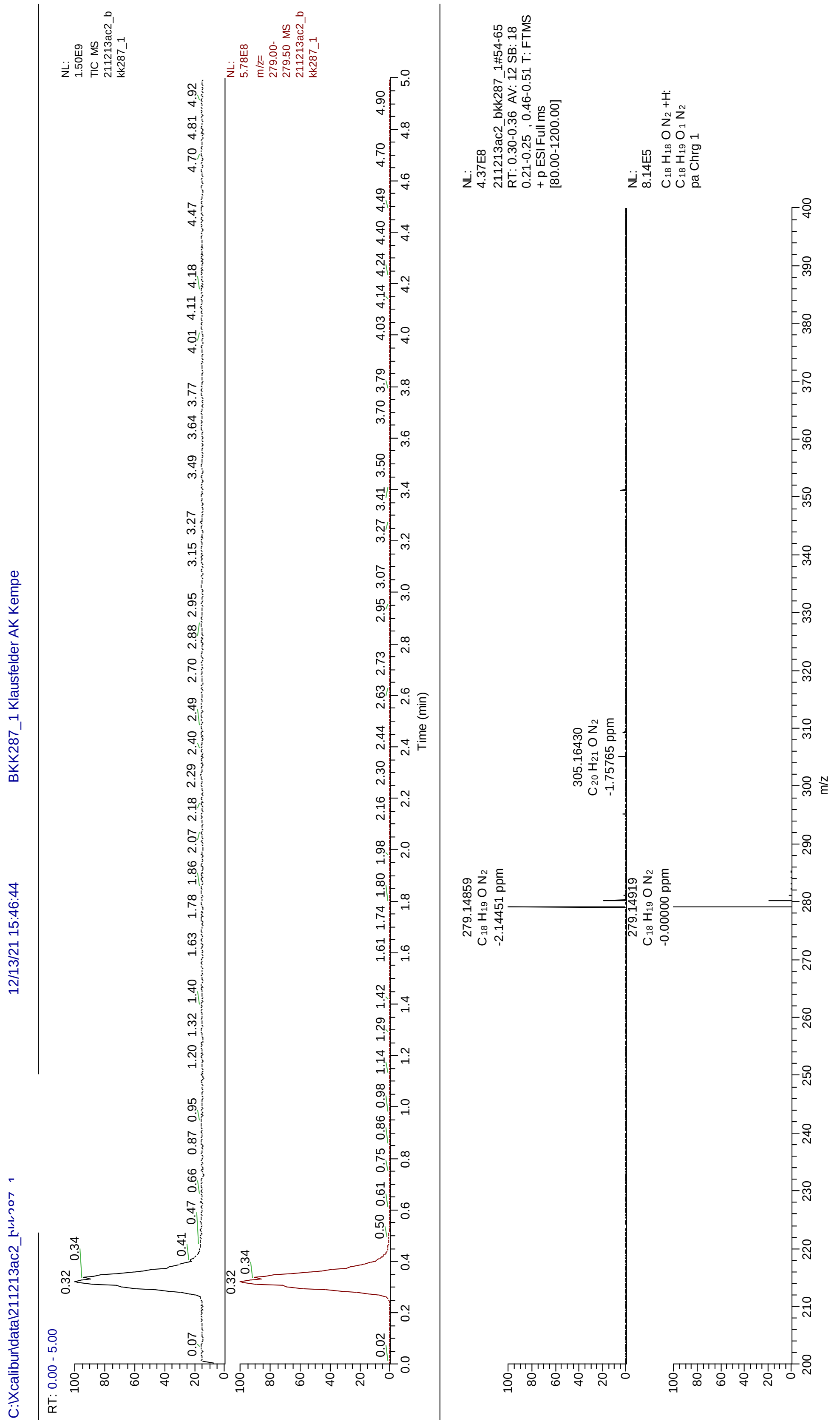

Figure S 106: HRMS-Spectra of compound (**25**).

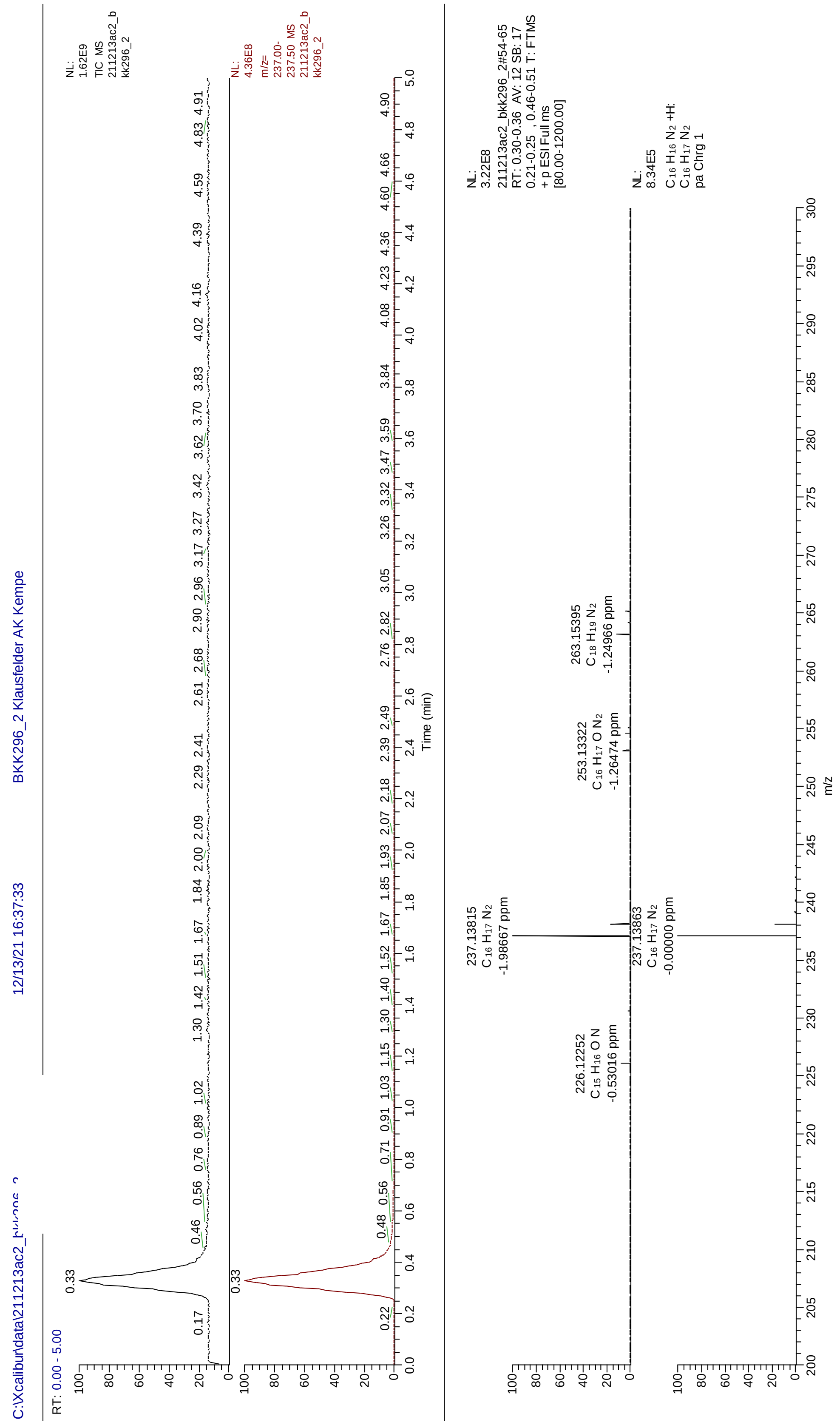

Figure S 107: HRMS-Spectra of compound (**26**).

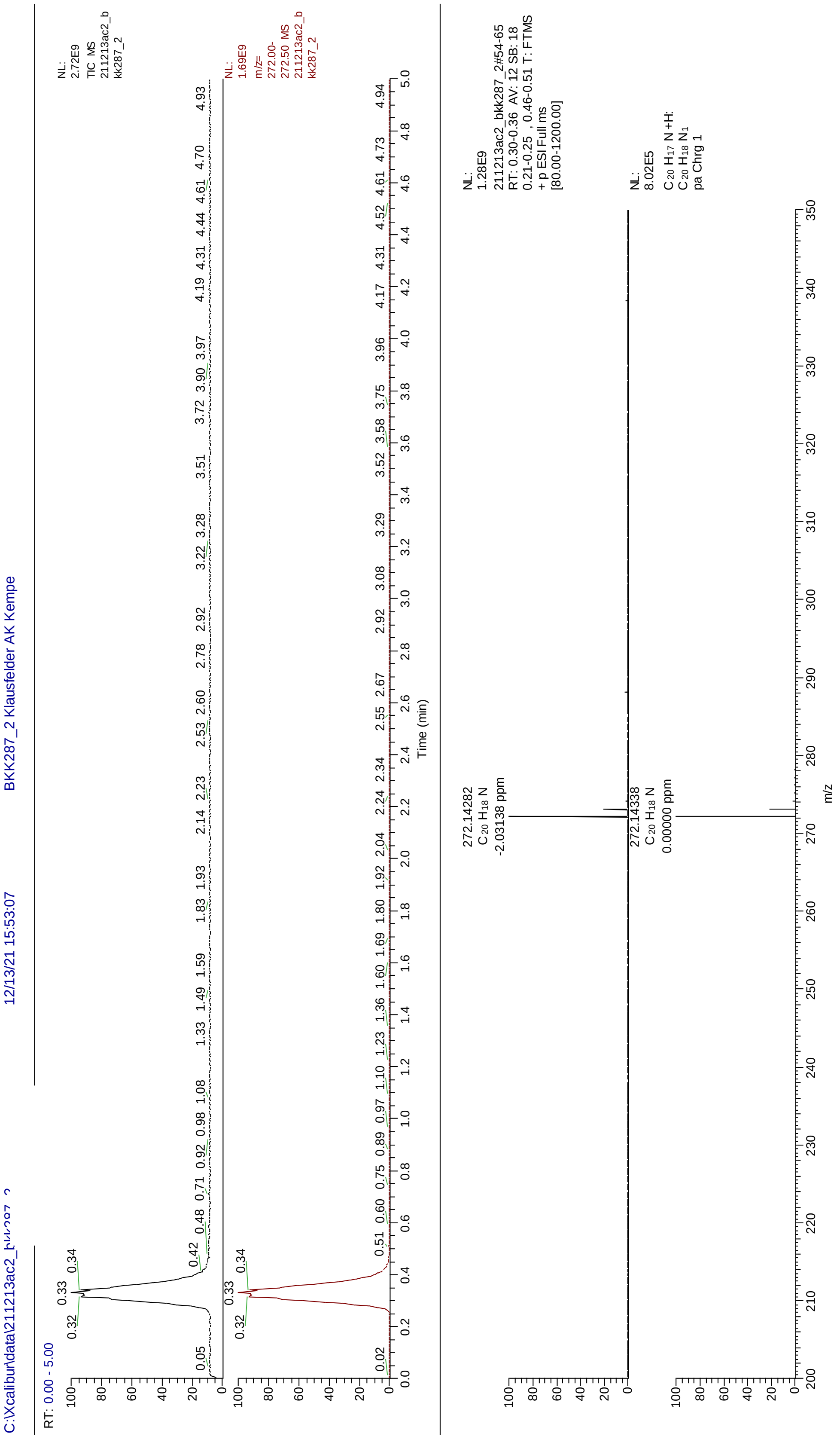

Figure S 108: HRMS-Spectra of compound (**27**).

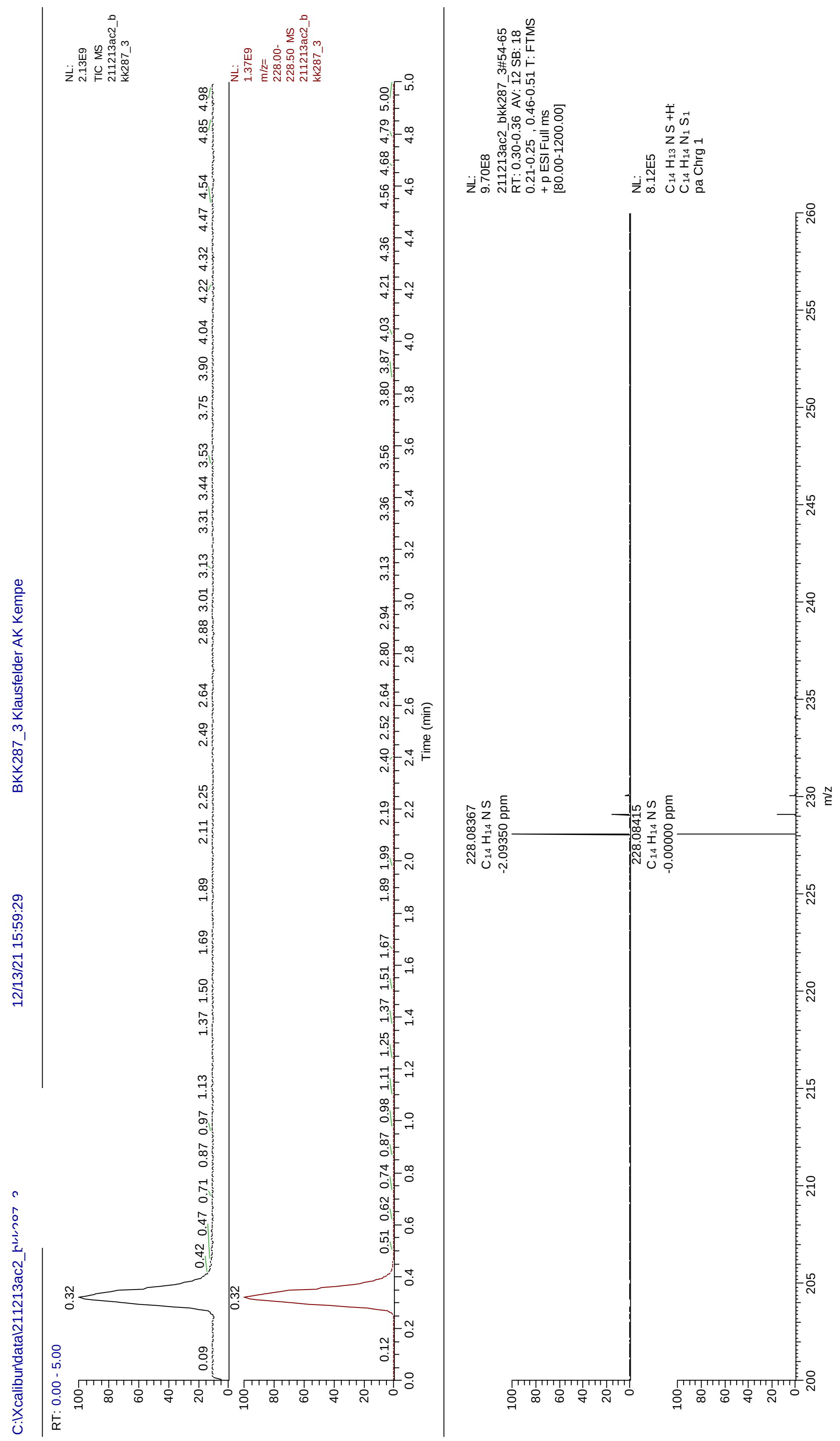

Figure S 109: HRMS-Spectra of compound (**28**).

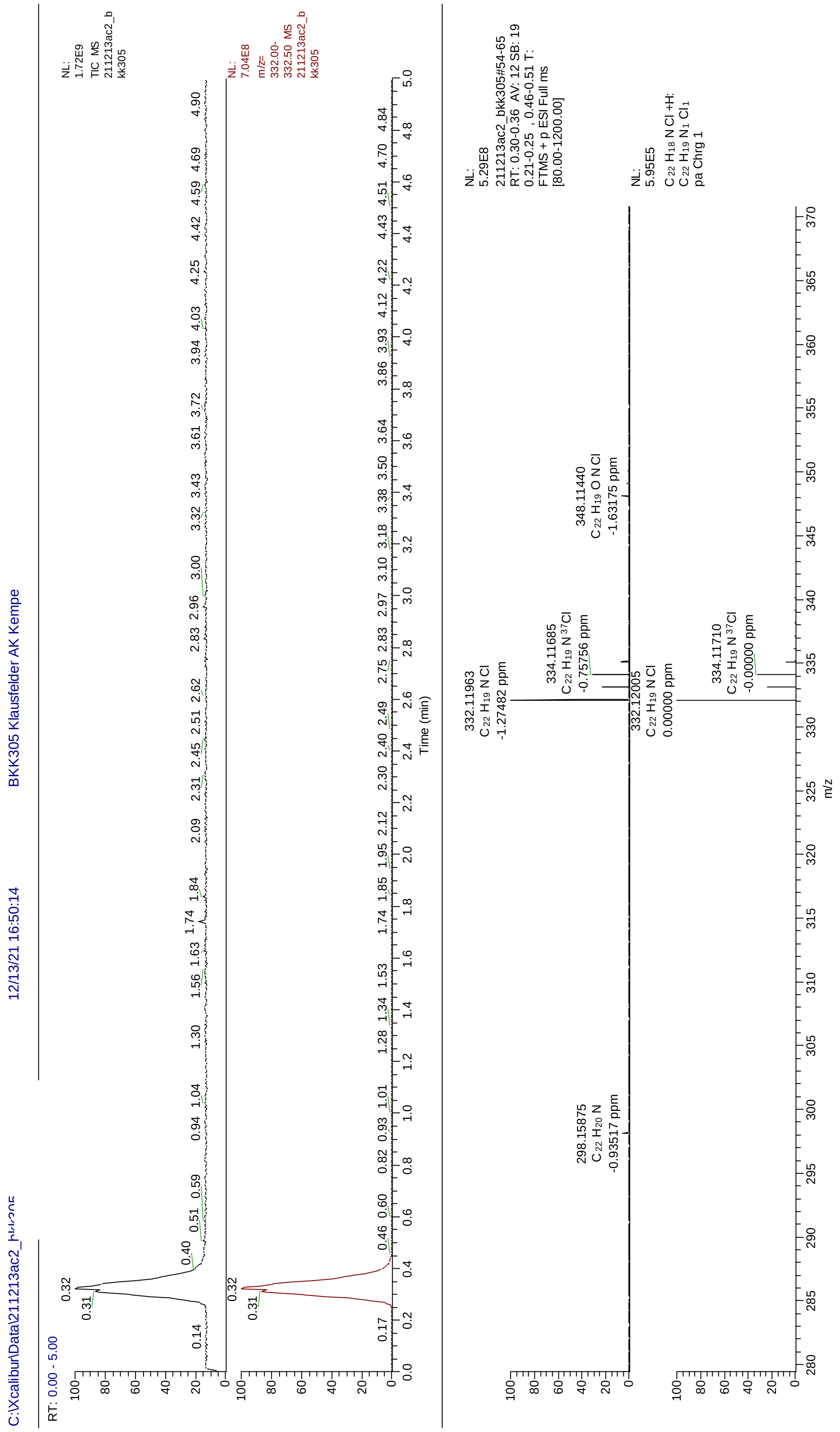

Figure S 110: HRMS-Spectra of compound (**31**).

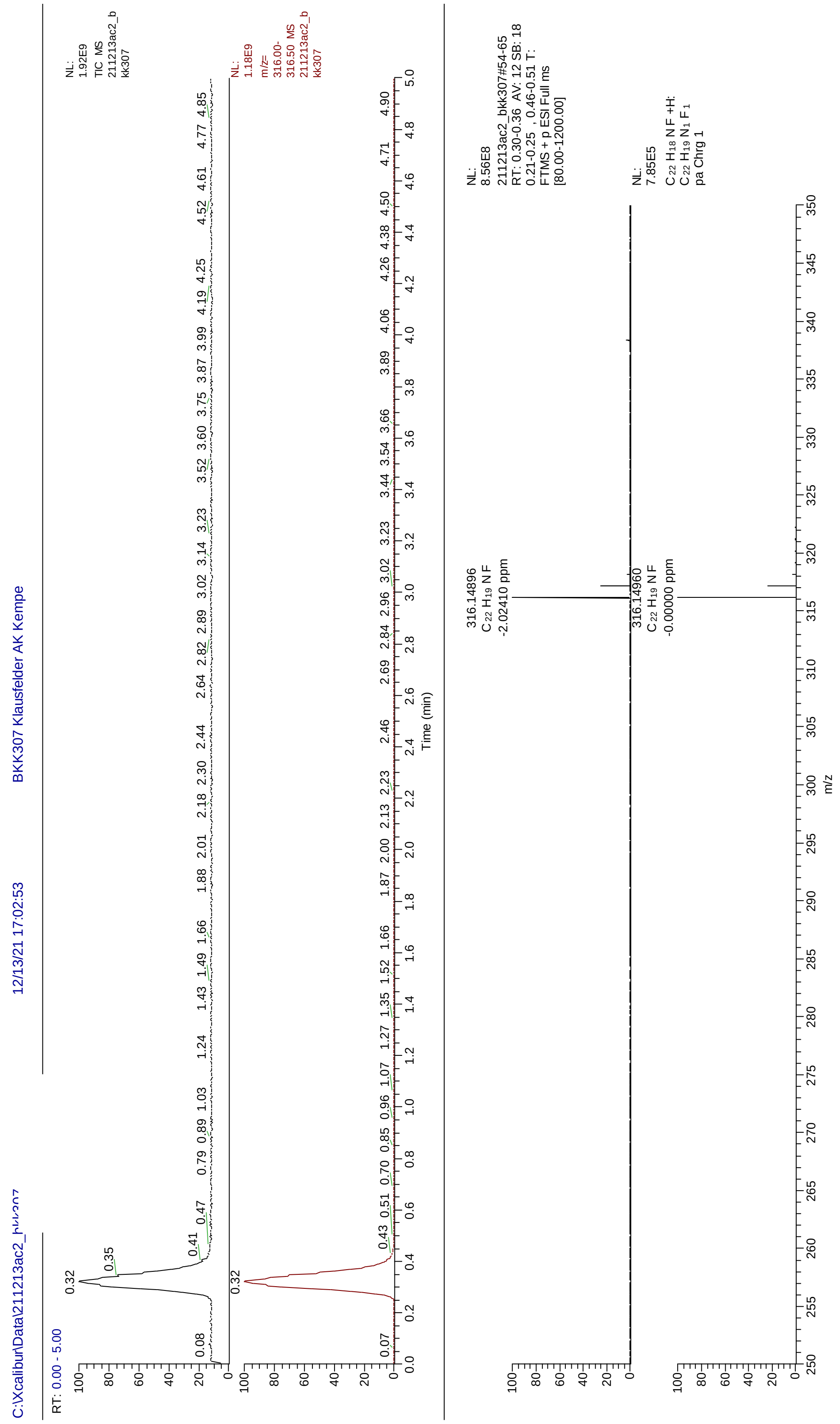

Figure S 111: HRMS-Spectra of compound (**32**).

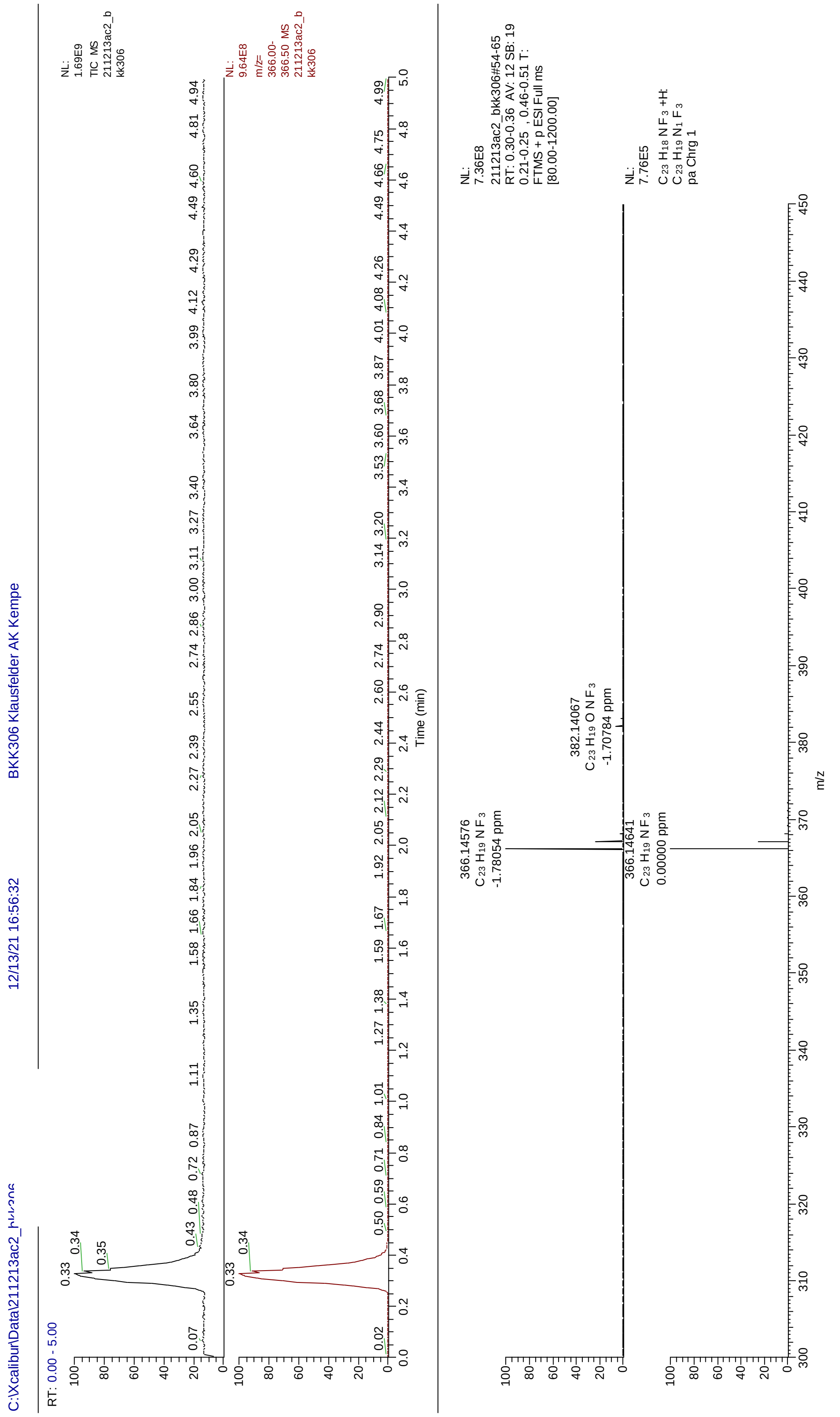

Figure S 112: HRMS-Spectra of compound (**33**).

12. Supplementary References

[1] H.-R. Wen, S.-J. Liu, X.-R. Xie, J. Bao, C.-M. Liu, J.-L. Chen, *Inorg. Chim. Acta* **2015**, *435*, 274.

[2] J. S. Elias, M. Risch, L. Giordano, A. N. Mansour, Y. Shao-Horn, *J. Am. Chem. Soc.* **2014**, *136*, 17193.

[3] C. Bäumler, C. Bauer, R. Kempe, *ChemSusChem* **2020**, *13*, 3110.

[4] a) B. Ding, Z. Zhang, Y. Liu, M. Sugiya, T. Imamoto, W. Zhang, *Org. Lett.* **2013**, *15*, 3690; b) G. Song, R. Liu, G. He, S. Yuan, H. Zhu, *Asian J. Org. Chem.* **2015**, *4*, 346; c) M. Cabrera, M. Simoens, G. Falchi, M. L. Lavaggi, O. E. Piro, E. E. Castellano, A. Vidal, A. Azqueta, A. Monge, A. L. de Ceráin et al., *Bioorg. Med. Chem.* **2007**, *15*, 3356; d) B. E. Moulton, A. K. Duhme-Klair, I. J. S. Fairlamb, J. M. Lynam, A. C. Whitwood, *Organometallics* **2007**, *26*, 6354; e) F. Hayat, A. Salahuddin, S. Umar, A. Azam, *Eur. J. Med. Chem.* **2010**, *45*, 4669; f) A. Wilhelm, L. A. Lopez-Garcia, K. Busschots, W. Fröhner, F. Maurer, S. Boettcher, H. Zhang, J. O. Schulze, R. M. Biondi, M. Engel, *J. Med. Chem.* **2012**, *55*, 9817; g) A. H. Bansode, G. Suryavanshi, *ACS omega* **2019**, *4*, 9636; h) R. Yoshii, A. Nagai, Y. Chujo, *J. Polym. Sci. A Polym. Chem.* **2010**, *48*, 5348.

[5] R. Gresser, H. Hartmann, M. Wrackmeyer, K. Leo, M. Riede, *Tetrahedron* **2011**, *67*, 7148.

[6] S. Schutting, T. Jokic, M. Strobl, S. M. Borisov, D. de Beer, I. Klimant, *J. Mater. Chem. C.* **2015**, *3*, 5474.

[7] P. Marcé, J. Lynch, A. J. Blacker, J. M. J. Williams, *Chem. Commun.* **2016**, *52*, 1013.

[8] H. Dai, H. Guan, *ACS Catal.* **2018**, *8*, 9125.

[9] W. Chen, J. Jiang, J. Gao, J. Chen, W. Shen, *Chin. Chem. Lett.* **1991**, *2*, 439.

[10] Y. Liang, D. Dong, Y. Lu, Y. Wang, W. Pan, Y. Chai, Q. Liu, *Synthesis* **2006**, *2006*, 3301.

[11] F. Cardona, J. Rocha, A. M. Silva, S. Guieu, *Dyes and Pigments* **2014**, *111*, 16.

[12] E. Elamparuthi, S. Sarathkumar, S. Girija, V. Anbazhagan, *Tetrahedron Lett.* **2014**, *55*, 3992.

[13] V. Dragisich, W. D. Wulff, K. Hogsteen, *Organometallics* **1990**, *9*, 2867.
